# The Flexibility of Oligosaccharides Unveiled Through Residual Dipolar Coupling Analysis

**DOI:** 10.3389/fmolb.2021.784318

**Published:** 2021-11-10

**Authors:** Ana Poveda, Giulio Fittolani, Peter H. Seeberger, Martina Delbianco, Jesús Jiménez-Barbero

**Affiliations:** ^1^ CICbioGUNE, Basque Research and Technology Alliance (BRTA), Derio, Spain; ^2^ Department of Biomolecular Systems, Max-Planck-Institute of Colloids and Interfaces, Potsdam, Germany; ^3^ Department of Chemistry and Biochemistry, Freie Universität Berlin, Berlin, Germany; ^4^ Ikerbasque, Basque Foundation for Science, Bilbao, Spain; ^5^ Department of Organic Chemistry II, Faculty of Science and Technology, University of the Basque Country, EHU-UPV, Leioa, Spain; ^6^ Centro de Investigacion Biomedica En Red de Enfermedades Respiratorias, Madrid, Spain

**Keywords:** glycans, NMR, RDC, 13C-labelling, automated glycan assembly

## Abstract

The intrinsic flexibility of glycans complicates the study of their structures and dynamics, which are often important for their biological function. NMR has provided insights into the conformational, dynamic and recognition features of glycans, but suffers from severe chemical shift degeneracy. We employed labelled glycans to explore the conformational behaviour of a β(1-6)-Glc hexasaccharide model through residual dipolar couplings (RDCs). RDC delivered information on the relative orientation of specific residues along the glycan chain and provided experimental clues for the existence of certain geometries. The use of two different aligning media demonstrated the adaptability of flexible oligosaccharide structures to different environments.

## Introduction

Saccharides also known as glycans, carbohydrates, or sugars are ubiquitous molecules in Nature, that serve in a large variety of roles, from plant cell construction and energy storage to mediation of key biomolecular recognition events (Varki et al., 2017). Despite their chemical similarity, glycan functions largely vary depending on the monosaccharide composition (i.e., relative stereochemistry), as well as on the regio- and stereochemistry of the glycosidic linkages (Gao and Chen, 2020; Gim et al., 2021). The chemical nature of the glycosidic linkages endows carbohydrates with a certain degree of flexibility that allows them to adopt a variety of three-dimensional shapes (Woods, 2018; Gimeno et al., 2020), related to their structural or biological functions (Kim et al., 2017; Gim et al., 2020; Srivastava et al., 2020; Dyukova et al., 2021). In the presence of a glycan receptor, conformational selection processes may easily take place, especially given the low energy barriers between the existing conformers (Zhang et al., 2019; Valverde et al., 2019a). This fact is particularly evident when the saccharide contains (1-6)-type glycosidic linkages that endow the corresponding carbohydrates with additional flexibility resulting in a larger range of possible conformations (Zerbetto et al., 2009; Hanashima et al., 2018).

The full understanding of the conformation, dynamics and interactions of carbohydrates remains a challenging task (Yu and Delbianco, 2020; Dedola et al., 2020), despite the enormous advances in several experimental techniques and theoretical methods. NMR has been extensively employed to assess the conformational, dynamic and recognition features of these flexible molecules. Recent developments using paramagnetic NMR approaches (Suzuki et al., 2017; Fernández de Toro et al., 2018) or NMR-active nuclei (^13^C, ^19^F) as labels permitted to circumvent the tremendous overlapping problem inherent to glycans (Fittolani et al., 2021; Moure et al., 2021), especially in the case of homo-oligosaccharides.

**FIGURE 1:**
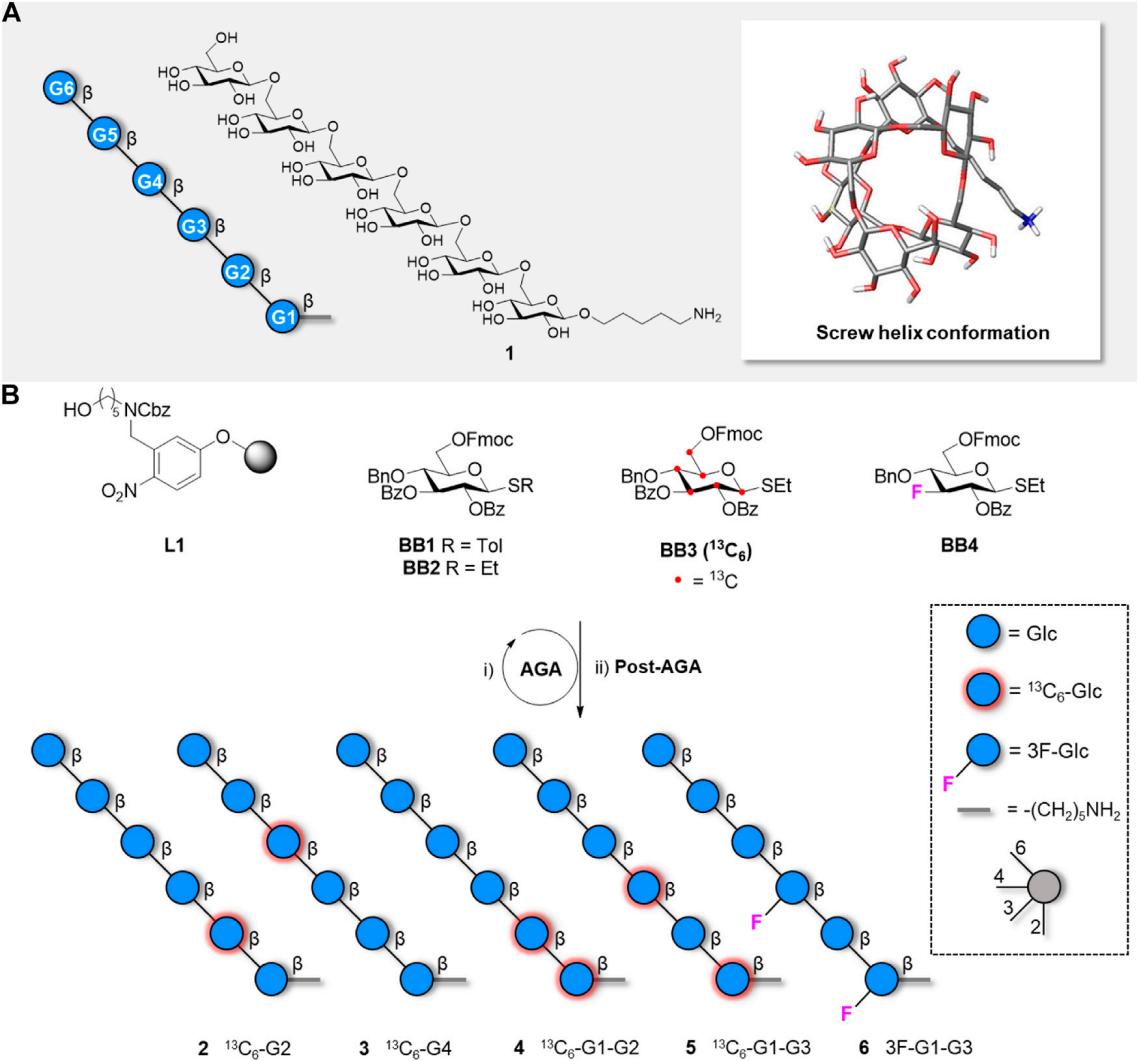
A) Representation following the Symbol Nomenclature For Glycans (SNFG), chemical structure, and global minimum conformation of hexasaccharide **1** as obtained by molecular dynamics simulations. B) Synthesis of five hexasaccharide analogues **2-6** bearing labelled functionalities in specific position of the glycan chain. The synthesis includes AGA and Post-AGA (detailed procedures are described in the Supplementary Material).

Fast access to a diverse set of complex glycans of biological interest, long polysaccharide structures, as well as natural and unnatural sugar-based materials was granted by innovations in synthetic chemistry, such as Automated Glycan Assembly (AGA) (Guberman and Seeberger, 2019). These well-defined glycans are valuable probes for structural analysis (Tyrikos-Ergas et al., 2020). Using AGA, we prepared a collection of oligo and polysaccharides that adopt different conformations depending on their monosaccharide sequence (Delbianco et al., 2018). Among them, the β(1-6)-Glc hexasaccharide **1** showed a particularly interesting behaviour; MD simulations predicted a variety of 3D conformations, including a helix-like shape (Figure 1A) and diverse extended or twisted geometries. A collection of single-residue ^13^C-labeled hexasaccharides permitted to break the chemical shift degeneracy of the hexamer and experimentally assess some geometrical features of its conformation at the single residue level (Delbianco et al., 2018). Still, a detailed description of the overall shape remained elusive, as “traditional” NMR parameters (i.e., NOEs, J-couplings) are limited to short-range distances (Valverde et al., 2019b; Krivdin, 2021) and cannot disclose the relative orientation of residues further apart in a linear saccharide chain (Battistel et al., 2014).

Residual dipolar couplings (RDCs) deliver information on the relative orientation of specific X-Y bonds between NMR-active nuclei (Tjandra and Bax, 1997). When these bonds are distributed along a molecule, the global analysis of RDC values may generate valuable information on the global molecular shape and/or assess the presence of a particular conformation (Bax and Grishaev, 2005). RDCs are generated in the so-called “alignment media” that can be viewed as an intermediate anisotropic state, between liquid and solid, and can be obtained by using specific liquid crystalline phases or stretched polymer gels (Reller et al., 2017; Lesot et al., 2020). Both approaches have been successfully applied to a variety of configurational and conformational problems in the carbohydrate field (Canales et al., 2012). With these methods, the molecules are partially aligned (less than 0.1% of the time) in the magnetic field and thus provide reduced “residual” dipolar couplings (in the order of Hz), which depend on the orientation of the corresponding X-Y bond with respect to the magnetic field (Troche-Pesqueira et al., 2017).

Herein, we employed two different alignment media to study the conformational behaviour of the β(1-6)-Glc hexasaccharide. ^13^C-labelled hexasaccharides (Delbianco et al., 2018) as well as fluorinated analogues prepared by AGA (Figure 1B) provided one bond ^13^C-^1^H RDC and ^13^C -^19^F RDCs respectively. The employed alignment media provided different types of interactions with the molecule, favouring different conformations. These results confirm the large oligosaccharide conformational flexibility and provide experimental clues for the existence of certain geometries predicted by MD.

## Results and Discussion

Our previous combined NMR/MD analysis of the β(1-6)-linked hexaglucoside **1** allowed us to deduce particular features around the individual glycosidic linkages. Still, while the MD simulations proposed the existence of a certain population of a helix-like structure (Figure 1A; Delbianco et al., 2018) no direct experimental evidence of the presence of the helical shape was obtained. The presence of five ω torsional degrees of freedom in the glycan backbone provides high flexibility to the saccharide chain that may adopt a variety of conformations. As mentioned above, NOE-based analysis of oligosaccharide conformation rarely provides a detailed picture of the global three-dimensional shape of glycans. This is especially true for linear, non-branched, oligosaccharides. In contrast, RDC analysis is a viable method to obtain global conformational information. Many interatomic vectors between NMR active nuclei may provide reliable experimental data that can be later analysed with the appropriate software protocols (Bax and Grishaev, 2005; Canales et al., 2012; Reller et al., 2017; Troche-Pesqueira et al., 2017; Lesot et al., 2020; Linclau et al., 2020). Since the different C-H vectors in a helix-like structure point towards different spatial orientations, we hypothesised that the measurement of specific C-H RDCs could provide experimental evidence of these shapes.

### Synthesis

Our previous study relied on synthetic analogues of hexasaccharide **1** bearing a single ^13^C-labelled Glc unit in different positions of the chain (e.g., Figure 1B, compounds **2–3**) (Delbianco et al., 2018). Additional compounds bearing two ^13^C-labelled Glc units (Figure 1B, compounds **4**, **5** and **S1**) were prepared to provide unambiguous and independent ^13^C-^1^H RDC values to allow for the analysis of the global conformational behaviour.

Within a β−Glc residue, all the intra-ring C-H vectors are parallel to each other and should provide the same RDC value. Therefore, in compounds **2-5** only the CH vectors at the methylene moiety could provide additional spatial information. An additional label, with a different orientation than the axial CH vectors, could generate additional spatial information. Given the excellent performance of ^19^F for NMR (Linclau et al., 2020), three ^19^F-containing hexaglucosides (compound **6**, **S2**, and **S3**) were targeted to obtain ^19^F-^13^C RDCs. The equatorial C-F bond was installed at position C-3, to minimize interference with folding. Two mono-fluorinated compounds (**S2** and **S3**) were used as chemical shift reference, whereas the di-fluorinated analogue **6** was employed in the RDC studies. All target compounds were obtained in good overall yields (13–21%) from **BB1**-**4** using standard AGA conditions (see Supplementary Material).

### NMR Analysis

Several weak alignment media are available for neutral molecules, offering different liquid crystal properties. To evaluate the presence of the helical conformer in solution, we selected the cromoglycate sodium salt (cromolyn) solution in D_2_O (Troche-Pesqueira et al., 2014) and the Otting’s medium (Rückert and Otting, 2000): C12E5/hexanol solution in D_2_O. The small ionic aromatic cromolyn (a mesogen) creates chromonic phases in which the rigid aromatic moieties self-aggregate in columns in a face-to-face fashion. The aromatic moieties arrange perpendicular to the axis of the formed cylinder. These cylinders in turn are aligned also perpendicular to the magnetic field (Yu and Saupe, 1982). The Otting medium (Rückert and Otting, 2000) generates lamellar L_α_ crystalline phases, which are formed by series of parallel lipidic bilayers, where the hydrophobic *n*-alkyl chains aggregate into planar bilayers with the hydrophilic poly (ethylene glycol) headgroups pointing towards the water phase. Within the NMR magnet, the bilayer surfaces orient parallel to the magnetic field direction. This medium has neutral charge, is insensitive to pH and salts, with little or no binding capacity, and can be used at temperatures close to 40°C.

All measurements were performed with each hexasaccharide in a different NMR tube. The splitting in the deuterium lock signal (^2^H Q splitting) confirmed a similar degree of alignment for each samples. The average obtained splitting in the cromolyn medium was 85 ±4 Hz , while in E5C12/hexanol was 27 ±1  ± 1 Hz, allowing for the comparison of the different samples within each medium. RDCs were experimentally determined from the analysis of HSQC NMR spectra at 800 MHz (Figure S1a, see the *General Materials and Methods* section for details) in two different weak alignment media (Tables 1, 2).

**TABLE 1:** RDCs (Hz) values measured in the **cromolyn medium** for the different ^13^C-labelled or ^19^F-containing hexasaccharides. The ^19^F-substituted and ^13^C-labelled residues are indicated. The specific RDC values in the Table correspond to the specified residue. The deuterium residual Quadrupolar splitting (Hz) for every measurement is also shown. The estimated error in the RDC values is ca. 1 Hz.

Compound	5 **[** ^**13**^ **C**_**6**_ **]-G1-G3**	2 **[** ^**13**^ **C**_**6**_ **]-G2**	5 **[** ^**13**^ **C**_**6**_ **]-G1-G3**	3 **[** ^**13**^ **C**_**6**_ **]-G4**	6 3F-G1-G3	6 3F-G1-G3
Residue	**G1**	**G2**	**G3**	**G4**	**3F-G1**	**3F-G3**
C1-H1	23	−19	−25	−4.2	18	−30
C2-H2	25.2	−23	−19.7	−1.2		
C3-H3		−21.9		−5	23	−24
C3-F	-	-	-	-	−13	13
C4-H4		−23.9		−5.3		
C5-H5		−19.5		−4.5		
C6-H6a	10.3	21.3	−0.1	−1.3		
C6-H6b	3.6	−10.2	2.9	4.3		
^2^H Q splitting	89	85	89	84	81	81

**TABLE 2:** RDCs (Hz) measured in the **C12E5/hexanol** medium for the different ^13^C-labelled hexasaccharides. The ^13^C-labelled residues are indicated. The specific RDC values in the Table correspond to the specified residue. The deuterium residual Quadrupolar splitting (Hz) for every measurement is also shown. The estimated error in the RDC values is ca. 1 Hz.

Compound	5 **[** ^**13**^ **C**_**6**_ **]-G1-G3**	4 **[** ^**13**^ **C**_**6**_ **]-G1-G2**	4 **[** ^**13**^ **C**_**6**_ **]-G1-G2**	5 **[** ^**13**^ **C**_**6**_ **]-G1-G3**	3 **[** ^**13**^ **C**_**6**_ **]-G4**
**Residue**	**G1**	**G1**	**G2**	**G3**	**G4**
C1-H1	11.8	10.5	9.87	8.8	4.9
C2-H2	12.7	10.6	9.0	6	4.8
C4-H4					5.3
C5-H5					8.5
C6-H6a					0
C6-H6b					0
^2^H Q splitting	26	26	26	27	28

**FIGURE 2:**
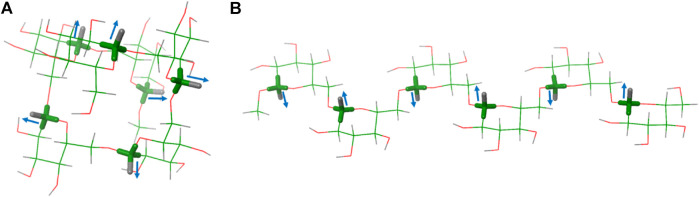
Two possible conformations of hexasaccharide **1**: **(A)** with all the ω bonds in *gg* conformation (*gg*_*6*_) and **(B)** with all the ω bonds in *gt* conformation (*gt*_*6*_).

The obtained results are strikingly different for both media. In the chromonic phase (Table 1), the measured RDCs display different signs, depending on the particular residue. Residue G1 always shows RDCs with positive values, while those for residues G2, G3 and G4 are negative. The RDC values within each ring are very similar, as expected from the parallel arrangement of the axial C-H vectors, as can be seen in compound **2** and **3** (residue G2 and G4, respectively). The differences in RDC absolute values for the different residues strongly support the different orientations of the Glc residues (G1, G2, G3, G4) along the hexasaccharide chain in the cromolyn phase. An identical behaviour was observed for compounds **5** and **6**, both labelled at residues G1 and G3 either with ^13^C or ^19^F. For these two compounds, the magnitude and sign measured for the anomeric C1-H1 RDC is similar. As expected, the RDC values for the equatorial C-F bond in residues G1 and G3 of compound **6** showed a different relative orientation than the axial C-H vectors within the same residue (RDC values with opposite sign). Moreover, the C-F RDC displays different signs for residues G1 and G3, as observed for the corresponding C-H vectors. These data are compatible with a helical shape of the molecule, with the C-H vectors at different Glc moieties pointing to different orientations (Figure 2A *gg*_*6*_ conformer).

The data in the C12E5/hexanol solution were drastically different (Table 2). Only positive values were found and the obtained anomeric C1-H1 RDCs for residues G1, G2, and G3 in different molecules were comparable in magnitude, but larger than those for G4. Taken together, these experimental data indicate different relative orientation of the Glc residues in the two media, suggesting a different conformational preference. In this case, the data are compatible with an extended conformation, with all the C-H vectors in parallel disposition (Figure 2B *gt*_*6*_ conformer).

### Comparison of Experiments and Models

**FIGURE 3:**
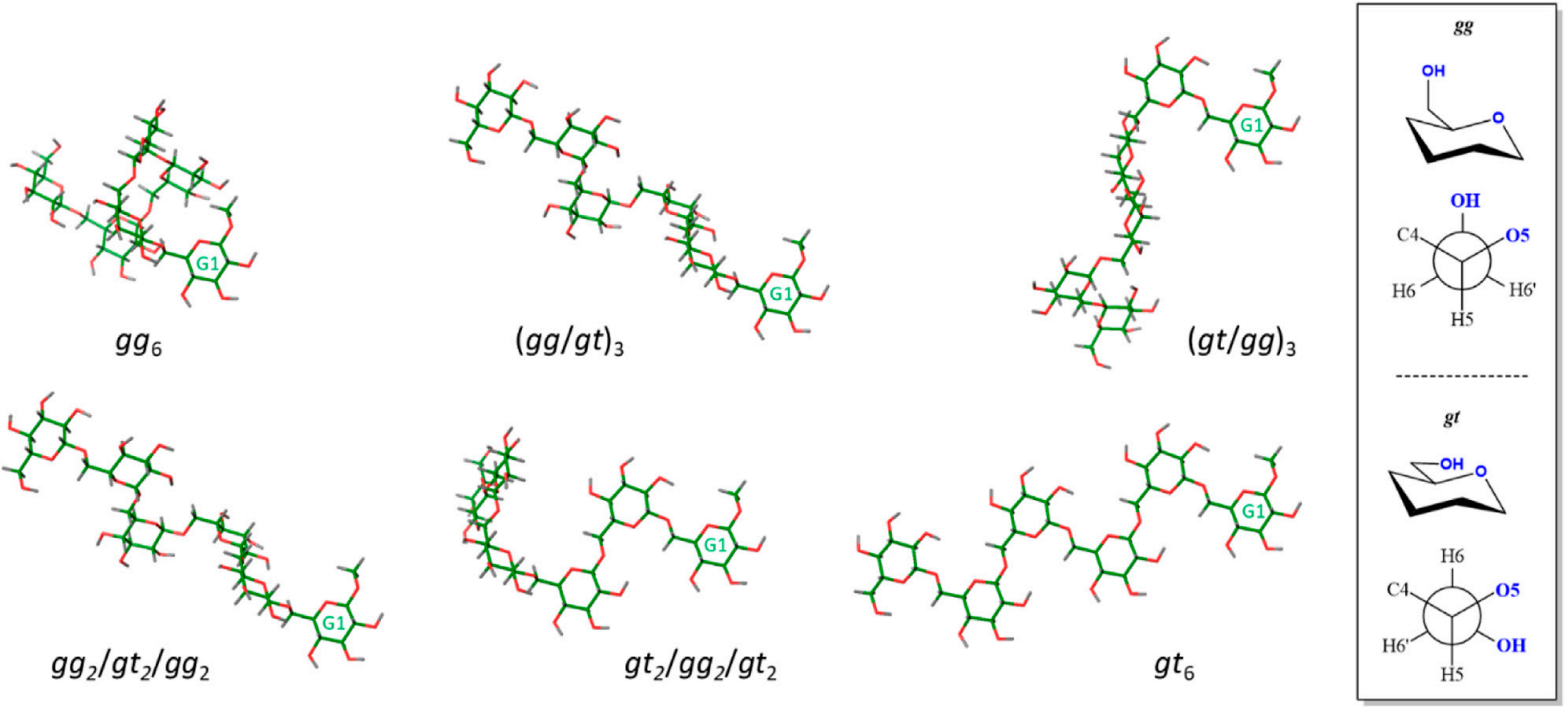
View of conformers selected for the fitting procedure. Residue G1 is always presented in the same orientation for comparison. Only the non-fluorinated structures are represented, since the fluorinated analogues showed the same behavior. A different perspective to that presented in Figure 1 for the helix-like structure (*gg*_6_, top left structure) is depicted. The description of the *gg* and *gt* orientations is shown in the right panel.

The experimental RDCs were fitted to those expected for different conformations of the hexasaccharide by using the MSPIN program, as described in the *General Materials and Methods* section (Navarro-Vazquez, 2012). Different conformations, helical, linear extended, and intermediate conformations were generated using the Macromodel suite of programs within Maestro 12.7 (Schrödinger, LLC, New York, NY, United States) and the AMBER* forcefield (see experimental section). For the helical conformation, the global minimum (Delbianco et al., 2018), Φ is kept within the exo-anomeric region, ψ adopts the *trans* conformation, and ω displays the *gauche-gauche* (*gg*) orientation. For the linear extended shape, all ω are in the *gauche-trans* (*gt*) rotamer (Figure 3). Other intermediate conformers were built with combinations of *gg* and *gt* rotamers.

The different data cross-fits are summarized in Table 3. The Cornillescu Quality factor (CQf), calculated from the fitting in MSPIN (see *General Materials and Methods* section), was used as quality measure. Additionally, the experimental RDC values were compared to those predicted by MSPIN for the canonical individual conformations (Tables S2abc).

**TABLE 3:** Cross-fitting of the experimental RDC data obtained in the chromonic medium or in the C12E5/hexanol solution for the model structures shown in Figure 3, with ω angles in the *gg* or *gt* conformations. The fitting of the experimental RDCs to other intermediate structures with mixed *gg* and *gt* orientations of the hydroxymethyl groups (*(gg/gt)*_*3*_ *, (gt/gg)*_*3,*_ *gg*_*2*_ */gt*_*2*_ */gg*_*2,*_ *gt*_*2*_ */gg*_*2*_ */gt*_*2*_) is also presented. The Cornillescu Quality factor (CQf) derived from the fitting in MSPIN is shown as a quality parameter of the adjustment. The best fitting for each compound is highlighted in green (bold). The following best fitting is light shaded.

Analysed compounds	CH_2_ included	Alignment medium	Conformers/*CQf*					
			*gg*_*6*_	*(gg/gt)*_*3*_	*(gt/gg)*_*3*_	*gg*_*2*_ */gt*_*2*_ */gg*_*2*_	*gt*_*2*_ */gg*_*2*_ */gt*_*2*_	*gt*_*6*_
2, 3, 5	Yes^a^	Cromolyn	**0.46**	0.50	0.80	0.51	0.63	0.53
2, 3, 6 (^19^F)	Yes^a^	Cromolyn	0.52	**0.26**	0.79	0.50	0.54	0.71
2, 3, 5	No^b^	Cromolyn	0.22	**0.11**	0.49	0.24	0.52	0.35
2, 3, 6 (^19^F)	No^b^	Cromolyn	0.42	**0.15**	0.72	0.48	0.39	0.64
3, 4, 5^b^	No^b^	C12E5/hexanol	0.93	0.31	0.32	0.27	**0.22**	0.25

a To fit the data of the four methylene protons (CH_2_) with RDC values, MSPIN program permutes their positions to make the calculation, so that 16 different spin assignments are generated. Only the best fit has been included.

b The data determined for the methylene protons were not included in the fitting. The estimated error in the RDC values is ca. 1 Hz.

The fitting analysis gathered in Table 3 used different data sets. Given the uncertainty provided by the methylene protons, results including or excluding the methylene RDCs were employed for the fitting processes. The results were further divided into two sets including (compounds **2**, **3**, and **6**) or excluding (compounds **2**, **3**, and **5**) the data measured for the fluorinated molecule. The experimental data were considered for fitting to the helix shape (all *gg*), to the extended geometry (all *gt*) and to the intermediate structures starting by either the *gg* or *gt* rotamers around the β(1–6) linkage (Figure 3). The comparison between the experimental RDCs and those predicted for the individual conformations according to MSPIN are reported in the Supplementary Material.

Data analysis indicates that the results obtained excluding the methylene protons provide better fittings than those including them. The helical conformation (all *gg*) provides the best fitting (although poor) when only the ^13^C-^1^H RDC values are included and the RDC data for the methylene moieties are considered. The relatively high CQf values (0.46 or above) suggest the existence of conformational equilibria around some of these linkages, so that no single rotamer can satisfactorily account for all the observed RDCs. When the ^13^C-^19^F RDC values are taken into consideration, the best fitting is obtained for a mixed *gg/gt* form. In this case, the fitting is considerably improved when the methylene data are neglected. For this set of data, the fitting for the helix shape is also reasonable. The fully extended conformer (all *gt*) always provides a poor fitting value (0.35 or above), even excluding the methylene protons. In contrast, for the C12E5/hexanol Otting’s medium, the fitting for the helix structure is rather poor in comparison to those obtained for the extended (best fit) or mixed geometries.

Even though it is difficult to provide a quantitative distribution of conformers, it seems that the population of extended conformers in cromolyn is rather low, while the helical conformer should partially contribute to the equilibrium. The existence of *gg* conformers is favoured in this medium, especially at the region of the reducing end of the hexasaccharide chain. Nevertheless, the *gt* rotamers should also contribute to the equilibrium, especially for internal linkages. In the C12E5/hexanol solution medium, the contribution of the helical conformer is probably negligible, while the fully extended conformer (i.e., all *gt*) should be significantly populated. Still, the existence of *gg* rotamers cannot be disputed. There is not a single structure (conformation), but many of them contributing to the final presentation. This fact also highlights that, in this case, as for other flexible molecules, it is not possible to derive a single 3D structure from the RDC data. This type of approach would generate a virtual structure, with no physical meaning.

The analysis of the RDCs demonstrates the high flexibility of the oligosaccharide. The results clearly show that the alignment medium is not inert and provides interactions with the molecule. The distinct chemical nature of the two employed media generates different interactions with the hexasaccharide, stabilizing different conformational distributions in the two environments. Since these possible intermolecular interactions, including CH-π interactions (Asensio et al., 2013), are quite weak, the observed modulation of the conformational populations reflects the low energy barriers among the contributing conformers.

## Conclusion

The conformational behavior of a flexible hexasaccharide was studied by NMR. A collection of selectively labelled hexasaccharides, bearing ^13^C-labelled or deoxyfluorinated Glc residues in specific positions, was prepared by AGA. This strategy permitted to overcome the extensive chemical shift degeneracy, allowing to measure specific NMR parameters (RDCs) related to the global 3D shapes of these molecules. Two distinct alignment media, displaying different physical-chemical properties when aligned in the presence of the large magnetic field provided by the NMR magnet, were tested. Drastically different RDC values were obtained for the hexasaccharide samples in the two different experimental conditions (chromonic and alcohol/ether phases), indicating different conformational behaviour. These data suggest that at least one of the alignment media strongly interact with the molecule, modulating its conformational behaviour. It is highly probable that the aromatic molecules in the chromonic medium provide aromatic-glycan interactions that drive the conformational equilibrium towards a significant population of the helix-like structure, the global minimum found in standard molecular mechanics calculations.

Given the relatively high CQf values for the fitting procedure and the chemical nature of the hexasaccharide with many torsional degrees of freedom, it is unlikely that a single conformation exists in solution, even in the presence of the alignment medium. The adaptability of flexible oligosaccharide structures to different environments is demonstrated. Moreover, it is evidenced that some alignment media are not innocuous and can establish interactions with the molecules under study, modulating their population distribution of conformers towards the geometries that provide the best intermolecular contacts. This modulation will depend on the chemical nature of the analyte, the energy barriers among the possible conformers, and the strength of the complementary interactions that may take place. Care should be taken when using external alignment media to explore molecular conformation and interactions through RDCs, especially in the absence of other experimental data.

## General Materials and Methods

### Synthesis

Automated glycan assembly (AGA) was performed on a home-built synthesizer developed at the Max Planck Institute of Colloids and Interfaces (Eller et al., 2013). All details concerning BB preparation, AGA modules, and post-AGA manipulations can be found in the Supplementary Material.

### Sample Preparation

#### C12E5/Hexanol Solution in D_2_O

Materials. D_2_O (99.9%, CIL), Pentaethylene glycol monododecyl ether C12E5 (98%, Sigma), and 1-hexanol (99.5%, Sigma) were used without further purification. Lamellar L_α_ phases were prepared by dissolving C12E5 in D_2_O and adding 1-hexanol alcohol in microliter (or fractions) steps to the desired final molar ratio under vigorous shaking. The solutions were biphasic at low alcohol concentrations and became transparent and opalescent when the L_α_ phase is formed. The composition of the final solution is reported in weight percent for the ratio C12E5 to solvent and the molar ratio of C12E5 to 1-hexanol is indicated by the factor r.

In our case, we prepared 1 ml of a stock solution with 940 μl of D_2_O and 60 μl of C12E5 (5.8% w/w). Then 1-hexanol was added in fractions of 0.2–1 μl following each addition by vigorous vortexing, up to reach the opalescent phase. At this point we calculated a factor r = 1.1. To prepare the NMR samples, 30 μl of a 4.6 mM solution of hexasaccharide were added in portions of 10 to a 150 μl of the C12E5/1-hexanol/D_2_O stock solution, following each addition by vigorous vortexing again. The hexasaccharide concentration was 0.77 mM. The final concentration of C12E5 was 4.9% w/w. These samples were prepared in 3.0 mm NMR capillaries suitable to the Bruker Match System.

The presence of the ordered phase was monitored by the observation of quadrupolar splitting Q of the ^2^H NMR signal of the solvent. After placing the sample in the magnet at 308 K, the quadrupolar splitting appeared within minutes. The final splitting was typically reached in 15–30 min. For these samples and temperature, the ^2^H Q splitting was 26–28 Hz.

#### Cromolyn Sodium Salt/Brine Solution in D_2_O

The Cromolyn/D_2_O/NaCl nematic phase stock solution was obtained by dissolving 50 mg of cromolyn (98%, Alfa Aesar) and 10 mg of NaCl in 0.66 mg of D_2_O at 50°C and then allowing the solution cool down.

To prepare the NMR samples, 30 μl of a 4.6 mM solution of hexasaccharide were added in portions of 10 to a 150 μl of the Cromolyn/D_2_O/NaCl stock solution, following each addition by vigorous vortexing again. The final concentration of Cromolyn was 7.4% w/w. These samples were measured in 3.0 mm NMR capillaries suitable to the Bruker Match System. For this system, the ^2^H Q splits found were 81–89 Hz.

### NMR Spectroscopy

All spectra were recorded on a Bruker AVANCE III 800 spectrometer operating at a frequency of 800.13 MHz for ^1^H, 201.19 MHz for ^13^C, and 122.83 MHz for ^2^H. One-bond ^1^H-^13^C coupling constants were extracted from HSQC spectra acquired without proton decoupling during the acquisition period. These HSQC spectra were acquired with spectral widths of 10 ppm for the direct proton dimension and 10 ppm for the indirect carbon dimension (F1 dimension was aliased) to achieve a time domain matrix of 8K × 512 complex points. These matrixes were apodized in both dimensions with a 90° shifted sinebell, and zero filled to 16 K × 2 K point.

Isotropic samples (D_2_O) and C12E5/Hexanol/D_2_O samples were shimmed with the TopShim routine, while Chromonic anisotropic samples were heated up to the point where the solution becomes isotropic (^2^H spectra were recorded to check that the deuterium signal was not a doublet), automatically shimmed using TopShim and then samples were allowed to cool down to the working temperatures.

### RDC Measurements

The coupling constants were extracted from 1D slices of the indirect frequency domain of the 2D spectrum. Each spectrum was duplicated and signals from α- and β-components of the multiplets were shifted relative to each other to reach an ideal overlap of the envelope of the second signal of the multiplet. The magnitude of the shift was the coupling constant value (Kummerloewe et al., 2010).

The residual dipolar couplings (D) were obtained by subtracting the ^1^J C-H splitting measured in a F2-coupled HSQC spectrum acquired in isotropic conditions (^1^J_CH_) to the same coupling obtained in anisotropic conditions (^1^T_CH_):

D1CH=T1CH‐J1CH

This then requires the acquisition of two sets of spectra: one in isotropic conditions, and the second in anisotropic media. For the C12E5/Hexanol medium samples, the isotropic values were obtained from a separate sample prepared in D_2_O. In the case of the cromolyn samples, both isotropic and anisotropic data were obtained in the same sample, just varying the temperature: 293 K for anisotropic conditions and 308 K for isotropic conditions. Given the spectral resolution, the error in the RDC values was estimated as ca. 1 Hz.

### MSPIN Analysis

For the MSPIN analysis two files are necessary (Navarro-Vazquez, 2012): a 3D Cartesian coordinates file and a text file with the experimental data which relates every atom pair with their experimental RDC values. Then, six conformers with different ω angle distribution, and their corresponding fluorinated analogues, were generated and energetically minimized using Schrödinger Macromodel software (Figure 2). The 3D Cartesian coordinates of each conformation were correlated with C12E5 and cromolyn experimental RDCs data, taking account all the possible combinations described above.

## Data Availability

The original contributions presented in the study are included in the article/Supplementary Material, further inquiries can be directed to the corresponding authors.
