# Supplementary material for: The Flexibility of Oligosaccharides Unveiled Through Residual Dipolar Coupling Analysis
Source: Front Mol Biosci. 2021 Nov 10;8:784318. doi: 10.3389/fmolb.2021.784318 (PMC8631391; doi:10.3389/fmolb.2021.784318)
Supplement: Supplementary file 1 [file DataSheet1.PDF]

Supporting Information

NMR analysis

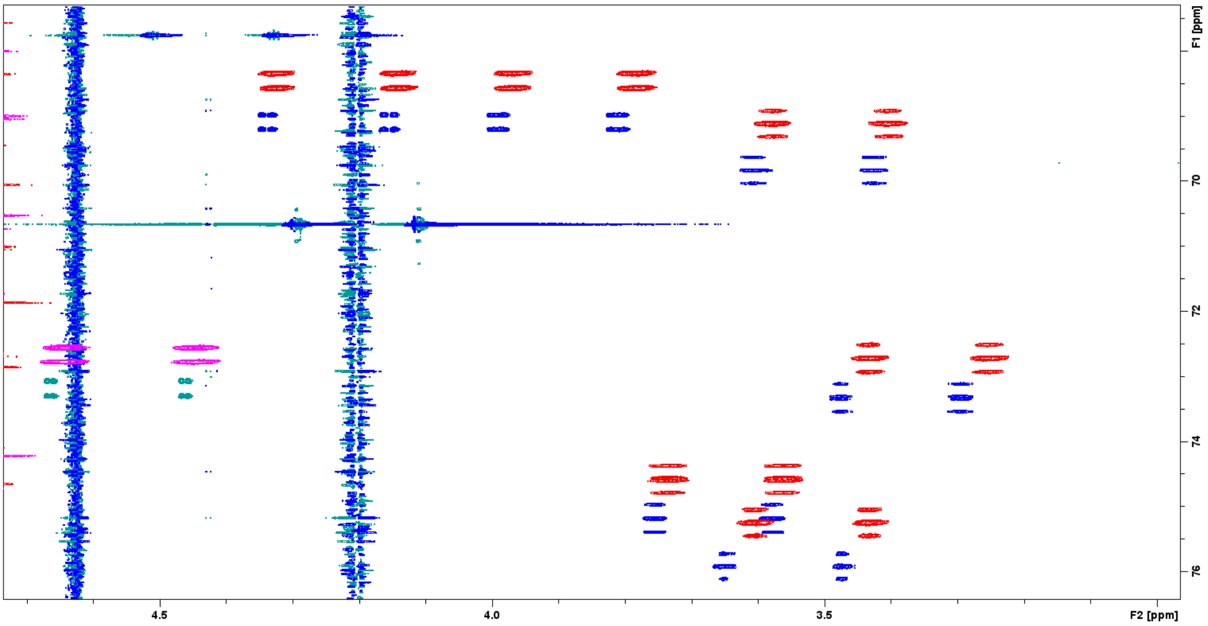

Figure S1a. Superimposition of the two HSQC spectra acquired for compound **2** in cromolyn medium: The blue-green spectrum corresponds to the isotropic phase at 318 K, whereas the red-pink spectrum corresponds to that in the chromonic phase at 293 K.

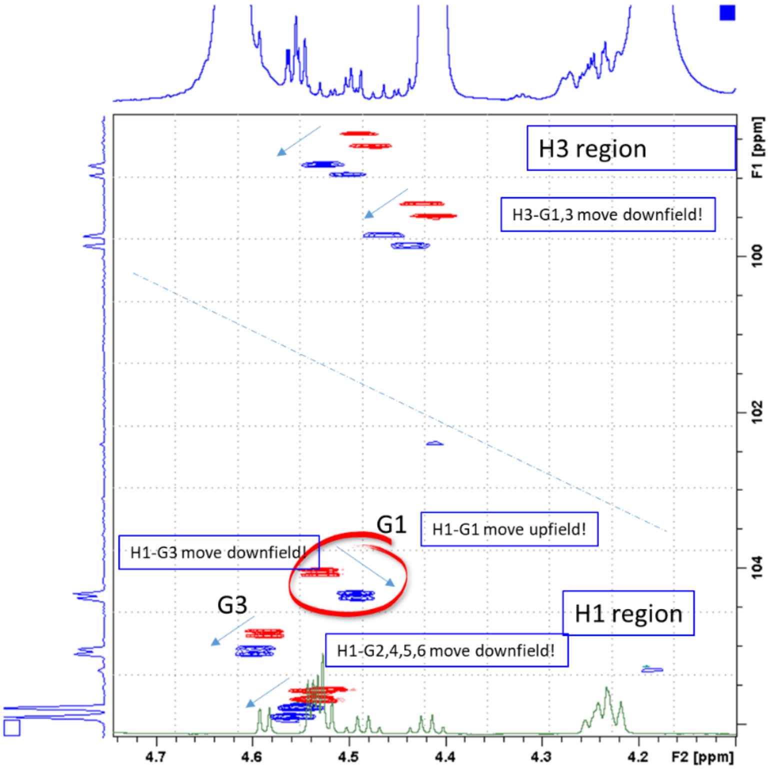

Figure S1b: Chemical shift perturbation due to temperature and/or medium (aromatic) on HSQC signals. Comparison between the two isotropic media preparations, **D_2O/298K** (red signals) and **CROMOLYN-D_2O/318K** (blue signals), for the fluorinated compound **6.**

Table S2a: Experimental (first row) and calculated RDCs (MSPIN) in cromolyn medium for different possible geometries of the hexasaccharide. Only the data for the conformations with the lowest CQF values are shown.

	CR-J Exp, [Hz]	gg6_all	gg6_tr	(gg/gt)3_all	(gg/gt)3_tr
G1-C1H1	23,0	25,7	29,5	14,9	24,3
G1-C2H2	25,2	28,0	29,1	12,5	23,7
G1-C6H6a	10,3	12,4		-5,2	
G1-C6H6b	3,6	-9,0		-15,8	
G2-C1H1	-19,0	-12,0	-22,2	-18,8	-21,9
G2-C2H2	-23,0	-15,6	-20,5	-17,9	-21,8
G2-C3H3	-21,9	-14,3	-22,1	-17,6	-22,0
G2-C4H4	-23,9	-10,2	-19,2	-19,5	-21,8
G2-C5H5	-19,5	-16,2	-15,6	-19,0	-21,5
G2-C6H6a	21,3	23,4		10,1	
G2-C6H6b	-10,2	-13,6		-18,3	
G3-C1H1	-25,0	-16,4	-18,2	-21,8	-21,3
G3-C2H2	-19,7	-12,8	-15,7	-21,1	-21,7
G3-C6H6a	0,0	-14,7		-3,3	
G3-C6H6b	2,9	-2,0		13,8	
G4-C1H1	-4,2	0,7	-4,6	-0,2	-3,7
G4-C2H2	-1,2	-1,9	-1,6	-2,2	-4,1
G4-C3H3	-5,0	-1,5	-4,1	-1,2	-3,5
G4-C4H4	-5,3	2,4	-2,5	-0,7	-4,2
G4-C5H5	-4,5	-1,4	2,6	-3,1	-4,8
G4-C6H6a	-1,3	1,0		-3,6	
G4-C6H6b	4,3	12,6		5,6	
Cornilescu Quality factor:		0,46	0,23	0,50	0,11

Table S2b: Experimental (first row) and calculated RDCs (MSPIN) in cromolyn medium, including fluorinated compounds. Only the data for the conformations with the lowest CQF values are shown.

	CRF-J Exp, [Hz]	(gg/gt)3_all	(gg/gt)3_tr	gg2/gt2/gg2_all	gt2/gg2/gt2_tr
G1F-C1H1	18,0	17,36	20,39	25,99	4,61
G1F-C3H3	23,0	16,55	19,8	19,02	20,2
G1F-C3F3	-13,0	-8,4	-9,14	1,1	-9,01
G2-C1H1	-19,0	-21,74	-22,01	-20,85	-17,09
G2-C2H2	-23,0	-20,26	-20,82	-15,52	-13,69
G2-C3H3	-21,9	-19,74	-20,03	-17,06	-9,98
G2-C4H4	-23,9	-22,86	-23,25	-22,77	-22,87
G2-C5H5	-19,5	-22,35	-23,26	-14,85	-25,14
G2-C6H6a	21,3	11,86		14,21	
G2-C6H6b	-10,2	-21,38		-17,73	
G3F-C1H1	-30,0	-27,02	-27,35	-12,12	-35,29
G3F-C3H3	-24,0	-24,26	-24,47	-19,08	-22,18
G3F-C3F3	13,0	12,68	14,6	-7,51	7
G4-C1H1	-4,2	-2,46	-2,01	-6,36	1,15
G4-C2H2	-1,2	-6,09	-6,01	-6,51	-9,02
G4-C3H3	-5,0	-4,45	-4,34	-9,4	-5,07
G4-C4H4	-5,3	-3,19	-2,69	-3,74	4,4
G4-C5H5	-4,5	-7,17	-6,97	-0,44	-12,05
G4-C6H6a	-1,3	-8,34		-3,76	
G4-C6H6b	4,3	6,26		14,18	
Cornilescu Quality factor:		0,27	0,15	0,50	0,39

Table S2c: Experimental (first row) and calculated RDCs (MSPIN) in E5C12/hex medium. Only the data for the conformations with the lowest CQF values are shown.

	E5C12- J Exp [Hz]	gt6	gt2/gg2/gt2
G1-C1H1	11,8	11,5	12,35
G1-C2H2	12,7	13,1	10,81
G2-C1H1	10,5	10,1	9,34
G2-C2H2	10,6	10,3	11,95
G3-C1H1	9,9	7,9	9,68
G3-C2H2	9,0	9,4	7,93
G4-C1H1	4,9	6,2	7,24
G4-C2H2	4,8	5,6	6,7
G4-C4H4	5,3	5,5	5,35
G4-C5H5	8,5	3,8	4,39
Cornilescu Quality factor:		0,25	0,22

Automated glycan assembly

General materials and methods

The automated syntheses were performed on a home-built synthesizer developed at the Max Planck Institute of Colloids and Interfaces. All solvents used were HPLC-grade. The solvents used for the building blocks, activator, TMSOTf and capping solutions were taken from an anhydrous solvent system (J.C. Meyer). The building blocks were co-evaporated three times with toluene and dried for 1 h on high vacuum before use. Oven-heated, argon-flushed flasks were used to prepare all moisture-sensitive solutions. Activator, capping, deprotection, acidic wash and building block solutions were freshly prepared and kept under argon during the automation run. All yields of products obtained by AGA were calculated on the basis of resin loading. Resin loading was determined following previously established procedures.[1]

Building blocks

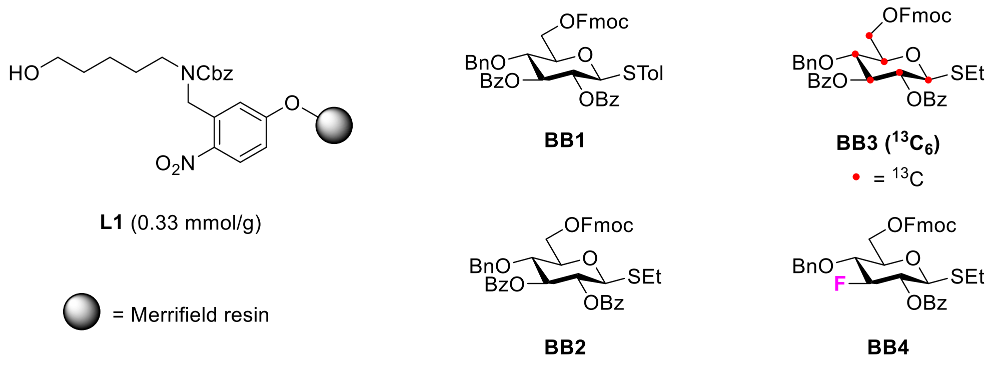

Figure S3. Solid support (L1) and building blocks (BBs) used for AGA.

Building blocks **BB1** was purchased from GlycoUniverse (Germany). Linkers **L1** and **BB2-4** were prepared according to previously established protocols.[2,3]

Preparation of stock solutions

- **Building block solution**: 0.08 mmol (6.5 equiv.) of building block was dissolved in DCM (1 mL).
- **NIS/TfOH activator solution**: 1.35 g (6.0 mmol) of recrystallized NIS was dissolved in 40 mL of a 2:1 v/v mixture of anhydrous DCM and anhydrous dioxane. Then triflic acid (55 µL, 0.6 mmol) was added. The solution is kept at 0°C for the duration of the automation run.
- **Fmoc deprotection solution**: A solution of 20% piperidine in DMF (v/v) was prepared.

- **TMSOTf solution**: TMSOTf (0.45 mL, 2.49 mmol) was added to DCM (40 mL).
- **Capping solution**: A solution of 10% acetic anhydride and 2% methanesulfonic acid in DCM (v/v) was prepared.

Modules for automated synthesis

Module A: Resin Preparation for Synthesis (20 min)

All automated syntheses were performed on 0.0125 mmol scale. Resin (**L1**, loading 0.33 mmol/g) was placed in the reaction vessel and swollen in DCM for 20 min at room temperature prior to synthesis. During this time, all reagent lines needed for the synthesis were washed and primed. After the swelling, the resin was washed with DMF, THF, and DCM (three times each with 2 mL for 25 s).

Module B: Acidic Wash with TMSOTf Solution (20 min)

The resin was swollen in 2 mL DCM and the temperature of the reaction vessel was adjusted to -20 °C. Upon reaching the low temperature, TMSOTf solution (1 mL) was added dropwise to the reaction vessel. After bubbling for 3 min, the acidic solution was drained and the resin was washed with 2 mL DCM for 25 s.

Action	Cycles	Solution	Amount	T	Incubation time
Cooling	-	-	-	-20 °C	(15 min)*
Deliver	1	DCM	2 mL	-20 °C	-
Deliver	1	TMSOTf solution	1 mL	-20 °C	3 min
Wash	1	DCM	2 mL	-20 °C	25 sec

*Time required to reach the desired temperature.

Module C: Thioglycoside Glycosylation (35 min)

The building block solution (0.08 mmol of BB in 1 mL of DCM per glycosylation) was delivered to the reaction vessel. After the set temperature (-20 °C) was reached, the reaction was started by dropwise addition of the NIS/TfOH activator solution (1.0 mL, excess). After completion of the reaction (5 min at -20 °C and 20 min at 0 °C), the solution was drained and the resin was washed with DCM, DCM:dioxane (1:2, 3 mL for 20 s) and DCM (two times, each with 2 mL for 25 s). The temperature of the reaction vessel was increased to 25 °C for the next module.

Action	Cycles	Solution	Amount	T	Incubation time
Cooling	-	-	-	-20 °C	-
Deliver	1	BB solution	1 mL	-20 °C	-
Deliver	1	NIS/TfOH activator solution	1 mL	-20 °C	-
Reaction time	1	-	-	-20 °C<br>to 0 °C	5 min<br>20 min
Wash	1	DCM	2 mL	0 °C	5 s
Wash	1	DCM : Dioxane (1:2)	2 mL	0 °C	20 s
Heating	-	-	-	25 °C	-
Wash	2	DCM	2 mL	> 0 °C	25 s

Module D: Capping (30 min)

The resin was washed with DMF (two times with 2 mL for 25 s) and the temperature of the reaction vessel was adjusted to 25 °C. 2 mL of Pyridine solution (10% in DMF) was delivered into the reaction vessel. After 1 min, the reaction solution was drained and the resin washed with DCM (three times with 3 mL for 25 s). 4 mL of capping solution was delivered into the reaction vessel. After 20 min, the reaction solution was drained and the resin washed with DCM (three times with 3 mL for 25 s).

Action	Cycles	Solution	Amount	T	Incubation time
Heating	-	-	-	25 °C	(5 min)*
Wash	2	DMF	2 mL	25 °C	25 s
Deliver	1	10% Pyridine in DMF	2 mL	25 °C	1 min
Wash	3	DCM	2 mL	25 °C	25 s
Deliver	1	Capping Solution	4 mL	25 °C	20 min
Wash	3	DCM	2 mL	25 °C	25 s

*Time required to reach the desired temperature.

Module E: Fmoc Deprotection (9 min)

The resin was washed with DMF (three times with 2 mL for 25 s) and the temperature of the reaction vessel was adjusted to 25 °C. 2 mL of Fmoc deprotection solution was delivered to the reaction vessel and kept under Ar bubbling. After 5 min, the reaction solution was drained and the resin washed with DMF (three times with 3 mL for 25 s) and DCM (five times each with 2 mL for 25 s). The temperature of the reaction vessel was decreased to -20 °C for the next module.

Action	Cycles	Solution	Amount	T	Incubation time
Wash	3	DMF	2 mL	25 °C	25 s
Deliver	1	Fmoc depr. solution	2 mL	25 °C	5 min
Wash	1	DMF	2 mL	25 °C	10 s
Cooling	-	-	-	-20 °C	-
Wash	3	DMF	2 mL	< 25 °C	25 s
Wash	5	DCM	2 mL	< 25 °C	25 s

Post-synthesizer manipulations

Module F: Cleavage from Solid Support

The oligosaccharides were cleaved from the solid support using a continuous-flow photoreactor as described previously.[4]

Module G: Solution-phase Methanolysis

The protected oligosaccharide was dissolved in MeOH : DCM (1.5 mL, 1:1). NaOMe in MeOH (0.5 M, 3 equiv. per benzoyl ester) was added and the solution was stirred at room temperature, neutralized with Amberlite IR-120 (H+ form), filtered and concentrated *in vacuo*. The crude compound was used for hydrogenolysis without further purification.

Module H: Hydrogenolysis at Ambient Pressure

The crude compound obtained from *Module G* was dissolved in 2 mL of EA:*t*BuOH:H_2O (2:1:1). 100% by weight Pd-C (10%) was added and the reaction was stirred in a flask equipped with a H_2 balloon. The reaction progress was monitored to avoid undesired side products formation. Upon completion, the reaction was filtered, and the Pd/C washed with EA, *t*BuOH, and H_2O. The filtrates were concentrated *in vacuo*.

Module I: Purification

The purification of the crudes was conducted using preparative normal phase HPLC (Agilent 1200 Series, Method A) or reverse phase HPLC (Agilent 1200 Series, Method C, and E). The pure compound was analyzed using analytical HPLC (Agilent 1200 Series, Method B, D, and F).

- **Method A:** (YMC-Diol-300 column, 150 x 20 mm), flow rate of 15 mL/min with Hexane and EtOAc as eluents [isocratic 20% EtOAc (5 min), linear gradient to 55% EtOAc (35 min), linear gradient to 100% EtOAc (5 min)].

- **Method B:** (YMC-Diol-300 column, 150 x 4.6 mm), flow rate of 1.0 mL/min with Hexane and EtOAc as eluents [isocratic 20% EtOAc (5 min), linear gradient to 55% EtOAc (35 min), linear gradient to 100% EtOAc (5 min)].

- **Method C:** (Hypercarb column, 150 x 10 mm) flow rate of 1.3 mL/min with H_2O (0.1% formic acid) and ACN as eluents [isocratic (5 min), linear gradient to 30% ACN (30 min), linear gradient to 100% ACN (5 min)].

- **Method D:** (Hypercarb column, 150 x 4.6 mm) flow rate of 0.7 mL/min with H_2O (0.1% formic acid) and ACN as eluents [isocratic (5 min), linear gradient to 30% ACN (30 min), linear gradient to 100% ACN (5 min)].

- **Method E:** (Synergi Hydro RP18 column, 250 x 10 mm) flow rate of 4.0 mL/min with H_2O (0.1% formic acid) and ACN as eluents [isocratic (5 min), linear gradient to 30% ACN (30 min), linear gradient to 100% ACN (5 min)].

- **Method F:** (Synergi Hydro RP18 column, 250 x 4.6 mm) flow rate of 1.0 mL/min with H_2O (0.1% formic acid) and ACN as eluents [isocratic (5 min), linear gradient to 30% ACN (30 min), linear gradient to 100% ACN (5 min)].

Following final purification, all deprotected products were lyophilized on a Christ Alpha 2-4 LD plus freeze dryer prior to characterization.

Oligosaccharides synthesis

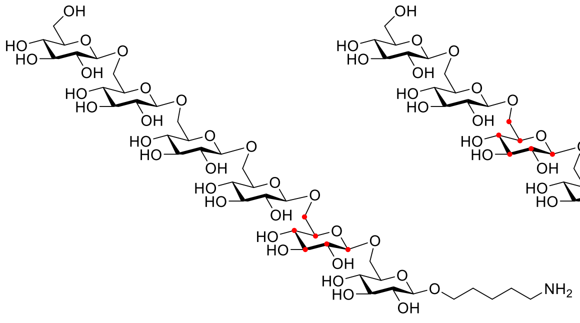

2 $^{13}C_6$-G2

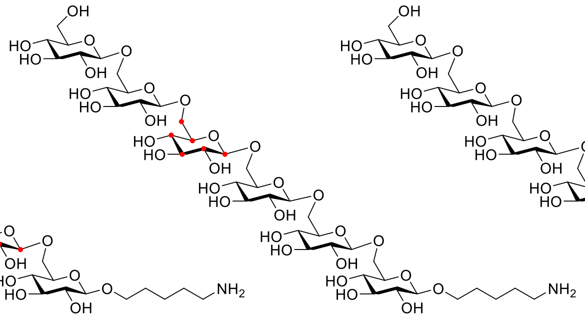

3 $^{13}C_6$-G4

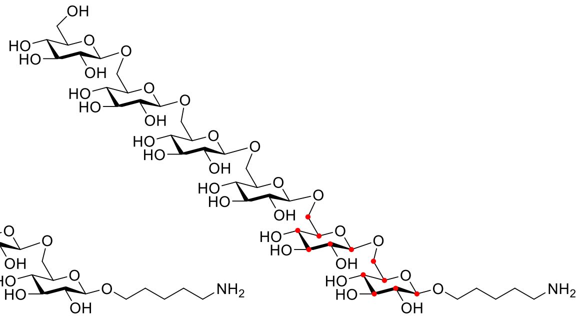

4 $^{13}C_6$-G1-G2 (15%)

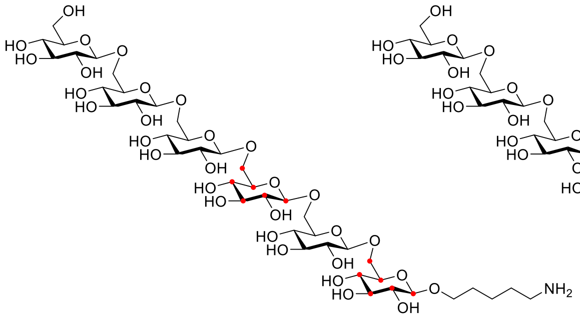

5 $^{13}C_6$-G1-G3 (18%)

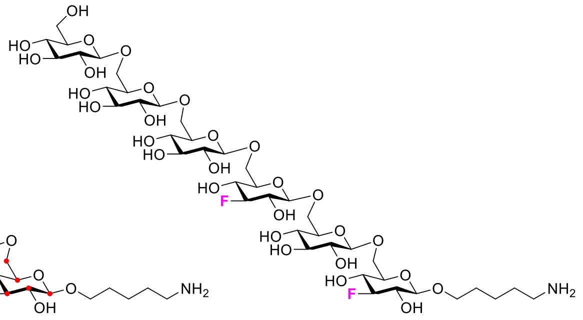

6 3F-G1-G3 (17%)

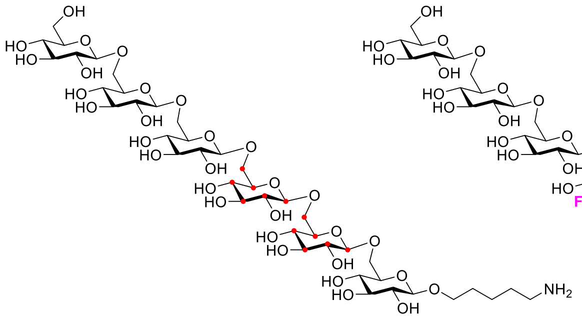

S1 $^{13}C_6$-G2-G3 (13%)

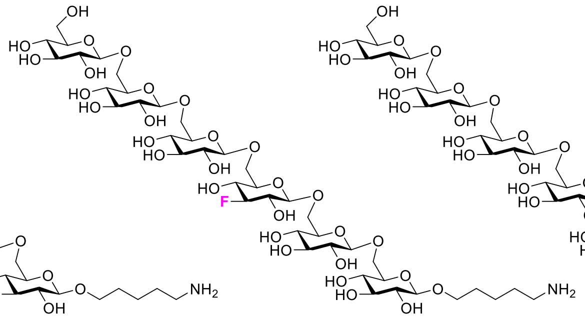

S2 3F-G3 (17%)

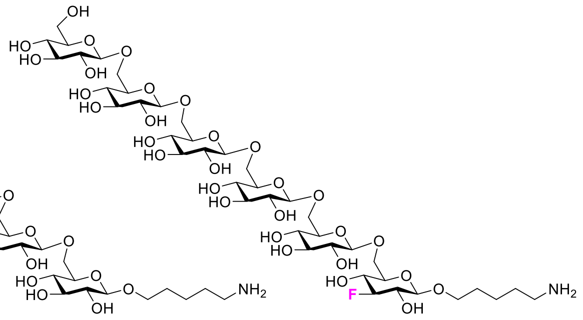

S3 3F-G1 (21%)

Figure S4. Labelled oligosaccharide synthesized. Compound **2** and **3** were synthesized as previously reported.[2] The overall yield is reported between parentheses (over 15 steps including AGA, photocleavage and global deprotection).

Synthesis of 4 $^{13}C_6$-G1-G2

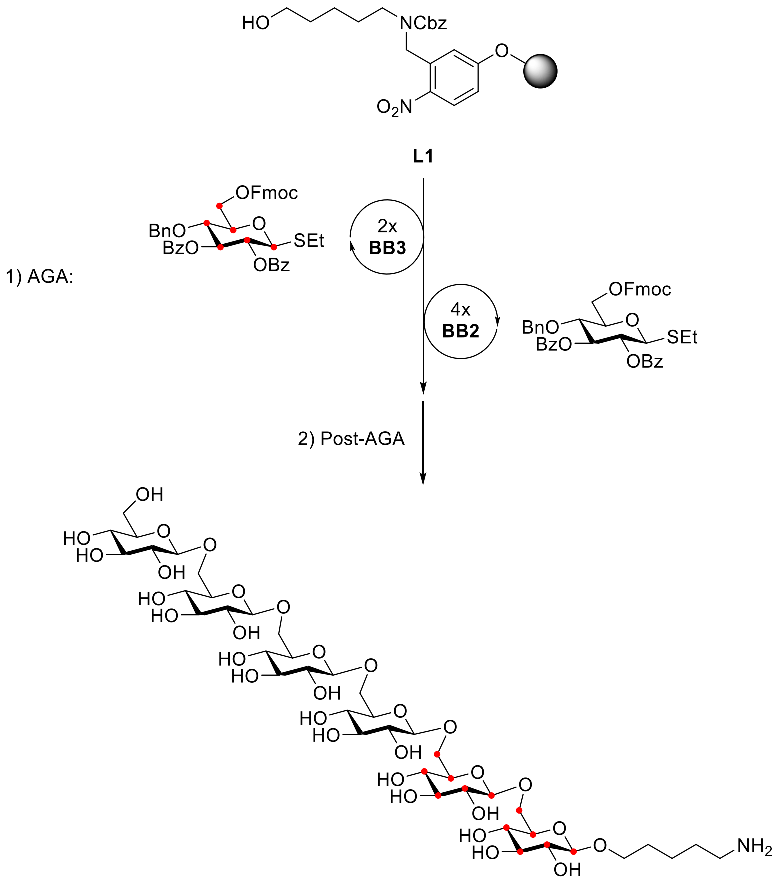

4 $^{13}C_6$-G_1-G_2

Step	BB	Modules	Notes
AGA		**A**	**L1** swelling
	2x **BB3**	**B**, **C**, **D**, **E**	**C**: (**BB3**, -20°C for 5 min, 0°C for 20 min)
	4x **BB2**	**B**, **C**, **D**, **E**	**C**: (**BB2**, -20°C for 5 min, 0°C for 20 min)
Post-AGA		**F, I*, G, H, I****	**I***: (Method A, t_R = 31.6 min)<br>**G**: (16 h)<br>**H**: (20 h)<br>**I****: (Method C: 31.7 min)

Automated synthesis, global deprotection, and purification afforded **4** as a white solid (2.3 mg, 15% overall yield).

Analytical data for **4**: ^{1}H NMR (700 MHz, Deuterium Oxide) δ 4.59 – 4.32 (m, 1H), 4.47 – 4.42 (m, 4H), 4.53 – 4.26 (m, 1H), 4.15 (dddd, *J* = 10.1, 6.0, 3.9, 2.1 Hz, 2H), 4.28 – 4.01 (m, 2H), 3.90 – 3.82 (m, 2H), 3.79 (dq, *J* = 10.9, 5.5 Hz, 3H), 3.71 – 3.59 (m, 2H), 3.59 – 3.53 (m, 3H), 3.53 – 3.46 (m, 1H), 3.46 – 3.37 (m, 9H), 3.32 – 3.08 (m, 37H), 2.94 (t, *J* = 8.0 Hz, 2H), 1.67 – 1.55 (m, 4H), 1.39 (p, *J* = 7.8 Hz, 2H). ^{13}C NMR (176 MHz, Deuterium Oxide) δ 102.89 (d, *J* = 46.4 Hz),

102.22 (d, *J* = 46.8 Hz), 75.66 (m), 74.88 (t, *J* = 42.4 Hz), 73.03 (dd, *J* = 47.3, 38.6, Hz), 69.78 – 69.04 (m), 68.76 (d, *J* = 44.0 Hz). (ESI-HRMS) m/z 1088.461 $[M+H]^+$ ($C_{29}{}^{13}C_{12}H^{74}NO_{31}$ requires 1088.464).

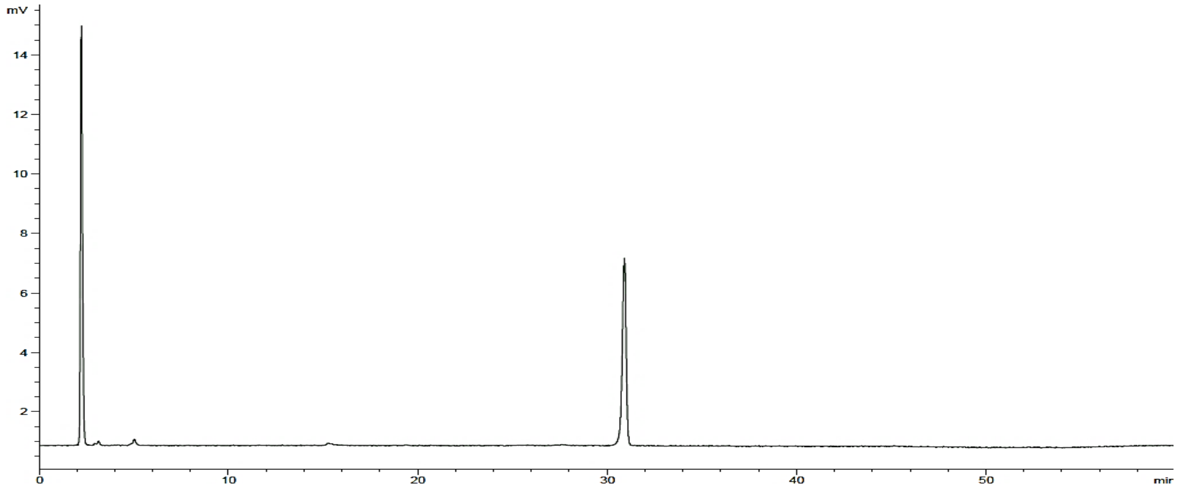

NP-HPLC of crude fully protected compound after photocleavage (Module F) (ELSD trace, Method B, t_R = 30.8 min).

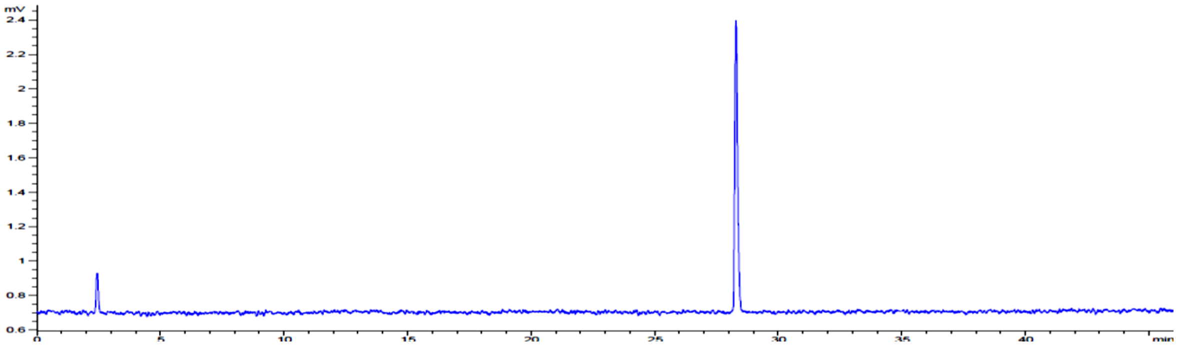

RP-HPLC of **4** (ELSD trace, Method D, t_R = 28.7 min).

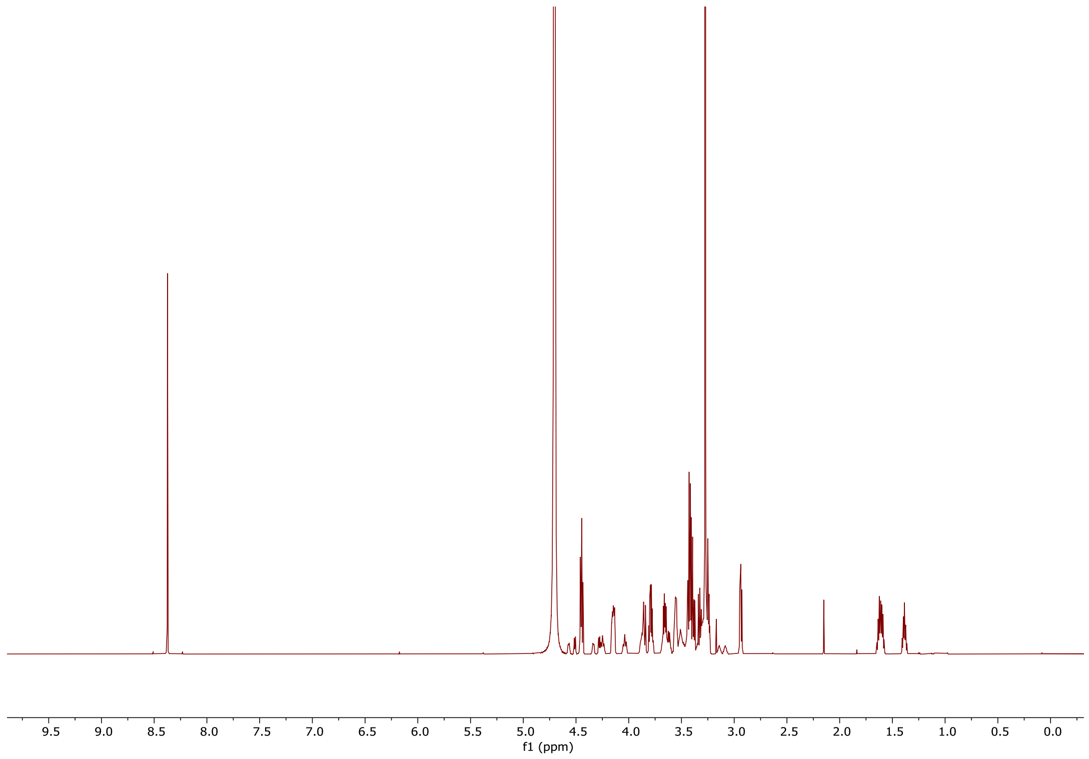

^{1}H-NMR spectrum of **4** (700 MHz, Deuterium Oxide).

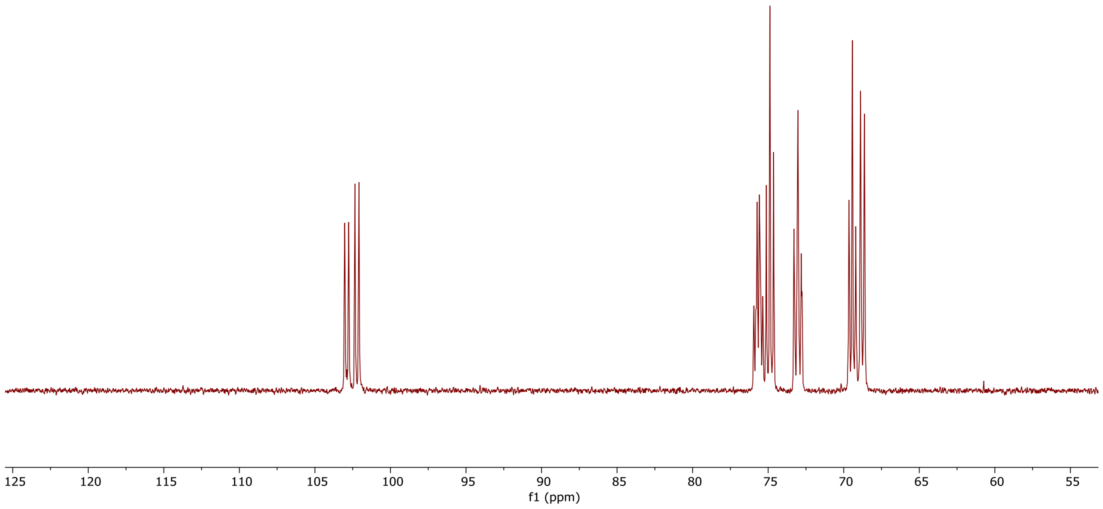

^{13}C-NMR spectrum of **4** (176 MHz, Deuterium Oxide).

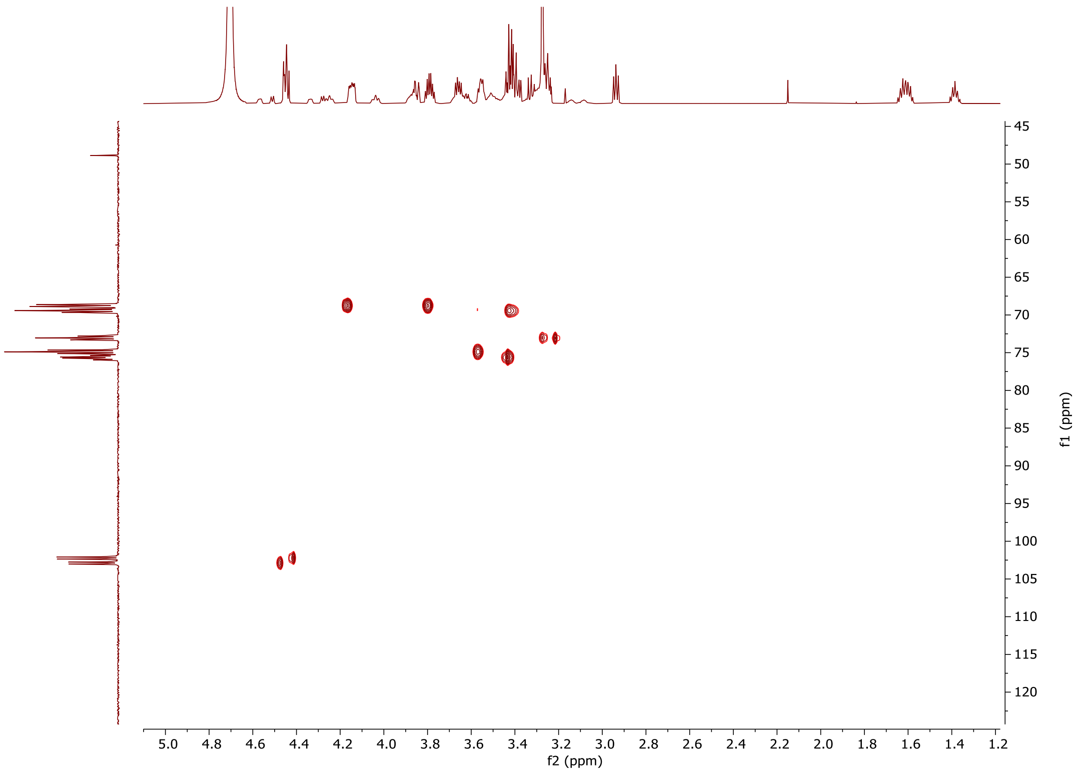

HSQC-NMR spectrum of **4** (Deuterium Oxide).

Synthesis of 5 $^{13}C_6$-G1-G3

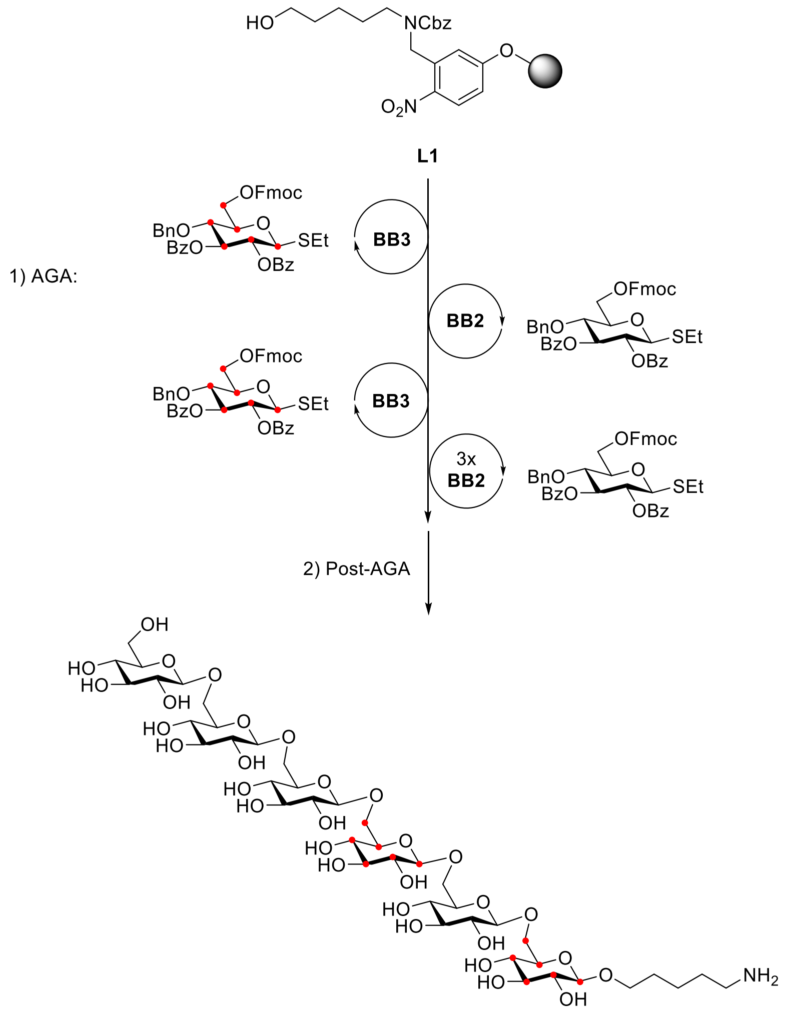

5 $^{13}C_6$-G1-G3

Step	BB	Modules	Notes
AGA		**A**	**L1** swelling
	**BB3**	**B**, **C**, **D**, **E**	**C**: (**BB3**, -20°C for 5 min, 0°C for 20 min)
	**BB2**	**B**, **C**, **D**, **E**	**C**: (**BB2**, -20°C for 5 min, 0°C for 20 min)
	**BB3**	**B**, **C**, **D**, **E**	**C**: (**BB3**, -20°C for 5 min, 0°C for 20 min)
	3x **BB2**	**B**, **C**, **D**, **E**	**C**: (**BB2**, -20°C for 5 min, 0°C for 20 min)
Post-AGA		**F, I*, G, H, I****	**I***: (Method A, t_R = 31.6 min)<br>**G**: (16 h)<br>**H**: (20 h)<br>**I****: (Method C: 31.7 min)

Automated synthesis, global deprotection, and purification afforded **5** as a white solid (2.4 mg, 18% overall yield).

Analytical data for **5**: ^{1}H NMR (700 MHz, Deuterium Oxide) 4.56 (dd, *J* = 161.9, 8.5 Hz, 1H), 4.45 (q, *J* = 6.3 Hz, 4H), 4.40 (dd, *J* = 160.9, 8.3 Hz, 1H), 4.28 – 4.01 (m, 2H), 4.17 – 4.12 (m, 3H), 3.92 – 3.82 (m, 2H), 3.79 (dq, *J* = 13.9, 6.9 Hz, 3H), 3.71 – 3.59 (m, 2H), 3.59 – 3.36 (m, 11H), 3.32 – 3.08 (m, 16H), 2.94 (t, *J* = 7.6 Hz, 2H), 1.61 (tt, *J* = 14.8, 7.2 Hz, 4H), 1.39 (q, *J* = 7.9 Hz, 2H). ^{13}C NMR (176 MHz, Deuterium Oxide) δ 102.97 (d, *J* = 46.6 Hz), 102.22 (d, *J* = 46.9 Hz), 76.10 – 75.23 (m), 74.89 (t, *J* = 42.4 Hz), 73.05 (dd, *J* = 46.9, 38.8 Hz), 69.44 (t, *J* = 40.0 Hz), 68.78 (d, *J* = 44.2 Hz). (ESI-HRMS) m/z 1088.465 $[M+H]^+$ ($C_{29}{}^{13}C_{12}H^{74}NO_{31}$ requires 1088.464).

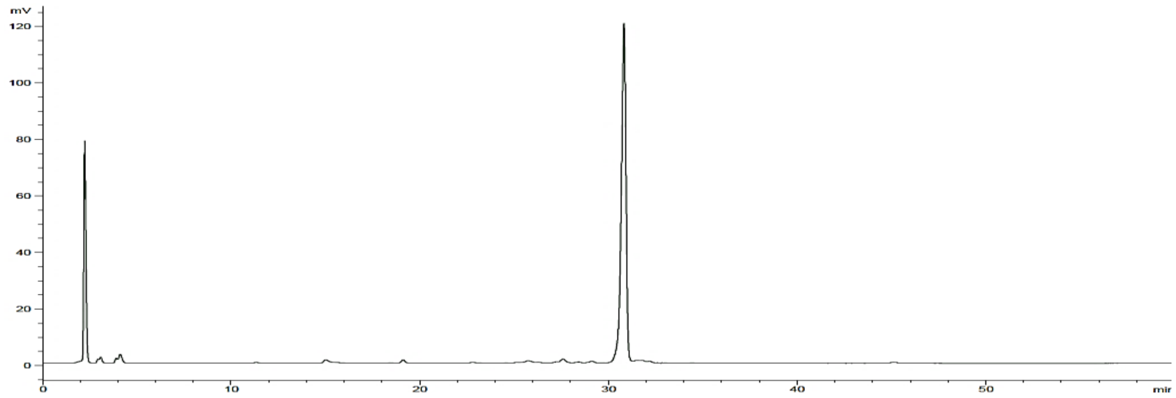

NP-HPLC of crude fully protected compound after photocleavage (Module F) (ELSD trace, Method B, t_R = 30.8 min).

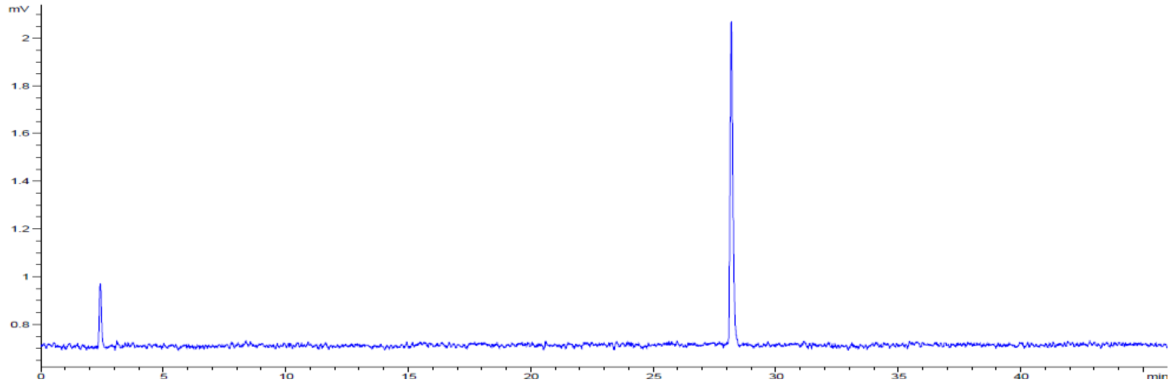

RP-HPLC of **5** (ELSD trace, Method D, t_R = 28.7 min).

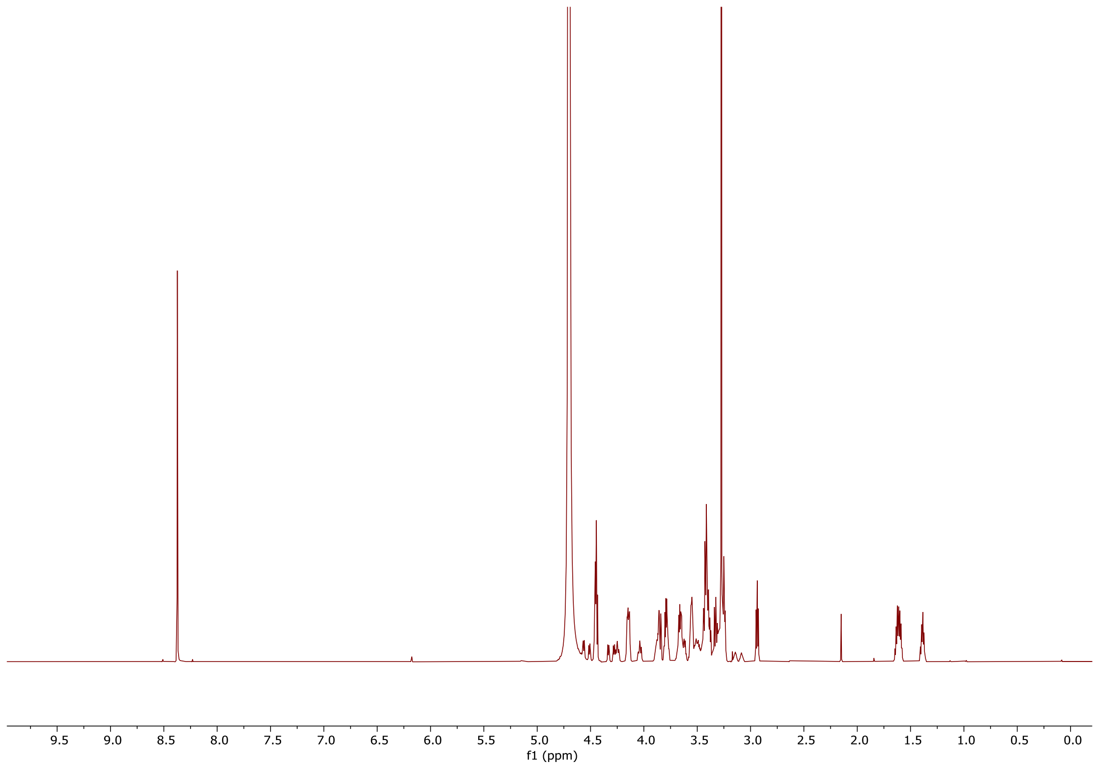

^{1}H-NMR spectrum of **5** (700 MHz, Deuterium Oxide).

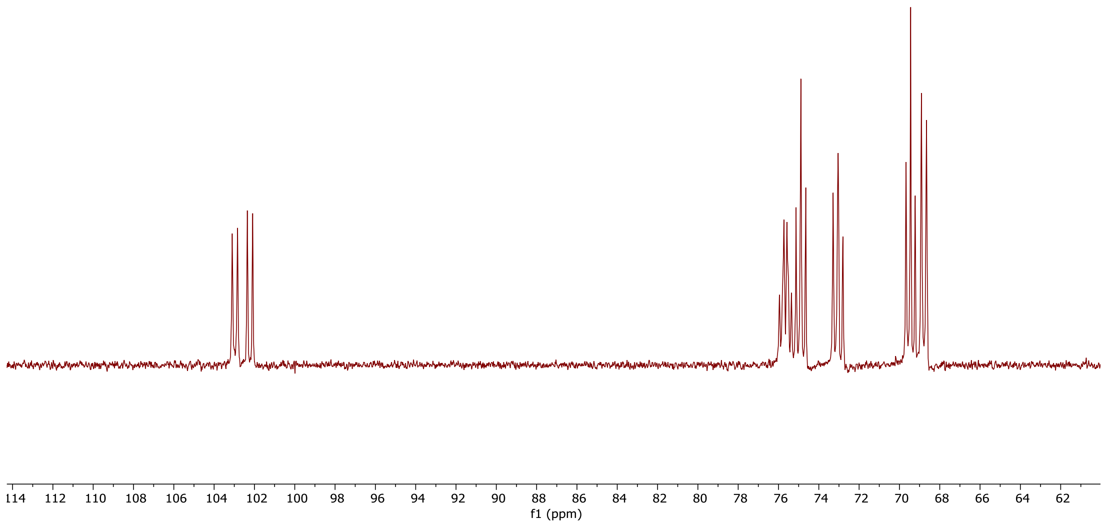

^{13}C-NMR spectrum of **5** (176 MHz, Deuterium Oxide).

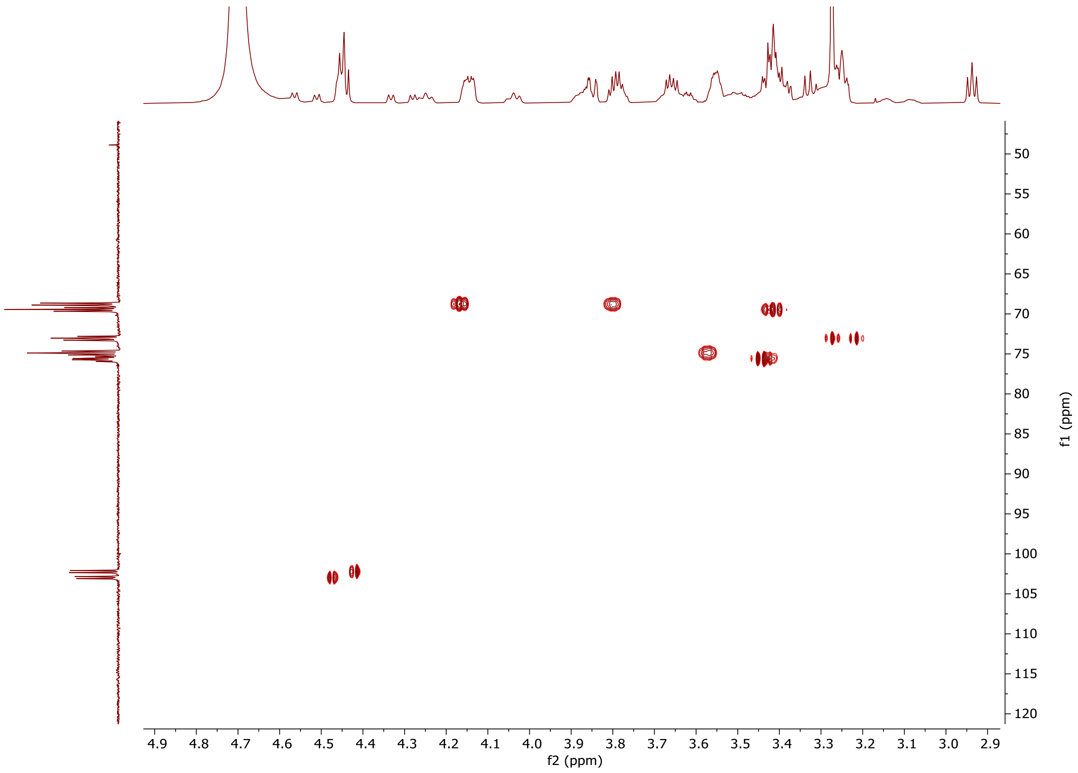

HSQC-NMR spectrum of **5** (Deuterium Oxide).

Synthesis of S1 $^{13}C_6$-G2-G3

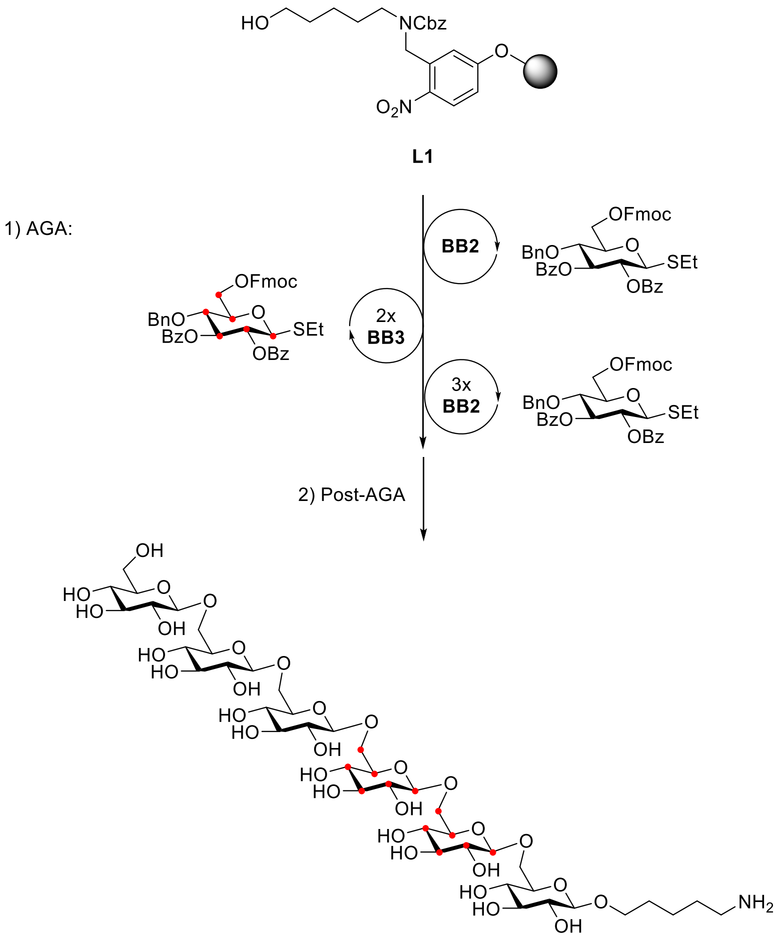

Step	BB	Modules	Notes
AGA		**A**	**L1** swelling
	**BB2**	**B**, **C**, **D**, **E**	**C**: (**BB2**, -20°C for 5 min, 0°C for 20 min)
	2x **BB3**	**B**, **C**, **D**, **E**	**C**: (**BB3**, -20°C for 5 min, 0°C for 20 min)
	3x **BB2**	**B**, **C**, **D**, **E**	**C**: (**BB2**, -20°C for 5 min, 0°C for 20 min)
Post-AGA		**F, I*, G, H, I****	**I***: (Method A, t_R = 31.6 min)<br>**G**: (16 h)<br>**H**: (20 h)<br>**I****: (Method C: 31.7 min)

Automated synthesis, global deprotection, and purification afforded **S1** as a white solid (1.8 mg, 13% overall yield).

Analytical data for **S1**: ^{1}H NMR (700 MHz, Deuterium Oxide) δ 4.59 – 4.30 (m, 2H), 4.45 (t, *J* = 7.5 Hz, 3H), 4.40 (d, *J* = 8.0 Hz, 1H), 4.28 – 4.01 (m, 2H), 4.17 – 4.12 (m, 3H), 3.86 (ddt, *J* = 12.4,

9.8, 4.7 Hz, 3H), 3.79 (dq, *J* = 12.4, 6.1 Hz, 3H), 3.71 – 3.59 (m, 2H), 3.59 – 3.47 (m, 4H), 3.46 – 3.28 (m, 8H), 3.28 – 3.22 (m, 3H), 3.19 (t, *J* = 8.6 Hz, 1H), 3.15 (d, *J* = 8.4 Hz, 1H), 2.94 (t, *J* = 7.6 Hz, 2H), 1.61 (dp, *J* = 14.4, 7.2 Hz, 4H), 1.39 (p, *J* = 7.8 Hz, 2H). ^{13}C NMR (151 MHz, Deuterium Oxide) δ 102.95 (d, *J* = 46.6, Hz), 102.91 (d, *J* = 46.6, 11.8 Hz), 75.58 (t, *J* = 39.2 Hz), 74.89 (t, *J* = 42.4 Hz), 73.02 (dd, *J* = 46.7, 39.1 Hz), 69.85 – 69.05 (m), 68.76 (d, *J* = 43.8 Hz). (ESI-HRMS) m/z 1088.463 [M+H]$^+$ ($C_{29}{}^{13}C_{12}H^{74}NO_{31}$ requires 1088.464).

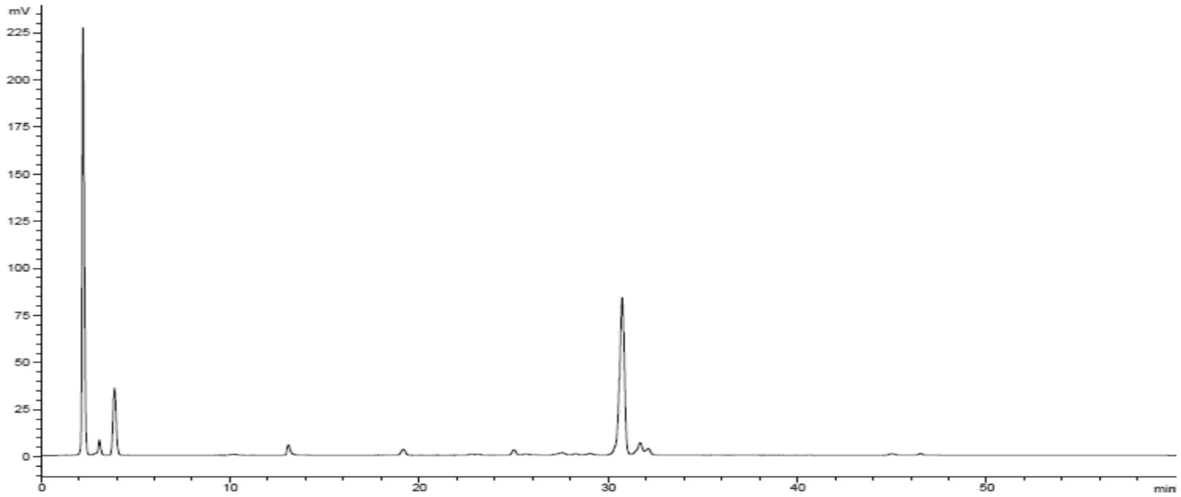

NP-HPLC of crude fully protected compound after photocleavage (Module F) (ELSD trace, Method B, t_R = 30.8 min).

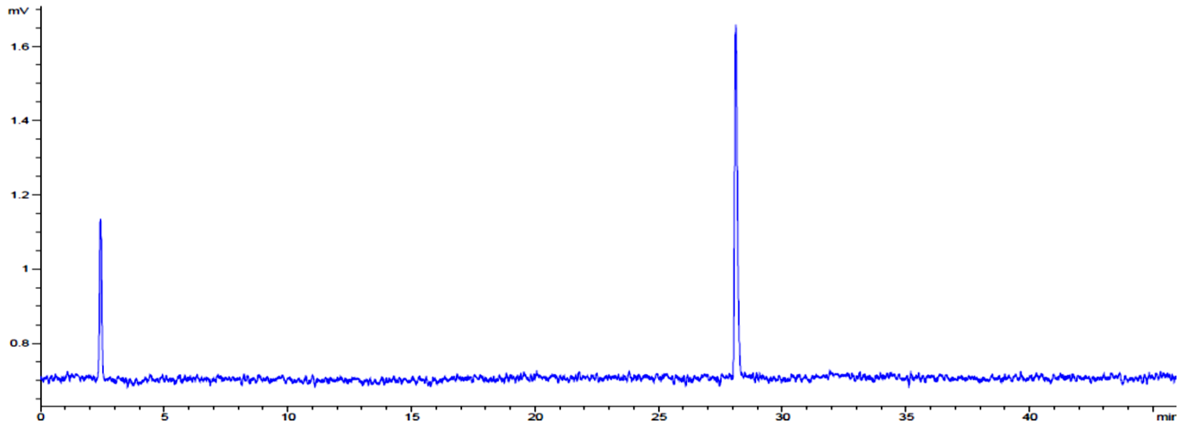

RP-HPLC of **S1** (ELSD trace, Method D, t_R = 28.7 min).

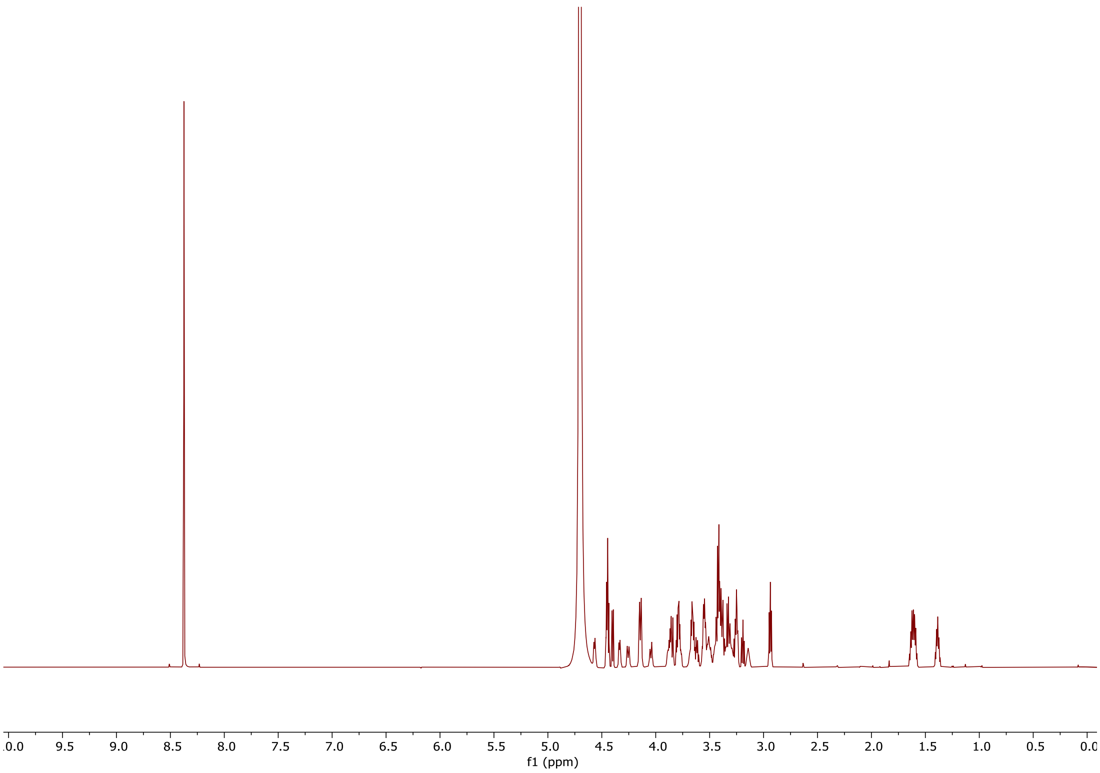

^{1}H-NMR spectrum of **S1** (700 MHz, Deuterium Oxide).

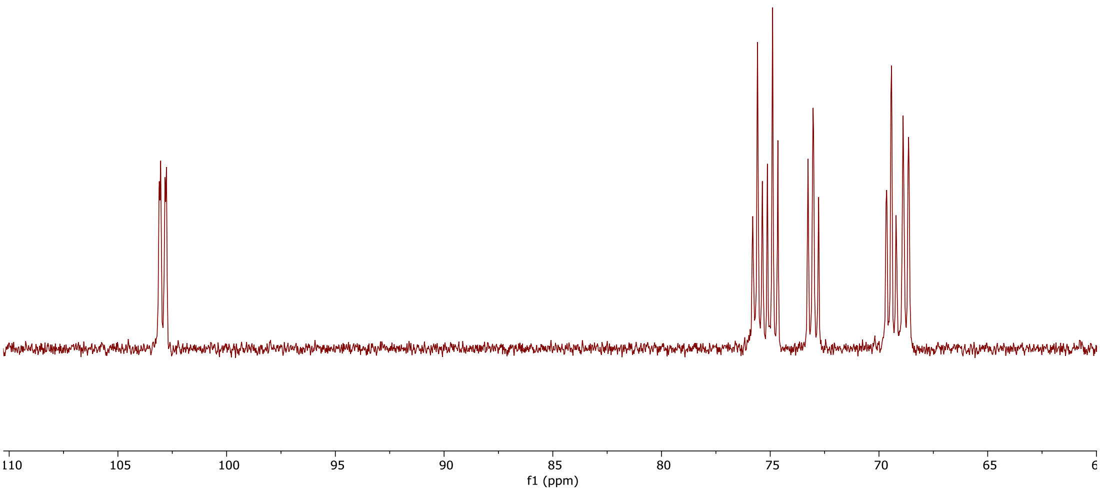

^{13}C-NMR spectrum of **S1** (176 MHz, Deuterium Oxide).

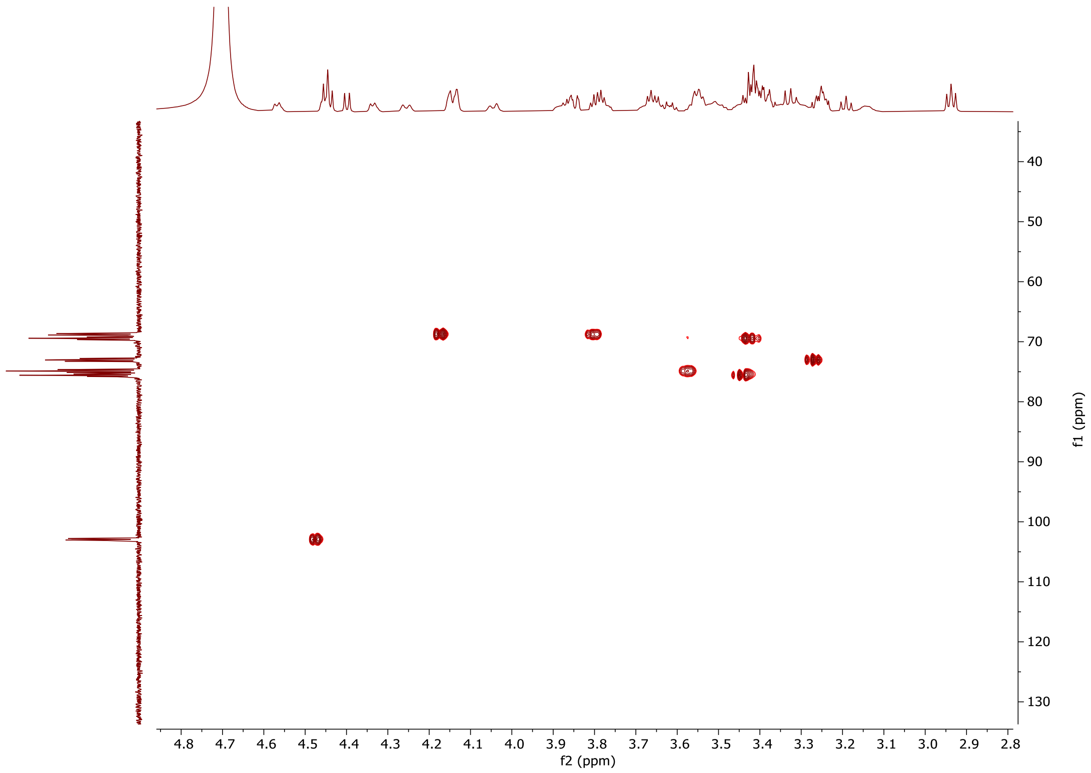

HSQC-NMR spectrum of **S1** (Deuterium Oxide).

Synthesis of 6 3F-G1-G3

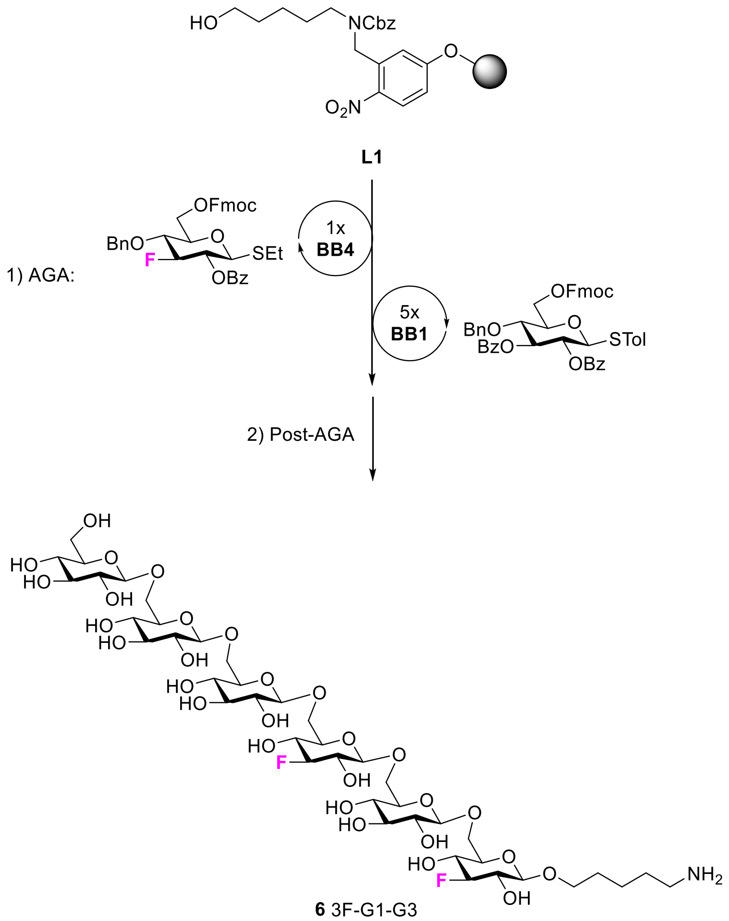

Step	BB	Modules	Notes
AGA		**A**	**L1** swelling
	3x **BB1**	**B**, **C**, **D**, **E**	**C**: (**BB1**, -20°C for 5 min, 0°C for 20 min)
	**BB4**	**B**, **C**, **D**, **E**	**C**: (**BB4**, -20°C for 5 min, 0°C for 20 min)
	2x **BB1**	**B**, **C**, **D**, **E**	**C**: (**BB1**, -20°C for 5 min, 0°C for 20 min)
Post-AGA		**F, I*, G, H, I****	**I***: (Method A, t_R = 30.2 min)<br>**G**: (16 h)<br>**H**: (20 h)<br>**I****: (Method E: 16.1 min)

Automated synthesis, global deprotection, and purification afforded **6** as a white solid (2.4 mg, 17% overall yield).

Analytical data for **6**: ^{1}H NMR (600 MHz, Deuterium Oxide) δ 4.55 (d, *J* = 8.0 Hz, 1H), 4.49 (dd, *J* = 8.1, 5.6 Hz, 6H), 4.40 – 4.31 (m, 1H), 4.20 (dd, *J* = 11.2, 5.4 Hz, 5H), 3.98 – 3.74 (m, 9H), 3.74 – 3.51 (m, 9H), 3.51 – 3.34 (m, 9H), 3.30 (t, *J* = 8.2 Hz, 4H), 2.98 (t, *J* = 7.5 Hz, 2H), 1.66 (h, *J* =

7.2 Hz, 4H), 1.43 (p, *J* = 7.9 Hz, 2H). ^{13}C NMR (151 MHz, Deuterium Oxide) δ 102.96 (d, *J* = 2.4 Hz), 102.84 (d, *J* = 2.4 Hz), 102.15, 102.07, 101.37, 101.29, 96.32 (d, *J* = 180.9 Hz), 95.17 (d, *J* = 180.9 Hz), 75.86, 75.61, 75.53, 74.87, 74.86, 74.80, 73.57, 73.53, 73.51, 73.47, 73.02, 72.98, 72.96, 71.63, 71.51, 70.29, 69.56, 69.40, 69.34, 68.89, 68.79, 68.54, 68.35, 67.77, 67.65, 60.67, 39.31, 28.10, 26.37, 22.02. ^{19}F NMR (376 MHz, Deuterium Oxide) δ -194.87 – -195.28 (m) (ESI-HRMS) m/z 1080.415 [M+H]$^+$ ($C_{41}H_{73}FNO_{30}$ requires 1078.416).

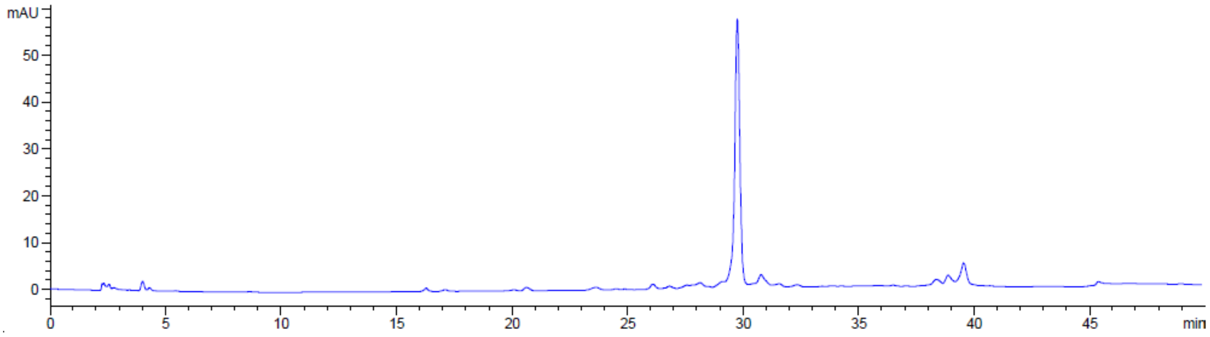

NP-HPLC of crude fully protected compound after photocleavage (Module F) (ELSD trace, Method B, t_R = 29.7 min).

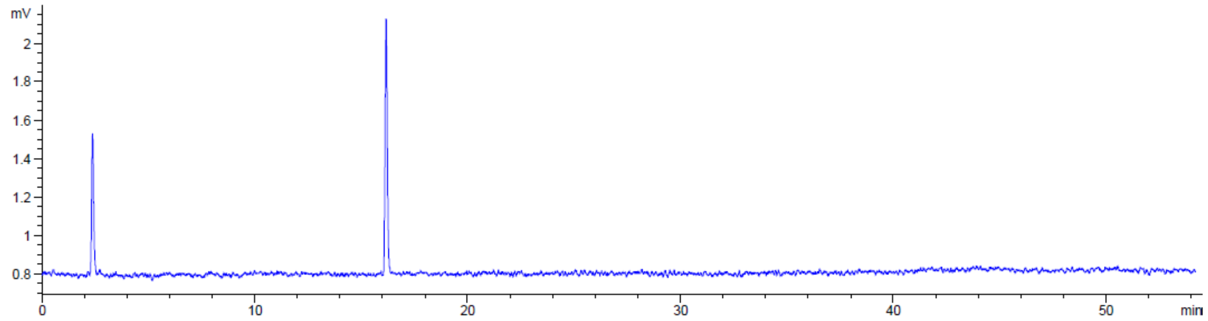

RP-HPLC of **6** (ELSD trace, Method F, t_R = 16.2 min).

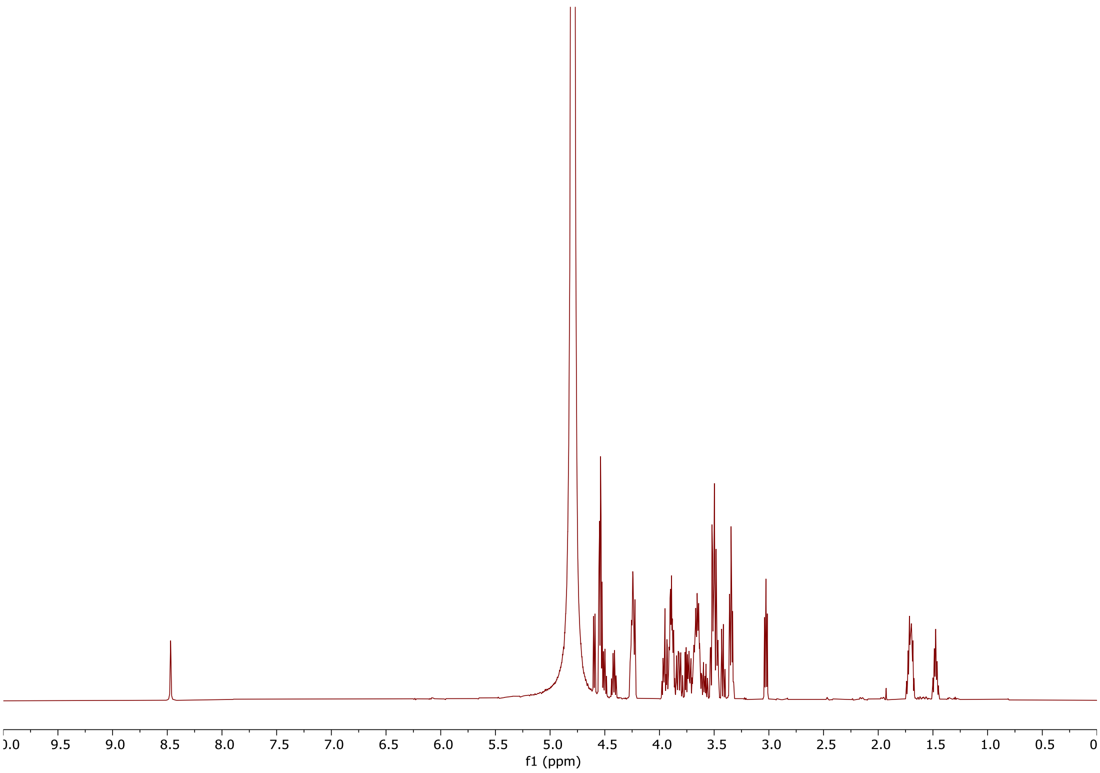

^{1}H-NMR spectrum of **6** (600 MHz, Deuterium Oxide).

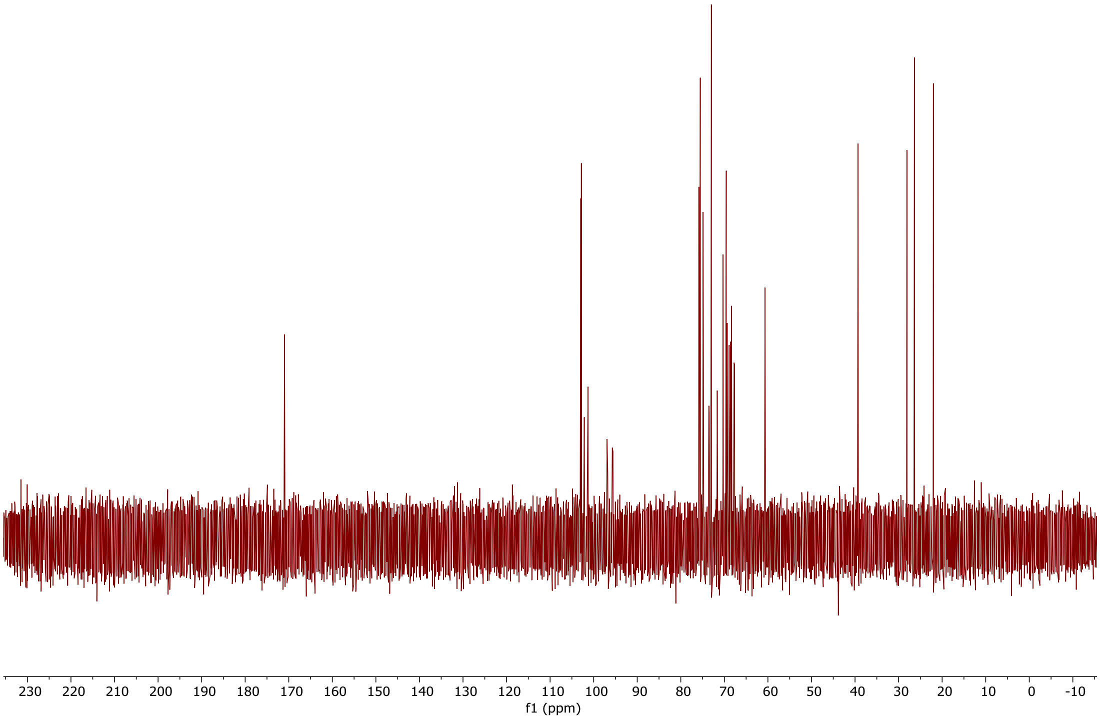

^{13}C-NMR spectrum of **6** (151 MHz, Deuterium Oxide).

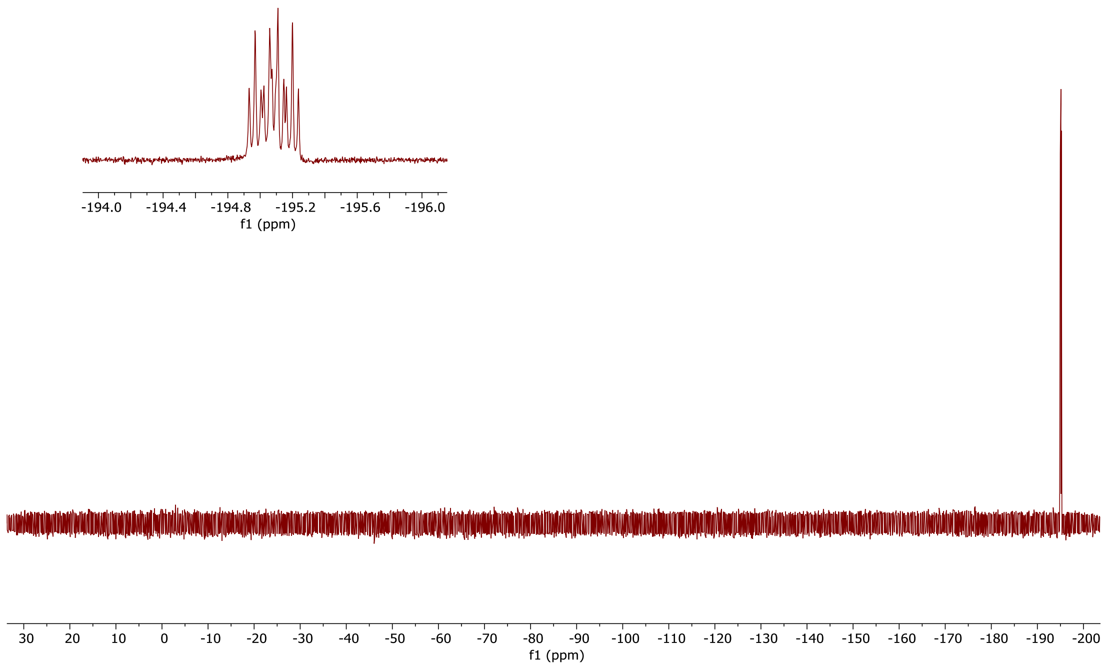

^{19}F-NMR spectrum of **6** (376 MHz, Deuterium Oxide).

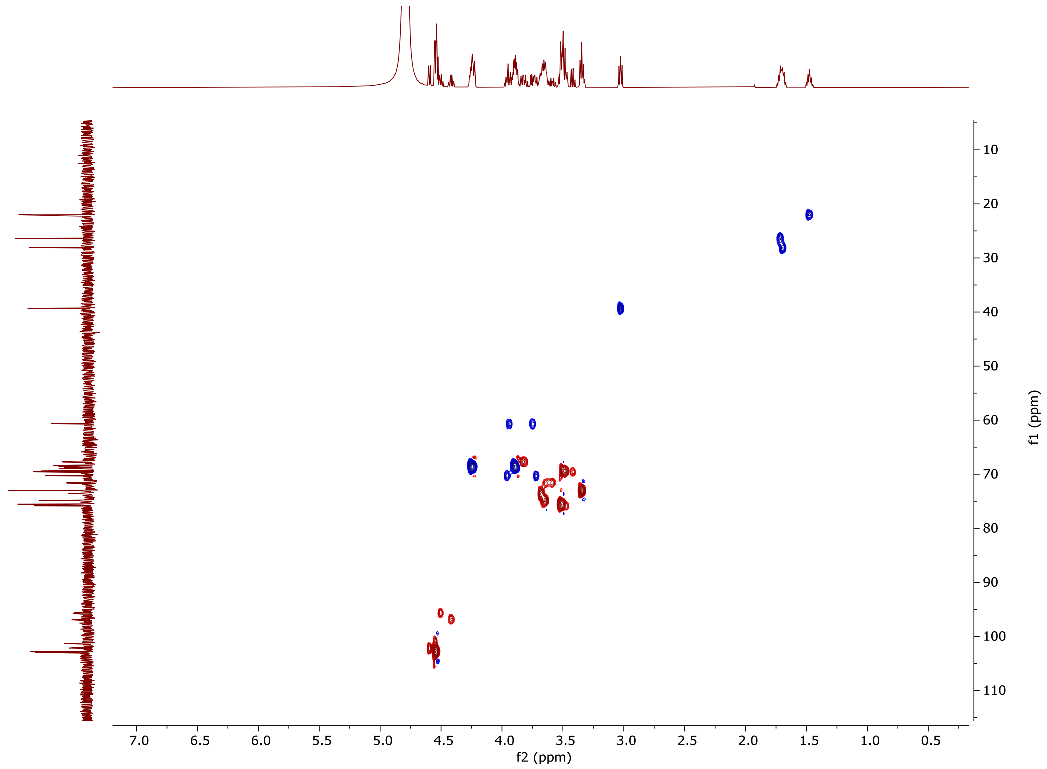

HSQC-NMR spectrum of **6** (Deuterium Oxide).

Synthesis of S2 3F-G3

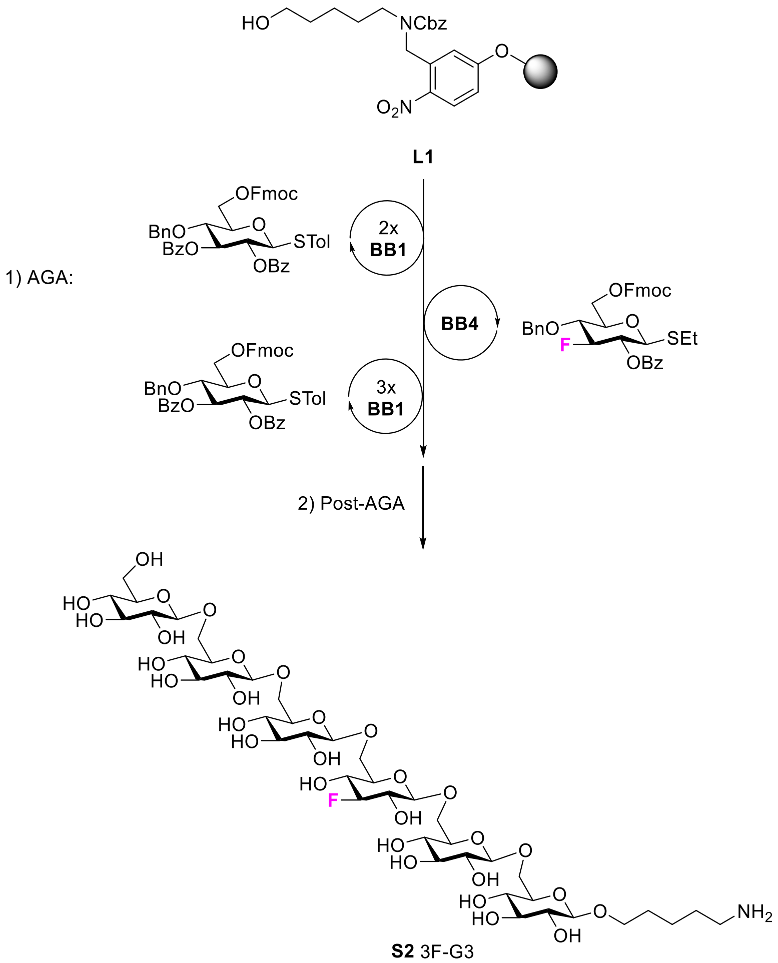

S2 3F-G3

Step	BB	Modules	Notes
AGA		**A**	**L1** swelling
	3x **BB1**	**B**, **C**, **D**, **E**	**C**: (**BB1**, -20°C for 5 min, 0°C for 20 min)
	**BB4**	**B**, **C**, **D**, **E**	**C**: (**BB4**, -20°C for 5 min, 0°C for 20 min)
	2x **BB1**	**B**, **C**, **D**, **E**	**C**: (**BB1**, -20°C for 5 min, 0°C for 20 min)
Post-AGA		**F**, **I***, **G**, **H**, **I****	**I***: (Method A, t_R = 33.5 min)<br>**G**: (16 h)<br>**H**: (20 h)<br>**I****: (Method E: 15.0 min)

Automated synthesis, global deprotection, and purification afforded **S2** as a white solid (2.4 mg, 17% overall yield).

Analytical data for **S2**: ^{1}H NMR (600 MHz, Deuterium Oxide) δ 4.60 (d, *J* = 8.0 Hz, 1H), 4.57 – 4.39 (m, 6H), 4.31 – 4.19 (m, 5H), 3.99 – 3.79 (m, 8H), 3.79 – 3.59 (m, 8H), 3.57 – 3.45 (m, 10H),

3.44 – 3.39 (m, 1H), 3.38 – 3.31 (m, 4H), 3.29 (t, J = 8.4 Hz, 1H), 3.02 (t, J = 7.6 Hz, 2H), 1.76 – 1.64 (m, 4H), 1.52 – 1.43 (m, 2H). ^{13}C NMR (151 MHz, Deuterium Oxide) δ 102.95, 102.83, 102.16, 102.06, 96.15 (d, J = 180.6 Hz), 75.85, 75.67, 75.60, 75.51, 74.85, 74.80, 73.56, 73.50, 73.01, 72.97, 71.63, 71.50, 70.13, 69.56, 69.38, 68.86, 68.77, 68.69, 68.54, 68.35, 67.76, 67.64, 60.66, 39.33, 28.13, 26.50, 22.04. ^{19}F NMR (376 MHz, Deuterium Oxide) δ -195.13 (dt, J = 52.9, 13.7 Hz). (ESI-HRMS) m/z 1078.420 $[M+H]^+$ ($C_{41}H_{73}FNO_{30}$ requires 1078.420).

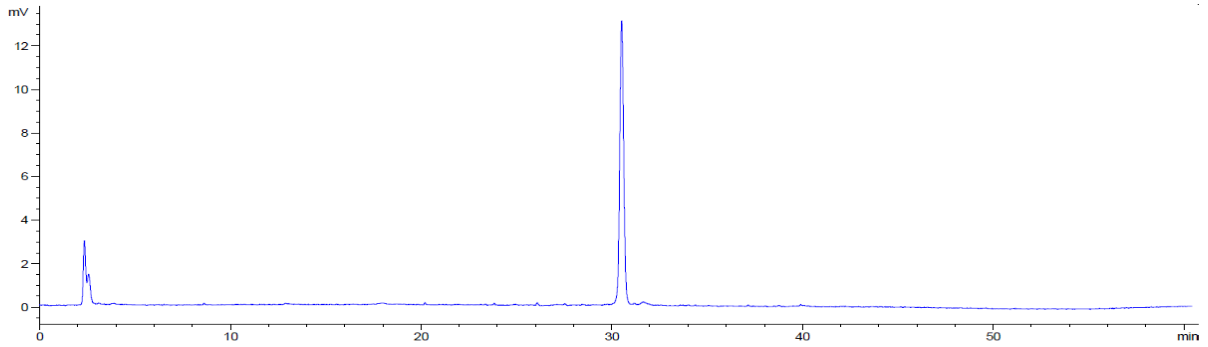

NP-HPLC of crude fully protected compound after photocleavage (Module F) (ELSD trace, Method B, t_R = 30.5 min).

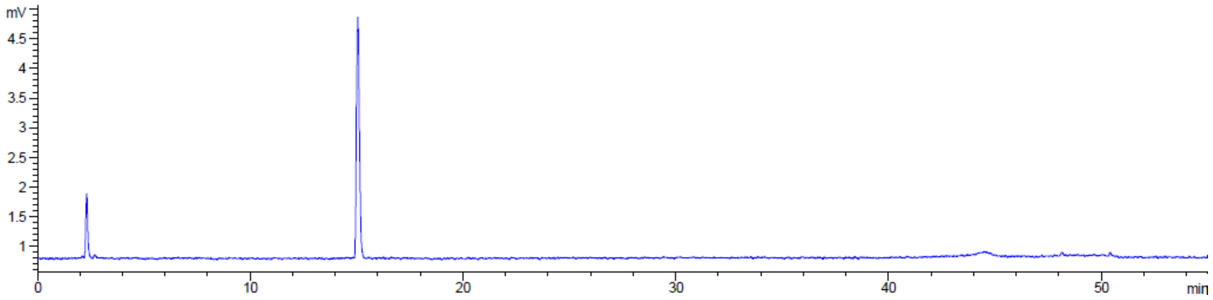

RP-HPLC of **S2** (ELSD trace, Method F, t_R = 15.0 min).

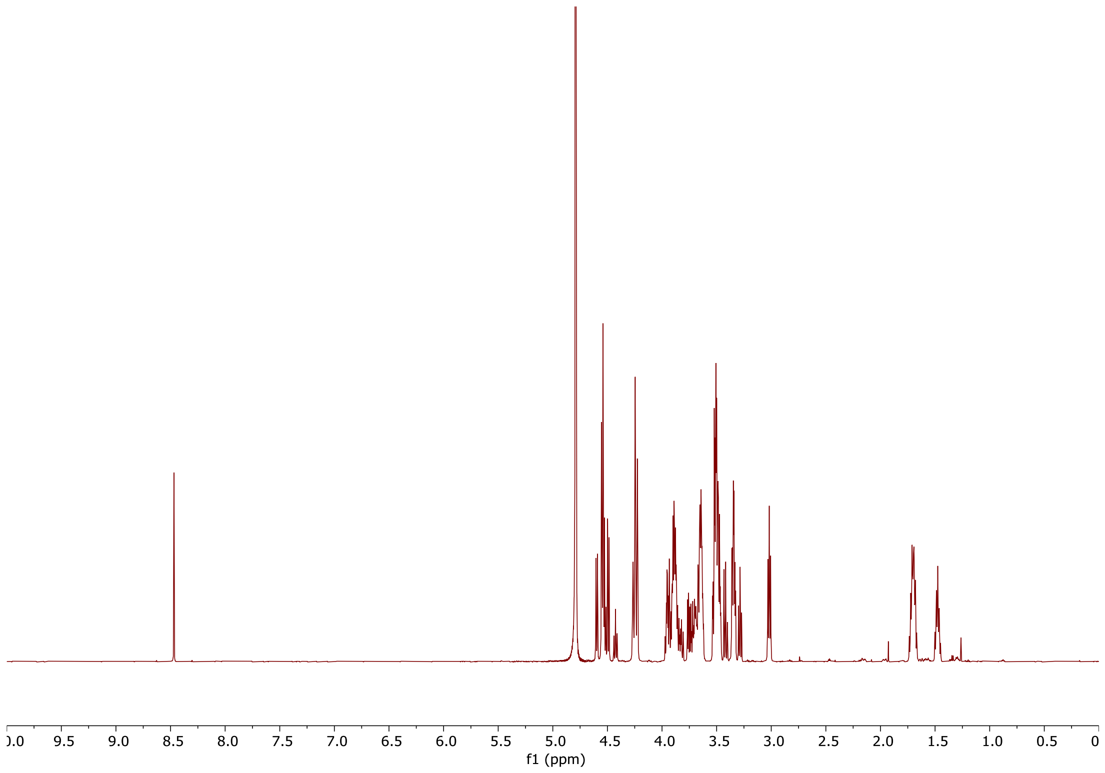

^{1}H-NMR spectrum of **S2** (600 MHz, Deuterium Oxide).

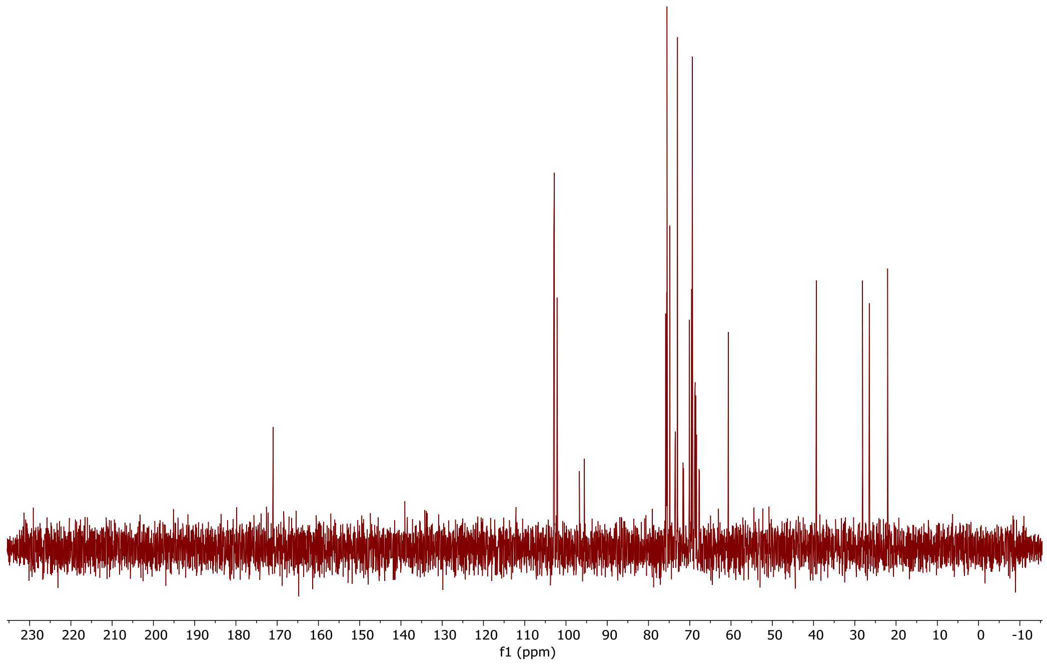

^{13}C-NMR spectrum of **S2** (151 MHz, Deuterium Oxide).

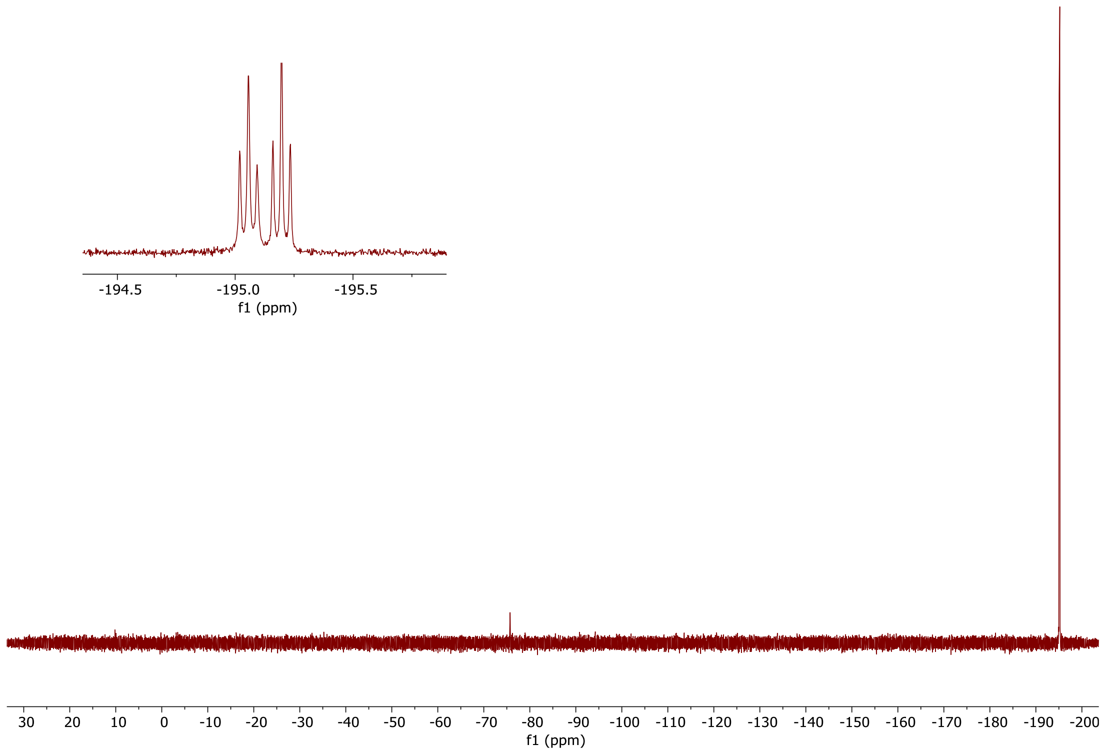

^{19}F-NMR spectrum of **S2** (376 MHz, Deuterium Oxide).

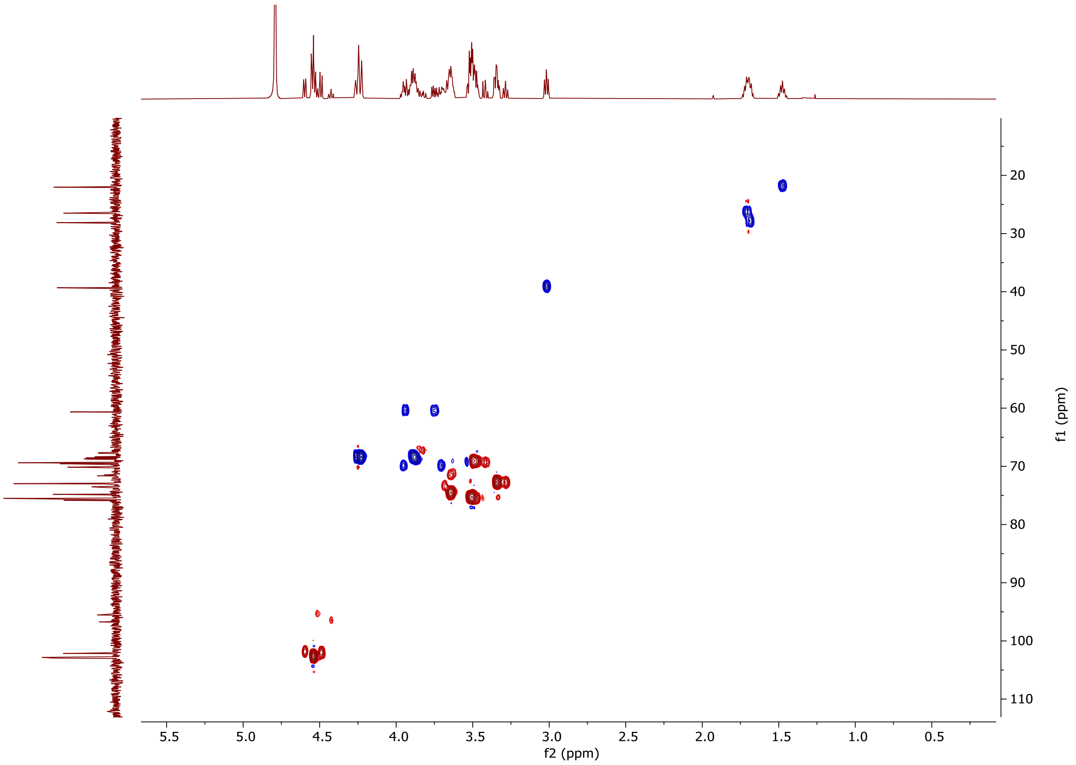

HSQC-NMR spectrum of **S2** (Deuterium Oxide).

Synthesis of S3 3F-G1

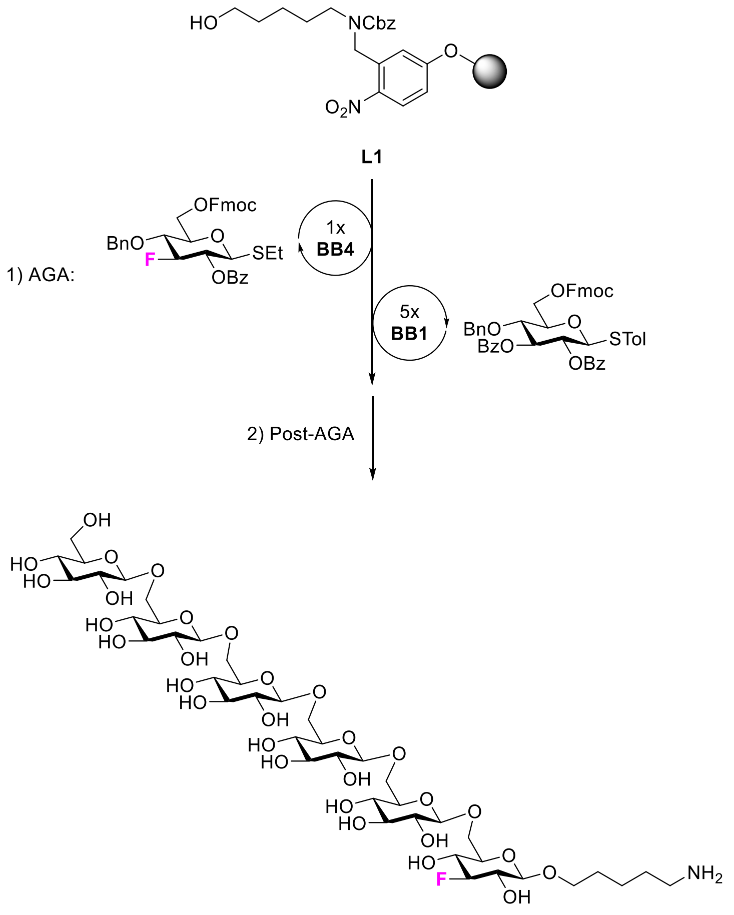

S3 3F-G1

Step	BB	Modules	Notes
AGA		**A**	**L1** swelling
	3x **BB1**	**B**, **C**, **D**, **E**	**C**: (**BB1**, -20°C for 5 min, 0°C for 20 min)
	**BB4**	**B**, **C**, **D**, **E**	**C**: (**BB4**, -20°C for 5 min, 0°C for 20 min)
	2x **BB1**	**B**, **C**, **D**, **E**	**C**: (**BB1**, -20°C for 5 min, 0°C for 20 min)
Post-AGA		**F, I*, G, H, I****	**I***: (Method A, t_R = 29.6 min)<br>**G**: (16 h)<br>**H**: (20 h)<br>**I****: (Method E: 14.4 min)

Automated synthesis, global deprotection, and purification afforded **S3** as a white solid (3.8 mg, 21% overall yield).

Analytical data for **S3**: ^{1}H NMR (600 MHz, Deuterium Oxide) δ 4.57 – 4.52 (m, 6H), 4.46 (dt, *J* = 52.8, 8.9 Hz, 1H, H-3), 4.24 (dq, *J* = 11.3, 3.0, 2.4 Hz, 5H), 3.99 – 3.85 (m, 7H), 3.81 (ddd, *J* = 13.8, 10.1, 8.8 Hz, 1H), 3.77 – 3.62 (m, 7H), 3.62 – 3.55 (m, 1H), 3.55 – 3.45 (m, 10H), 3.45 – 3.39

(m, 1H), 3.34 (ddt, *J* = 9.6, 7.9, 2.1 Hz, 5H), 3.04 – 2.99 (m, 2H), 1.70 (h, *J* = 7.2, 6.7 Hz, 4H), 1.47 (p, *J* = 7.7, 7.2 Hz, 2H). ^{13}C NMR (151 MHz, Deuterium Oxide) δ 102.95, 102.92, 102.88, 102.83, 101.37, 101.29, 96.32 (d, *J* = 181.0 Hz, C-3), 75.86, 75.60, 75.52, 74.87, 74.84, 74.82, 73.56, 73.50, 73.02, 72.98, 71.62, 71.50, 70.31, 69.56, 69.41, 69.39, 69.35, 68.76, 68.72, 68.70, 68.54, 68.35, 67.78, 67.66, 60.66, 39.34, 28.11, 26.53, 22.03. ^{19}F NMR (376 MHz, Deuterium Oxide) δ -195.04 (dt, *J* = 52.7, 13.9 Hz). (ESI-HRMS) m/z 1078.419 $[M+H]^+$ ($C_{41}H_{73}FNO_{30}$ requires 1078.420).

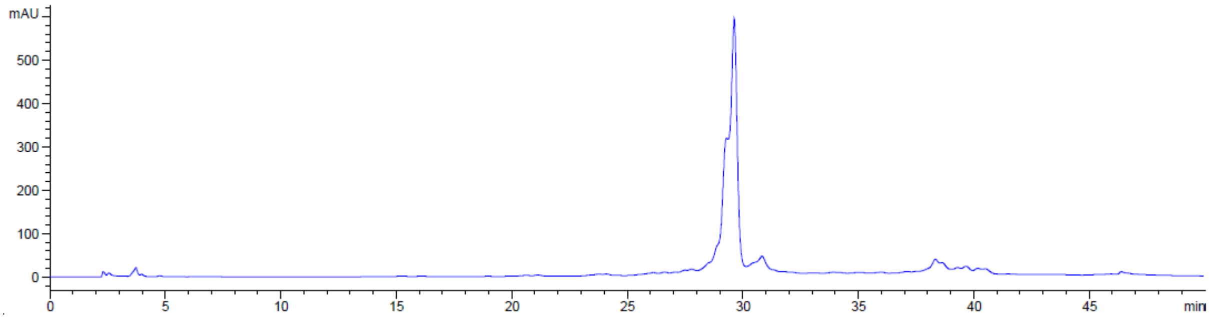

NP-HPLC of crude fully protected compound after photocleavage (Module F) (ELSD trace, Method B, t_R = 29.6 min).

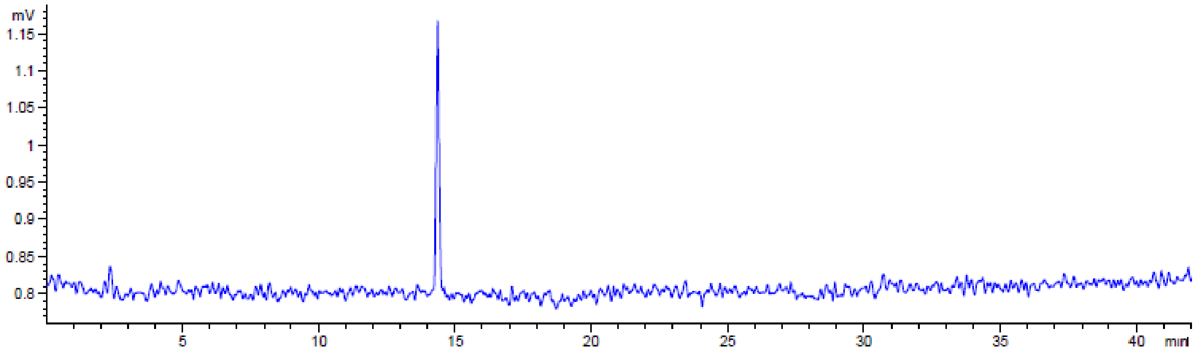

RP-HPLC of **S3** (ELSD trace, Method F, t_R = 15.3 min).

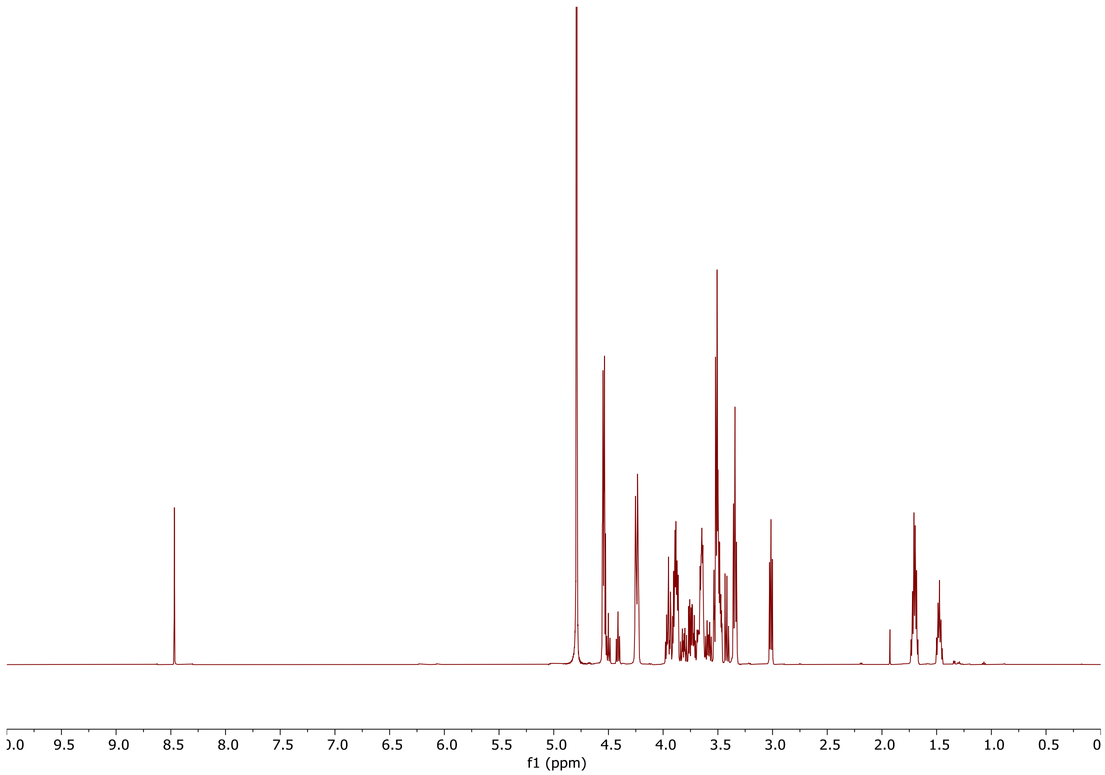

^{1}H-NMR spectrum of **S3** (600 MHz, Deuterium Oxide).

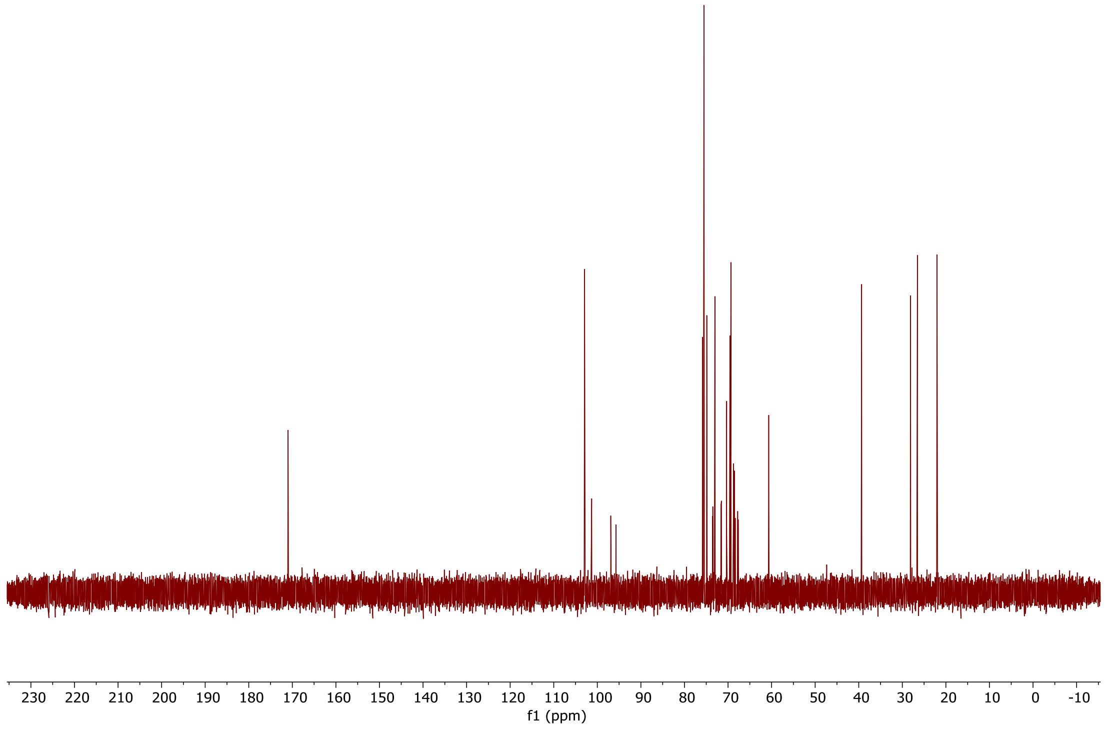

^{13}C-NMR spectrum of **S3** (151 MHz, Deuterium Oxide).

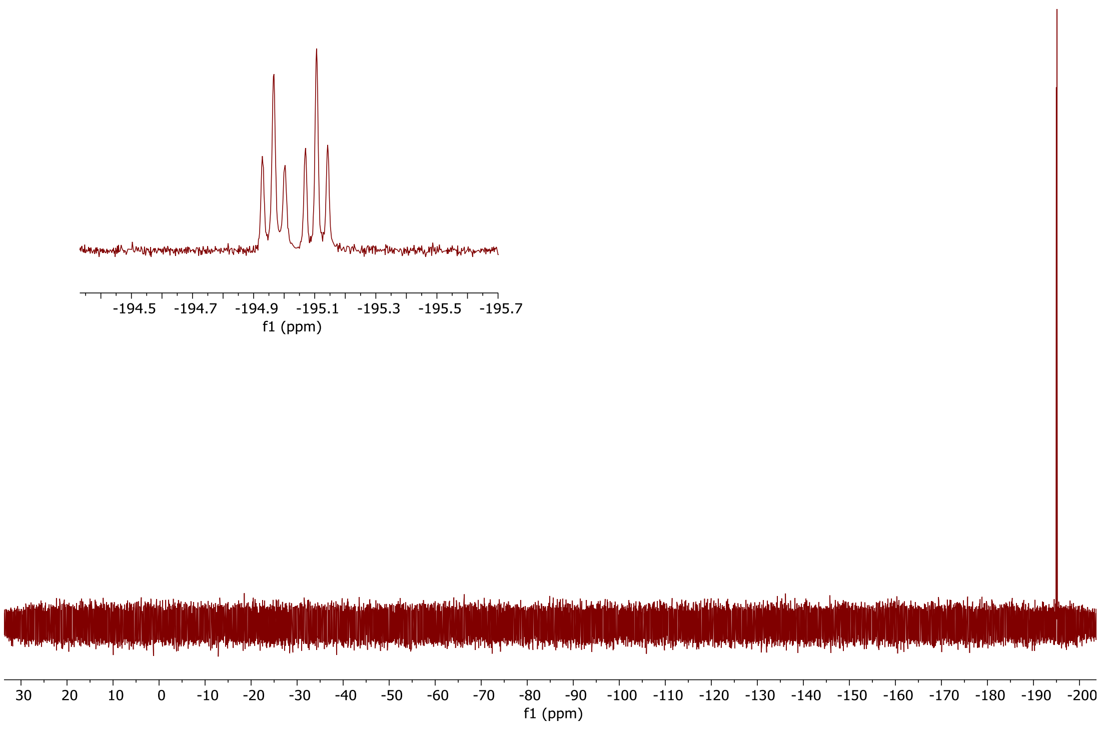

^{19}F-NMR spectrum of **S3** (376 MHz, Deuterium Oxide).

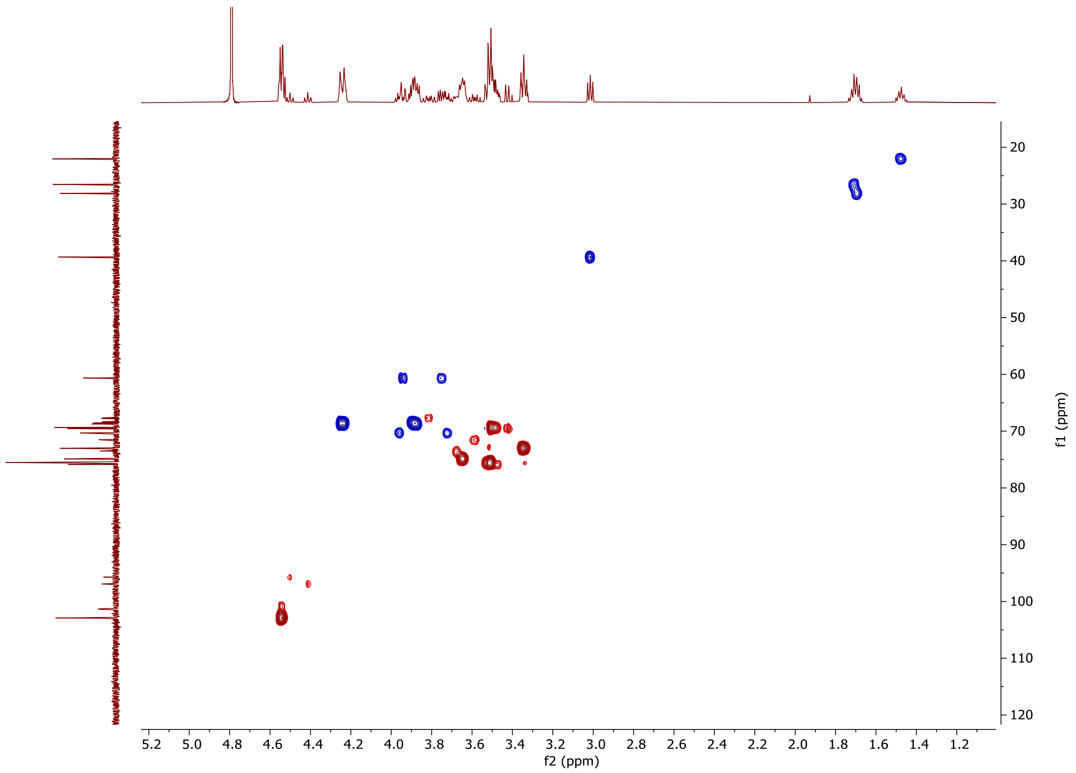

HSQC-NMR spectrum of **S3** (Deuterium Oxide).

References

(1) Gude, M.; Ryf, J.; White, P. D. An Accurate Method for the Quantitation of Fmoc-Derivatized Solid Phase Supports. *Lett. Pept. Sci.* **2002**, *9* (4), 203–206. https://doi.org/doi.org/10.1023/A:1024148619149.

(2) Delbianco, M.; Kononov, A.; Poveda, A.; Yu, Y.; Diercks, T.; Jiménez-Barbero, J.; Seeberger, P. H. Well-Defined Oligo- and Polysaccharides as Ideal Probes for Structural Studies. *J. Am. Chem. Soc.* **2018**, *140* (16), 5421–5426. https://doi.org/10.1021/jacs.8b00254.

(3) Gim, S.; Fittolani, G.; Yu, Y.; Zhu, Y.; Seeberger, P. H.; Ogawa, Y.; Delbianco, M. Targeted Chemical Modifications Identify Key Features of Carbohydrate Assemblies and Generate Tailored Carbohydrate Materials. *Chem. – A Eur. J.* **2021**, chem.202102164. https://doi.org/10.1002/chem.202102164.

(4) Hurevich, M.; Kandasamy, J.; Ponnappa, B. M.; Collot, M.; Kopetzki, D.; McQuade, D. T.; Seeberger, P. H. Continuous Photochemical Cleavage of Linkers for Solid-Phase Synthesis. *Org. Lett.* **2014**, *16* (6), 1794–1797. https://doi.org/10.1021/ol500530q.
