# Supplementary material for: The Flexibility of Oligosaccharides Unveiled Through Residual Dipolar Coupling Analysis
Source: Front Mol Biosci. 2021 Nov 10;8:784318. doi: 10.3389/fmolb.2021.784318 (PMC8631391; doi:10.3389/fmolb.2021.784318)
Supplement: Supplementary file 2 [file DataSheet2.docx]

MSPIN raw data

---------------------------------------

gg6

CR_all

!* MSpin-RDC Plugin *!

********

!* Computation flags *!

Method: SVD

Scaling mode: Hz

Field (T): 18.7923

1H Larmor Frequency: 800.13

Scale QCSA with axial component: False

Include CSA gel shift (isotropic) correction:False

Optimize CSA gel shift (isotropic) correction scale:False

Estimate CSA gel shift (isotropic) correction scale:False

Gel Shift Correction Scale: 0.15

Single Tensor: False

Superimpose: False

Average methyl groups: False

Average methylene groups: False

Average phenyl groups: False

Bootstrapping: False

RDC Std. Error [ppm]: 1

CSA Std. Error [ppm]: 0.01

PCS Std. Error [ppm]: 0.01

DQ Std. Error [Hz]: 1

********

!* Permutations *!

There are 16 possible permutations on the original data set

********

Data set: #1

Computed data for frame #1

RDC Data:

I J Exp. [Hz] Comp. [Hz]

C5 H16 23.00 17.50

C12 H22 25.20 20.59

C14 H24 10.30 1.95

C14 H25 3.60 -0.49

C26 H37 -19.00 -9.96

C33 H43 -23.00 -15.18

C31 H41 -21.90 -12.97

C29 H39 -23.90 -8.86

C28 H38 -19.50 -17.76

C35 H45 21.30 1.13

C35 H46 -10.20 10.12

C47 H58 -25.00 -8.36

C54 H64 -19.70 -4.36

C56 H66 0.00 -6.27

C56 H67 2.90 -19.17

C68 H79 -4.20 3.08

C75 H85 -1.20 -1.61

C73 H83 -5.00 -0.06

C71 H81 -5.30 4.79

C70 H80 -4.50 -2.32

C77 H87 -1.30 9.88

C77 H88 4.30 11.26

Cornilescu Quality factor: 0.72787

Alignment tensor information:

A'x= 4.640e-04

A'y= 1.215e-03

A'z=-1.679e-03

Saupe tensor

S'x= 6.960e-04

S'y= 1.823e-03

S'z=-2.519e-03

Alignment tensor eigenvectors

e[x]=(-0.475, 0.737,-0.482)

e[y]=(-0.559, 0.170, 0.811)

e[z]=( 0.680, 0.654, 0.331)

Alignment tensor in laboratory coordinates:

[-2.916e-04,-1.025e-03,-8.233e-04]

[-1.025e-03,-4.319e-04,-3.606e-04]

[-8.233e-04,-3.606e-04,7.235e-04]

SVD condition number is 1.507e+01

Axial component Aa = -2.519e-03

Rhombic component Ar = -7.512e-04

Field=18.79 Teslas[ 2.27]

rhombicity R = 0.298

Asimmetry parameter etha =4.474e-01

GDO = 3.050e-03

ZY'Z'' Euler Angles (degrees)

Set 1

(43.9,70.7,59.3)

Set 2

(-136.1,-70.7,-120.7)

********Data set: #2

Computed data for frame #1

RDC Data:

I J Exp. [Hz] Comp. [Hz]

C5 H16 23.00 17.27

C12 H22 25.20 20.08

C14 H25 10.30 4.60

C14 H24 3.60 -2.41

C26 H37 -19.00 -11.34

C33 H43 -23.00 -15.94

C31 H41 -21.90 -14.07

C29 H39 -23.90 -10.07

C28 H38 -19.50 -17.84

C35 H45 21.30 -0.82

C35 H46 -10.20 11.48

C47 H58 -25.00 -7.72

C54 H64 -19.70 -3.79

C56 H66 0.00 -4.87

C56 H67 2.90 -18.30

C68 H79 -4.20 2.33

C75 H85 -1.20 -1.74

C73 H83 -5.00 -0.53

C71 H81 -5.30 4.12

C70 H80 -4.50 -2.01

C77 H87 -1.30 8.31

C77 H88 4.30 11.33

Cornilescu Quality factor: 0.723893

Alignment tensor information:

A'x= 2.861e-04

A'y= 1.162e-03

A'z=-1.448e-03

Saupe tensor

S'x= 4.292e-04

S'y= 1.742e-03

S'z=-2.172e-03

Alignment tensor eigenvectors

e[x]=(-0.467, 0.730,-0.499)

e[y]=(-0.567, 0.186, 0.802)

e[z]=( 0.679, 0.658, 0.327)

Alignment tensor in laboratory coordinates:

[-2.305e-04,-8.663e-04,-7.832e-04]

[-8.663e-04,-4.335e-04,-2.422e-04]

[-7.832e-04,-2.422e-04,6.641e-04]

SVD condition number is 1.507e+01

Axial component Aa = -2.172e-03

Rhombic component Ar = -8.755e-04

Field=18.79 Teslas[ 2.27]

rhombicity R = 0.403

Asimmetry parameter etha =6.047e-01

GDO = 2.727e-03

ZY'Z'' Euler Angles (degrees)

Set 1

(44.1,70.9,58.1)

Set 2

(-135.9,-70.9,-121.9)

********Data set: #3

Computed data for frame #1

RDC Data:

I J Exp. [Hz] Comp. [Hz]

C5 H16 23.00 25.50

C12 H22 25.20 28.73

C14 H24 10.30 -3.49

C14 H25 3.60 9.63

C26 H37 -19.00 -9.73

C33 H43 -23.00 -14.65

C31 H41 -21.90 -12.79

C29 H39 -23.90 -7.80

C28 H38 -19.50 -16.06

C35 H46 21.30 20.89

C35 H45 -10.20 -11.83

C47 H58 -25.00 -16.76

C54 H64 -19.70 -12.20

C56 H66 0.00 -4.92

C56 H67 2.90 -15.22

C68 H79 -4.20 1.13

C75 H85 -1.20 -2.91

C73 H83 -5.00 -1.87

C71 H81 -5.30 3.23

C70 H80 -4.50 -2.78

C77 H87 -1.30 10.51

C77 H88 4.30 5.29

Cornilescu Quality factor: 0.530789

Alignment tensor information:

A'x= 8.753e-05

A'y= 1.359e-03

A'z=-1.447e-03

Saupe tensor

S'x= 1.313e-04

S'y= 2.039e-03

S'z=-2.170e-03

Alignment tensor eigenvectors

e[x]=(-0.429, 0.764,-0.483)

e[y]=(-0.583, 0.174, 0.793)

e[z]=( 0.690, 0.622, 0.371)

Alignment tensor in laboratory coordinates:

[-2.105e-04,-7.875e-04,-9.807e-04]

[-7.875e-04,-4.668e-04,-1.776e-04]

[-9.807e-04,-1.776e-04,6.773e-04]

SVD condition number is 1.507e+01

Axial component Aa = -2.170e-03

Rhombic component Ar = -1.272e-03

Field=18.79 Teslas[ 2.27]

rhombicity R = 0.586

Asimmetry parameter etha =8.790e-01

GDO = 2.950e-03

ZY'Z'' Euler Angles (degrees)

Set 1

(42.0,68.2,58.7)

Set 2

(-138.0,-68.2,-121.3)

********Data set: #4

Computed data for frame #1

RDC Data:

I J Exp. [Hz] Comp. [Hz]

C5 H16 23.00 25.27

C12 H22 25.20 28.22

C14 H25 10.30 14.72

C14 H24 3.60 -7.85

C26 H37 -19.00 -11.11

C33 H43 -23.00 -15.41

C31 H41 -21.90 -13.89

C29 H39 -23.90 -9.01

C28 H38 -19.50 -16.14

C35 H46 21.30 22.25

C35 H45 -10.20 -13.78

C47 H58 -25.00 -16.12

C54 H64 -19.70 -11.63

C56 H66 0.00 -3.51

C56 H67 2.90 -14.35

C68 H79 -4.20 0.38

C75 H85 -1.20 -3.03

C73 H83 -5.00 -2.34

C71 H81 -5.30 2.56

C70 H80 -4.50 -2.46

C77 H87 -1.30 8.95

C77 H88 4.30 5.37

Cornilescu Quality factor: 0.486404

Alignment tensor information:

A'x=-9.066e-05

A'y=-1.215e-03

A'z= 1.306e-03

Saupe tensor

S'x=-1.360e-04

S'y=-1.823e-03

S'z= 1.959e-03

Alignment tensor eigenvectors

e[x]=( 0.415,-0.768, 0.487)

e[y]=( 0.694, 0.614, 0.376)

e[z]=(-0.588, 0.182, 0.788)

Alignment tensor in laboratory coordinates:

[-1.494e-04,-6.289e-04,-9.407e-04]

[-6.289e-04,-4.684e-04,-5.913e-05]

[-9.407e-04,-5.913e-05,6.179e-04]

SVD condition number is 1.507e+01

Axial component Aa = 1.959e-03

Rhombic component Ar = 1.125e-03

Field=18.79 Teslas[ 2.27]

rhombicity R = 0.574

Asimmetry parameter etha =8.612e-01

GDO = 2.649e-03

ZY'Z'' Euler Angles (degrees)

Set 1

(162.8,38.0,142.4)

Set 2

(-17.2,-38.0,-37.6)

********Data set: #5

Computed data for frame #1

RDC Data:

I J Exp. [Hz] Comp. [Hz]

C5 H16 23.00 17.44

C12 H22 25.20 20.50

C14 H24 10.30 1.81

C14 H25 3.60 -0.40

C26 H37 -19.00 -10.39

C33 H43 -23.00 -15.62

C31 H41 -21.90 -13.39

C29 H39 -23.90 -9.34

C28 H38 -19.50 -18.26

C35 H45 21.30 1.27

C35 H46 -10.20 10.02

C47 H58 -25.00 -8.40

C54 H64 -19.70 -4.43

C56 H67 0.00 -19.76

C56 H66 2.90 -5.77

C68 H79 -4.20 3.60

C75 H85 -1.20 -1.08

C73 H83 -5.00 0.47

C71 H81 -5.30 5.29

C70 H80 -4.50 -1.81

C77 H87 -1.30 10.17

C77 H88 4.30 11.11

Cornilescu Quality factor: 0.717682

Alignment tensor information:

A'x= 4.724e-04

A'y= 1.207e-03

A'z=-1.679e-03

Saupe tensor

S'x= 7.085e-04

S'y= 1.811e-03

S'z=-2.519e-03

Alignment tensor eigenvectors

e[x]=(-0.483, 0.738,-0.471)

e[y]=(-0.552, 0.161, 0.818)

e[z]=( 0.679, 0.656, 0.330)

Alignment tensor in laboratory coordinates:

[-2.970e-04,-1.024e-03,-8.138e-04]

[-1.024e-03,-4.333e-04,-3.684e-04]

[-8.138e-04,-3.684e-04,7.304e-04]

SVD condition number is 1.507e+01

Axial component Aa = -2.519e-03

Rhombic component Ar = -7.347e-04

Field=18.79 Teslas[ 2.27]

rhombicity R = 0.292

Asimmetry parameter etha =4.375e-01

GDO = 3.045e-03

ZY'Z'' Euler Angles (degrees)

Set 1

(44.0,70.8,60.1)

Set 2

(-136.0,-70.8,-119.9)

********Data set: #6

Computed data for frame #1

RDC Data:

I J Exp. [Hz] Comp. [Hz]

C5 H16 23.00 17.21

C12 H22 25.20 19.99

C14 H25 10.30 4.68

C14 H24 3.60 -2.55

C26 H37 -19.00 -11.77

C33 H43 -23.00 -16.38

C31 H41 -21.90 -14.50

C29 H39 -23.90 -10.55

C28 H38 -19.50 -18.33

C35 H45 21.30 -0.68

C35 H46 -10.20 11.38

C47 H58 -25.00 -7.76

C54 H64 -19.70 -3.86

C56 H67 0.00 -18.89

C56 H66 2.90 -4.36

C68 H79 -4.20 2.85

C75 H85 -1.20 -1.20

C73 H83 -5.00 -0.00

C71 H81 -5.30 4.62

C70 H80 -4.50 -1.49

C77 H87 -1.30 8.60

C77 H88 4.30 11.19

Cornilescu Quality factor: 0.713239

Alignment tensor information:

A'x= 2.950e-04

A'y= 1.153e-03

A'z=-1.448e-03

Saupe tensor

S'x= 4.425e-04

S'y= 1.730e-03

S'z=-2.172e-03

Alignment tensor eigenvectors

e[x]=(-0.475, 0.731,-0.491)

e[y]=(-0.561, 0.178, 0.808)

e[z]=( 0.678, 0.659, 0.325)

Alignment tensor in laboratory coordinates:

[-2.360e-04,-8.650e-04,-7.738e-04]

[-8.650e-04,-4.349e-04,-2.500e-04]

[-7.738e-04,-2.500e-04,6.709e-04]

SVD condition number is 1.507e+01

Axial component Aa = -2.172e-03

Rhombic component Ar = -8.582e-04

Field=18.79 Teslas[ 2.27]

rhombicity R = 0.395

Asimmetry parameter etha =5.926e-01

GDO = 2.720e-03

ZY'Z'' Euler Angles (degrees)

Set 1

(44.2,71.0,58.7)

Set 2

(-135.8,-71.0,-121.3)

********Data set: #7

Computed data for frame #1

RDC Data:

I J Exp. [Hz] Comp. [Hz]

C5 H16 23.00 25.44

C12 H22 25.20 28.64

C14 H24 10.30 -3.63

C14 H25 3.60 9.72

C26 H37 -19.00 -10.16

C33 H43 -23.00 -15.09

C31 H41 -21.90 -13.22

C29 H39 -23.90 -8.28

C28 H38 -19.50 -16.55

C35 H46 21.30 20.79

C35 H45 -10.20 -11.69

C47 H58 -25.00 -16.80

C54 H64 -19.70 -12.27

C56 H67 0.00 -15.81

C56 H66 2.90 -4.42

C68 H79 -4.20 1.65

C75 H85 -1.20 -2.37

C73 H83 -5.00 -1.34

C71 H81 -5.30 3.73

C70 H80 -4.50 -2.26

C77 H87 -1.30 10.80

C77 H88 4.30 5.15

Cornilescu Quality factor: 0.519477

Alignment tensor information:

A'x= 9.603e-05

A'y= 1.350e-03

A'z=-1.447e-03

Saupe tensor

S'x= 1.440e-04

S'y= 2.026e-03

S'z=-2.170e-03

Alignment tensor eigenvectors

e[x]=(-0.435, 0.763,-0.478)

e[y]=(-0.579, 0.169, 0.797)

e[z]=( 0.689, 0.624, 0.368)

Alignment tensor in laboratory coordinates:

[-2.160e-04,-7.861e-04,-9.713e-04]

[-7.861e-04,-4.682e-04,-1.854e-04]

[-9.713e-04,-1.854e-04,6.842e-04]

SVD condition number is 1.507e+01

Axial component Aa = -2.170e-03

Rhombic component Ar = -1.254e-03

Field=18.79 Teslas[ 2.27]

rhombicity R = 0.578

Asimmetry parameter etha =8.672e-01

GDO = 2.939e-03

ZY'Z'' Euler Angles (degrees)

Set 1

(42.1,68.4,59.1)

Set 2

(-137.9,-68.4,-120.9)

********Data set: #8

Computed data for frame #1

RDC Data:

I J Exp. [Hz] Comp. [Hz]

C5 H16 23.00 25.21

C12 H22 25.20 28.13

C14 H25 10.30 14.80

C14 H24 3.60 -7.99

C26 H37 -19.00 -11.54

C33 H43 -23.00 -15.85

C31 H41 -21.90 -14.32

C29 H39 -23.90 -9.50

C28 H38 -19.50 -16.63

C35 H46 21.30 22.15

C35 H45 -10.20 -13.64

C47 H58 -25.00 -16.17

C54 H64 -19.70 -11.70

C56 H67 0.00 -14.94

C56 H66 2.90 -3.01

C68 H79 -4.20 0.90

C75 H85 -1.20 -2.50

C73 H83 -5.00 -1.81

C71 H81 -5.30 3.06

C70 H80 -4.50 -1.94

C77 H87 -1.30 9.23

C77 H88 4.30 5.22

Cornilescu Quality factor: 0.473418

Alignment tensor information:

A'x=-8.204e-05

A'y=-1.215e-03

A'z= 1.297e-03

Saupe tensor

S'x=-1.231e-04

S'y=-1.823e-03

S'z= 1.946e-03

Alignment tensor eigenvectors

e[x]=( 0.422,-0.767, 0.484)

e[y]=( 0.693, 0.617, 0.373)

e[z]=(-0.584, 0.178, 0.792)

Alignment tensor in laboratory coordinates:

[-1.549e-04,-6.276e-04,-9.312e-04]

[-6.276e-04,-4.698e-04,-6.692e-05]

[-9.312e-04,-6.692e-05,6.248e-04]

SVD condition number is 1.507e+01

Axial component Aa = 1.946e-03

Rhombic component Ar = 1.133e-03

Field=18.79 Teslas[ 2.27]

rhombicity R = 0.582

Asimmetry parameter etha =8.735e-01

GDO = 2.641e-03

ZY'Z'' Euler Angles (degrees)

Set 1

(163.1,37.7,142.4)

Set 2

(-16.9,-37.7,-37.6)

********Data set: #9

Computed data for frame #1

RDC Data:

I J Exp. [Hz] Comp. [Hz]

C5 H16 23.00 18.01

C12 H22 25.20 20.49

C14 H24 10.30 0.91

C14 H25 3.60 -2.90

C26 H37 -19.00 -10.37

C33 H43 -23.00 -14.90

C31 H41 -21.90 -12.94

C29 H39 -23.90 -9.59

C28 H38 -19.50 -17.34

C35 H45 21.30 1.16

C35 H46 -10.20 11.32

C47 H58 -25.00 -8.56

C54 H64 -19.70 -5.44

C56 H66 0.00 -5.22

C56 H67 2.90 -18.96

C68 H79 -4.20 2.84

C75 H85 -1.20 -1.04

C73 H83 -5.00 0.29

C71 H81 -5.30 4.15

C70 H80 -4.50 -1.75

C77 H88 -1.30 7.01

C77 H87 4.30 13.28

Cornilescu Quality factor: 0.724304

Alignment tensor information:

A'x= 4.450e-04

A'y= 1.002e-03

A'z=-1.447e-03

Saupe tensor

S'x= 6.675e-04

S'y= 1.503e-03

S'z=-2.170e-03

Alignment tensor eigenvectors

e[x]=(-0.523, 0.761,-0.385)

e[y]=(-0.497, 0.095, 0.862)

e[z]=( 0.693, 0.642, 0.329)

Alignment tensor in laboratory coordinates:

[-3.248e-04,-8.675e-04,-6.695e-04]

[-8.675e-04,-3.299e-04,-3.537e-04]

[-6.695e-04,-3.537e-04,6.547e-04]

SVD condition number is 1.507e+01

Axial component Aa = -2.170e-03

Rhombic component Ar = -5.568e-04

Field=18.79 Teslas[ 2.27]

rhombicity R = 0.257

Asimmetry parameter etha =3.848e-01

GDO = 2.597e-03

ZY'Z'' Euler Angles (degrees)

Set 1

(42.8,70.8,65.9)

Set 2

(-137.2,-70.8,-114.1)

********Data set: #10

Computed data for frame #1

RDC Data:

I J Exp. [Hz] Comp. [Hz]

C5 H16 23.00 17.79

C12 H22 25.20 19.98

C14 H25 10.30 2.18

C14 H24 3.60 -3.45

C26 H37 -19.00 -11.76

C33 H43 -23.00 -15.66

C31 H41 -21.90 -14.04

C29 H39 -23.90 -10.81

C28 H38 -19.50 -17.42

C35 H45 21.30 -0.79

C35 H46 -10.20 12.68

C47 H58 -25.00 -7.92

C54 H64 -19.70 -4.87

C56 H66 0.00 -3.82

C56 H67 2.90 -18.09

C68 H79 -4.20 2.09

C75 H85 -1.20 -1.17

C73 H83 -5.00 -0.18

C71 H81 -5.30 3.48

C70 H80 -4.50 -1.44

C77 H88 -1.30 7.08

C77 H87 4.30 11.71

Cornilescu Quality factor: 0.722715

Alignment tensor information:

A'x= 2.712e-04

A'y= 9.442e-04

A'z=-1.215e-03

Saupe tensor

S'x= 4.068e-04

S'y= 1.416e-03

S'z=-1.823e-03

Alignment tensor eigenvectors

e[x]=(-0.501, 0.755,-0.424)

e[y]=(-0.518, 0.132, 0.845)

e[z]=( 0.694, 0.643, 0.325)

Alignment tensor in laboratory coordinates:

[-2.638e-04,-7.090e-04,-6.294e-04]

[-7.090e-04,-3.315e-04,-2.353e-04]

[-6.294e-04,-2.353e-04,5.953e-04]

SVD condition number is 1.507e+01

Axial component Aa = -1.823e-03

Rhombic component Ar = -6.730e-04

Field=18.79 Teslas[ 2.27]

rhombicity R = 0.369

Asimmetry parameter etha =5.537e-01

GDO = 2.261e-03

ZY'Z'' Euler Angles (degrees)

Set 1

(42.8,71.1,63.3)

Set 2

(-137.2,-71.1,-116.7)

********Data set: #11

Computed data for frame #1

RDC Data:

I J Exp. [Hz] Comp. [Hz]

C5 H16 23.00 26.01

C12 H22 25.20 28.63

C14 H24 10.30 -4.53

C14 H25 3.60 7.22

C26 H37 -19.00 -10.15

C33 H43 -23.00 -14.37

C31 H41 -21.90 -12.76

C29 H39 -23.90 -8.54

C28 H38 -19.50 -15.63

C35 H46 21.30 22.09

C35 H45 -10.20 -11.80

C47 H58 -25.00 -16.96

C54 H64 -19.70 -13.28

C56 H66 0.00 -3.87

C56 H67 2.90 -15.01

C68 H79 -4.20 0.89

C75 H85 -1.20 -2.34

C73 H83 -5.00 -1.52

C71 H81 -5.30 2.59

C70 H80 -4.50 -2.20

C77 H88 -1.30 1.04

C77 H87 4.30 13.92

Cornilescu Quality factor: 0.512438

Alignment tensor information:

A'x= 7.860e-05

A'y= 1.138e-03

A'z=-1.217e-03

Saupe tensor

S'x= 1.179e-04

S'y= 1.707e-03

S'z=-1.825e-03

Alignment tensor eigenvectors

e[x]=(-0.439, 0.789,-0.430)

e[y]=(-0.554, 0.139, 0.821)

e[z]=( 0.707, 0.598, 0.376)

Alignment tensor in laboratory coordinates:

[-2.438e-04,-6.302e-04,-8.269e-04]

[-6.302e-04,-3.648e-04,-1.707e-04]

[-8.269e-04,-1.707e-04,6.086e-04]

SVD condition number is 1.507e+01

Axial component Aa = -1.825e-03

Rhombic component Ar = -1.060e-03

Field=18.79 Teslas[ 2.27]

rhombicity R = 0.581

Asimmetry parameter etha =8.708e-01

GDO = 2.475e-03

ZY'Z'' Euler Angles (degrees)

Set 1

(40.2,67.9,62.3)

Set 2

(-139.8,-67.9,-117.7)

********Data set: #12

Computed data for frame #1

RDC Data:

I J Exp. [Hz] Comp. [Hz]

C5 H16 23.00 25.79

C12 H22 25.20 28.12

C14 H25 10.30 12.30

C14 H24 3.60 -8.89

C26 H37 -19.00 -11.53

C33 H43 -23.00 -15.13

C31 H41 -21.90 -13.86

C29 H39 -23.90 -9.75

C28 H38 -19.50 -15.71

C35 H46 21.30 23.45

C35 H45 -10.20 -13.75

C47 H58 -25.00 -16.33

C54 H64 -19.70 -12.71

C56 H66 0.00 -2.47

C56 H67 2.90 -14.14

C68 H79 -4.20 0.14

C75 H85 -1.20 -2.46

C73 H83 -5.00 -1.99

C71 H81 -5.30 1.92

C70 H80 -4.50 -1.89

C77 H88 -1.30 1.11

C77 H87 4.30 12.35

Cornilescu Quality factor: 0.470021

Alignment tensor information:

A'x=-9.652e-05

A'y=-9.870e-04

A'z= 1.084e-03

Saupe tensor

S'x=-1.448e-04

S'y=-1.481e-03

S'z= 1.625e-03

Alignment tensor eigenvectors

e[x]=( 0.410,-0.801, 0.435)

e[y]=( 0.718, 0.578, 0.387)

e[z]=(-0.562, 0.154, 0.813)

Alignment tensor in laboratory coordinates:

[-1.827e-04,-4.716e-04,-7.868e-04]

[-4.716e-04,-3.664e-04,-5.222e-05]

[-7.868e-04,-5.222e-05,5.491e-04]

SVD condition number is 1.507e+01

Axial component Aa = 1.625e-03

Rhombic component Ar = 8.905e-04

Field=18.79 Teslas[ 2.27]

rhombicity R = 0.548

Asimmetry parameter etha =8.219e-01

GDO = 2.171e-03

ZY'Z'' Euler Angles (degrees)

Set 1

(164.7,35.6,138.3)

Set 2

(-15.3,-35.6,-41.7)

********Data set: #13

Computed data for frame #1

RDC Data:

I J Exp. [Hz] Comp. [Hz]

C5 H16 23.00 17.96

C12 H22 25.20 20.40

C14 H24 10.30 0.77

C14 H25 3.60 -2.81

C26 H37 -19.00 -10.81

C33 H43 -23.00 -15.34

C31 H41 -21.90 -13.37

C29 H39 -23.90 -10.08

C28 H38 -19.50 -17.83

C35 H45 21.30 1.30

C35 H46 -10.20 11.22

C47 H58 -25.00 -8.60

C54 H64 -19.70 -5.51

C56 H67 0.00 -19.55

C56 H66 2.90 -4.72

C68 H79 -4.20 3.36

C75 H85 -1.20 -0.51

C73 H83 -5.00 0.82

C71 H81 -5.30 4.65

C70 H80 -4.50 -1.24

C77 H88 -1.30 6.86

C77 H87 4.30 13.57

Cornilescu Quality factor: 0.713424

Alignment tensor information:

A'x= 4.508e-04

A'y= 9.963e-04

A'z=-1.447e-03

Saupe tensor

S'x= 6.762e-04

S'y= 1.494e-03

S'z=-2.171e-03

Alignment tensor eigenvectors

e[x]=(-0.534, 0.761,-0.367)

e[y]=(-0.485, 0.079, 0.871)

e[z]=( 0.692, 0.643, 0.327)

Alignment tensor in laboratory coordinates:

[-3.303e-04,-8.662e-04,-6.600e-04]

[-8.662e-04,-3.313e-04,-3.615e-04]

[-6.600e-04,-3.615e-04,6.616e-04]

SVD condition number is 1.507e+01

Axial component Aa = -2.171e-03

Rhombic component Ar = -5.455e-04

Field=18.79 Teslas[ 2.27]

rhombicity R = 0.251

Asimmetry parameter etha =3.770e-01

GDO = 2.594e-03

ZY'Z'' Euler Angles (degrees)

Set 1

(42.9,70.9,67.2)

Set 2

(-137.1,-70.9,-112.8)

********Data set: #14

Computed data for frame #1

RDC Data:

I J Exp. [Hz] Comp. [Hz]

C5 H16 23.00 17.73

C12 H22 25.20 19.88

C14 H25 10.30 2.27

C14 H24 3.60 -3.59

C26 H37 -19.00 -12.19

C33 H43 -23.00 -16.10

C31 H41 -21.90 -14.47

C29 H39 -23.90 -11.29

C28 H38 -19.50 -17.91

C35 H45 21.30 -0.65

C35 H46 -10.20 12.58

C47 H58 -25.00 -7.97

C54 H64 -19.70 -4.95

C56 H67 0.00 -18.68

C56 H66 2.90 -3.31

C68 H79 -4.20 2.61

C75 H85 -1.20 -0.63

C73 H83 -5.00 0.35

C71 H81 -5.30 3.98

C70 H80 -4.50 -0.92

C77 H88 -1.30 6.94

C77 H87 4.30 12.00

Cornilescu Quality factor: 0.7114

Alignment tensor information:

A'x= 2.781e-04

A'y= 9.376e-04

A'z=-1.216e-03

Saupe tensor

S'x= 4.171e-04

S'y= 1.406e-03

S'z=-1.824e-03

Alignment tensor eigenvectors

e[x]=(-0.511, 0.755,-0.411)

e[y]=(-0.508, 0.120, 0.853)

e[z]=( 0.693, 0.645, 0.323)

Alignment tensor in laboratory coordinates:

[-2.693e-04,-7.076e-04,-6.199e-04]

[-7.076e-04,-3.329e-04,-2.431e-04]

[-6.199e-04,-2.431e-04,6.022e-04]

SVD condition number is 1.507e+01

Axial component Aa = -1.824e-03

Rhombic component Ar = -6.595e-04

Field=18.79 Teslas[ 2.27]

rhombicity R = 0.362

Asimmetry parameter etha =5.425e-01

GDO = 2.255e-03

ZY'Z'' Euler Angles (degrees)

Set 1

(42.9,71.2,64.3)

Set 2

(-137.1,-71.2,-115.7)

********Data set: #15

Computed data for frame #1

RDC Data:

I J Exp. [Hz] Comp. [Hz]

C5 H16 23.00 25.96

C12 H22 25.20 28.54

C14 H24 10.30 -4.67

C14 H25 3.60 7.31

C26 H37 -19.00 -10.58

C33 H43 -23.00 -14.81

C31 H41 -21.90 -13.19

C29 H39 -23.90 -9.02

C28 H38 -19.50 -16.12

C35 H46 21.30 21.99

C35 H45 -10.20 -11.66

C47 H58 -25.00 -17.00

C54 H64 -19.70 -13.35

C56 H67 0.00 -15.60

C56 H66 2.90 -3.37

C68 H79 -4.20 1.41

C75 H85 -1.20 -1.80

C73 H83 -5.00 -0.99

C71 H81 -5.30 3.09

C70 H80 -4.50 -1.69

C77 H88 -1.30 0.89

C77 H87 4.30 14.21

Cornilescu Quality factor: 0.499798

Alignment tensor information:

A'x= 8.579e-05

A'y= 1.131e-03

A'z=-1.217e-03

Saupe tensor

S'x= 1.287e-04

S'y= 1.696e-03

S'z=-1.825e-03

Alignment tensor eigenvectors

e[x]=(-0.446, 0.788,-0.423)

e[y]=(-0.549, 0.132, 0.825)

e[z]=( 0.707, 0.601, 0.374)

Alignment tensor in laboratory coordinates:

[-2.492e-04,-6.288e-04,-8.174e-04]

[-6.288e-04,-3.662e-04,-1.785e-04]

[-8.174e-04,-1.785e-04,6.155e-04]

SVD condition number is 1.507e+01

Axial component Aa = -1.825e-03

Rhombic component Ar = -1.045e-03

Field=18.79 Teslas[ 2.27]

rhombicity R = 0.573

Asimmetry parameter etha =8.590e-01

GDO = 2.465e-03

ZY'Z'' Euler Angles (degrees)

Set 1

(40.4,68.1,62.8)

Set 2

(-139.6,-68.1,-117.2)

********Data set: #16

Computed data for frame #1

RDC Data:

I J Exp. [Hz] Comp. [Hz]

C5 H16 23.00 25.73

C12 H22 25.20 28.02

C14 H25 10.30 12.39

C14 H24 3.60 -9.03

C26 H37 -19.00 -11.96

C33 H43 -23.00 -15.57

C31 H41 -21.90 -14.29

C29 H39 -23.90 -10.23

C28 H38 -19.50 -16.20

C35 H46 21.30 23.35

C35 H45 -10.20 -13.61

C47 H58 -25.00 -16.37

C54 H64 -19.70 -12.79

C56 H67 0.00 -14.73

C56 H66 2.90 -1.96

C68 H79 -4.20 0.66

C75 H85 -1.20 -1.93

C73 H83 -5.00 -1.46

C71 H81 -5.30 2.42

C70 H80 -4.50 -1.37

C77 H88 -1.30 0.97

C77 H87 4.30 12.64

Cornilescu Quality factor: 0.455566

Alignment tensor information:

A'x=-8.917e-05

A'y=-9.864e-04

A'z= 1.076e-03

Saupe tensor

S'x=-1.338e-04

S'y=-1.480e-03

S'z= 1.613e-03

Alignment tensor eigenvectors

e[x]=( 0.419,-0.800, 0.430)

e[y]=( 0.717, 0.582, 0.384)

e[z]=(-0.557, 0.148, 0.817)

Alignment tensor in laboratory coordinates:

[-1.882e-04,-4.702e-04,-7.774e-04]

[-4.702e-04,-3.678e-04,-6.001e-05]

[-7.774e-04,-6.001e-05,5.560e-04]

SVD condition number is 1.507e+01

Axial component Aa = 1.613e-03

Rhombic component Ar = 8.973e-04

Field=18.79 Teslas[ 2.27]

rhombicity R = 0.556

Asimmetry parameter etha =8.342e-01

GDO = 2.163e-03

ZY'Z'' Euler Angles (degrees)

Set 1

(165.2,35.2,138.3)

Set 2

(-14.8,-35.2,-41.7)

********

MSpin-RDC pluginmi. jul. 21 14:43:01 2021

gg6

CR_tr

!* MSpin-RDC Plugin *!

********

!* Computation flags *!

Method: SVD

Scaling mode: Hz

Field (T): 18.7923

1H Larmor Frequency: 800.13

Scale QCSA with axial component: False

Include CSA gel shift (isotropic) correction:False

Optimize CSA gel shift (isotropic) correction scale:False

Estimate CSA gel shift (isotropic) correction scale:False

Gel Shift Correction Scale: 0.15

Single Tensor: False

Superimpose: False

Average methyl groups: False

Average methylene groups: False

Average phenyl groups: False

Bootstrapping: False

RDC Std. Error [ppm]: 1

CSA Std. Error [ppm]: 0.01

PCS Std. Error [ppm]: 0.01

DQ Std. Error [Hz]: 1

********

!* Permutations *!

There are no permutations on the original data set

********

Data set: #1

Computed data for frame #1

RDC Data:

I J Exp. [Hz] Comp. [Hz]

C5 H16 23.00 29.46

C12 H22 25.20 29.14

C26 H37 -19.00 -22.17

C33 H43 -23.00 -20.46

C31 H41 -21.90 -22.08

C29 H39 -23.90 -19.18

C28 H38 -19.50 -15.58

C47 H58 -25.00 -18.21

C54 H64 -19.70 -15.73

C68 H79 -4.20 -4.62

C75 H85 -1.20 -1.56

C73 H83 -5.00 -4.06

C71 H81 -5.30 -2.53

C70 H80 -4.50 2.61

Cornilescu Quality factor: 0.225149

Alignment tensor information:

A'x= 5.416e-04

A'y= 1.042e-03

A'z=-1.584e-03

Saupe tensor

S'x= 8.123e-04

S'y= 1.563e-03

S'z=-2.376e-03

Alignment tensor eigenvectors

e[x]=(-0.569, 0.181, 0.802)

e[y]=( 0.661, 0.680, 0.316)

e[z]=(-0.489, 0.710,-0.507)

Alignment tensor in laboratory coordinates:

[ 2.526e-04,9.631e-04,-4.217e-04]

[ 9.631e-04,-2.987e-04,8.723e-04]

[-4.217e-04,8.723e-04,4.605e-05]

SVD condition number is 3.603e+01

Axial component Aa = -2.376e-03

Rhombic component Ar = -5.007e-04

Field=18.79 Teslas[ 2.27]

rhombicity R = 0.211

Asimmetry parameter etha =3.161e-01

GDO = 2.811e-03

ZY'Z'' Euler Angles (degrees)

Set 1

(124.5,120.4,158.5)

Set 2

(-55.5,-120.4,-21.5)

********

MSpin-RDC pluginmi. jul. 21 14:50:34 2021

gg6

E5C12

!* MSpin-RDC Plugin *!

********

!* Computation flags *!

Method: SVD

Scaling mode: Hz

Field (T): 18.7923

1H Larmor Frequency: 800.13

Scale QCSA with axial component: False

Include CSA gel shift (isotropic) correction:False

Optimize CSA gel shift (isotropic) correction scale:False

Estimate CSA gel shift (isotropic) correction scale:False

Gel Shift Correction Scale: 0.15

Single Tensor: False

Superimpose: False

Average methyl groups: False

Average methylene groups: False

Average phenyl groups: False

Bootstrapping: False

RDC Std. Error [ppm]: 1

CSA Std. Error [ppm]: 0.01

PCS Std. Error [ppm]: 0.01

DQ Std. Error [Hz]: 1

********

!* Permutations *!

There are no permutations on the original data set

********

Data set: #1

Computed data for frame #1

RDC Data:

I J Exp. [Hz] Comp. [Hz]

C5 H16 11.80 0.47

C12 H22 12.70 2.23

C26 H37 10.50 5.92

C33 H43 10.60 2.59

C47 H58 9.90 -2.41

C54 H64 9.00 -1.35

C68 H79 4.90 3.71

C75 H85 4.80 0.31

C71 H81 5.30 3.72

C70 H80 8.50 -1.61

C77 H87 0.00 3.96

C77 H88 0.00 3.54

Cornilescu Quality factor: 0.931092

Alignment tensor information:

A'x= 4.224e-04

A'y= 7.867e-04

A'z=-1.209e-03

Saupe tensor

S'x= 6.336e-04

S'y= 1.180e-03

S'z=-1.814e-03

Alignment tensor eigenvectors

e[x]=(-0.611, 0.210, 0.763)

e[y]=( 0.425,-0.726, 0.540)

e[z]=( 0.668, 0.655, 0.355)

Alignment tensor in laboratory coordinates:

[-2.387e-04,-8.255e-04,-3.025e-04]

[-8.255e-04,-8.492e-05,-5.217e-04]

[-3.025e-04,-5.217e-04,3.236e-04]

SVD condition number is 2.221e+01

Axial component Aa = -1.814e-03

Rhombic component Ar = -3.643e-04

Field=18.79 Teslas[ 2.27]

rhombicity R = 0.201

Asimmetry parameter etha =3.013e-01

GDO = 2.141e-03

ZY'Z'' Euler Angles (degrees)

Set 1

(44.4,69.2,144.7)

Set 2

(-135.6,-69.2,-35.3)

********

MSpin-RDC pluginmi. jul. 21 15:53:45 2021

gg6F

CRF_all

!* MSpin-RDC Plugin *!

********

!* Computation flags *!

Method: SVD

Scaling mode: Hz

Field (T): 18.7923

1H Larmor Frequency: 800.13

Scale QCSA with axial component: False

Include CSA gel shift (isotropic) correction:False

Optimize CSA gel shift (isotropic) correction scale:False

Estimate CSA gel shift (isotropic) correction scale:False

Gel Shift Correction Scale: 0.15

Single Tensor: False

Superimpose: False

Average methyl groups: False

Average methylene groups: False

Average phenyl groups: False

Bootstrapping: False

RDC Std. Error [ppm]: 1

CSA Std. Error [ppm]: 0.01

PCS Std. Error [ppm]: 0.01

DQ Std. Error [Hz]: 1

********

!* Permutations *!

There are 4 possible permutations on the original data set

********

Data set: #1

Computed data for frame #1

RDC Data:

I J Exp. [Hz] Comp. [Hz]

C5 H16 18.00 16.19

C10 H20 23.00 16.76

C10 F11 -13.00 4.62

C25 H36 -19.00 -14.72

C32 H42 -23.00 -16.03

C30 H40 -21.90 -15.79

C28 H38 -23.90 -13.71

C27 H37 -19.50 -15.68

C34 H44 21.30 -1.94

C34 H45 -10.20 16.12

C46 H57 -30.00 -3.45

C51 H61 -24.00 -1.64

C51 F52 13.00 -5.76

C66 H77 -4.20 -0.53

C73 H83 -1.20 -1.27

C71 H81 -5.00 -1.45

C69 H79 -5.30 0.59

C68 H78 -4.50 -0.59

C75 H85 -1.30 8.22

C75 H86 4.30 4.69

Cornilescu Quality factor: 0.790036

Alignment tensor information:

A'x=-1.824e-04

A'y=-3.657e-04

A'z= 5.481e-04

Saupe tensor

S'x=-2.737e-04

S'y=-5.485e-04

S'z= 8.222e-04

Alignment tensor eigenvectors

e[x]=( 0.394,-0.802, 0.449)

e[y]=( 0.777, 0.551, 0.304)

e[z]=(-0.491, 0.229, 0.840)

Alignment tensor in laboratory coordinates:

[-1.168e-04,-1.607e-04,-3.448e-04]

[-1.607e-04,-1.997e-04,1.101e-04]

[-3.448e-04,1.101e-04,3.166e-04]

SVD condition number is 1.008e+01

Axial component Aa = 8.222e-04

Rhombic component Ar = 1.832e-04

Field=18.79 Teslas[ 2.27]

rhombicity R = 0.223

Asimmetry parameter etha =3.343e-01

GDO = 9.755e-04

ZY'Z'' Euler Angles (degrees)

Set 1

(155.0,32.8,145.9)

Set 2

(-25.0,-32.8,-34.1)

********Data set: #2

Computed data for frame #1

RDC Data:

I J Exp. [Hz] Comp. [Hz]

C5 H16 18.00 24.20

C10 H20 23.00 24.01

C10 F11 -13.00 11.76

C25 H36 -19.00 -15.81

C32 H42 -23.00 -17.57

C30 H40 -21.90 -17.59

C28 H38 -23.90 -13.42

C27 H37 -19.50 -16.01

C34 H45 21.30 26.41

C34 H44 -10.20 -14.75

C46 H57 -30.00 -14.75

C51 H61 -24.00 -12.55

C51 F52 13.00 -4.47

C66 H77 -4.20 -2.34

C73 H83 -1.20 -3.22

C71 H81 -5.00 -3.84

C69 H79 -5.30 -0.25

C68 H78 -4.50 -1.69

C75 H85 -1.30 6.56

C75 H86 4.30 0.55

Cornilescu Quality factor: 0.542682

Alignment tensor information:

A'x=-3.628e-04

A'y=-6.728e-04

A'z= 1.036e-03

Saupe tensor

S'x=-5.442e-04

S'y=-1.009e-03

S'z= 1.553e-03

Alignment tensor eigenvectors

e[x]=( 0.574, 0.791, 0.210)

e[y]=( 0.560,-0.567, 0.605)

e[z]=( 0.597,-0.230,-0.768)

Alignment tensor in laboratory coordinates:

[ 3.910e-05,-9.354e-05,-7.468e-04]

[-9.354e-05,-3.885e-04,3.530e-04]

[-7.468e-04,3.530e-04,3.494e-04]

SVD condition number is 1.008e+01

Axial component Aa = 1.553e-03

Rhombic component Ar = 3.100e-04

Field=18.79 Teslas[ 2.27]

rhombicity R = 0.200

Asimmetry parameter etha =2.993e-01

GDO = 1.833e-03

ZY'Z'' Euler Angles (degrees)

Set 1

(-21.0,140.2,109.1)

Set 2

(159.0,-140.2,-70.9)

********Data set: #3

Computed data for frame #1

RDC Data:

I J Exp. [Hz] Comp. [Hz]

C5 H16 18.00 16.13

C10 H20 23.00 16.32

C10 F11 -13.00 2.97

C25 H36 -19.00 -15.00

C32 H42 -23.00 -15.79

C30 H40 -21.90 -15.63

C28 H38 -23.90 -14.48

C27 H37 -19.50 -15.64

C34 H44 21.30 -2.43

C34 H45 -10.20 16.07

C46 H57 -30.00 -3.82

C51 H61 -24.00 -2.68

C51 F52 13.00 -6.33

C66 H77 -4.20 -0.82

C73 H83 -1.20 -0.93

C71 H81 -5.00 -1.19

C69 H79 -5.30 -0.22

C68 H78 -4.50 -0.42

C75 H86 -1.30 0.21

C75 H85 4.30 13.55

Cornilescu Quality factor: 0.779401

Alignment tensor information:

A'x=-1.047e-04

A'y=-2.404e-04

A'z= 3.451e-04

Saupe tensor

S'x=-1.570e-04

S'y=-3.607e-04

S'z= 5.177e-04

Alignment tensor eigenvectors

e[x]=( 0.024,-0.986, 0.163)

e[y]=( 0.947, 0.075, 0.313)

e[z]=(-0.321, 0.146, 0.936)

Alignment tensor in laboratory coordinates:

[-1.801e-04,-3.076e-05,-1.753e-04]

[-3.076e-05,-9.579e-05,5.848e-05]

[-1.753e-04,5.848e-05,2.759e-04]

SVD condition number is 1.008e+01

Axial component Aa = 5.177e-04

Rhombic component Ar = 1.358e-04

Field=18.79 Teslas[ 2.27]

rhombicity R = 0.262

Asimmetry parameter etha =3.934e-01

GDO = 6.205e-04

ZY'Z'' Euler Angles (degrees)

Set 1

(155.5,20.7,117.5)

Set 2

(-24.5,-20.7,-62.5)

********Data set: #4

Computed data for frame #1

RDC Data:

I J Exp. [Hz] Comp. [Hz]

C5 H16 18.00 24.14

C10 H20 23.00 23.56

C10 F11 -13.00 10.11

C25 H36 -19.00 -16.10

C32 H42 -23.00 -17.34

C30 H40 -21.90 -17.43

C28 H38 -23.90 -14.18

C27 H37 -19.50 -15.98

C34 H45 21.30 26.37

C34 H44 -10.20 -15.23

C46 H57 -30.00 -15.12

C51 H61 -24.00 -13.59

C51 F52 13.00 -5.04

C66 H77 -4.20 -2.63

C73 H83 -1.20 -2.88

C71 H81 -5.00 -3.59

C69 H79 -5.30 -1.06

C68 H78 -4.50 -1.52

C75 H86 -1.30 -3.94

C75 H85 4.30 11.90

Cornilescu Quality factor: 0.522379

Alignment tensor information:

A'x=-1.786e-04

A'y=-6.107e-04

A'z= 7.892e-04

Saupe tensor

S'x=-2.678e-04

S'y=-9.160e-04

S'z= 1.184e-03

Alignment tensor eigenvectors

e[x]=( 0.584, 0.785, 0.206)

e[y]=(-0.588, 0.584,-0.560)

e[z]=(-0.560, 0.206, 0.802)

Alignment tensor in laboratory coordinates:

[-2.412e-05,3.644e-05,-5.773e-04]

[ 3.644e-05,-2.846e-04,3.014e-04]

[-5.773e-04,3.014e-04,3.087e-04]

SVD condition number is 1.008e+01

Axial component Aa = 1.184e-03

Rhombic component Ar = 4.321e-04

Field=18.79 Teslas[ 2.27]

rhombicity R = 0.365

Asimmetry parameter etha =5.475e-01

GDO = 1.466e-03

ZY'Z'' Euler Angles (degrees)

Set 1

(159.8,36.7,-110.2)

Set 2

(-20.2,-36.7,69.8)

********

MSpin-RDC pluginju. jul. 22 16:37:10 2021

gg6F

CRF_tr

!* MSpin-RDC Plugin *!

********

!* Computation flags *!

Method: SVD

Scaling mode: Hz

Field (T): 18.7923

1H Larmor Frequency: 800.13

Scale QCSA with axial component: False

Include CSA gel shift (isotropic) correction:False

Optimize CSA gel shift (isotropic) correction scale:False

Estimate CSA gel shift (isotropic) correction scale:False

Gel Shift Correction Scale: 0.15

Single Tensor: False

Superimpose: False

Average methyl groups: False

Average methylene groups: False

Average phenyl groups: False

Bootstrapping: False

RDC Std. Error [ppm]: 1

CSA Std. Error [ppm]: 0.01

PCS Std. Error [ppm]: 0.01

DQ Std. Error [Hz]: 1

********

!* Permutations *!

There are no permutations on the original data set

********

Data set: #1

Computed data for frame #1

RDC Data:

I J Exp. [Hz] Comp. [Hz]

C5 H16 18.00 26.45

C10 H20 23.00 22.30

C10 F11 -13.00 -2.82

C25 H36 -19.00 -19.33

C32 H42 -23.00 -16.14

C30 H40 -21.90 -16.97

C28 H38 -23.90 -21.33

C27 H37 -19.50 -16.26

C46 H57 -30.00 -21.41

C51 H61 -24.00 -25.64

C51 F52 13.00 -9.97

C66 H77 -4.20 -5.84

C73 H83 -1.20 -0.54

C71 H81 -5.00 -2.12

C69 H79 -5.30 -8.56

C68 H78 -4.50 -0.45

Cornilescu Quality factor: 0.422449

Alignment tensor information:

A'x= 2.148e-04

A'y= 1.443e-03

A'z=-1.657e-03

Saupe tensor

S'x= 3.222e-04

S'y= 2.164e-03

S'z=-2.486e-03

Alignment tensor eigenvectors

e[x]=(-0.210, 0.453,-0.866)

e[y]=( 0.573, 0.775, 0.266)

e[z]=( 0.792,-0.441,-0.423)

Alignment tensor in laboratory coordinates:

[-5.558e-04,1.199e-03,8.141e-04]

[ 1.199e-03,5.881e-04,-9.531e-05]

[ 8.141e-04,-9.531e-05,-3.235e-05]

SVD condition number is 2.227e+01

Axial component Aa = -2.486e-03

Rhombic component Ar = -1.228e-03

Field=18.79 Teslas[ 2.27]

rhombicity R = 0.494

Asimmetry parameter etha =7.408e-01

GDO = 3.241e-03

ZY'Z'' Euler Angles (degrees)

Set 1

(-29.1,115.0,17.1)

Set 2

(150.9,-115.0,-162.9)

********

MSpin-RDC pluginmi. jul. 21 15:07:52 2021

gt6

CR_all

!* MSpin-RDC Plugin *!

********

!* Computation flags *!

Method: SVD

Scaling mode: Hz

Field (T): 18.7923

1H Larmor Frequency: 800.13

Scale QCSA with axial component: False

Include CSA gel shift (isotropic) correction:False

Optimize CSA gel shift (isotropic) correction scale:False

Estimate CSA gel shift (isotropic) correction scale:False

Gel Shift Correction Scale: 0.15

Single Tensor: False

Superimpose: False

Average methyl groups: False

Average methylene groups: False

Average phenyl groups: False

Bootstrapping: False

RDC Std. Error [ppm]: 1

CSA Std. Error [ppm]: 0.01

PCS Std. Error [ppm]: 0.01

DQ Std. Error [Hz]: 1

********

!* Permutations *!

There are 16 possible permutations on the original data set

********

Data set: #1

Computed data for frame #1

RDC Data:

I J Exp. [Hz] Comp. [Hz]

C5 H16 23.00 2.24

C12 H22 25.20 1.37

C14 H24 10.30 -0.38

C14 H25 3.60 5.11

C26 H37 -19.00 -4.29

C33 H43 -23.00 0.57

C31 H41 -21.90 -0.82

C29 H39 -23.90 -4.52

C28 H38 -19.50 0.27

C35 H45 21.30 1.73

C35 H46 -10.20 -4.42

C47 H58 -25.00 -9.64

C54 H64 -19.70 -11.08

C56 H66 0.00 -12.67

C56 H67 2.90 1.76

C68 H79 -4.20 -12.79

C75 H85 -1.20 -7.84

C73 H83 -5.00 -9.30

C71 H81 -5.30 -12.72

C70 H80 -4.50 -7.80

C77 H87 -1.30 -6.15

C77 H88 4.30 -1.55

Cornilescu Quality factor: 0.895471

Alignment tensor information:

A'x=-3.008e-04

A'y=-1.221e-03

A'z= 1.522e-03

Saupe tensor

S'x=-4.513e-04

S'y=-1.831e-03

S'z= 2.282e-03

Alignment tensor eigenvectors

e[x]=( 0.742,-0.444,-0.503)

e[y]=( 0.634, 0.708, 0.311)

e[z]=( 0.217,-0.550, 0.807)

Alignment tensor in laboratory coordinates:

[-5.848e-04,-6.308e-04,1.382e-04]

[-6.308e-04,-2.111e-04,-1.010e-03]

[ 1.382e-04,-1.010e-03,7.959e-04]

SVD condition number is 2.818e+01

Axial component Aa = 2.282e-03

Rhombic component Ar = 9.198e-04

Field=18.79 Teslas[ 2.27]

rhombicity R = 0.403

Asimmetry parameter etha =6.045e-01

GDO = 2.866e-03

ZY'Z'' Euler Angles (degrees)

Set 1

(-68.4,36.2,31.8)

Set 2

(111.6,-36.2,-148.2)

********Data set: #2

Computed data for frame #1

RDC Data:

I J Exp. [Hz] Comp. [Hz]

C5 H16 23.00 1.47

C12 H22 25.20 0.10

C14 H25 10.30 8.42

C14 H24 3.60 -1.83

C26 H37 -19.00 -5.03

C33 H43 -23.00 0.01

C31 H41 -21.90 -1.67

C29 H39 -23.90 -5.07

C28 H38 -19.50 0.18

C35 H45 21.30 1.39

C35 H46 -10.20 -4.97

C47 H58 -25.00 -9.99

C54 H64 -19.70 -11.97

C56 H66 0.00 -13.83

C56 H67 2.90 5.03

C68 H79 -4.20 -12.70

C75 H85 -1.20 -7.48

C73 H83 -5.00 -9.26

C71 H81 -5.30 -12.41

C70 H80 -4.50 -6.99

C77 H87 -1.30 -5.56

C77 H88 4.30 -1.14

Cornilescu Quality factor: 0.884283

Alignment tensor information:

A'x=-4.897e-04

A'y=-1.091e-03

A'z= 1.581e-03

Saupe tensor

S'x=-7.345e-04

S'y=-1.637e-03

S'z= 2.371e-03

Alignment tensor eigenvectors

e[x]=( 0.756,-0.414,-0.507)

e[y]=( 0.615, 0.715, 0.332)

e[z]=( 0.225,-0.563, 0.795)

Alignment tensor in laboratory coordinates:

[-6.119e-04,-5.272e-04,2.485e-04]

[-5.272e-04,-1.417e-04,-1.069e-03]

[ 2.485e-04,-1.069e-03,7.536e-04]

SVD condition number is 2.818e+01

Axial component Aa = 2.371e-03

Rhombic component Ar = 6.014e-04

Field=18.79 Teslas[ 2.27]

rhombicity R = 0.254

Asimmetry parameter etha =3.805e-01

GDO = 2.835e-03

ZY'Z'' Euler Angles (degrees)

Set 1

(-68.2,37.3,33.2)

Set 2

(111.8,-37.3,-146.8)

********Data set: #3

Computed data for frame #1

RDC Data:

I J Exp. [Hz] Comp. [Hz]

C5 H16 23.00 -3.17

C12 H22 25.20 -1.72

C14 H24 10.30 -1.61

C14 H25 3.60 2.86

C26 H37 -19.00 -5.39

C33 H43 -23.00 -4.47

C31 H41 -21.90 -3.88

C29 H39 -23.90 -6.21

C28 H38 -19.50 -6.18

C35 H46 21.30 13.07

C35 H45 -10.20 -4.86

C47 H58 -25.00 -8.63

C54 H64 -19.70 -7.07

C56 H66 0.00 -6.57

C56 H67 2.90 2.68

C68 H79 -4.20 -11.32

C75 H85 -1.20 -10.56

C73 H83 -5.00 -9.91

C71 H81 -5.30 -12.08

C70 H80 -4.50 -12.16

C77 H87 -1.30 -10.81

C77 H88 4.30 11.76

Cornilescu Quality factor: 0.85543

Alignment tensor information:

A'x= 8.773e-05

A'y= 5.360e-04

A'z=-6.238e-04

Saupe tensor

S'x= 1.316e-04

S'y= 8.041e-04

S'z=-9.357e-04

Alignment tensor eigenvectors

e[x]=( 0.048, 0.293,-0.955)

e[y]=(-0.699, 0.693, 0.177)

e[z]=( 0.713, 0.659, 0.238)

Alignment tensor in laboratory coordinates:

[-5.524e-05,-5.517e-04,-1.762e-04]

[-5.517e-04,-6.275e-06,-5.659e-05]

[-1.762e-04,-5.659e-05,6.152e-05]

SVD condition number is 2.818e+01

Axial component Aa = -9.357e-04

Rhombic component Ar = -4.483e-04

Field=18.79 Teslas[ 2.27]

rhombicity R = 0.479

Asimmetry parameter etha =7.187e-01

GDO = 1.212e-03

ZY'Z'' Euler Angles (degrees)

Set 1

(42.7,76.2,10.5)

Set 2

(-137.3,-76.2,-169.5)

********Data set: #4

Computed data for frame #1

RDC Data:

I J Exp. [Hz] Comp. [Hz]

C5 H16 23.00 -3.94

C12 H22 25.20 -2.98

C14 H25 10.30 6.17

C14 H24 3.60 -3.06

C26 H37 -19.00 -6.13

C33 H43 -23.00 -5.03

C31 H41 -21.90 -4.73

C29 H39 -23.90 -6.75

C28 H38 -19.50 -6.27

C35 H46 21.30 12.52

C35 H45 -10.20 -5.19

C47 H58 -25.00 -8.97

C54 H64 -19.70 -7.96

C56 H66 0.00 -7.73

C56 H67 2.90 5.94

C68 H79 -4.20 -11.22

C75 H85 -1.20 -10.20

C73 H83 -5.00 -9.87

C71 H81 -5.30 -11.78

C70 H80 -4.50 -11.35

C77 H87 -1.30 -10.22

C77 H88 4.30 12.16

Cornilescu Quality factor: 0.845238

Alignment tensor information:

A'x= 4.488e-05

A'y= 4.494e-04

A'z=-4.942e-04

Saupe tensor

S'x= 6.733e-05

S'y= 6.740e-04

S'z=-7.414e-04

Alignment tensor eigenvectors

e[x]=( 0.252, 0.071,-0.965)

e[y]=( 0.633,-0.767, 0.109)

e[z]=( 0.732, 0.638, 0.238)

Alignment tensor in laboratory coordinates:

[-8.227e-05,-4.481e-04,-6.598e-05]

[-4.481e-04,6.307e-05,-1.156e-04]

[-6.598e-05,-1.156e-04,1.921e-05]

SVD condition number is 2.818e+01

Axial component Aa = -7.414e-04

Rhombic component Ar = -4.045e-04

Field=18.79 Teslas[ 2.27]

rhombicity R = 0.546

Asimmetry parameter etha =8.184e-01

GDO = 9.891e-04

ZY'Z'' Euler Angles (degrees)

Set 1

(41.1,76.2,6.4)

Set 2

(-138.9,-76.2,-173.6)

********Data set: #5

Computed data for frame #1

RDC Data:

I J Exp. [Hz] Comp. [Hz]

C5 H16 23.00 2.19

C12 H22 25.20 1.58

C14 H24 10.30 0.01

C14 H25 3.60 3.58

C26 H37 -19.00 -4.02

C33 H43 -23.00 0.51

C31 H41 -21.90 -0.67

C29 H39 -23.90 -4.33

C28 H38 -19.50 0.03

C35 H45 21.30 1.51

C35 H46 -10.20 -4.16

C47 H58 -25.00 -9.27

C54 H64 -19.70 -10.40

C56 H67 0.00 0.40

C56 H66 2.90 -11.79

C68 H79 -4.20 -12.45

C75 H85 -1.20 -7.86

C73 H83 -5.00 -9.11

C71 H81 -5.30 -12.47

C70 H80 -4.50 -8.02

C77 H87 -1.30 -6.37

C77 H88 4.30 -1.86

Cornilescu Quality factor: 0.903572

Alignment tensor information:

A'x=-2.032e-04

A'y=-1.219e-03

A'z= 1.422e-03

Saupe tensor

S'x=-3.048e-04

S'y=-1.828e-03

S'z= 2.133e-03

Alignment tensor eigenvectors

e[x]=( 0.743,-0.451,-0.495)

e[y]=( 0.635, 0.709, 0.307)

e[z]=( 0.212,-0.542, 0.813)

Alignment tensor in laboratory coordinates:

[-5.394e-04,-6.444e-04,8.289e-05]

[-6.444e-04,-2.359e-04,-9.372e-04]

[ 8.289e-05,-9.372e-04,7.753e-04]

SVD condition number is 2.818e+01

Axial component Aa = 2.133e-03

Rhombic component Ar = 1.016e-03

Field=18.79 Teslas[ 2.27]

rhombicity R = 0.476

Asimmetry parameter etha =7.142e-01

GDO = 2.759e-03

ZY'Z'' Euler Angles (degrees)

Set 1

(-68.6,35.6,31.8)

Set 2

(111.4,-35.6,-148.2)

********Data set: #6

Computed data for frame #1

RDC Data:

I J Exp. [Hz] Comp. [Hz]

C5 H16 23.00 1.42

C12 H22 25.20 0.31

C14 H25 10.30 6.89

C14 H24 3.60 -1.45

C26 H37 -19.00 -4.76

C33 H43 -23.00 -0.05

C31 H41 -21.90 -1.53

C29 H39 -23.90 -4.87

C28 H38 -19.50 -0.07

C35 H45 21.30 1.18

C35 H46 -10.20 -4.71

C47 H58 -25.00 -9.62

C54 H64 -19.70 -11.29

C56 H67 0.00 3.66

C56 H66 2.90 -12.95

C68 H79 -4.20 -12.35

C75 H85 -1.20 -7.51

C73 H83 -5.00 -9.08

C71 H81 -5.30 -12.16

C70 H80 -4.50 -7.21

C77 H87 -1.30 -5.78

C77 H88 4.30 -1.46

Cornilescu Quality factor: 0.8952

Alignment tensor information:

A'x=-3.916e-04

A'y=-1.089e-03

A'z= 1.480e-03

Saupe tensor

S'x=-5.874e-04

S'y=-1.633e-03

S'z= 2.221e-03

Alignment tensor eigenvectors

e[x]=( 0.753,-0.423,-0.504)

e[y]=( 0.620, 0.714, 0.325)

e[z]=( 0.223,-0.557, 0.800)

Alignment tensor in laboratory coordinates:

[-5.664e-04,-5.409e-04,1.932e-04]

[-5.409e-04,-1.666e-04,-9.963e-04]

[ 1.932e-04,-9.963e-04,7.330e-04]

SVD condition number is 2.818e+01

Axial component Aa = 2.221e-03

Rhombic component Ar = 6.972e-04

Field=18.79 Teslas[ 2.27]

rhombicity R = 0.314

Asimmetry parameter etha =4.709e-01

GDO = 2.703e-03

ZY'Z'' Euler Angles (degrees)

Set 1

(-68.2,36.9,32.8)

Set 2

(111.8,-36.9,-147.2)

********Data set: #7

Computed data for frame #1

RDC Data:

I J Exp. [Hz] Comp. [Hz]

C5 H16 23.00 -3.22

C12 H22 25.20 -1.51

C14 H24 10.30 -1.22

C14 H25 3.60 1.33

C26 H37 -19.00 -5.11

C33 H43 -23.00 -4.53

C31 H41 -21.90 -3.74

C29 H39 -23.90 -6.01

C28 H38 -19.50 -6.43

C35 H46 21.30 13.34

C35 H45 -10.20 -5.08

C47 H58 -25.00 -8.26

C54 H64 -19.70 -6.39

C56 H67 0.00 1.31

C56 H66 2.90 -5.69

C68 H79 -4.20 -10.98

C75 H85 -1.20 -10.58

C73 H83 -5.00 -9.72

C71 H81 -5.30 -11.84

C70 H80 -4.50 -12.38

C77 H87 -1.30 -11.02

C77 H88 4.30 11.44

Cornilescu Quality factor: 0.860615

Alignment tensor information:

A'x= 1.888e-05

A'y= 6.026e-04

A'z=-6.215e-04

Saupe tensor

S'x= 2.832e-05

S'y= 9.040e-04

S'z=-9.323e-04

Alignment tensor eigenvectors

e[x]=(-0.061, 0.381,-0.923)

e[y]=(-0.706, 0.637, 0.310)

e[z]=( 0.706, 0.670, 0.230)

Alignment tensor in laboratory coordinates:

[-9.783e-06,-5.654e-04,-2.316e-04]

[-5.654e-04,-3.112e-05,1.650e-05]

[-2.316e-04,1.650e-05,4.090e-05]

SVD condition number is 2.818e+01

Axial component Aa = -9.323e-04

Rhombic component Ar = -5.838e-04

Field=18.79 Teslas[ 2.27]

rhombicity R = 0.626

Asimmetry parameter etha =9.393e-01

GDO = 1.292e-03

ZY'Z'' Euler Angles (degrees)

Set 1

(43.5,76.7,18.5)

Set 2

(-136.5,-76.7,-161.5)

********Data set: #8

Computed data for frame #1

RDC Data:

I J Exp. [Hz] Comp. [Hz]

C5 H16 23.00 -3.99

C12 H22 25.20 -2.77

C14 H25 10.30 4.64

C14 H24 3.60 -2.68

C26 H37 -19.00 -5.86

C33 H43 -23.00 -5.09

C31 H41 -21.90 -4.59

C29 H39 -23.90 -6.56

C28 H38 -19.50 -6.52

C35 H46 21.30 12.78

C35 H45 -10.20 -5.41

C47 H58 -25.00 -8.60

C54 H64 -19.70 -7.28

C56 H67 0.00 4.58

C56 H66 2.90 -6.85

C68 H79 -4.20 -10.88

C75 H85 -1.20 -10.23

C73 H83 -5.00 -9.68

C71 H81 -5.30 -11.53

C70 H80 -4.50 -11.57

C77 H87 -1.30 -10.43

C77 H88 4.30 11.85

Cornilescu Quality factor: 0.853332

Alignment tensor information:

A'x= 2.148e-05

A'y= 4.697e-04

A'z=-4.912e-04

Saupe tensor

S'x= 3.222e-05

S'y= 7.046e-04

S'z=-7.368e-04

Alignment tensor eigenvectors

e[x]=( 0.098, 0.241,-0.966)

e[y]=(-0.684, 0.721, 0.111)

e[z]=( 0.723, 0.650, 0.235)

Alignment tensor in laboratory coordinates:

[-3.682e-05,-4.618e-04,-1.213e-04]

[-4.618e-04,3.823e-05,-4.252e-05]

[-1.213e-04,-4.252e-05,-1.410e-06]

SVD condition number is 2.818e+01

Axial component Aa = -7.368e-04

Rhombic component Ar = -4.483e-04

Field=18.79 Teslas[ 2.27]

rhombicity R = 0.608

Asimmetry parameter etha =9.125e-01

GDO = 1.013e-03

ZY'Z'' Euler Angles (degrees)

Set 1

(41.9,76.4,6.6)

Set 2

(-138.1,-76.4,-173.4)

********Data set: #9

Computed data for frame #1

RDC Data:

I J Exp. [Hz] Comp. [Hz]

C5 H16 23.00 2.31

C12 H22 25.20 1.24

C14 H24 10.30 -0.57

C14 H25 3.60 5.07

C26 H37 -19.00 -4.16

C33 H43 -23.00 0.74

C31 H41 -21.90 -0.76

C29 H39 -23.90 -4.31

C28 H38 -19.50 0.64

C35 H45 21.30 1.98

C35 H46 -10.20 -7.36

C47 H58 -25.00 -9.30

C54 H64 -19.70 -10.97

C56 H66 0.00 -12.67

C56 H67 2.90 1.55

C68 H79 -4.20 -12.21

C75 H85 -1.20 -7.19

C73 H83 -5.00 -8.78

C71 H81 -5.30 -12.04

C70 H80 -4.50 -6.95

C77 H88 -1.30 -4.30

C77 H87 4.30 -5.42

Cornilescu Quality factor: 0.898846

Alignment tensor information:

A'x=-3.612e-04

A'y=-1.206e-03

A'z= 1.568e-03

Saupe tensor

S'x=-5.418e-04

S'y=-1.810e-03

S'z= 2.351e-03

Alignment tensor eigenvectors

e[x]=( 0.769,-0.426,-0.477)

e[y]=( 0.605, 0.725, 0.328)

e[z]=( 0.206,-0.541, 0.815)

Alignment tensor in laboratory coordinates:

[-5.888e-04,-5.858e-04,1.560e-04]

[-5.858e-04,-2.415e-04,-1.052e-03]

[ 1.560e-04,-1.052e-03,8.303e-04]

SVD condition number is 2.818e+01

Axial component Aa = 2.351e-03

Rhombic component Ar = 8.452e-04

Field=18.79 Teslas[ 2.27]

rhombicity R = 0.359

Asimmetry parameter etha =5.392e-01

GDO = 2.906e-03

ZY'Z'' Euler Angles (degrees)

Set 1

(-69.2,35.4,34.6)

Set 2

(110.8,-35.4,-145.4)

********Data set: #10

Computed data for frame #1

RDC Data:

I J Exp. [Hz] Comp. [Hz]

C5 H16 23.00 1.54

C12 H22 25.20 -0.03

C14 H25 10.30 8.38

C14 H24 3.60 -2.03

C26 H37 -19.00 -4.91

C33 H43 -23.00 0.18

C31 H41 -21.90 -1.61

C29 H39 -23.90 -4.85

C28 H38 -19.50 0.54

C35 H45 21.30 1.64

C35 H46 -10.20 -7.91

C47 H58 -25.00 -9.64

C54 H64 -19.70 -11.86

C56 H66 0.00 -13.84

C56 H67 2.90 4.82

C68 H79 -4.20 -12.11

C75 H85 -1.20 -6.84

C73 H83 -5.00 -8.75

C71 H81 -5.30 -11.73

C70 H80 -4.50 -6.14

C77 H88 -1.30 -3.90

C77 H87 4.30 -4.84

Cornilescu Quality factor: 0.887484

Alignment tensor information:

A'x=-5.460e-04

A'y=-1.080e-03

A'z= 1.626e-03

Saupe tensor

S'x=-8.190e-04

S'y=-1.619e-03

S'z= 2.438e-03

Alignment tensor eigenvectors

e[x]=( 0.797,-0.376,-0.473)

e[y]=( 0.565, 0.742, 0.361)

e[z]=( 0.215,-0.555, 0.804)

Alignment tensor in laboratory coordinates:

[-6.159e-04,-4.822e-04,2.663e-04]

[-4.822e-04,-1.721e-04,-1.111e-03]

[ 2.663e-04,-1.111e-03,7.880e-04]

SVD condition number is 2.818e+01

Axial component Aa = 2.438e-03

Rhombic component Ar = 5.335e-04

Field=18.79 Teslas[ 2.27]

rhombicity R = 0.219

Asimmetry parameter etha =3.282e-01

GDO = 2.890e-03

ZY'Z'' Euler Angles (degrees)

Set 1

(-68.8,36.5,37.4)

Set 2

(111.2,-36.5,-142.6)

********Data set: #11

Computed data for frame #1

RDC Data:

I J Exp. [Hz] Comp. [Hz]

C5 H16 23.00 -3.10

C12 H22 25.20 -1.84

C14 H24 10.30 -1.80

C14 H25 3.60 2.82

C26 H37 -19.00 -5.26

C33 H43 -23.00 -4.30

C31 H41 -21.90 -3.82

C29 H39 -23.90 -5.99

C28 H38 -19.50 -5.82

C35 H46 21.30 10.13

C35 H45 -10.20 -4.61

C47 H58 -25.00 -8.28

C54 H64 -19.70 -6.96

C56 H66 0.00 -6.57

C56 H67 2.90 2.47

C68 H79 -4.20 -10.74

C75 H85 -1.20 -9.91

C73 H83 -5.00 -9.39

C71 H81 -5.30 -11.40

C70 H80 -4.50 -11.31

C77 H88 -1.30 9.00

C77 H87 4.30 -10.08

Cornilescu Quality factor: 0.880794

Alignment tensor information:

A'x= 1.386e-04

A'y= 4.638e-04

A'z=-6.024e-04

Saupe tensor

S'x= 2.079e-04

S'y= 6.957e-04

S'z=-9.036e-04

Alignment tensor eigenvectors

e[x]=( 0.095, 0.263,-0.960)

e[y]=(-0.709, 0.695, 0.120)

e[z]=( 0.698, 0.670, 0.253)

Alignment tensor in laboratory coordinates:

[-5.922e-05,-5.067e-04,-1.584e-04]

[-5.067e-04,-3.666e-05,-9.838e-05]

[-1.584e-04,-9.838e-05,9.588e-05]

SVD condition number is 2.818e+01

Axial component Aa = -9.036e-04

Rhombic component Ar = -3.252e-04

Field=18.79 Teslas[ 2.27]

rhombicity R = 0.360

Asimmetry parameter etha =5.399e-01

GDO = 1.117e-03

ZY'Z'' Euler Angles (degrees)

Set 1

(43.8,75.4,7.1)

Set 2

(-136.2,-75.4,-172.9)

********Data set: #12

Computed data for frame #1

RDC Data:

I J Exp. [Hz] Comp. [Hz]

C5 H16 23.00 -3.87

C12 H22 25.20 -3.11

C14 H25 10.30 6.13

C14 H24 3.60 -3.25

C26 H37 -19.00 -6.01

C33 H43 -23.00 -4.86

C31 H41 -21.90 -4.67

C29 H39 -23.90 -6.54

C28 H38 -19.50 -5.91

C35 H46 21.30 9.58

C35 H45 -10.20 -4.94

C47 H58 -25.00 -8.62

C54 H64 -19.70 -7.85

C56 H66 0.00 -7.73

C56 H67 2.90 5.74

C68 H79 -4.20 -10.64

C75 H85 -1.20 -9.56

C73 H83 -5.00 -9.36

C71 H81 -5.30 -11.10

C70 H80 -4.50 -10.51

C77 H88 -1.30 9.41

C77 H87 4.30 -9.49

Cornilescu Quality factor: 0.870678

Alignment tensor information:

A'x= 6.770e-05

A'y= 4.042e-04

A'z=-4.719e-04

Saupe tensor

S'x= 1.015e-04

S'y= 6.063e-04

S'z=-7.079e-04

Alignment tensor eigenvectors

e[x]=(-0.366, 0.028, 0.930)

e[y]=( 0.598,-0.759, 0.258)

e[z]=( 0.713, 0.651, 0.260)

Alignment tensor in laboratory coordinates:

[-8.625e-05,-4.032e-04,-4.818e-05]

[-4.032e-04,3.268e-05,-1.574e-04]

[-4.818e-05,-1.574e-04,5.357e-05]

SVD condition number is 2.818e+01

Axial component Aa = -7.079e-04

Rhombic component Ar = -3.365e-04

Field=18.79 Teslas[ 2.27]

rhombicity R = 0.475

Asimmetry parameter etha =7.131e-01

GDO = 9.154e-04

ZY'Z'' Euler Angles (degrees)

Set 1

(42.4,74.9,164.5)

Set 2

(-137.6,-74.9,-15.5)

********Data set: #13

Computed data for frame #1

RDC Data:

I J Exp. [Hz] Comp. [Hz]

C5 H16 23.00 2.26

C12 H22 25.20 1.45

C14 H24 10.30 -0.19

C14 H25 3.60 3.53

C26 H37 -19.00 -3.89

C33 H43 -23.00 0.68

C31 H41 -21.90 -0.62

C29 H39 -23.90 -4.11

C28 H38 -19.50 0.39

C35 H45 21.30 1.76

C35 H46 -10.20 -7.10

C47 H58 -25.00 -8.93

C54 H64 -19.70 -10.29

C56 H67 0.00 0.19

C56 H66 2.90 -11.79

C68 H79 -4.20 -11.87

C75 H85 -1.20 -7.22

C73 H83 -5.00 -8.59

C71 H81 -5.30 -11.79

C70 H80 -4.50 -7.17

C77 H88 -1.30 -4.62

C77 H87 4.30 -5.64

Cornilescu Quality factor: 0.906795

Alignment tensor information:

A'x=-2.645e-04

A'y=-1.204e-03

A'z= 1.469e-03

Saupe tensor

S'x=-3.968e-04

S'y=-1.806e-03

S'z= 2.203e-03

Alignment tensor eigenvectors

e[x]=( 0.768,-0.436,-0.469)

e[y]=( 0.609, 0.725, 0.322)

e[z]=( 0.200,-0.533, 0.822)

Alignment tensor in laboratory coordinates:

[-5.434e-04,-5.995e-04,1.007e-04]

[-5.995e-04,-2.663e-04,-9.790e-04]

[ 1.007e-04,-9.790e-04,8.097e-04]

SVD condition number is 2.818e+01

Axial component Aa = 2.203e-03

Rhombic component Ar = 9.398e-04

Field=18.79 Teslas[ 2.27]

rhombicity R = 0.427

Asimmetry parameter etha =6.398e-01

GDO = 2.792e-03

ZY'Z'' Euler Angles (degrees)

Set 1

(-69.4,34.7,34.5)

Set 2

(110.6,-34.7,-145.5)

********Data set: #14

Computed data for frame #1

RDC Data:

I J Exp. [Hz] Comp. [Hz]

C5 H16 23.00 1.49

C12 H22 25.20 0.18

C14 H25 10.30 6.84

C14 H24 3.60 -1.64

C26 H37 -19.00 -4.64

C33 H43 -23.00 0.12

C31 H41 -21.90 -1.47

C29 H39 -23.90 -4.66

C28 H38 -19.50 0.30

C35 H45 21.30 1.43

C35 H46 -10.20 -7.65

C47 H58 -25.00 -9.27

C54 H64 -19.70 -11.18

C56 H67 0.00 3.46

C56 H66 2.90 -12.96

C68 H79 -4.20 -11.77

C75 H85 -1.20 -6.86

C73 H83 -5.00 -8.56

C71 H81 -5.30 -11.48

C70 H80 -4.50 -6.36

C77 H88 -1.30 -4.21

C77 H87 4.30 -5.05

Cornilescu Quality factor: 0.898239

Alignment tensor information:

A'x=-4.493e-04

A'y=-1.077e-03

A'z= 1.526e-03

Saupe tensor

S'x=-6.740e-04

S'y=-1.615e-03

S'z= 2.289e-03

Alignment tensor eigenvectors

e[x]=( 0.789,-0.393,-0.472)

e[y]=( 0.577, 0.738, 0.349)

e[z]=( 0.211,-0.548, 0.809)

Alignment tensor in laboratory coordinates:

[-5.704e-04,-4.959e-04,2.109e-04]

[-4.959e-04,-1.970e-04,-1.038e-03]

[ 2.109e-04,-1.038e-03,7.674e-04]

SVD condition number is 2.818e+01

Axial component Aa = 2.289e-03

Rhombic component Ar = 6.272e-04

Field=18.79 Teslas[ 2.27]

rhombicity R = 0.274

Asimmetry parameter etha =4.110e-01

GDO = 2.752e-03

ZY'Z'' Euler Angles (degrees)

Set 1

(-68.9,36.0,36.5)

Set 2

(111.1,-36.0,-143.5)

********Data set: #15

Computed data for frame #1

RDC Data:

I J Exp. [Hz] Comp. [Hz]

C5 H16 23.00 -3.15

C12 H22 25.20 -1.63

C14 H24 10.30 -1.42

C14 H25 3.60 1.28

C26 H37 -19.00 -4.99

C33 H43 -23.00 -4.36

C31 H41 -21.90 -3.68

C29 H39 -23.90 -5.80

C28 H38 -19.50 -6.06

C35 H46 21.30 10.39

C35 H45 -10.20 -4.82

C47 H58 -25.00 -7.91

C54 H64 -19.70 -6.28

C56 H67 0.00 1.11

C56 H66 2.90 -5.69

C68 H79 -4.20 -10.39

C75 H85 -1.20 -9.94

C73 H83 -5.00 -9.20

C71 H81 -5.30 -11.16

C70 H80 -4.50 -11.53

C77 H88 -1.30 8.69

C77 H87 4.30 -10.29

Cornilescu Quality factor: 0.885707

Alignment tensor information:

A'x= 7.304e-05

A'y= 5.274e-04

A'z=-6.004e-04

Saupe tensor

S'x= 1.096e-04

S'y= 7.911e-04

S'z=-9.006e-04

Alignment tensor eigenvectors

e[x]=(-0.055, 0.387,-0.920)

e[y]=(-0.720, 0.623, 0.306)

e[z]=( 0.692, 0.680, 0.244)

Alignment tensor in laboratory coordinates:

[-1.376e-05,-5.204e-04,-2.138e-04]

[-5.204e-04,-6.150e-05,-2.529e-05]

[-2.138e-04,-2.529e-05,7.526e-05]

SVD condition number is 2.818e+01

Axial component Aa = -9.006e-04

Rhombic component Ar = -4.543e-04

Field=18.79 Teslas[ 2.27]

rhombicity R = 0.504

Asimmetry parameter etha =7.567e-01

GDO = 1.179e-03

ZY'Z'' Euler Angles (degrees)

Set 1

(44.5,75.9,18.4)

Set 2

(-135.5,-75.9,-161.6)

********Data set: #16

Computed data for frame #1

RDC Data:

I J Exp. [Hz] Comp. [Hz]

C5 H16 23.00 -3.92

C12 H22 25.20 -2.90

C14 H25 10.30 4.59

C14 H24 3.60 -2.87

C26 H37 -19.00 -5.73

C33 H43 -23.00 -4.92

C31 H41 -21.90 -4.53

C29 H39 -23.90 -6.34

C28 H38 -19.50 -6.16

C35 H46 21.30 9.84

C35 H45 -10.20 -5.16

C47 H58 -25.00 -8.25

C54 H64 -19.70 -7.17

C56 H67 0.00 4.38

C56 H66 2.90 -6.86

C68 H79 -4.20 -10.30

C75 H85 -1.20 -9.58

C73 H83 -5.00 -9.17

C71 H81 -5.30 -10.85

C70 H80 -4.50 -10.73

C77 H88 -1.30 9.09

C77 H87 4.30 -9.70

Cornilescu Quality factor: 0.878413

Alignment tensor information:

A'x= 6.798e-05

A'y= 4.014e-04

A'z=-4.693e-04

Saupe tensor

S'x= 1.020e-04

S'y= 6.020e-04

S'z=-7.040e-04

Alignment tensor eigenvectors

e[x]=( 0.167, 0.196,-0.966)

e[y]=(-0.689, 0.724, 0.028)

e[z]=( 0.705, 0.661, 0.256)

Alignment tensor in laboratory coordinates:

[-4.079e-05,-4.169e-04,-1.035e-04]

[-4.169e-04,7.838e-06,-8.431e-05]

[-1.035e-04,-8.431e-05,3.295e-05]

SVD condition number is 2.818e+01

Axial component Aa = -7.040e-04

Rhombic component Ar = -3.334e-04

Field=18.79 Teslas[ 2.27]

rhombicity R = 0.474

Asimmetry parameter etha =7.103e-01

GDO = 9.097e-04

ZY'Z'' Euler Angles (degrees)

Set 1

(43.2,75.2,1.7)

Set 2

(-136.8,-75.2,-178.3)

********

MSpin-RDC pluginju. jul. 22 17:37:11 2021

gt6

CR_tr

!* MSpin-RDC Plugin *!

********

!* Computation flags *!

Method: SVD

Scaling mode: Hz

Field (T): 18.7923

1H Larmor Frequency: 800.13

Scale QCSA with axial component: False

Include CSA gel shift (isotropic) correction:False

Optimize CSA gel shift (isotropic) correction scale:False

Estimate CSA gel shift (isotropic) correction scale:False

Gel Shift Correction Scale: 0.15

Single Tensor: False

Superimpose: False

Average methyl groups: False

Average methylene groups: False

Average phenyl groups: False

Bootstrapping: False

RDC Std. Error [ppm]: 1

CSA Std. Error [ppm]: 0.01

PCS Std. Error [ppm]: 0.01

DQ Std. Error [Hz]: 1

********

!* Permutations *!

There are no permutations on the original data set

********

Data set: #1

Computed data for frame #1

RDC Data:

I J Exp. [Hz] Comp. [Hz]

C5 H16 23.00 1.82

C12 H22 25.20 18.21

C26 H37 -19.00 -13.56

C33 H43 -23.00 -8.76

C31 H41 -21.90 -2.91

C29 H39 -23.90 -16.05

C28 H38 -19.50 -22.29

C47 H58 -25.00 -19.34

C54 H64 -19.70 -30.94

C68 H79 -4.20 -4.67

C75 H85 -1.20 -11.36

C73 H83 -5.00 -13.15

C71 H81 -5.30 2.77

C70 H80 -4.50 -3.89

Cornilescu Quality factor: 0.581749

Alignment tensor information:

A'x= 2.629e-04

A'y= 8.211e-02

A'z=-8.237e-02

Saupe tensor

S'x= 3.944e-04

S'y= 1.232e-01

S'z=-1.236e-01

Alignment tensor eigenvectors

e[x]=( 0.918,-0.242, 0.314)

e[y]=( 0.361, 0.185,-0.914)

e[z]=( 0.163, 0.953, 0.257)

Alignment tensor in laboratory coordinates:

[ 8.757e-03,-7.355e-03,-3.049e-02]

[-7.355e-03,-7.193e-02,-3.405e-02]

[-3.049e-02,-3.405e-02,6.317e-02]

SVD condition number is 1.095e+03

Axial component Aa = -1.236e-01

Rhombic component Ar = -8.185e-02

Field=18.79 Teslas[ 2.27]

rhombicity R = 0.662

Asimmetry parameter etha =9.936e-01

GDO = 1.744e-01

ZY'Z'' Euler Angles (degrees)

Set 1

(80.3,75.1,-109.0)

Set 2

(-99.7,-75.1,71.0)

********

MSpin-RDC pluginju. jul. 22 17:39:15 2021

gt6

E5C12

!* MSpin-RDC Plugin *!

********

!* Computation flags *!

Method: SVD

Scaling mode: Hz

Field (T): 18.7923

1H Larmor Frequency: 800.13

Scale QCSA with axial component: False

Include CSA gel shift (isotropic) correction:False

Optimize CSA gel shift (isotropic) correction scale:False

Estimate CSA gel shift (isotropic) correction scale:False

Gel Shift Correction Scale: 0.15

Single Tensor: False

Superimpose: False

Average methyl groups: False

Average methylene groups: False

Average phenyl groups: False

Bootstrapping: False

RDC Std. Error [ppm]: 1

CSA Std. Error [ppm]: 0.01

PCS Std. Error [ppm]: 0.01

DQ Std. Error [Hz]: 1

********

!* Permutations *!

There are no permutations on the original data set

********

Data set: #1

Computed data for frame #1

RDC Data:

I J Exp. [Hz] Comp. [Hz]

C5 H16 11.80 11.48

C12 H22 12.70 13.13

C26 H37 10.50 10.05

C33 H43 10.60 10.31

C47 H58 9.90 7.87

C54 H64 9.00 9.39

C68 H79 4.90 6.15

C75 H85 4.80 5.57

C71 H81 5.30 5.47

C70 H80 8.50 3.78

C77 H87 0.00 4.78

C77 H88 0.00 -0.01

Cornilescu Quality factor: 0.247671

Alignment tensor information:

A'x=-1.214e-04

A'y=-2.301e-03

A'z= 2.422e-03

Saupe tensor

S'x=-1.822e-04

S'y=-3.451e-03

S'z= 3.634e-03

Alignment tensor eigenvectors

e[x]=( 0.829,-0.339, 0.445)

e[y]=( 0.400, 0.915,-0.047)

e[z]=(-0.391, 0.218, 0.894)

Alignment tensor in laboratory coordinates:

[-8.139e-05,-1.015e-03,-8.486e-04]

[-1.015e-03,-1.826e-03,5.895e-04]

[-8.486e-04,5.895e-04,1.908e-03]

SVD condition number is 9.229e+02

Axial component Aa = 3.634e-03

Rhombic component Ar = 2.179e-03

Field=18.79 Teslas[ 2.27]

rhombicity R = 0.600

Asimmetry parameter etha =8.997e-01

GDO = 4.973e-03

ZY'Z'' Euler Angles (degrees)

Set 1

(150.9,26.6,-173.9)

Set 2

(-29.1,-26.6,6.1)

********

MSpin-RDC pluginju. jul. 22 17:43:19 2021

gt6F

CRF_all

!* MSpin-RDC Plugin *!

********

!* Computation flags *!

Method: SVD

Scaling mode: Hz

Field (T): 18.7923

1H Larmor Frequency: 800.13

Scale QCSA with axial component: False

Include CSA gel shift (isotropic) correction:False

Optimize CSA gel shift (isotropic) correction scale:False

Estimate CSA gel shift (isotropic) correction scale:False

Gel Shift Correction Scale: 0.15

Single Tensor: False

Superimpose: False

Average methyl groups: False

Average methylene groups: False

Average phenyl groups: False

Bootstrapping: False

RDC Std. Error [ppm]: 1

CSA Std. Error [ppm]: 0.01

PCS Std. Error [ppm]: 0.01

DQ Std. Error [Hz]: 1

********

!* Permutations *!

There are 4 possible permutations on the original data set

********

Data set: #1

Computed data for frame #1

RDC Data:

I J Exp. [Hz] Comp. [Hz]

C5 H16 18.00 -3.34

C10 H20 23.00 6.45

C10 F11 -13.00 -6.76

C25 H36 -19.00 -16.36

C32 H42 -23.00 -0.60

C30 H40 -21.90 -7.96

C28 H38 -23.90 -14.70

C27 H37 -19.50 4.14

C34 H44 21.30 4.89

C34 H45 -10.20 -7.38

C46 H57 -30.00 -25.59

C51 H61 -24.00 -18.21

C51 F52 13.00 7.55

C66 H77 -4.20 -20.20

C73 H83 -1.20 -4.02

C71 H81 -5.00 -11.86

C69 H79 -5.30 -17.54

C68 H78 -4.50 1.28

C75 H85 -1.30 2.39

C75 H86 4.30 1.12

Cornilescu Quality factor: 0.727229

Alignment tensor information:

A'x=-2.386e-03

A'y=-2.525e-03

A'z= 4.911e-03

Saupe tensor

S'x=-3.579e-03

S'y=-3.788e-03

S'z= 7.367e-03

Alignment tensor eigenvectors

e[x]=( 0.960, 0.211, 0.182)

e[y]=( 0.200,-0.063,-0.978)

e[z]=(-0.195, 0.975,-0.103)

Alignment tensor in laboratory coordinates:

[-2.115e-03,-1.384e-03,1.735e-04]

[-1.384e-03,4.557e-03,-7.411e-04]

[ 1.735e-04,-7.411e-04,-2.442e-03]

SVD condition number is 4.178e+01

Axial component Aa = 7.367e-03

Rhombic component Ar = 1.397e-04

Field=18.79 Teslas[ 2.27]

rhombicity R = 0.019

Asimmetry parameter etha =2.845e-02

GDO = 8.508e-03

ZY'Z'' Euler Angles (degrees)

Set 1

(101.3,95.9,-100.6)

Set 2

(-78.7,-95.9,79.4)

********Data set: #2

Computed data for frame #1

RDC Data:

I J Exp. [Hz] Comp. [Hz]

C5 H16 18.00 -6.49

C10 H20 23.00 0.37

C10 F11 -13.00 -3.58

C25 H36 -19.00 -15.60

C32 H42 -23.00 -4.63

C30 H40 -21.90 -9.75

C28 H38 -23.90 -14.48

C27 H37 -19.50 -1.38

C34 H45 21.30 10.05

C34 H44 -10.20 -0.83

C46 H57 -30.00 -22.04

C51 H61 -24.00 -16.91

C51 F52 13.00 6.31

C66 H77 -4.20 -18.39

C73 H83 -1.20 -7.21

C71 H81 -5.00 -12.61

C69 H79 -5.30 -16.60

C68 H78 -4.50 -3.61

C75 H85 -1.30 -2.79

C75 H86 4.30 15.39

Cornilescu Quality factor: 0.732537

Alignment tensor information:

A'x=-1.568e-03

A'y=-1.736e-03

A'z= 3.303e-03

Saupe tensor

S'x=-2.352e-03

S'y=-2.603e-03

S'z= 4.955e-03

Alignment tensor eigenvectors

e[x]=(-0.126, 0.068, 0.990)

e[y]=( 0.978, 0.175, 0.113)

e[z]=(-0.165, 0.982,-0.089)

Alignment tensor in laboratory coordinates:

[-1.596e-03,-8.185e-04,5.265e-05]

[-8.185e-04,3.127e-03,-4.271e-04]

[ 5.265e-05,-4.271e-04,-1.532e-03]

SVD condition number is 4.178e+01

Axial component Aa = 4.955e-03

Rhombic component Ar = 1.679e-04

Field=18.79 Teslas[ 2.27]

rhombicity R = 0.034

Asimmetry parameter etha =5.083e-02

GDO = 5.725e-03

ZY'Z'' Euler Angles (degrees)

Set 1

(99.5,95.1,173.5)

Set 2

(-80.5,-95.1,-6.5)

********Data set: #3

Computed data for frame #1

RDC Data:

I J Exp. [Hz] Comp. [Hz]

C5 H16 18.00 -3.39

C10 H20 23.00 6.41

C10 F11 -13.00 -6.69

C25 H36 -19.00 -16.50

C32 H42 -23.00 -0.34

C30 H40 -21.90 -7.99

C28 H38 -23.90 -14.70

C27 H37 -19.50 4.72

C34 H44 21.30 5.37

C34 H45 -10.20 -10.36

C46 H57 -30.00 -25.68

C51 H61 -24.00 -18.35

C51 F52 13.00 7.82

C66 H77 -4.20 -19.75

C73 H83 -1.20 -3.14

C71 H81 -5.00 -11.30

C69 H79 -5.30 -16.93

C68 H78 -4.50 2.48

C75 H86 -1.30 -1.42

C75 H85 4.30 3.50

Cornilescu Quality factor: 0.723003

Alignment tensor information:

A'x=-2.391e-03

A'y=-2.669e-03

A'z= 5.060e-03

Saupe tensor

S'x=-3.587e-03

S'y=-4.003e-03

S'z= 7.590e-03

Alignment tensor eigenvectors

e[x]=( 0.928, 0.219, 0.301)

e[y]=( 0.316,-0.037,-0.948)

e[z]=(-0.197, 0.975,-0.103)

Alignment tensor in laboratory coordinates:

[-2.130e-03,-1.427e-03,2.346e-04]

[-1.427e-03,4.691e-03,-7.596e-04]

[ 2.346e-04,-7.596e-04,-2.561e-03]

SVD condition number is 4.178e+01

Axial component Aa = 7.590e-03

Rhombic component Ar = 2.773e-04

Field=18.79 Teslas[ 2.27]

rhombicity R = 0.037

Asimmetry parameter etha =5.481e-02

GDO = 8.770e-03

ZY'Z'' Euler Angles (degrees)

Set 1

(101.4,95.9,-107.6)

Set 2

(-78.6,-95.9,72.4)

********Data set: #4

Computed data for frame #1

RDC Data:

I J Exp. [Hz] Comp. [Hz]

C5 H16 18.00 -6.54

C10 H20 23.00 0.34

C10 F11 -13.00 -3.51

C25 H36 -19.00 -15.74

C32 H42 -23.00 -4.38

C30 H40 -21.90 -9.78

C28 H38 -23.90 -14.48

C27 H37 -19.50 -0.80

C34 H45 21.30 7.07

C34 H44 -10.20 -0.35

C46 H57 -30.00 -22.13

C51 H61 -24.00 -17.05

C51 F52 13.00 6.58

C66 H77 -4.20 -17.95

C73 H83 -1.20 -6.33

C71 H81 -5.00 -12.05

C69 H79 -5.30 -15.99

C68 H78 -4.50 -2.40

C75 H86 -1.30 12.85

C75 H85 4.30 -1.68

Cornilescu Quality factor: 0.75431

Alignment tensor information:

A'x=-1.676e-03

A'y=-1.775e-03

A'z= 3.451e-03

Saupe tensor

S'x=-2.514e-03

S'y=-2.662e-03

S'z= 5.176e-03

Alignment tensor eigenvectors

e[x]=( 0.383, 0.149, 0.912)

e[y]=( 0.908, 0.120,-0.401)

e[z]=(-0.169, 0.982,-0.090)

Alignment tensor in laboratory coordinates:

[-1.611e-03,-8.620e-04,1.138e-04]

[-8.620e-04,3.261e-03,-4.455e-04]

[ 1.138e-04,-4.455e-04,-1.651e-03]

SVD condition number is 4.178e+01

Axial component Aa = 5.176e-03

Rhombic component Ar = 9.919e-05

Field=18.79 Teslas[ 2.27]

rhombicity R = 0.019

Asimmetry parameter etha =2.875e-02

GDO = 5.978e-03

ZY'Z'' Euler Angles (degrees)

Set 1

(99.8,95.1,-156.2)

Set 2

(-80.2,-95.1,23.8)

********

MSpin-RDC pluginju. jul. 22 16:46:10 2021

gt6F

CRF_tr

!* MSpin-RDC Plugin *!

********

!* Computation flags *!

Method: SVD

Scaling mode: Hz

Field (T): 18.7923

1H Larmor Frequency: 800.13

Scale QCSA with axial component: False

Include CSA gel shift (isotropic) correction:False

Optimize CSA gel shift (isotropic) correction scale:False

Estimate CSA gel shift (isotropic) correction scale:False

Gel Shift Correction Scale: 0.15

Single Tensor: False

Superimpose: False

Average methyl groups: False

Average methylene groups: False

Average phenyl groups: False

Bootstrapping: False

RDC Std. Error [ppm]: 1

CSA Std. Error [ppm]: 0.01

PCS Std. Error [ppm]: 0.01

DQ Std. Error [Hz]: 1

********

!* Permutations *!

There are no permutations on the original data set

********

Data set: #1

Computed data for frame #1

RDC Data:

I J Exp. [Hz] Comp. [Hz]

C5 H16 18.00 -4.33

C10 H20 23.00 3.81

C10 F11 -13.00 -19.95

C25 H36 -19.00 -14.21

C32 H42 -23.00 -7.91

C30 H40 -21.90 -8.72

C28 H38 -23.90 -14.79

C27 H37 -19.50 -7.58

C46 H57 -30.00 -23.29

C51 H61 -24.00 -15.97

C51 F52 13.00 20.36

C66 H77 -4.20 -17.58

C73 H83 -1.20 -5.89

C71 H81 -5.00 -12.79

C69 H79 -5.30 -13.92

C68 H78 -4.50 2.49

Cornilescu Quality factor: 0.644921

Alignment tensor information:

A'x= 6.915e-05

A'y= 2.361e-02

A'z=-2.368e-02

Saupe tensor

S'x= 1.037e-04

S'y= 3.541e-02

S'z=-3.552e-02

Alignment tensor eigenvectors

e[x]=( 0.706, 0.662,-0.252)

e[y]=(-0.708, 0.678,-0.199)

e[z]=( 0.039, 0.318, 0.947)

Alignment tensor in laboratory coordinates:

[ 1.182e-02,-1.159e-02,2.434e-03]

[-1.159e-02,8.485e-03,-1.034e-02]

[ 2.434e-03,-1.034e-02,-2.030e-02]

SVD condition number is 3.000e+02

Axial component Aa = -3.552e-02

Rhombic component Ar = -2.354e-02

Field=18.79 Teslas[ 2.27]

rhombicity R = 0.663

Asimmetry parameter etha =9.942e-01

GDO = 5.013e-02

ZY'Z'' Euler Angles (degrees)

Set 1

(83.0,18.7,-38.3)

Set 2

(-97.0,-18.7,141.7)

********

MSpin-RDC pluginmi. jul. 21 16:29:48 2021

(gg/gt)3

CR_all

!* MSpin-RDC Plugin *!

********

!* Computation flags *!

Method: SVD

Scaling mode: Hz

Field (T): 18.7923

1H Larmor Frequency: 800.13

Scale QCSA with axial component: False

Include CSA gel shift (isotropic) correction:False

Optimize CSA gel shift (isotropic) correction scale:False

Estimate CSA gel shift (isotropic) correction scale:False

Gel Shift Correction Scale: 1

Single Tensor: False

Superimpose: False

Average methyl groups: False

Average methylene groups: False

Average phenyl groups: False

Bootstrapping: False

RDC Std. Error [ppm]: 1

CSA Std. Error [ppm]: 0.01

PCS Std. Error [ppm]: 0.01

DQ Std. Error [Hz]: 1

********

!* Permutations *!

There are 16 possible permutations on the original data set

********

Data set: #1

Computed data for frame #1

RDC Data:

I J Exp. [Hz] Comp. [Hz]

C5 H16 23.00 22.70

C12 H22 25.20 18.55

C14 H24 10.30 -9.85

C14 H25 3.60 -6.07

C26 H37 -19.00 -12.54

C33 H43 -23.00 -9.08

C31 H41 -21.90 -9.59

C29 H39 -23.90 -13.42

C28 H38 -19.50 -10.36

C35 H45 21.30 -9.31

C35 H46 -10.20 -1.92

C47 H58 -25.00 -19.25

C54 H64 -19.70 -19.33

C56 H66 0.00 -3.55

C56 H67 2.90 15.54

C68 H79 -4.20 4.08

C75 H85 -1.20 -0.60

C73 H83 -5.00 2.32

C71 H81 -5.30 2.50

C70 H80 -4.50 -3.42

C77 H87 -1.30 -3.56

C77 H88 4.30 4.10

Cornilescu Quality factor: 0.690509

Alignment tensor information:

A'x= 2.104e-04

A'y= 1.204e-03

A'z=-1.415e-03

Saupe tensor

S'x= 3.157e-04

S'y= 1.807e-03

S'z=-2.122e-03

Alignment tensor eigenvectors

e[x]=(-0.271, 0.509,-0.817)

e[y]=( 0.599, 0.753, 0.271)

e[z]=( 0.753,-0.416,-0.509)

Alignment tensor in laboratory coordinates:

[-3.545e-04,9.584e-04,7.846e-04]

[ 9.584e-04,4.927e-04,-1.419e-04]

[ 7.846e-04,-1.419e-04,-1.382e-04]

SVD condition number is 1.866e+01

Axial component Aa = -2.122e-03

Rhombic component Ar = -9.939e-04

Field=18.79 Teslas[ 2.27]

rhombicity R = 0.468

Asimmetry parameter etha =7.025e-01

GDO = 2.736e-03

ZY'Z'' Euler Angles (degrees)

Set 1

(-28.9,120.6,18.3)

Set 2

(151.1,-120.6,-161.7)

********Data set: #2

Computed data for frame #1

RDC Data:

I J Exp. [Hz] Comp. [Hz]

C5 H16 23.00 22.61

C12 H22 25.20 19.76

C14 H25 10.30 -2.26

C14 H24 3.60 -14.17

C26 H37 -19.00 -13.30

C33 H43 -23.00 -10.15

C31 H41 -21.90 -11.20

C29 H39 -23.90 -13.59

C28 H38 -19.50 -10.14

C35 H45 21.30 -9.75

C35 H46 -10.20 -2.64

C47 H58 -25.00 -17.60

C54 H64 -19.70 -18.82

C56 H66 0.00 -2.17

C56 H67 2.90 15.43

C68 H79 -4.20 3.25

C75 H85 -1.20 0.21

C73 H83 -5.00 2.70

C71 H81 -5.30 1.70

C70 H80 -4.50 -2.63

C77 H87 -1.30 -1.66

C77 H88 4.30 5.96

Cornilescu Quality factor: 0.675976

Alignment tensor information:

A'x=-1.430e-04

A'y=-9.741e-04

A'z= 1.117e-03

Saupe tensor

S'x=-2.145e-04

S'y=-1.461e-03

S'z= 1.676e-03

Alignment tensor eigenvectors

e[x]=( 0.172,-0.489, 0.855)

e[y]=( 0.788,-0.453,-0.417)

e[z]=( 0.591, 0.745, 0.308)

Alignment tensor in laboratory coordinates:

[-2.191e-04,8.517e-04,5.026e-04]

[ 8.517e-04,3.871e-04,1.325e-04]

[ 5.026e-04,1.325e-04,-1.680e-04]

SVD condition number is 1.866e+01

Axial component Aa = 1.676e-03

Rhombic component Ar = 8.311e-04

Field=18.79 Teslas[ 2.27]

rhombicity R = 0.496

Asimmetry parameter etha =7.440e-01

GDO = 2.186e-03

ZY'Z'' Euler Angles (degrees)

Set 1

(51.6,72.1,-154.0)

Set 2

(-128.4,-72.1,26.0)

********Data set: #3

Computed data for frame #1

RDC Data:

I J Exp. [Hz] Comp. [Hz]

C5 H16 23.00 14.95

C12 H22 25.20 11.33

C14 H24 10.30 -11.43

C14 H25 3.60 -8.96

C26 H37 -19.00 -18.05

C33 H43 -23.00 -16.78

C31 H41 -21.90 -15.99

C29 H39 -23.90 -19.27

C28 H38 -19.50 -19.21

C35 H46 21.30 10.80

C35 H45 -10.20 -17.89

C47 H58 -25.00 -23.46

C54 H64 -19.70 -21.63

C56 H66 0.00 -4.72

C56 H67 2.90 13.91

C68 H79 -4.20 0.67

C75 H85 -1.20 -3.05

C73 H83 -5.00 -1.58

C71 H81 -5.30 0.14

C70 H80 -4.50 -3.84

C77 H87 -1.30 -5.46

C77 H88 4.30 3.70

Cornilescu Quality factor: 0.512058

Alignment tensor information:

A'x= 4.064e-04

A'y= 7.667e-04

A'z=-1.173e-03

Saupe tensor

S'x= 6.097e-04

S'y= 1.150e-03

S'z=-1.760e-03

Alignment tensor eigenvectors

e[x]=( 0.530, 0.805, 0.267)

e[y]=( 0.325,-0.483, 0.813)

e[z]=( 0.783,-0.344,-0.518)

Alignment tensor in laboratory coordinates:

[-5.250e-04,3.685e-04,7.361e-04]

[ 3.685e-04,3.042e-04,-4.225e-04]

[ 7.361e-04,-4.225e-04,2.208e-04]

SVD condition number is 1.866e+01

Axial component Aa = -1.760e-03

Rhombic component Ar = -3.603e-04

Field=18.79 Teslas[ 2.27]

rhombicity R = 0.205

Asimmetry parameter etha =3.071e-01

GDO = 2.079e-03

ZY'Z'' Euler Angles (degrees)

Set 1

(-23.7,121.2,108.2)

Set 2

(156.3,-121.2,-71.8)

********Data set: #4

Computed data for frame #1

RDC Data:

I J Exp. [Hz] Comp. [Hz]

C5 H16 23.00 14.87

C12 H22 25.20 12.54

C14 H25 10.30 -5.15

C14 H24 3.60 -15.75

C26 H37 -19.00 -18.81

C33 H43 -23.00 -17.85

C31 H41 -21.90 -17.61

C29 H39 -23.90 -19.45

C28 H38 -19.50 -18.99

C35 H46 21.30 10.08

C35 H45 -10.20 -18.33

C47 H58 -25.00 -21.82

C54 H64 -19.70 -21.11

C56 H66 0.00 -3.34

C56 H67 2.90 13.80

C68 H79 -4.20 -0.16

C75 H85 -1.20 -2.24

C73 H83 -5.00 -1.20

C71 H81 -5.30 -0.65

C70 H80 -4.50 -3.05

C77 H87 -1.30 -3.57

C77 H88 4.30 5.57

Cornilescu Quality factor: 0.495638

Alignment tensor information:

A'x= 2.953e-04

A'y= 4.398e-04

A'z=-7.351e-04

Saupe tensor

S'x= 4.430e-04

S'y= 6.597e-04

S'z=-1.103e-03

Alignment tensor eigenvectors

e[x]=( 0.282, 0.951,-0.123)

e[y]=( 0.474,-0.027, 0.880)

e[z]=( 0.834,-0.307,-0.458)

Alignment tensor in laboratory coordinates:

[-3.896e-04,2.617e-04,4.542e-04]

[ 2.617e-04,1.986e-04,-1.481e-04]

[ 4.542e-04,-1.481e-04,1.909e-04]

SVD condition number is 1.866e+01

Axial component Aa = -1.103e-03

Rhombic component Ar = -1.445e-04

Field=18.79 Teslas[ 2.27]

rhombicity R = 0.131

Asimmetry parameter etha =1.965e-01

GDO = 1.286e-03

ZY'Z'' Euler Angles (degrees)

Set 1

(-20.2,117.3,82.1)

Set 2

(159.8,-117.3,-97.9)

********Data set: #5

Computed data for frame #1

RDC Data:

I J Exp. [Hz] Comp. [Hz]

C5 H16 23.00 21.94

C12 H22 25.20 17.83

C14 H24 10.30 -9.91

C14 H25 3.60 -5.48

C26 H37 -19.00 -12.47

C33 H43 -23.00 -8.99

C31 H41 -21.90 -9.53

C29 H39 -23.90 -13.33

C28 H38 -19.50 -10.23

C35 H45 21.30 -9.20

C35 H46 -10.20 -1.77

C47 H58 -25.00 -19.15

C54 H64 -19.70 -19.28

C56 H67 0.00 14.72

C56 H66 2.90 -3.07

C68 H79 -4.20 4.46

C75 H85 -1.20 -0.15

C73 H83 -5.00 2.75

C71 H81 -5.30 2.88

C70 H80 -4.50 -2.97

C77 H87 -1.30 -3.05

C77 H88 4.30 4.54

Cornilescu Quality factor: 0.704981

Alignment tensor information:

A'x= 1.915e-04

A'y= 1.205e-03

A'z=-1.397e-03

Saupe tensor

S'x= 2.873e-04

S'y= 1.808e-03

S'z=-2.095e-03

Alignment tensor eigenvectors

e[x]=(-0.266, 0.516,-0.814)

e[y]=( 0.603, 0.748, 0.277)

e[z]=( 0.752,-0.417,-0.510)

Alignment tensor in laboratory coordinates:

[-3.378e-04,9.560e-04,7.786e-04]

[ 9.560e-04,4.822e-04,-1.284e-04]

[ 7.786e-04,-1.284e-04,-1.444e-04]

SVD condition number is 1.866e+01

Axial component Aa = -2.095e-03

Rhombic component Ar = -1.014e-03

Field=18.79 Teslas[ 2.27]

rhombicity R = 0.484

Asimmetry parameter etha =7.258e-01

GDO = 2.720e-03

ZY'Z'' Euler Angles (degrees)

Set 1

(-29.0,120.7,18.8)

Set 2

(151.0,-120.7,-161.2)

********Data set: #6

Computed data for frame #1

RDC Data:

I J Exp. [Hz] Comp. [Hz]

C5 H16 23.00 21.86

C12 H22 25.20 19.04

C14 H25 10.30 -1.67

C14 H24 3.60 -14.23

C26 H37 -19.00 -13.23

C33 H43 -23.00 -10.07

C31 H41 -21.90 -11.14

C29 H39 -23.90 -13.51

C28 H38 -19.50 -10.01

C35 H45 21.30 -9.64

C35 H46 -10.20 -2.49

C47 H58 -25.00 -17.50

C54 H64 -19.70 -18.76

C56 H67 0.00 14.61

C56 H66 2.90 -1.69

C68 H79 -4.20 3.63

C75 H85 -1.20 0.66

C73 H83 -5.00 3.14

C71 H81 -5.30 2.09

C70 H80 -4.50 -2.17

C77 H87 -1.30 -1.16

C77 H88 4.30 6.41

Cornilescu Quality factor: 0.689569

Alignment tensor information:

A'x=-1.622e-04

A'y=-9.562e-04

A'z= 1.118e-03

Saupe tensor

S'x=-2.434e-04

S'y=-1.434e-03

S'z= 1.678e-03

Alignment tensor eigenvectors

e[x]=( 0.165,-0.492, 0.855)

e[y]=( 0.787,-0.456,-0.415)

e[z]=( 0.594, 0.741, 0.312)

Alignment tensor in laboratory coordinates:

[-2.024e-04,8.493e-04,4.967e-04]

[ 8.493e-04,3.766e-04,1.460e-04]

[ 4.967e-04,1.460e-04,-1.742e-04]

SVD condition number is 1.866e+01

Axial component Aa = 1.678e-03

Rhombic component Ar = 7.940e-04

Field=18.79 Teslas[ 2.27]

rhombicity R = 0.473

Asimmetry parameter etha =7.099e-01

GDO = 2.168e-03

ZY'Z'' Euler Angles (degrees)

Set 1

(51.3,71.8,-154.1)

Set 2

(-128.7,-71.8,25.9)

********Data set: #7

Computed data for frame #1

RDC Data:

I J Exp. [Hz] Comp. [Hz]

C5 H16 23.00 14.19

C12 H22 25.20 10.61

C14 H24 10.30 -11.49

C14 H25 3.60 -8.37

C26 H37 -19.00 -17.99

C33 H43 -23.00 -16.69

C31 H41 -21.90 -15.94

C29 H39 -23.90 -19.19

C28 H38 -19.50 -19.08

C35 H46 21.30 10.95

C35 H45 -10.20 -17.78

C47 H58 -25.00 -23.36

C54 H64 -19.70 -21.57

C56 H67 0.00 13.09

C56 H66 2.90 -4.24

C68 H79 -4.20 1.04

C75 H85 -1.20 -2.60

C73 H83 -5.00 -1.15

C71 H81 -5.30 0.52

C70 H80 -4.50 -3.38

C77 H87 -1.30 -4.96

C77 H88 4.30 4.15

Cornilescu Quality factor: 0.530936

Alignment tensor information:

A'x= 4.057e-04

A'y= 7.492e-04

A'z=-1.155e-03

Saupe tensor

S'x= 6.085e-04

S'y= 1.124e-03

S'z=-1.732e-03

Alignment tensor eigenvectors

e[x]=( 0.522, 0.818, 0.243)

e[y]=( 0.341,-0.461, 0.819)

e[z]=( 0.782,-0.344,-0.520)

Alignment tensor in laboratory coordinates:

[-5.083e-04,3.661e-04,7.302e-04]

[ 3.661e-04,2.938e-04,-4.091e-04]

[ 7.302e-04,-4.091e-04,2.146e-04]

SVD condition number is 1.866e+01

Axial component Aa = -1.732e-03

Rhombic component Ar = -3.435e-04

Field=18.79 Teslas[ 2.27]

rhombicity R = 0.198

Asimmetry parameter etha =2.974e-01

GDO = 2.044e-03

ZY'Z'' Euler Angles (degrees)

Set 1

(-23.8,121.3,106.5)

Set 2

(156.2,-121.3,-73.5)

********Data set: #8

Computed data for frame #1

RDC Data:

I J Exp. [Hz] Comp. [Hz]

C5 H16 23.00 14.11

C12 H22 25.20 11.83

C14 H25 10.30 -4.56

C14 H24 3.60 -15.81

C26 H37 -19.00 -18.74

C33 H43 -23.00 -17.76

C31 H41 -21.90 -17.55

C29 H39 -23.90 -19.36

C28 H38 -19.50 -18.86

C35 H46 21.30 10.23

C35 H45 -10.20 -18.22

C47 H58 -25.00 -21.71

C54 H64 -19.70 -21.05

C56 H67 0.00 12.98

C56 H66 2.90 -2.86

C68 H79 -4.20 0.22

C75 H85 -1.20 -1.79

C73 H83 -5.00 -0.77

C71 H81 -5.30 -0.27

C70 H80 -4.50 -2.59

C77 H87 -1.30 -3.06

C77 H88 4.30 6.02

Cornilescu Quality factor: 0.513529

Alignment tensor information:

A'x= 2.821e-04

A'y= 4.341e-04

A'z=-7.162e-04

Saupe tensor

S'x= 4.231e-04

S'y= 6.512e-04

S'z=-1.074e-03

Alignment tensor eigenvectors

e[x]=( 0.243, 0.950,-0.194)

e[y]=( 0.497, 0.050, 0.866)

e[z]=( 0.833,-0.307,-0.460)

Alignment tensor in laboratory coordinates:

[-3.729e-04,2.593e-04,4.482e-04]

[ 2.593e-04,1.882e-04,-1.347e-04]

[ 4.482e-04,-1.347e-04,1.847e-04]

SVD condition number is 1.866e+01

Axial component Aa = -1.074e-03

Rhombic component Ar = -1.521e-04

Field=18.79 Teslas[ 2.27]

rhombicity R = 0.142

Asimmetry parameter etha =2.123e-01

GDO = 1.254e-03

ZY'Z'' Euler Angles (degrees)

Set 1

(-20.3,117.4,77.4)

Set 2

(159.7,-117.4,-102.6)

********Data set: #9

Computed data for frame #1

RDC Data:

I J Exp. [Hz] Comp. [Hz]

C5 H16 23.00 22.30

C12 H22 25.20 18.49

C14 H24 10.30 -9.13

C14 H25 3.60 -5.33

C26 H37 -19.00 -12.48

C33 H43 -23.00 -9.72

C31 H41 -21.90 -9.92

C29 H39 -23.90 -13.37

C28 H38 -19.50 -11.16

C35 H45 21.30 -10.12

C35 H46 -10.20 -3.00

C47 H58 -25.00 -18.47

C54 H64 -19.70 -18.16

C56 H66 0.00 -2.52

C56 H67 2.90 16.47

C68 H79 -4.20 4.49

C75 H85 -1.20 0.11

C73 H83 -5.00 2.68

C71 H81 -5.30 3.15

C70 H80 -4.50 -2.25

C77 H88 -1.30 0.17

C77 H87 4.30 -2.67

Cornilescu Quality factor: 0.698498

Alignment tensor information:

A'x= 3.047e-04

A'y= 9.978e-04

A'z=-1.303e-03

Saupe tensor

S'x= 4.571e-04

S'y= 1.497e-03

S'z=-1.954e-03

Alignment tensor eigenvectors

e[x]=(-0.280, 0.479,-0.832)

e[y]=( 0.589, 0.770, 0.245)

e[z]=( 0.758,-0.422,-0.498)

Alignment tensor in laboratory coordinates:

[-3.784e-04,8.282e-04,7.062e-04]

[ 8.282e-04,4.300e-04,-2.065e-04]

[ 7.062e-04,-2.065e-04,-5.153e-05]

SVD condition number is 1.866e+01

Axial component Aa = -1.954e-03

Rhombic component Ar = -6.930e-04

Field=18.79 Teslas[ 2.27]

rhombicity R = 0.355

Asimmetry parameter etha =5.321e-01

GDO = 2.410e-03

ZY'Z'' Euler Angles (degrees)

Set 1

(-29.1,119.8,16.4)

Set 2

(150.9,-119.8,-163.6)

********Data set: #10

Computed data for frame #1

RDC Data:

I J Exp. [Hz] Comp. [Hz]

C5 H16 23.00 22.21

C12 H22 25.20 19.70

C14 H25 10.30 -1.52

C14 H24 3.60 -13.45

C26 H37 -19.00 -13.24

C33 H43 -23.00 -10.79

C31 H41 -21.90 -11.53

C29 H39 -23.90 -13.55

C28 H38 -19.50 -10.94

C35 H45 21.30 -10.56

C35 H46 -10.20 -3.72

C47 H58 -25.00 -16.82

C54 H64 -19.70 -17.64

C56 H66 0.00 -1.13

C56 H67 2.90 16.36

C68 H79 -4.20 3.66

C75 H85 -1.20 0.92

C73 H83 -5.00 3.07

C71 H81 -5.30 2.36

C70 H80 -4.50 -1.45

C77 H88 -1.30 2.03

C77 H87 4.30 -0.78

Cornilescu Quality factor: 0.684091

Alignment tensor information:

A'x=-4.196e-05

A'y=-8.673e-04

A'z= 9.093e-04

Saupe tensor

S'x=-6.293e-05

S'y=-1.301e-03

S'z= 1.364e-03

Alignment tensor eigenvectors

e[x]=( 0.156,-0.468, 0.870)

e[y]=( 0.797,-0.460,-0.391)

e[z]=( 0.583, 0.754, 0.301)

Alignment tensor in laboratory coordinates:

[-2.430e-04,7.214e-04,4.242e-04]

[ 7.214e-04,3.243e-04,6.791e-05]

[ 4.242e-04,6.791e-05,-8.138e-05]

SVD condition number is 1.866e+01

Axial component Aa = 1.364e-03

Rhombic component Ar = 8.253e-04

Field=18.79 Teslas[ 2.27]

rhombicity R = 0.605

Asimmetry parameter etha =9.077e-01

GDO = 1.871e-03

ZY'Z'' Euler Angles (degrees)

Set 1

(52.3,72.5,-155.8)

Set 2

(-127.7,-72.5,24.2)

********Data set: #11

Computed data for frame #1

RDC Data:

I J Exp. [Hz] Comp. [Hz]

C5 H16 23.00 14.55

C12 H22 25.20 11.27

C14 H24 10.30 -10.72

C14 H25 3.60 -8.22

C26 H37 -19.00 -18.00

C33 H43 -23.00 -17.41

C31 H41 -21.90 -16.32

C29 H39 -23.90 -19.23

C28 H38 -19.50 -20.00

C35 H46 21.30 9.72

C35 H45 -10.20 -18.70

C47 H58 -25.00 -22.68

C54 H64 -19.70 -20.45

C56 H66 0.00 -3.69

C56 H67 2.90 14.83

C68 H79 -4.20 1.08

C75 H85 -1.20 -2.34

C73 H83 -5.00 -1.22

C71 H81 -5.30 0.80

C70 H80 -4.50 -2.66

C77 H88 -1.30 -0.23

C77 H87 4.30 -4.58

Cornilescu Quality factor: 0.525828

Alignment tensor information:

A'x= 2.028e-04

A'y= 8.593e-04

A'z=-1.062e-03

Saupe tensor

S'x= 3.041e-04

S'y= 1.289e-03

S'z=-1.593e-03

Alignment tensor eigenvectors

e[x]=( 0.530, 0.785, 0.322)

e[y]=( 0.287,-0.523, 0.803)

e[z]=( 0.798,-0.333,-0.502)

Alignment tensor in laboratory coordinates:

[-5.489e-04,2.382e-04,6.577e-04]

[ 2.382e-04,2.415e-04,-4.871e-04]

[ 6.577e-04,-4.871e-04,3.074e-04]

SVD condition number is 1.866e+01

Axial component Aa = -1.593e-03

Rhombic component Ar = -6.565e-04

Field=18.79 Teslas[ 2.27]

rhombicity R = 0.412

Asimmetry parameter etha =6.182e-01

GDO = 2.008e-03

ZY'Z'' Euler Angles (degrees)

Set 1

(-22.7,120.1,111.8)

Set 2

(157.3,-120.1,-68.2)

********Data set: #12

Computed data for frame #1

RDC Data:

I J Exp. [Hz] Comp. [Hz]

C5 H16 23.00 14.47

C12 H22 25.20 12.48

C14 H25 10.30 -4.41

C14 H24 3.60 -15.04

C26 H37 -19.00 -18.76

C33 H43 -23.00 -18.49

C31 H41 -21.90 -17.93

C29 H39 -23.90 -19.40

C28 H38 -19.50 -19.78

C35 H46 21.30 9.00

C35 H45 -10.20 -19.15

C47 H58 -25.00 -21.03

C54 H64 -19.70 -19.93

C56 H66 0.00 -2.31

C56 H67 2.90 14.72

C68 H79 -4.20 0.25

C75 H85 -1.20 -1.53

C73 H83 -5.00 -0.84

C71 H81 -5.30 0.00

C70 H80 -4.50 -1.87

C77 H88 -1.30 1.64

C77 H87 4.30 -2.68

Cornilescu Quality factor: 0.509794

Alignment tensor information:

A'x= 1.309e-04

A'y= 5.038e-04

A'z=-6.347e-04

Saupe tensor

S'x= 1.964e-04

S'y= 7.557e-04

S'z=-9.521e-04

Alignment tensor eigenvectors

e[x]=( 0.395, 0.880, 0.264)

e[y]=( 0.299,-0.395, 0.868)

e[z]=( 0.869,-0.264,-0.419)

Alignment tensor in laboratory coordinates:

[-4.134e-04,1.314e-04,3.757e-04]

[ 1.314e-04,1.359e-04,-2.127e-04]

[ 3.757e-04,-2.127e-04,2.776e-04]

SVD condition number is 1.866e+01

Axial component Aa = -9.521e-04

Rhombic component Ar = -3.729e-04

Field=18.79 Teslas[ 2.27]

rhombicity R = 0.392

Asimmetry parameter etha =5.875e-01

GDO = 1.190e-03

ZY'Z'' Euler Angles (degrees)

Set 1

(-16.9,114.8,106.9)

Set 2

(163.1,-114.8,-73.1)

********Data set: #13

Computed data for frame #1

RDC Data:

I J Exp. [Hz] Comp. [Hz]

C5 H16 23.00 21.54

C12 H22 25.20 17.77

C14 H24 10.30 -9.19

C14 H25 3.60 -4.74

C26 H37 -19.00 -12.42

C33 H43 -23.00 -9.63

C31 H41 -21.90 -9.86

C29 H39 -23.90 -13.29

C28 H38 -19.50 -11.03

C35 H45 21.30 -10.01

C35 H46 -10.20 -2.85

C47 H58 -25.00 -18.36

C54 H64 -19.70 -18.10

C56 H67 0.00 15.65

C56 H66 2.90 -2.03

C68 H79 -4.20 4.86

C75 H85 -1.20 0.57

C73 H83 -5.00 3.12

C71 H81 -5.30 3.53

C70 H80 -4.50 -1.79

C77 H88 -1.30 0.61

C77 H87 4.30 -2.17

Cornilescu Quality factor: 0.712724

Alignment tensor information:

A'x= 2.863e-04

A'y= 9.983e-04

A'z=-1.285e-03

Saupe tensor

S'x= 4.295e-04

S'y= 1.497e-03

S'z=-1.927e-03

Alignment tensor eigenvectors

e[x]=(-0.273, 0.489,-0.829)

e[y]=( 0.594, 0.763, 0.255)

e[z]=( 0.757,-0.423,-0.498)

Alignment tensor in laboratory coordinates:

[-3.617e-04,8.257e-04,7.002e-04]

[ 8.257e-04,4.195e-04,-1.930e-04]

[ 7.002e-04,-1.930e-04,-5.777e-05]

SVD condition number is 1.866e+01

Axial component Aa = -1.927e-03

Rhombic component Ar = -7.120e-04

Field=18.79 Teslas[ 2.27]

rhombicity R = 0.369

Asimmetry parameter etha =5.542e-01

GDO = 2.390e-03

ZY'Z'' Euler Angles (degrees)

Set 1

(-29.2,119.9,17.1)

Set 2

(150.8,-119.9,-162.9)

********Data set: #14

Computed data for frame #1

RDC Data:

I J Exp. [Hz] Comp. [Hz]

C5 H16 23.00 21.46

C12 H22 25.20 18.98

C14 H25 10.30 -0.93

C14 H24 3.60 -13.51

C26 H37 -19.00 -13.18

C33 H43 -23.00 -10.71

C31 H41 -21.90 -11.47

C29 H39 -23.90 -13.47

C28 H38 -19.50 -10.81

C35 H45 21.30 -10.45

C35 H46 -10.20 -3.57

C47 H58 -25.00 -16.72

C54 H64 -19.70 -17.59

C56 H67 0.00 15.53

C56 H66 2.90 -0.65

C68 H79 -4.20 4.04

C75 H85 -1.20 1.38

C73 H83 -5.00 3.50

C71 H81 -5.30 2.74

C70 H80 -4.50 -1.00

C77 H88 -1.30 2.48

C77 H87 4.30 -0.27

Cornilescu Quality factor: 0.69744

Alignment tensor information:

A'x=-6.086e-05

A'y=-8.495e-04

A'z= 9.104e-04

Saupe tensor

S'x=-9.129e-05

S'y=-1.274e-03

S'z= 1.366e-03

Alignment tensor eigenvectors

e[x]=( 0.147,-0.472, 0.869)

e[y]=( 0.796,-0.465,-0.387)

e[z]=( 0.587, 0.749, 0.307)

Alignment tensor in laboratory coordinates:

[-2.263e-04,7.190e-04,4.182e-04]

[ 7.190e-04,3.139e-04,8.140e-05]

[ 4.182e-04,8.140e-05,-8.762e-05]

SVD condition number is 1.866e+01

Axial component Aa = 1.366e-03

Rhombic component Ar = 7.887e-04

Field=18.79 Teslas[ 2.27]

rhombicity R = 0.578

Asimmetry parameter etha =8.663e-01

GDO = 1.849e-03

ZY'Z'' Euler Angles (degrees)

Set 1

(51.9,72.1,-156.0)

Set 2

(-128.1,-72.1,24.0)

********Data set: #15

Computed data for frame #1

RDC Data:

I J Exp. [Hz] Comp. [Hz]

C5 H16 23.00 13.80

C12 H22 25.20 10.55

C14 H24 10.30 -10.77

C14 H25 3.60 -7.63

C26 H37 -19.00 -17.93

C33 H43 -23.00 -17.33

C31 H41 -21.90 -16.27

C29 H39 -23.90 -19.14

C28 H38 -19.50 -19.87

C35 H46 21.30 9.87

C35 H45 -10.20 -18.59

C47 H58 -25.00 -22.57

C54 H64 -19.70 -20.39

C56 H67 0.00 14.01

C56 H66 2.90 -3.21

C68 H79 -4.20 1.45

C75 H85 -1.20 -1.88

C73 H83 -5.00 -0.79

C71 H81 -5.30 1.18

C70 H80 -4.50 -2.21

C77 H88 -1.30 0.22

C77 H87 4.30 -4.07

Cornilescu Quality factor: 0.544118

Alignment tensor information:

A'x= 2.030e-04

A'y= 8.405e-04

A'z=-1.044e-03

Saupe tensor

S'x= 3.045e-04

S'y= 1.261e-03

S'z=-1.565e-03

Alignment tensor eigenvectors

e[x]=( 0.528, 0.791, 0.310)

e[y]=( 0.295,-0.513, 0.806)

e[z]=( 0.796,-0.335,-0.504)

Alignment tensor in laboratory coordinates:

[-5.322e-04,2.358e-04,6.517e-04]

[ 2.358e-04,2.310e-04,-4.736e-04]

[ 6.517e-04,-4.736e-04,3.012e-04]

SVD condition number is 1.866e+01

Axial component Aa = -1.565e-03

Rhombic component Ar = -6.375e-04

Field=18.79 Teslas[ 2.27]

rhombicity R = 0.407

Asimmetry parameter etha =6.109e-01

GDO = 1.969e-03

ZY'Z'' Euler Angles (degrees)

Set 1

(-22.8,120.3,111.0)

Set 2

(157.2,-120.3,-69.0)

********Data set: #16

Computed data for frame #1

RDC Data:

I J Exp. [Hz] Comp. [Hz]

C5 H16 23.00 13.71

C12 H22 25.20 11.77

C14 H25 10.30 -3.82

C14 H24 3.60 -15.09

C26 H37 -19.00 -18.69

C33 H43 -23.00 -18.40

C31 H41 -21.90 -17.88

C29 H39 -23.90 -19.32

C28 H38 -19.50 -19.65

C35 H46 21.30 9.15

C35 H45 -10.20 -19.04

C47 H58 -25.00 -20.93

C54 H64 -19.70 -19.88

C56 H67 0.00 13.90

C56 H66 2.90 -1.83

C68 H79 -4.20 0.63

C75 H85 -1.20 -1.07

C73 H83 -5.00 -0.41

C71 H81 -5.30 0.38

C70 H80 -4.50 -1.42

C77 H88 -1.30 2.09

C77 H87 4.30 -2.18

Cornilescu Quality factor: 0.52709

Alignment tensor information:

A'x= 1.281e-04

A'y= 4.874e-04

A'z=-6.155e-04

Saupe tensor

S'x= 1.921e-04

S'y= 7.312e-04

S'z=-9.232e-04

Alignment tensor eigenvectors

e[x]=( 0.387, 0.890, 0.239)

e[y]=( 0.312,-0.370, 0.875)

e[z]=( 0.868,-0.264,-0.421)

Alignment tensor in laboratory coordinates:

[-3.968e-04,1.290e-04,3.697e-04]

[ 1.290e-04,1.254e-04,-1.992e-04]

[ 3.697e-04,-1.992e-04,2.713e-04]

SVD condition number is 1.866e+01

Axial component Aa = -9.232e-04

Rhombic component Ar = -3.594e-04

Field=18.79 Teslas[ 2.27]

rhombicity R = 0.389

Asimmetry parameter etha =5.839e-01

GDO = 1.153e-03

ZY'Z'' Euler Angles (degrees)

Set 1

(-16.9,114.9,105.3)

Set 2

(163.1,-114.9,-74.7)

********

MSpin-RDC pluginma. jul. 20 18:32:53 2021

(gg/gt)3

CR_tr

!* MSpin-RDC Plugin *!

********

!* Computation flags *!

Method: SVD

Scaling mode: Hz

Field (T): 18.7923

1H Larmor Frequency: 800.13

Scale QCSA with axial component: False

Include CSA gel shift (isotropic) correction:False

Optimize CSA gel shift (isotropic) correction scale:False

Estimate CSA gel shift (isotropic) correction scale:False

Gel Shift Correction Scale: 1

Single Tensor: False

Superimpose: False

Average methyl groups: False

Average methylene groups: False

Average phenyl groups: False

Bootstrapping: False

RDC Std. Error [ppm]: 1

CSA Std. Error [ppm]: 0.01

PCS Std. Error [ppm]: 0.01

DQ Std. Error [Hz]: 1

********

!* Permutations *!

There are no permutations on the original data set

********

Data set: #1

Computed data for frame #1

RDC Data:

I J Exp. [Hz] Comp. [Hz]

C5 H16 23.00 24.34

C12 H22 25.20 23.65

C26 H37 -19.00 -21.87

C33 H43 -23.00 -21.84

C31 H41 -21.90 -22.01

C29 H39 -23.90 -21.80

C28 H38 -19.50 -21.53

C47 H58 -25.00 -21.30

C54 H64 -19.70 -21.71

C68 H79 -4.20 -3.70

C75 H85 -1.20 -4.07

C73 H83 -5.00 -3.50

C71 H81 -5.30 -4.15

C70 H80 -4.50 -4.84

Cornilescu Quality factor: 0.106642

Alignment tensor information:

A'x= 7.409e-05

A'y= 3.298e-04

A'z=-4.039e-04

Saupe tensor

S'x= 1.111e-04

S'y= 4.947e-04

S'z=-6.058e-04

Alignment tensor eigenvectors

e[x]=( 0.142, 0.851,-0.505)

e[y]=( 0.139, 0.488, 0.862)

e[z]=( 0.980,-0.193,-0.049)

Alignment tensor in laboratory coordinates:

[-3.800e-04,1.077e-04,5.361e-05]

[ 1.077e-04,1.173e-04,1.030e-04]

[ 5.361e-05,1.030e-04,2.627e-04]

SVD condition number is 3.426e+01

Axial component Aa = -6.058e-04

Rhombic component Ar = -2.557e-04

Field=18.79 Teslas[ 2.27]

rhombicity R = 0.422

Asimmetry parameter etha =6.331e-01

GDO = 7.664e-04

ZY'Z'' Euler Angles (degrees)

Set 1

(-11.1,92.8,59.6)

Set 2

(168.9,-92.8,-120.4)

********

MSpin-RDC pluginma. jul. 20 18:40:28 2021

(gg/gt)3

E5C12

!* MSpin-RDC Plugin *!

********

!* Computation flags *!

Method: SVD

Scaling mode: Hz

Field (T): 18.7923

1H Larmor Frequency: 800.13

Scale QCSA with axial component: False

Include CSA gel shift (isotropic) correction:False

Optimize CSA gel shift (isotropic) correction scale:False

Estimate CSA gel shift (isotropic) correction scale:False

Gel Shift Correction Scale: 1

Single Tensor: False

Superimpose: False

Average methyl groups: False

Average methylene groups: False

Average phenyl groups: False

Bootstrapping: False

RDC Std. Error [ppm]: 1

CSA Std. Error [ppm]: 0.01

PCS Std. Error [ppm]: 0.01

DQ Std. Error [Hz]: 1

********

!* Permutations *!

There are no permutations on the original data set

********

Data set: #1

Computed data for frame #1

RDC Data:

I J Exp. [Hz] Comp. [Hz]

C5 H16 11.80 11.26

C12 H22 12.70 11.76

C26 H37 10.50 9.74

C33 H43 10.60 12.43

C47 H58 9.90 10.13

C54 H64 9.00 6.49

C68 H79 4.90 5.27

C75 H85 4.80 5.25

C71 H81 5.30 3.82

C70 H80 8.50 2.37

C77 H87 0.00 5.37

C77 H88 0.00 -1.70

Cornilescu Quality factor: 0.312912

Alignment tensor information:

A'x= 5.464e-05

A'y= 1.078e-03

A'z=-1.132e-03

Saupe tensor

S'x= 8.197e-05

S'y= 1.617e-03

S'z=-1.699e-03

Alignment tensor eigenvectors

e[x]=(-0.599, 0.445, 0.666)

e[y]=( 0.701, 0.694, 0.166)

e[z]=(-0.388, 0.566,-0.728)

Alignment tensor in laboratory coordinates:

[ 3.783e-04,7.581e-04,-2.163e-04]

[ 7.581e-04,1.672e-04,6.063e-04]

[-2.163e-04,6.063e-04,-5.455e-04]

SVD condition number is 2.109e+01

Axial component Aa = -1.699e-03

Rhombic component Ar = -1.023e-03

Field=18.79 Teslas[ 2.27]

rhombicity R = 0.602

Asimmetry parameter etha =9.035e-01

GDO = 2.327e-03

ZY'Z'' Euler Angles (degrees)

Set 1

(124.4,136.7,166.0)

Set 2

(-55.6,-136.7,-14.0)

********

MSpin-RDC pluginmi. jul. 21 11:01:58 2021

(gg/gt)3F

CRF_all

!* MSpin-RDC Plugin *!

********

!* Computation flags *!

Method: SVD

Scaling mode: Hz

Field (T): 18.7923

1H Larmor Frequency: 800.13

Scale QCSA with axial component: False

Include CSA gel shift (isotropic) correction:False

Optimize CSA gel shift (isotropic) correction scale:False

Estimate CSA gel shift (isotropic) correction scale:False

Gel Shift Correction Scale: 0.15

Single Tensor: False

Superimpose: False

Average methyl groups: False

Average methylene groups: False

Average phenyl groups: False

Bootstrapping: False

RDC Std. Error [ppm]: 1

CSA Std. Error [ppm]: 0.01

PCS Std. Error [ppm]: 0.01

DQ Std. Error [Hz]: 1

********

!* Permutations *!

There are 4 possible permutations on the original data set

********

Data set: #1

Computed data for frame #1

RDC Data:

I J Exp. [Hz] Comp. [Hz]

C5 H16 18.00 27.01

C10 H20 23.00 22.17

C10 F11 -13.00 -7.99

C25 H36 -19.00 -16.83

C32 H42 -23.00 -12.15

C30 H40 -21.90 -13.50

C28 H38 -23.90 -17.62

C27 H37 -19.50 -12.70

C34 H44 21.30 -12.24

C34 H45 -10.20 -3.62

C46 H57 -30.00 -24.78

C51 H61 -24.00 -21.25

C51 F52 13.00 13.01

C66 H77 -4.20 1.79

C73 H83 -1.20 -3.31

C71 H81 -5.00 0.52

C69 H79 -5.30 -0.46

C68 H78 -4.50 -7.28

C75 H85 -1.30 -6.26

C75 H86 4.30 7.60

Cornilescu Quality factor: 0.552318

Alignment tensor information:

A'x=-9.861e-05

A'y=-1.563e-03

A'z= 1.661e-03

Saupe tensor

S'x=-1.479e-04

S'y=-2.344e-03

S'z= 2.492e-03

Alignment tensor eigenvectors

e[x]=( 0.228,-0.500, 0.835)

e[y]=( 0.771,-0.431,-0.469)

e[z]=( 0.595, 0.751, 0.288)

Alignment tensor in laboratory coordinates:

[-3.469e-04,1.272e-03,8.299e-04]

[ 1.272e-03,6.214e-04,8.422e-05]

[ 8.299e-04,8.422e-05,-2.745e-04]

SVD condition number is 1.937e+01

Axial component Aa = 2.492e-03

Rhombic component Ar = 1.464e-03

Field=18.79 Teslas[ 2.27]

rhombicity R = 0.588

Asimmetry parameter etha =8.813e-01

GDO = 3.390e-03

ZY'Z'' Euler Angles (degrees)

Set 1

(51.6,73.3,-150.7)

Set 2

(-128.4,-73.3,29.3)

********Data set: #2

Computed data for frame #1

RDC Data:

I J Exp. [Hz] Comp. [Hz]

C5 H16 18.00 17.36

C10 H20 23.00 16.55

C10 F11 -13.00 -8.40

C25 H36 -19.00 -21.74

C32 H42 -23.00 -20.26

C30 H40 -21.90 -19.74

C28 H38 -23.90 -22.86

C27 H37 -19.50 -22.35

C34 H45 21.30 11.86

C34 H44 -10.20 -21.38

C46 H57 -30.00 -27.02

C51 H61 -24.00 -24.26

C51 F52 13.00 12.68

C66 H77 -4.20 -2.46

C73 H83 -1.20 -6.09

C71 H81 -5.00 -4.45

C69 H79 -5.30 -3.19

C68 H78 -4.50 -7.17

C75 H85 -1.30 -8.34

C75 H86 4.30 6.26

Cornilescu Quality factor: 0.268349

Alignment tensor information:

A'x= 5.032e-04

A'y= 6.812e-04

A'z=-1.184e-03

Saupe tensor

S'x= 7.548e-04

S'y= 1.022e-03

S'z=-1.777e-03

Alignment tensor eigenvectors

e[x]=( 0.471, 0.855, 0.218)

e[y]=( 0.350,-0.408, 0.843)

e[z]=( 0.809,-0.321,-0.492)

Alignment tensor in laboratory coordinates:

[-5.807e-04,4.134e-04,7.240e-04]

[ 4.134e-04,3.587e-04,-3.276e-04]

[ 7.240e-04,-3.276e-04,2.220e-04]

SVD condition number is 1.937e+01

Axial component Aa = -1.777e-03

Rhombic component Ar = -1.779e-04

Field=18.79 Teslas[ 2.27]

rhombicity R = 0.100

Asimmetry parameter etha =1.502e-01

GDO = 2.063e-03

ZY'Z'' Euler Angles (degrees)

Set 1

(-21.6,119.4,104.5)

Set 2

(158.4,-119.4,-75.5)

********Data set: #3

Computed data for frame #1

RDC Data:

I J Exp. [Hz] Comp. [Hz]

C5 H16 18.00 26.59

C10 H20 23.00 22.58

C10 F11 -13.00 -7.34

C25 H36 -19.00 -16.61

C32 H42 -23.00 -12.79

C30 H40 -21.90 -13.73

C28 H38 -23.90 -17.40

C27 H37 -19.50 -13.57

C34 H44 21.30 -13.06

C34 H45 -10.20 -4.57

C46 H57 -30.00 -23.57

C51 H61 -24.00 -20.41

C51 F52 13.00 13.27

C66 H77 -4.20 2.34

C73 H83 -1.20 -2.38

C71 H81 -5.00 0.97

C69 H79 -5.30 0.42

C68 H78 -4.50 -5.76

C75 H86 -1.30 3.23

C75 H85 4.30 -5.16

Cornilescu Quality factor: 0.571909

Alignment tensor information:

A'x= 3.927e-05

A'y= 1.393e-03

A'z=-1.432e-03

Saupe tensor

S'x= 5.891e-05

S'y= 2.090e-03

S'z=-2.148e-03

Alignment tensor eigenvectors

e[x]=(-0.227, 0.483,-0.846)

e[y]=( 0.587, 0.760, 0.277)

e[z]=( 0.777,-0.434,-0.456)

Alignment tensor in laboratory coordinates:

[-3.817e-04,1.101e-03,7.421e-04]

[ 1.101e-03,5.451e-04,-6.135e-06]

[ 7.421e-04,-6.135e-06,-1.634e-04]

SVD condition number is 1.937e+01

Axial component Aa = -2.148e-03

Rhombic component Ar = -1.354e-03

Field=18.79 Teslas[ 2.27]

rhombicity R = 0.630

Asimmetry parameter etha =9.452e-01

GDO = 2.984e-03

ZY'Z'' Euler Angles (degrees)

Set 1

(-29.2,117.2,18.1)

Set 2

(150.8,-117.2,-161.9)

********Data set: #4

Computed data for frame #1

RDC Data:

I J Exp. [Hz] Comp. [Hz]

C5 H16 18.00 16.95

C10 H20 23.00 16.96

C10 F11 -13.00 -7.75

C25 H36 -19.00 -21.52

C32 H42 -23.00 -20.90

C30 H40 -21.90 -19.97

C28 H38 -23.90 -22.64

C27 H37 -19.50 -23.22

C34 H45 21.30 10.91

C34 H44 -10.20 -22.20

C46 H57 -30.00 -25.81

C51 H61 -24.00 -23.41

C51 F52 13.00 12.94

C66 H77 -4.20 -1.91

C73 H83 -1.20 -5.17

C71 H81 -5.00 -4.00

C69 H79 -5.30 -2.32

C68 H78 -4.50 -5.64

C75 H86 -1.30 1.88

C75 H85 4.30 -7.24

Cornilescu Quality factor: 0.309021

Alignment tensor information:

A'x= 2.466e-04

A'y= 8.132e-04

A'z=-1.060e-03

Saupe tensor

S'x= 3.699e-04

S'y= 1.220e-03

S'z=-1.590e-03

Alignment tensor eigenvectors

e[x]=( 0.482, 0.805, 0.347)

e[y]=( 0.275,-0.515, 0.812)

e[z]=( 0.832,-0.296,-0.469)

Alignment tensor in laboratory coordinates:

[-6.155e-04,2.415e-04,6.363e-04]

[ 2.415e-04,2.824e-04,-4.180e-04]

[ 6.363e-04,-4.180e-04,3.331e-04]

SVD condition number is 1.937e+01

Axial component Aa = -1.590e-03

Rhombic component Ar = -5.666e-04

Field=18.79 Teslas[ 2.27]

rhombicity R = 0.356

Asimmetry parameter etha =5.346e-01

GDO = 1.962e-03

ZY'Z'' Euler Angles (degrees)

Set 1

(-19.6,118.0,113.1)

Set 2

(160.4,-118.0,-66.9)

********

MSpin-RDC pluginju. jul. 22 16:48:33 2021

(gg/gt)3F

CRF_tr

!* MSpin-RDC Plugin *!

********

!* Computation flags *!

Method: SVD

Scaling mode: Hz

Field (T): 18.7923

1H Larmor Frequency: 800.13

Scale QCSA with axial component: False

Include CSA gel shift (isotropic) correction:False

Optimize CSA gel shift (isotropic) correction scale:False

Estimate CSA gel shift (isotropic) correction scale:False

Gel Shift Correction Scale: 1

Single Tensor: False

Superimpose: False

Average methyl groups: False

Average methylene groups: False

Average phenyl groups: False

Bootstrapping: False

RDC Std. Error [ppm]: 1

CSA Std. Error [ppm]: 0.01

PCS Std. Error [ppm]: 0.01

DQ Std. Error [Hz]: 1

********

!* Permutations *!

There are no permutations on the original data set

********

Data set: #1

Computed data for frame #1

RDC Data:

I J Exp. [Hz] Comp. [Hz]

C5 H16 18.00 20.39

C10 H20 23.00 19.80

C10 F11 -13.00 -9.14

C25 H36 -19.00 -22.01

C32 H42 -23.00 -20.82

C30 H40 -21.90 -20.03

C28 H38 -23.90 -23.25

C27 H37 -19.50 -23.26

C46 H57 -30.00 -27.35

C51 H61 -24.00 -24.47

C51 F52 13.00 14.60

C66 H77 -4.20 -2.01

C73 H83 -1.20 -6.01

C71 H81 -5.00 -4.34

C69 H79 -5.30 -2.69

C68 H78 -4.50 -6.97

Cornilescu Quality factor: 0.149666

Alignment tensor information:

A'x= 4.476e-04

A'y= 8.121e-04

A'z=-1.260e-03

Saupe tensor

S'x= 6.714e-04

S'y= 1.218e-03

S'z=-1.890e-03

Alignment tensor eigenvectors

e[x]=( 0.528, 0.766, 0.367)

e[y]=( 0.247,-0.551, 0.797)

e[z]=( 0.813,-0.330,-0.480)

Alignment tensor in laboratory coordinates:

[-6.581e-04,4.079e-04,7.382e-04]

[ 4.079e-04,3.728e-04,-4.305e-04]

[ 7.382e-04,-4.305e-04,2.853e-04]

SVD condition number is 2.700e+01

Axial component Aa = -1.890e-03

Rhombic component Ar = -3.645e-04

Field=18.79 Teslas[ 2.27]

rhombicity R = 0.193

Asimmetry parameter etha =2.893e-01

GDO = 2.227e-03

ZY'Z'' Euler Angles (degrees)

Set 1

(-22.1,118.7,114.7)

Set 2

(157.9,-118.7,-65.3)

********

MSpin-RDC pluginmi. jul. 21 11:49:01 2021

(gt/gg)3

CR_all

!* MSpin-RDC Plugin *!

********

!* Computation flags *!

Method: SVD

Scaling mode: Hz

Field (T): 18.7923

1H Larmor Frequency: 800.13

Scale QCSA with axial component: False

Include CSA gel shift (isotropic) correction:False

Optimize CSA gel shift (isotropic) correction scale:False

Estimate CSA gel shift (isotropic) correction scale:False

Gel Shift Correction Scale: 1

Single Tensor: False

Superimpose: False

Average methyl groups: False

Average methylene groups: False

Average phenyl groups: False

Bootstrapping: False

RDC Std. Error [ppm]: 1

CSA Std. Error [ppm]: 0.01

PCS Std. Error [ppm]: 0.01

DQ Std. Error [Hz]: 1

********

!* Permutations *!

There are 16 possible permutations on the original data set

********

Data set: #1

Computed data for frame #1

RDC Data:

I J Exp. [Hz] Comp. [Hz]

C5 H16 23.00 -1.96

C12 H22 25.20 0.23

C14 H24 10.30 -1.15

C14 H25 3.60 -5.30

C26 H37 -19.00 -10.49

C33 H43 -23.00 -6.12

C31 H41 -21.90 -6.67

C29 H39 -23.90 -14.20

C28 H38 -19.50 -7.71

C35 H45 21.30 13.30

C35 H46 -10.20 -7.97

C47 H58 -25.00 2.97

C54 H64 -19.70 -4.08

C56 H66 0.00 -7.85

C56 H67 2.90 12.80

C68 H79 -4.20 -12.37

C75 H85 -1.20 -9.81

C73 H83 -5.00 -12.59

C71 H81 -5.30 -8.15

C70 H80 -4.50 -4.69

C77 H87 -1.30 -6.58

C77 H88 4.30 1.82

Cornilescu Quality factor: 0.844319

Alignment tensor information:

A'x= 7.977e-04

A'y= 1.321e-03

A'z=-2.119e-03

Saupe tensor

S'x= 1.197e-03

S'y= 1.982e-03

S'z=-3.179e-03

Alignment tensor eigenvectors

e[x]=(-0.403, 0.172, 0.899)

e[y]=( 0.089,-0.970, 0.226)

e[z]=( 0.911, 0.171, 0.376)

Alignment tensor in laboratory coordinates:

[-1.618e-03,-4.997e-04,-9.875e-04]

[-4.997e-04,1.205e-03,-3.023e-04]

[-9.875e-04,-3.023e-04,4.128e-04]

SVD condition number is 2.627e+01

Axial component Aa = -3.179e-03

Rhombic component Ar = -5.237e-04

Field=18.79 Teslas[ 2.27]

rhombicity R = 0.165

Asimmetry parameter etha =2.472e-01

GDO = 3.726e-03

ZY'Z'' Euler Angles (degrees)

Set 1

(10.6,67.9,165.9)

Set 2

(-169.4,-67.9,-14.1)

********Data set: #2

Computed data for frame #1

RDC Data:

I J Exp. [Hz] Comp. [Hz]

C5 H16 23.00 -3.20

C12 H22 25.20 -1.11

C14 H25 10.30 -1.75

C14 H24 3.60 -2.29

C26 H37 -19.00 -10.79

C33 H43 -23.00 -6.94

C31 H41 -21.90 -7.37

C29 H39 -23.90 -14.21

C28 H38 -19.50 -8.48

C35 H45 21.30 11.31

C35 H46 -10.20 -8.25

C47 H58 -25.00 2.01

C54 H64 -19.70 -4.22

C56 H66 0.00 -7.57

C56 H67 2.90 14.54

C68 H79 -4.20 -11.64

C75 H85 -1.20 -9.34

C73 H83 -5.00 -11.80

C71 H81 -5.30 -7.95

C70 H80 -4.50 -4.82

C77 H87 -1.30 -7.02

C77 H88 4.30 0.20

Cornilescu Quality factor: 0.847021

Alignment tensor information:

A'x= 7.256e-04

A'y= 1.179e-03

A'z=-1.904e-03

Saupe tensor

S'x= 1.088e-03

S'y= 1.768e-03

S'z=-2.856e-03

Alignment tensor eigenvectors

e[x]=(-0.416, 0.231, 0.880)

e[y]=( 0.068,-0.957, 0.284)

e[z]=( 0.907, 0.178, 0.382)

Alignment tensor in laboratory coordinates:

[-1.435e-03,-4.531e-04,-9.027e-04]

[-4.531e-04,1.057e-03,-3.014e-04]

[-9.027e-04,-3.014e-04,3.780e-04]

SVD condition number is 2.627e+01

Axial component Aa = -2.856e-03

Rhombic component Ar = -4.530e-04

Field=18.79 Teslas[ 2.27]

rhombicity R = 0.159

Asimmetry parameter etha =2.379e-01

GDO = 3.345e-03

ZY'Z'' Euler Angles (degrees)

Set 1

(11.1,67.5,162.1)

Set 2

(-168.9,-67.5,-17.9)

********Data set: #3

Computed data for frame #1

RDC Data:

I J Exp. [Hz] Comp. [Hz]

C5 H16 23.00 -0.55

C12 H22 25.20 -2.54

C14 H24 10.30 -5.97

C14 H25 3.60 -2.07

C26 H37 -19.00 -12.72

C33 H43 -23.00 -5.13

C31 H41 -21.90 -8.04

C29 H39 -23.90 -13.80

C28 H38 -19.50 -2.53

C35 H46 21.30 21.47

C35 H45 -10.20 -9.92

C47 H58 -25.00 -3.99

C54 H64 -19.70 -10.62

C56 H66 0.00 -12.25

C56 H67 2.90 8.56

C68 H79 -4.20 -8.61

C75 H85 -1.20 -10.40

C73 H83 -5.00 -11.79

C71 H81 -5.30 -3.18

C70 H80 -4.50 -6.12

C77 H87 -1.30 -6.93

C77 H88 4.30 3.85

Cornilescu Quality factor: 0.807895

Alignment tensor information:

A'x=-5.708e-04

A'y=-1.983e-03

A'z= 2.553e-03

Saupe tensor

S'x=-8.562e-04

S'y=-2.974e-03

S'z= 3.830e-03

Alignment tensor eigenvectors

e[x]=(-0.489, 0.045, 0.871)

e[y]=( 0.862, 0.179, 0.474)

e[z]=(-0.134, 0.983,-0.126)

Alignment tensor in laboratory coordinates:

[-1.563e-03,-6.297e-04,-5.246e-04]

[-6.297e-04,2.403e-03,-5.063e-04]

[-5.246e-04,-5.063e-04,-8.391e-04]

SVD condition number is 2.627e+01

Axial component Aa = 3.830e-03

Rhombic component Ar = 1.412e-03

Field=18.79 Teslas[ 2.27]

rhombicity R = 0.369

Asimmetry parameter etha =5.529e-01

GDO = 4.749e-03

ZY'Z'' Euler Angles (degrees)

Set 1

(97.8,97.2,151.4)

Set 2

(-82.2,-97.2,-28.6)

********Data set: #4

Computed data for frame #1

RDC Data:

I J Exp. [Hz] Comp. [Hz]

C5 H16 23.00 -1.78

C12 H22 25.20 -3.87

C14 H25 10.30 1.49

C14 H24 3.60 -7.11

C26 H37 -19.00 -13.02

C33 H43 -23.00 -5.95

C31 H41 -21.90 -8.74

C29 H39 -23.90 -13.82

C28 H38 -19.50 -3.31

C35 H46 21.30 21.19

C35 H45 -10.20 -11.91

C47 H58 -25.00 -4.96

C54 H64 -19.70 -10.77

C56 H66 0.00 -11.97

C56 H67 2.90 10.29

C68 H79 -4.20 -7.88

C75 H85 -1.20 -9.93

C73 H83 -5.00 -11.00

C71 H81 -5.30 -2.98

C70 H80 -4.50 -6.25

C77 H87 -1.30 -7.37

C77 H88 4.30 2.22

Cornilescu Quality factor: 0.798051

Alignment tensor information:

A'x=-6.259e-04

A'y=-1.778e-03

A'z= 2.404e-03

Saupe tensor

S'x=-9.389e-04

S'y=-2.667e-03

S'z= 3.605e-03

Alignment tensor eigenvectors

e[x]=(-0.527, 0.042, 0.849)

e[y]=( 0.839, 0.185, 0.512)

e[z]=(-0.136, 0.982,-0.133)

Alignment tensor in laboratory coordinates:

[-1.381e-03,-5.830e-04,-4.397e-04]

[-5.830e-04,2.254e-03,-5.054e-04]

[-4.397e-04,-5.054e-04,-8.739e-04]

SVD condition number is 2.627e+01

Axial component Aa = 3.605e-03

Rhombic component Ar = 1.152e-03

Field=18.79 Teslas[ 2.27]

rhombicity R = 0.319

Asimmetry parameter etha =4.792e-01

GDO = 4.396e-03

ZY'Z'' Euler Angles (degrees)

Set 1

(97.9,97.7,148.9)

Set 2

(-82.1,-97.7,-31.1)

********Data set: #5

Computed data for frame #1

RDC Data:

I J Exp. [Hz] Comp. [Hz]

C5 H16 23.00 -2.00

C12 H22 25.20 0.16

C14 H24 10.30 -1.14

C14 H25 3.60 -5.92

C26 H37 -19.00 -10.14

C33 H43 -23.00 -5.99

C31 H41 -21.90 -6.48

C29 H39 -23.90 -13.75

C28 H38 -19.50 -7.58

C35 H45 21.30 13.58

C35 H46 -10.20 -7.70

C47 H58 -25.00 3.24

C54 H64 -19.70 -3.59

C56 H67 0.00 11.65

C56 H66 2.90 -7.28

C68 H79 -4.20 -11.82

C75 H85 -1.20 -9.25

C73 H83 -5.00 -11.97

C71 H81 -5.30 -7.77

C70 H80 -4.50 -4.29

C77 H87 -1.30 -5.70

C77 H88 4.30 1.63

Cornilescu Quality factor: 0.857057

Alignment tensor information:

A'x= 7.942e-04

A'y= 1.250e-03

A'z=-2.044e-03

Saupe tensor

S'x= 1.191e-03

S'y= 1.875e-03

S'z=-3.066e-03

Alignment tensor eigenvectors

e[x]=(-0.402, 0.175, 0.899)

e[y]=( 0.086,-0.970, 0.227)

e[z]=( 0.912, 0.168, 0.375)

Alignment tensor in laboratory coordinates:

[-1.562e-03,-4.731e-04,-9.605e-04]

[-4.731e-04,1.143e-03,-2.793e-04]

[-9.605e-04,-2.793e-04,4.196e-04]

SVD condition number is 2.627e+01

Axial component Aa = -3.066e-03

Rhombic component Ar = -4.556e-04

Field=18.79 Teslas[ 2.27]

rhombicity R = 0.149

Asimmetry parameter etha =2.229e-01

GDO = 3.584e-03

ZY'Z'' Euler Angles (degrees)

Set 1

(10.5,68.0,165.8)

Set 2

(-169.5,-68.0,-14.2)

********Data set: #6

Computed data for frame #1

RDC Data:

I J Exp. [Hz] Comp. [Hz]

C5 H16 23.00 -3.24

C12 H22 25.20 -1.17

C14 H25 10.30 -2.36

C14 H24 3.60 -2.27

C26 H37 -19.00 -10.44

C33 H43 -23.00 -6.81

C31 H41 -21.90 -7.18

C29 H39 -23.90 -13.76

C28 H38 -19.50 -8.35

C35 H45 21.30 11.59

C35 H46 -10.20 -7.98

C47 H58 -25.00 2.27

C54 H64 -19.70 -3.73

C56 H67 0.00 13.38

C56 H66 2.90 -6.99

C68 H79 -4.20 -11.09

C75 H85 -1.20 -8.78

C73 H83 -5.00 -11.18

C71 H81 -5.30 -7.56

C70 H80 -4.50 -4.42

C77 H87 -1.30 -6.14

C77 H88 4.30 0.00

Cornilescu Quality factor: 0.860642

Alignment tensor information:

A'x= 7.216e-04

A'y= 1.107e-03

A'z=-1.829e-03

Saupe tensor

S'x= 1.082e-03

S'y= 1.661e-03

S'z=-2.744e-03

Alignment tensor eigenvectors

e[x]=(-0.415, 0.245, 0.876)

e[y]=( 0.060,-0.954, 0.295)

e[z]=( 0.908, 0.175, 0.381)

Alignment tensor in laboratory coordinates:

[-1.379e-03,-4.264e-04,-8.756e-04]

[-4.264e-04,9.946e-04,-2.784e-04]

[-8.756e-04,-2.784e-04,3.848e-04]

SVD condition number is 2.627e+01

Axial component Aa = -2.744e-03

Rhombic component Ar = -3.858e-04

Field=18.79 Teslas[ 2.27]

rhombicity R = 0.141

Asimmetry parameter etha =2.110e-01

GDO = 3.203e-03

ZY'Z'' Euler Angles (degrees)

Set 1

(10.9,67.6,161.4)

Set 2

(-169.1,-67.6,-18.6)

********Data set: #7

Computed data for frame #1

RDC Data:

I J Exp. [Hz] Comp. [Hz]

C5 H16 23.00 -0.58

C12 H22 25.20 -2.60

C14 H24 10.30 -5.96

C14 H25 3.60 -2.68

C26 H37 -19.00 -12.37

C33 H43 -23.00 -5.00

C31 H41 -21.90 -7.85

C29 H39 -23.90 -13.35

C28 H38 -19.50 -2.40

C35 H46 21.30 21.74

C35 H45 -10.20 -9.64

C47 H58 -25.00 -3.73

C54 H64 -19.70 -10.13

C56 H67 0.00 7.40

C56 H66 2.90 -11.68

C68 H79 -4.20 -8.06

C75 H85 -1.20 -9.84

C73 H83 -5.00 -11.17

C71 H81 -5.30 -2.80

C70 H80 -4.50 -5.71

C77 H87 -1.30 -6.05

C77 H88 4.30 3.65

Cornilescu Quality factor: 0.8213

Alignment tensor information:

A'x=-5.748e-04

A'y=-1.908e-03

A'z= 2.482e-03

Saupe tensor

S'x=-8.622e-04

S'y=-2.861e-03

S'z= 3.724e-03

Alignment tensor eigenvectors

e[x]=(-0.491, 0.043, 0.870)

e[y]=( 0.861, 0.177, 0.478)

e[z]=(-0.133, 0.983,-0.123)

Alignment tensor in laboratory coordinates:

[-1.508e-03,-6.030e-04,-4.976e-04]

[-6.030e-04,2.340e-03,-4.833e-04]

[-4.976e-04,-4.833e-04,-8.323e-04]

SVD condition number is 2.627e+01

Axial component Aa = 3.724e-03

Rhombic component Ar = 1.333e-03

Field=18.79 Teslas[ 2.27]

rhombicity R = 0.358

Asimmetry parameter etha =5.369e-01

GDO = 4.599e-03

ZY'Z'' Euler Angles (degrees)

Set 1

(97.7,97.1,151.2)

Set 2

(-82.3,-97.1,-28.8)

********Data set: #8

Computed data for frame #1

RDC Data:

I J Exp. [Hz] Comp. [Hz]

C5 H16 23.00 -1.82

C12 H22 25.20 -3.94

C14 H25 10.30 0.87

C14 H24 3.60 -7.10

C26 H37 -19.00 -12.67

C33 H43 -23.00 -5.82

C31 H41 -21.90 -8.55

C29 H39 -23.90 -13.36

C28 H38 -19.50 -3.17

C35 H46 21.30 21.46

C35 H45 -10.20 -11.63

C47 H58 -25.00 -4.69

C54 H64 -19.70 -10.27

C56 H67 0.00 9.13

C56 H66 2.90 -11.40

C68 H79 -4.20 -7.33

C75 H85 -1.20 -9.37

C73 H83 -5.00 -10.38

C71 H81 -5.30 -2.60

C70 H80 -4.50 -5.84

C77 H87 -1.30 -6.49

C77 H88 4.30 2.02

Cornilescu Quality factor: 0.812597

Alignment tensor information:

A'x=-6.293e-04

A'y=-1.703e-03

A'z= 2.332e-03

Saupe tensor

S'x=-9.440e-04

S'y=-2.555e-03

S'z= 3.499e-03

Alignment tensor eigenvectors

e[x]=(-0.533, 0.039, 0.845)

e[y]=( 0.835, 0.183, 0.518)

e[z]=(-0.135, 0.982,-0.131)

Alignment tensor in laboratory coordinates:

[-1.325e-03,-5.563e-04,-4.127e-04]

[-5.563e-04,2.192e-03,-4.824e-04]

[-4.127e-04,-4.824e-04,-8.671e-04]

SVD condition number is 2.627e+01

Axial component Aa = 3.499e-03

Rhombic component Ar = 1.074e-03

Field=18.79 Teslas[ 2.27]

rhombicity R = 0.307

Asimmetry parameter etha =4.604e-01

GDO = 4.249e-03

ZY'Z'' Euler Angles (degrees)

Set 1

(97.8,97.5,148.5)

Set 2

(-82.2,-97.5,-31.5)

********Data set: #9

Computed data for frame #1

RDC Data:

I J Exp. [Hz] Comp. [Hz]

C5 H16 23.00 -3.54

C12 H22 25.20 -1.09

C14 H24 10.30 -1.88

C14 H25 3.60 -5.04

C26 H37 -19.00 -9.92

C33 H43 -23.00 -6.88

C31 H41 -21.90 -6.94

C29 H39 -23.90 -13.37

C28 H38 -19.50 -8.84

C35 H45 21.30 13.60

C35 H46 -10.20 -8.09

C47 H58 -25.00 2.04

C54 H64 -19.70 -3.89

C56 H66 0.00 -7.40

C56 H67 2.90 11.18

C68 H79 -4.20 -12.55

C75 H85 -1.20 -9.68

C73 H83 -5.00 -12.23

C71 H81 -5.30 -9.27

C70 H80 -4.50 -5.29

C77 H88 -1.30 -0.55

C77 H87 4.30 -2.33

Cornilescu Quality factor: 0.850671

Alignment tensor information:

A'x= 8.514e-04

A'y= 9.338e-04

A'z=-1.785e-03

Saupe tensor

S'x= 1.277e-03

S'y= 1.401e-03

S'z=-2.678e-03

Alignment tensor eigenvectors

e[x]=(-0.380, 0.696, 0.609)

e[y]=(-0.164,-0.699, 0.696)

e[z]=( 0.910, 0.164, 0.380)

Alignment tensor in laboratory coordinates:

[-1.332e-03,-3.849e-04,-9.207e-04]

[-3.849e-04,8.204e-04,-2.045e-04]

[-9.207e-04,-2.045e-04,5.114e-04]

SVD condition number is 2.627e+01

Axial component Aa = -2.678e-03

Rhombic component Ar = -8.236e-05

Field=18.79 Teslas[ 2.27]

rhombicity R = 0.031

Asimmetry parameter etha =4.614e-02

GDO = 3.094e-03

ZY'Z'' Euler Angles (degrees)

Set 1

(10.2,67.7,131.2)

Set 2

(-169.8,-67.7,-48.8)

********Data set: #10

Computed data for frame #1

RDC Data:

I J Exp. [Hz] Comp. [Hz]

C5 H16 23.00 -4.78

C12 H22 25.20 -2.42

C14 H25 10.30 -1.48

C14 H24 3.60 -3.02

C26 H37 -19.00 -10.22

C33 H43 -23.00 -7.70

C31 H41 -21.90 -7.64

C29 H39 -23.90 -13.38

C28 H38 -19.50 -9.62

C35 H45 21.30 11.61

C35 H46 -10.20 -8.37

C47 H58 -25.00 1.08

C54 H64 -19.70 -4.03

C56 H66 0.00 -7.12

C56 H67 2.90 12.92

C68 H79 -4.20 -11.82

C75 H85 -1.20 -9.21

C73 H83 -5.00 -11.44

C71 H81 -5.30 -9.06

C70 H80 -4.50 -5.42

C77 H88 -1.30 -2.18

C77 H87 4.30 -2.77

Cornilescu Quality factor: 0.851874

Alignment tensor information:

A'x= 7.142e-04

A'y= 8.560e-04

A'z=-1.570e-03

Saupe tensor

S'x= 1.071e-03

S'y= 1.284e-03

S'z=-2.355e-03

Alignment tensor eigenvectors

e[x]=(-0.312, 0.889, 0.336)

e[y]=(-0.286,-0.425, 0.859)

e[z]=( 0.906, 0.172, 0.387)

Alignment tensor in laboratory coordinates:

[-1.149e-03,-3.383e-04,-8.359e-04]

[-3.383e-04,6.724e-04,-2.036e-04]

[-8.359e-04,-2.036e-04,4.765e-04]

SVD condition number is 2.627e+01

Axial component Aa = -2.355e-03

Rhombic component Ar = -1.417e-04

Field=18.79 Teslas[ 2.27]

rhombicity R = 0.060

Asimmetry parameter etha =9.026e-02

GDO = 2.725e-03

ZY'Z'' Euler Angles (degrees)

Set 1

(10.7,67.2,111.3)

Set 2

(-169.3,-67.2,-68.7)

********Data set: #11

Computed data for frame #1

RDC Data:

I J Exp. [Hz] Comp. [Hz]

C5 H16 23.00 -2.13

C12 H22 25.20 -3.85

C14 H24 10.30 -6.70

C14 H25 3.60 -1.81

C26 H37 -19.00 -12.15

C33 H43 -23.00 -5.88

C31 H41 -21.90 -8.31

C29 H39 -23.90 -12.97

C28 H38 -19.50 -3.67

C35 H46 21.30 21.35

C35 H45 -10.20 -9.62

C47 H58 -25.00 -4.92

C54 H64 -19.70 -10.44

C56 H66 0.00 -11.80

C56 H67 2.90 6.94

C68 H79 -4.20 -8.79

C75 H85 -1.20 -10.27

C73 H83 -5.00 -11.43

C71 H81 -5.30 -4.30

C70 H80 -4.50 -6.72

C77 H88 -1.30 1.47

C77 H87 4.30 -2.68

Cornilescu Quality factor: 0.817607

Alignment tensor information:

A'x=-4.816e-04

A'y=-1.655e-03

A'z= 2.136e-03

Saupe tensor

S'x=-7.224e-04

S'y=-2.482e-03

S'z= 3.205e-03

Alignment tensor eigenvectors

e[x]=(-0.515, 0.034, 0.857)

e[y]=( 0.847, 0.175, 0.502)

e[z]=(-0.132, 0.984,-0.119)

Alignment tensor in laboratory coordinates:

[-1.277e-03,-5.148e-04,-4.578e-04]

[-5.148e-04,2.018e-03,-4.085e-04]

[-4.578e-04,-4.085e-04,-7.405e-04]

SVD condition number is 2.627e+01

Axial component Aa = 3.205e-03

Rhombic component Ar = 1.173e-03

Field=18.79 Teslas[ 2.27]

rhombicity R = 0.366

Asimmetry parameter etha =5.491e-01

GDO = 3.969e-03

ZY'Z'' Euler Angles (degrees)

Set 1

(97.7,96.8,149.6)

Set 2

(-82.3,-96.8,-30.4)

********Data set: #12

Computed data for frame #1

RDC Data:

I J Exp. [Hz] Comp. [Hz]

C5 H16 23.00 -3.36

C12 H22 25.20 -5.18

C14 H25 10.30 1.75

C14 H24 3.60 -7.84

C26 H37 -19.00 -12.45

C33 H43 -23.00 -6.70

C31 H41 -21.90 -9.01

C29 H39 -23.90 -12.99

C28 H38 -19.50 -4.44

C35 H46 21.30 21.07

C35 H45 -10.20 -11.61

C47 H58 -25.00 -5.88

C54 H64 -19.70 -10.58

C56 H66 0.00 -11.52

C56 H67 2.90 8.67

C68 H79 -4.20 -8.06

C75 H85 -1.20 -9.80

C73 H83 -5.00 -10.64

C71 H81 -5.30 -4.09

C70 H80 -4.50 -6.85

C77 H88 -1.30 -0.16

C77 H87 4.30 -3.12

Cornilescu Quality factor: 0.806319

Alignment tensor information:

A'x=-5.316e-04

A'y=-1.455e-03

A'z= 1.986e-03

Saupe tensor

S'x=-7.974e-04

S'y=-2.182e-03

S'z= 2.979e-03

Alignment tensor eigenvectors

e[x]=(-0.569, 0.029, 0.822)

e[y]=( 0.812, 0.182, 0.555)

e[z]=(-0.134, 0.983,-0.127)

Alignment tensor in laboratory coordinates:

[-1.094e-03,-4.682e-04,-3.729e-04]

[-4.682e-04,1.870e-03,-4.076e-04]

[-3.729e-04,-4.076e-04,-7.753e-04]

SVD condition number is 2.627e+01

Axial component Aa = 2.979e-03

Rhombic component Ar = 9.230e-04

Field=18.79 Teslas[ 2.27]

rhombicity R = 0.310

Asimmetry parameter etha =4.647e-01

GDO = 3.621e-03

ZY'Z'' Euler Angles (degrees)

Set 1

(97.8,97.3,146.0)

Set 2

(-82.2,-97.3,-34.0)

********Data set: #13

Computed data for frame #1

RDC Data:

I J Exp. [Hz] Comp. [Hz]

C5 H16 23.00 -3.58

C12 H22 25.20 -1.15

C14 H24 10.30 -1.87

C14 H25 3.60 -5.65

C26 H37 -19.00 -9.57

C33 H43 -23.00 -6.75

C31 H41 -21.90 -6.76

C29 H39 -23.90 -12.92

C28 H38 -19.50 -8.71

C35 H45 21.30 13.88

C35 H46 -10.20 -7.82

C47 H58 -25.00 2.31

C54 H64 -19.70 -3.40

C56 H67 0.00 10.02

C56 H66 2.90 -6.83

C68 H79 -4.20 -12.00

C75 H85 -1.20 -9.13

C73 H83 -5.00 -11.61

C71 H81 -5.30 -8.88

C70 H80 -4.50 -4.89

C77 H88 -1.30 -0.75

C77 H87 4.30 -1.45

Cornilescu Quality factor: 0.861999

Alignment tensor information:

A'x= 8.107e-04

A'y= 8.996e-04

A'z=-1.710e-03

Saupe tensor

S'x= 1.216e-03

S'y= 1.349e-03

S'z=-2.565e-03

Alignment tensor eigenvectors

e[x]=(-0.280, 0.917, 0.286)

e[y]=(-0.301,-0.366, 0.880)

e[z]=( 0.912, 0.160, 0.379)

Alignment tensor in laboratory coordinates:

[-1.276e-03,-3.582e-04,-8.937e-04]

[-3.582e-04,7.580e-04,-1.815e-04]

[-8.937e-04,-1.815e-04,5.182e-04]

SVD condition number is 2.627e+01

Axial component Aa = -2.565e-03

Rhombic component Ar = -8.886e-05

Field=18.79 Teslas[ 2.27]

rhombicity R = 0.035

Asimmetry parameter etha =5.196e-02

GDO = 2.964e-03

ZY'Z'' Euler Angles (degrees)

Set 1

(10.0,67.8,108.0)

Set 2

(-170.0,-67.8,-72.0)

********Data set: #14

Computed data for frame #1

RDC Data:

I J Exp. [Hz] Comp. [Hz]

C5 H16 23.00 -4.82

C12 H22 25.20 -2.49

C14 H25 10.30 -2.10

C14 H24 3.60 -3.01

C26 H37 -19.00 -9.87

C33 H43 -23.00 -7.57

C31 H41 -21.90 -7.46

C29 H39 -23.90 -12.93

C28 H38 -19.50 -9.48

C35 H45 21.30 11.89

C35 H46 -10.20 -8.10

C47 H58 -25.00 1.35

C54 H64 -19.70 -3.54

C56 H67 0.00 11.76

C56 H66 2.90 -6.54

C68 H79 -4.20 -11.27

C75 H85 -1.20 -8.66

C73 H83 -5.00 -10.82

C71 H81 -5.30 -8.68

C70 H80 -4.50 -5.02

C77 H88 -1.30 -2.37

C77 H87 4.30 -1.89

Cornilescu Quality factor: 0.864106

Alignment tensor information:

A'x= 6.585e-04

A'y= 8.365e-04

A'z=-1.495e-03

Saupe tensor

S'x= 9.878e-04

S'y= 1.255e-03

S'z=-2.243e-03

Alignment tensor eigenvectors

e[x]=(-0.251, 0.952, 0.176)

e[y]=(-0.338,-0.257, 0.905)

e[z]=( 0.907, 0.167, 0.386)

Alignment tensor in laboratory coordinates:

[-1.093e-03,-3.116e-04,-8.088e-04]

[-3.116e-04,6.099e-04,-1.806e-04]

[-8.088e-04,-1.806e-04,4.834e-04]

SVD condition number is 2.627e+01

Axial component Aa = -2.243e-03

Rhombic component Ar = -1.780e-04

Field=18.79 Teslas[ 2.27]

rhombicity R = 0.079

Asimmetry parameter etha =1.191e-01

GDO = 2.599e-03

ZY'Z'' Euler Angles (degrees)

Set 1

(10.5,67.3,101.0)

Set 2

(-169.5,-67.3,-79.0)

********Data set: #15

Computed data for frame #1

RDC Data:

I J Exp. [Hz] Comp. [Hz]

C5 H16 23.00 -2.17

C12 H22 25.20 -3.91

C14 H24 10.30 -6.69

C14 H25 3.60 -2.42

C26 H37 -19.00 -11.80

C33 H43 -23.00 -5.75

C31 H41 -21.90 -8.13

C29 H39 -23.90 -12.52

C28 H38 -19.50 -3.53

C35 H46 21.30 21.62

C35 H45 -10.20 -9.33

C47 H58 -25.00 -4.65

C54 H64 -19.70 -9.94

C56 H67 0.00 5.78

C56 H66 2.90 -11.23

C68 H79 -4.20 -8.24

C75 H85 -1.20 -9.72

C73 H83 -5.00 -10.81

C71 H81 -5.30 -3.92

C70 H80 -4.50 -6.32

C77 H88 -1.30 1.28

C77 H87 4.30 -1.80

Cornilescu Quality factor: 0.82949

Alignment tensor information:

A'x=-4.853e-04

A'y=-1.580e-03

A'z= 2.066e-03

Saupe tensor

S'x=-7.280e-04

S'y=-2.370e-03

S'z= 3.098e-03

Alignment tensor eigenvectors

e[x]=(-0.520, 0.031, 0.854)

e[y]=( 0.844, 0.172, 0.508)

e[z]=(-0.131, 0.985,-0.115)

Alignment tensor in laboratory coordinates:

[-1.222e-03,-4.881e-04,-4.307e-04]

[-4.881e-04,1.955e-03,-3.855e-04]

[-4.307e-04,-3.855e-04,-7.337e-04]

SVD condition number is 2.627e+01

Axial component Aa = 3.098e-03

Rhombic component Ar = 1.095e-03

Field=18.79 Teslas[ 2.27]

rhombicity R = 0.353

Asimmetry parameter etha =5.301e-01

GDO = 3.821e-03

ZY'Z'' Euler Angles (degrees)

Set 1

(97.6,96.6,149.3)

Set 2

(-82.4,-96.6,-30.7)

********Data set: #16

Computed data for frame #1

RDC Data:

I J Exp. [Hz] Comp. [Hz]

C5 H16 23.00 -3.40

C12 H22 25.20 -5.25

C14 H25 10.30 1.14

C14 H24 3.60 -7.83

C26 H37 -19.00 -12.10

C33 H43 -23.00 -6.57

C31 H41 -21.90 -8.83

C29 H39 -23.90 -12.53

C28 H38 -19.50 -4.31

C35 H46 21.30 21.34

C35 H45 -10.20 -11.33

C47 H58 -25.00 -5.62

C54 H64 -19.70 -10.08

C56 H67 0.00 7.51

C56 H66 2.90 -10.95

C68 H79 -4.20 -7.51

C75 H85 -1.20 -9.24

C73 H83 -5.00 -10.02

C71 H81 -5.30 -3.71

C70 H80 -4.50 -6.45

C77 H88 -1.30 -0.35

C77 H87 4.30 -2.24

Cornilescu Quality factor: 0.819335

Alignment tensor information:

A'x=-5.340e-04

A'y=-1.381e-03

A'z= 1.915e-03

Saupe tensor

S'x=-8.010e-04

S'y=-2.072e-03

S'z= 2.873e-03

Alignment tensor eigenvectors

e[x]=(-0.580, 0.025, 0.815)

e[y]=( 0.804, 0.180, 0.567)

e[z]=(-0.132, 0.983,-0.124)

Alignment tensor in laboratory coordinates:

[-1.039e-03,-4.415e-04,-3.459e-04]

[-4.415e-04,1.807e-03,-3.846e-04]

[-3.459e-04,-3.846e-04,-7.685e-04]

SVD condition number is 2.627e+01

Axial component Aa = 2.873e-03

Rhombic component Ar = 8.473e-04

Field=18.79 Teslas[ 2.27]

rhombicity R = 0.295

Asimmetry parameter etha =4.424e-01

GDO = 3.476e-03

ZY'Z'' Euler Angles (degrees)

Set 1

(97.7,97.1,145.2)

Set 2

(-82.3,-97.1,-34.8)

********

MSpin-RDC pluginmi. jul. 21 12:05:04 2021

(gt/gg)3

CR_tr

!* MSpin-RDC Plugin *!

********

!* Computation flags *!

Method: SVD

Scaling mode: Hz

Field (T): 18.7923

1H Larmor Frequency: 800.13

Scale QCSA with axial component: False

Include CSA gel shift (isotropic) correction:False

Optimize CSA gel shift (isotropic) correction scale:False

Estimate CSA gel shift (isotropic) correction scale:False

Gel Shift Correction Scale: 1

Single Tensor: False

Superimpose: False

Average methyl groups: False

Average methylene groups: False

Average phenyl groups: False

Bootstrapping: False

RDC Std. Error [ppm]: 1

CSA Std. Error [ppm]: 0.01

PCS Std. Error [ppm]: 0.01

DQ Std. Error [Hz]: 1

********

!* Permutations *!

There are no permutations on the original data set

********

Data set: #1

Computed data for frame #1

RDC Data:

I J Exp. [Hz] Comp. [Hz]

C5 H16 23.00 5.71

C12 H22 25.20 20.34

C26 H37 -19.00 -17.53

C33 H43 -23.00 -7.65

C31 H41 -21.90 -5.56

C29 H39 -23.90 -35.12

C28 H38 -19.50 -19.52

C47 H58 -25.00 -20.55

C54 H64 -19.70 -20.07

C68 H79 -4.20 -0.74

C75 H85 -1.20 -8.33

C73 H83 -5.00 -5.51

C71 H81 -5.30 0.68

C70 H80 -4.50 -10.15

Cornilescu Quality factor: 0.490439

Alignment tensor information:

A'x=-4.203e-04

A'y=-6.492e-03

A'z= 6.912e-03

Saupe tensor

S'x=-6.305e-04

S'y=-9.738e-03

S'z= 1.037e-02

Alignment tensor eigenvectors

e[x]=(-0.793, 0.314, 0.522)

e[y]=( 0.582, 0.647, 0.493)

e[z]=(-0.183, 0.695,-0.696)

Alignment tensor in laboratory coordinates:

[-2.229e-03,-3.216e-03,-8.085e-04]

[-3.216e-03,5.763e-04,-5.481e-03]

[-8.085e-04,-5.481e-03,1.652e-03]

SVD condition number is 5.517e+01

Axial component Aa = 1.037e-02

Rhombic component Ar = 6.071e-03

Field=18.79 Teslas[ 2.27]

rhombicity R = 0.586

Asimmetry parameter etha =8.784e-01

GDO = 1.409e-02

ZY'Z'' Euler Angles (degrees)

Set 1

(104.7,134.1,136.6)

Set 2

(-75.3,-134.1,-43.4)

********

MSpin-RDC pluginmi. jul. 21 12:23:22 2021

(gt/gg)3

E5C12

!* MSpin-RDC Plugin *!

********

!* Computation flags *!

Method: SVD

Scaling mode: Hz

Field (T): 18.7923

1H Larmor Frequency: 800.13

Scale QCSA with axial component: False

Include CSA gel shift (isotropic) correction:False

Optimize CSA gel shift (isotropic) correction scale:False

Estimate CSA gel shift (isotropic) correction scale:False

Gel Shift Correction Scale: 1

Single Tensor: False

Superimpose: False

Average methyl groups: False

Average methylene groups: False

Average phenyl groups: False

Bootstrapping: False

RDC Std. Error [ppm]: 1

CSA Std. Error [ppm]: 0.01

PCS Std. Error [ppm]: 0.01

DQ Std. Error [Hz]: 1

********

!* Permutations *!

There are no permutations on the original data set

********

Data set: #1

Computed data for frame #1

RDC Data:

I J Exp. [Hz] Comp. [Hz]

C5 H16 11.80 7.89

C12 H22 12.70 9.95

C26 H37 10.50 12.04

C33 H43 10.60 9.10

C47 H58 9.90 9.18

C54 H64 9.00 10.20

C68 H79 4.90 4.99

C75 H85 4.80 7.38

C71 H81 5.30 3.54

C70 H80 8.50 7.03

C77 H87 0.00 0.01

C77 H88 0.00 6.69

Cornilescu Quality factor: 0.318507

Alignment tensor information:

A'x= 2.700e-04

A'y= 8.720e-04

A'z=-1.142e-03

Saupe tensor

S'x= 4.050e-04

S'y= 1.308e-03

S'z=-1.713e-03

Alignment tensor eigenvectors

e[x]=(-0.987,-0.132, 0.089)

e[y]=( 0.068, 0.158, 0.985)

e[z]=(-0.144, 0.979,-0.147)

Alignment tensor in laboratory coordinates:

[ 2.436e-04,2.050e-04,1.033e-05]

[ 2.050e-04,-1.067e-03,2.976e-04]

[ 1.033e-05,2.976e-04,8.234e-04]

SVD condition number is 4.524e+01

Axial component Aa = -1.713e-03

Rhombic component Ar = -6.020e-04

Field=18.79 Teslas[ 2.27]

rhombicity R = 0.351

Asimmetry parameter etha =5.272e-01

GDO = 2.111e-03

ZY'Z'' Euler Angles (degrees)

Set 1

(98.4,98.5,95.2)

Set 2

(-81.6,-98.5,-84.8)

********

MSpin-RDC pluginmi. jul. 21 12:39:48 2021

(gt/gg)3F

CRF_all

!* MSpin-RDC Plugin *!

********

!* Computation flags *!

Method: SVD

Scaling mode: Hz

Field (T): 18.7923

1H Larmor Frequency: 800.13

Scale QCSA with axial component: False

Include CSA gel shift (isotropic) correction:False

Optimize CSA gel shift (isotropic) correction scale:False

Estimate CSA gel shift (isotropic) correction scale:False

Gel Shift Correction Scale: 0.15

Single Tensor: False

Superimpose: False

Average methyl groups: False

Average methylene groups: False

Average phenyl groups: False

Bootstrapping: False

RDC Std. Error [ppm]: 1

CSA Std. Error [ppm]: 0.01

PCS Std. Error [ppm]: 0.01

DQ Std. Error [Hz]: 1

********

!* Permutations *!

There are 4 possible permutations on the original data set

********

Data set: #1

Computed data for frame #1

RDC Data:

I J Exp. [Hz] Comp. [Hz]

C5 H16 18.00 -2.67

C10 H20 23.00 4.18

C10 F11 -13.00 -5.08

C25 H36 -19.00 -11.08

C32 H42 -23.00 -7.83

C30 H40 -21.90 -8.10

C28 H38 -23.90 -14.55

C27 H37 -19.50 -9.61

C34 H44 21.30 12.93

C34 H45 -10.20 -10.31

C46 H57 -30.00 -1.95

C51 H61 -24.00 -5.43

C51 F52 13.00 -10.84

C66 H77 -4.20 -14.34

C73 H83 -1.20 -12.27

C71 H81 -5.00 -14.38

C69 H79 -5.30 -11.43

C68 H78 -4.50 -8.45

C75 H85 -1.30 -8.91

C75 H86 4.30 -3.71

Cornilescu Quality factor: 0.816455

Alignment tensor information:

A'x= 6.830e-04

A'y= 1.017e-03

A'z=-1.700e-03

Saupe tensor

S'x= 1.024e-03

S'y= 1.525e-03

S'z=-2.550e-03

Alignment tensor eigenvectors

e[x]=(-0.446, 0.441, 0.779)

e[y]=(-0.030,-0.877, 0.480)

e[z]=( 0.894, 0.191, 0.405)

Alignment tensor in laboratory coordinates:

[-1.223e-03,-3.986e-04,-8.670e-04]

[-3.986e-04,8.528e-04,-3.247e-04]

[-8.670e-04,-3.247e-04,3.697e-04]

SVD condition number is 2.170e+01

Axial component Aa = -2.550e-03

Rhombic component Ar = -3.339e-04

Field=18.79 Teslas[ 2.27]

rhombicity R = 0.131

Asimmetry parameter etha =1.964e-01

GDO = 2.973e-03

ZY'Z'' Euler Angles (degrees)

Set 1

(12.1,66.1,148.4)

Set 2

(-167.9,-66.1,-31.6)

********Data set: #2

Computed data for frame #1

RDC Data:

I J Exp. [Hz] Comp. [Hz]

C5 H16 18.00 -5.47

C10 H20 23.00 0.34

C10 F11 -13.00 1.26

C25 H36 -19.00 -11.94

C32 H42 -23.00 -6.68

C30 H40 -21.90 -8.69

C28 H38 -23.90 -13.35

C27 H37 -19.50 -5.23

C34 H45 21.30 19.18

C34 H44 -10.20 -10.45

C46 H57 -30.00 -7.96

C51 H61 -24.00 -14.73

C51 F52 13.00 -8.72

C66 H77 -4.20 -11.11

C73 H83 -1.20 -12.51

C71 H81 -5.00 -13.39

C69 H79 -5.30 -7.40

C68 H78 -4.50 -9.56

C75 H85 -1.30 -11.61

C75 H86 4.30 -3.85

Cornilescu Quality factor: 0.794365

Alignment tensor information:

A'x=-3.164e-04

A'y=-1.579e-03

A'z= 1.895e-03

Saupe tensor

S'x=-4.746e-04

S'y=-2.368e-03

S'z= 2.843e-03

Alignment tensor eigenvectors

e[x]=(-0.557, 0.052, 0.829)

e[y]=( 0.819, 0.200, 0.538)

e[z]=(-0.138, 0.978,-0.155)

Alignment tensor in laboratory coordinates:

[-1.121e-03,-5.056e-04,-5.089e-04]

[-5.056e-04,1.750e-03,-4.704e-04]

[-5.089e-04,-4.704e-04,-6.287e-04]

SVD condition number is 2.170e+01

Axial component Aa = 2.843e-03

Rhombic component Ar = 1.263e-03

Field=18.79 Teslas[ 2.27]

rhombicity R = 0.444

Asimmetry parameter etha =6.661e-01

GDO = 3.629e-03

ZY'Z'' Euler Angles (degrees)

Set 1

(98.0,98.9,147.0)

Set 2

(-82.0,-98.9,-33.0)

********Data set: #3

Computed data for frame #1

RDC Data:

I J Exp. [Hz] Comp. [Hz]

C5 H16 18.00 -3.63

C10 H20 23.00 2.30

C10 F11 -13.00 -3.90

C25 H36 -19.00 -11.00

C32 H42 -23.00 -8.59

C30 H40 -21.90 -8.55

C28 H38 -23.90 -14.24

C27 H37 -19.50 -10.60

C34 H44 21.30 13.56

C34 H45 -10.20 -10.14

C46 H57 -30.00 -2.36

C51 H61 -24.00 -4.77

C51 F52 13.00 -10.25

C66 H77 -4.20 -14.49

C73 H83 -1.20 -12.15

C71 H81 -5.00 -14.15

C69 H79 -5.30 -12.15

C68 H78 -4.50 -8.76

C75 H86 -1.30 -4.94

C75 H85 4.30 -4.27

Cornilescu Quality factor: 0.819194

Alignment tensor information:

A'x= 6.283e-04

A'y= 8.520e-04

A'z=-1.480e-03

Saupe tensor

S'x= 9.425e-04

S'y= 1.278e-03

S'z=-2.221e-03

Alignment tensor eigenvectors

e[x]=(-0.340, 0.871, 0.354)

e[y]=(-0.290,-0.455, 0.842)

e[z]=( 0.895, 0.183, 0.407)

Alignment tensor in laboratory coordinates:

[-1.041e-03,-3.160e-04,-8.234e-04]

[-3.160e-04,6.039e-04,-2.431e-04]

[-8.234e-04,-2.431e-04,4.368e-04]

SVD condition number is 2.170e+01

Axial component Aa = -2.221e-03

Rhombic component Ar = -2.237e-04

Field=18.79 Teslas[ 2.27]

rhombicity R = 0.101

Asimmetry parameter etha =1.511e-01

GDO = 2.579e-03

ZY'Z'' Euler Angles (degrees)

Set 1

(11.6,66.0,112.8)

Set 2

(-168.4,-66.0,-67.2)

********Data set: #4

Computed data for frame #1

RDC Data:

I J Exp. [Hz] Comp. [Hz]

C5 H16 18.00 -6.43

C10 H20 23.00 -1.54

C10 F11 -13.00 2.44

C25 H36 -19.00 -11.86

C32 H42 -23.00 -7.45

C30 H40 -21.90 -9.13

C28 H38 -23.90 -13.04

C27 H37 -19.50 -6.21

C34 H45 21.30 19.35

C34 H44 -10.20 -9.82

C46 H57 -30.00 -8.37

C51 H61 -24.00 -14.07

C51 F52 13.00 -8.13

C66 H77 -4.20 -11.25

C73 H83 -1.20 -12.39

C71 H81 -5.00 -13.17

C69 H79 -5.30 -8.12

C68 H78 -4.50 -9.86

C75 H86 -1.30 -5.08

C75 H85 4.30 -6.97

Cornilescu Quality factor: 0.800351

Alignment tensor information:

A'x=-2.520e-04

A'y=-1.365e-03

A'z= 1.617e-03

Saupe tensor

S'x=-3.779e-04

S'y=-2.048e-03

S'z= 2.426e-03

Alignment tensor eigenvectors

e[x]=(-0.577, 0.041, 0.815)

e[y]=( 0.805, 0.195, 0.560)

e[z]=(-0.136, 0.980,-0.146)

Alignment tensor in laboratory coordinates:

[-9.393e-04,-4.230e-04,-4.653e-04]

[-4.230e-04,1.501e-03,-3.888e-04]

[-4.653e-04,-3.888e-04,-5.617e-04]

SVD condition number is 2.170e+01

Axial component Aa = 2.426e-03

Rhombic component Ar = 1.113e-03

Field=18.79 Teslas[ 2.27]

rhombicity R = 0.459

Asimmetry parameter etha =6.884e-01

GDO = 3.116e-03

ZY'Z'' Euler Angles (degrees)

Set 1

(97.9,98.4,145.5)

Set 2

(-82.1,-98.4,-34.5)

********

MSpin-RDC pluginju. jul. 22 16:51:16 2021

(gt/gg)3F

CRF_tr

!* MSpin-RDC Plugin *!

********

!* Computation flags *!

Method: SVD

Scaling mode: Hz

Field (T): 18.7923

1H Larmor Frequency: 800.13

Scale QCSA with axial component: False

Include CSA gel shift (isotropic) correction:False

Optimize CSA gel shift (isotropic) correction scale:False

Estimate CSA gel shift (isotropic) correction scale:False

Gel Shift Correction Scale: 0.15

Single Tensor: False

Superimpose: False

Average methyl groups: False

Average methylene groups: False

Average phenyl groups: False

Bootstrapping: False

RDC Std. Error [ppm]: 1

CSA Std. Error [ppm]: 0.01

PCS Std. Error [ppm]: 0.01

DQ Std. Error [Hz]: 1

********

!* Permutations *!

There are no permutations on the original data set

********

Data set: #1

Computed data for frame #1

RDC Data:

I J Exp. [Hz] Comp. [Hz]

C5 H16 18.00 -5.07

C10 H20 23.00 2.87

C10 F11 -13.00 -22.01

C25 H36 -19.00 -14.32

C32 H42 -23.00 -7.69

C30 H40 -21.90 -9.97

C28 H38 -23.90 -16.79

C27 H37 -19.50 -6.43

C46 H57 -30.00 -14.17

C51 H61 -24.00 -22.44

C51 F52 13.00 -11.75

C66 H77 -4.20 -6.32

C73 H83 -1.20 -10.89

C71 H81 -5.00 -10.11

C69 H79 -5.30 -3.21

C68 H78 -4.50 -9.95

Cornilescu Quality factor: 0.723906

Alignment tensor information:

A'x=-7.110e-04

A'y=-1.920e-03

A'z= 2.631e-03

Saupe tensor

S'x=-1.067e-03

S'y=-2.880e-03

S'z= 3.947e-03

Alignment tensor eigenvectors

e[x]=(-0.764, 0.060, 0.643)

e[y]=( 0.610, 0.391, 0.689)

e[z]=(-0.210, 0.918,-0.335)

Alignment tensor in laboratory coordinates:

[-1.014e-03,-9.342e-04,-2.728e-04]

[-9.342e-04,1.923e-03,-1.354e-03]

[-2.728e-04,-1.354e-03,-9.096e-04]

SVD condition number is 3.945e+01

Axial component Aa = 3.947e-03

Rhombic component Ar = 1.209e-03

Field=18.79 Teslas[ 2.27]

rhombicity R = 0.306

Asimmetry parameter etha =4.595e-01

GDO = 4.792e-03

ZY'Z'' Euler Angles (degrees)

Set 1

(102.9,109.6,133.0)

Set 2

(-77.1,-109.6,-47.0)

********

MSpin-RDC pluginmi. jul. 21 14:20:09 2021

gg2/gt2/gg2

CR_all

!* MSpin-RDC Plugin *!

********

!* Computation flags *!

Method: SVD

Scaling mode: Hz

Field (T): 18.7923

1H Larmor Frequency: 800.13

Scale QCSA with axial component: False

Include CSA gel shift (isotropic) correction:False

Optimize CSA gel shift (isotropic) correction scale:False

Estimate CSA gel shift (isotropic) correction scale:False

Gel Shift Correction Scale: 0.15

Single Tensor: False

Superimpose: False

Average methyl groups: False

Average methylene groups: False

Average phenyl groups: False

Bootstrapping: False

RDC Std. Error [ppm]: 1

CSA Std. Error [ppm]: 0.01

PCS Std. Error [ppm]: 0.01

DQ Std. Error [Hz]: 1

********

!* Permutations *!

There are 16 possible permutations on the original data set

********

Data set: #1

Computed data for frame #1

RDC Data:

I J Exp. [Hz] Comp. [Hz]

C5 H16 23.00 19.99

C12 H22 25.20 14.78

C14 H24 10.30 -10.55

C14 H25 3.60 -1.05

C26 H37 -19.00 -16.33

C33 H43 -23.00 -15.91

C31 H41 -21.90 -14.48

C29 H39 -23.90 -20.08

C28 H38 -19.50 -19.20

C35 H45 21.30 7.50

C35 H46 -10.20 -0.36

C47 H58 -25.00 3.17

C54 H64 -19.70 -2.32

C56 H66 0.00 -6.44

C56 H67 2.90 11.50

C68 H79 -4.20 -10.89

C75 H85 -1.20 -6.28

C73 H83 -5.00 -9.66

C71 H81 -5.30 -9.90

C70 H80 -4.50 -3.15

C77 H87 -1.30 -4.30

C77 H88 4.30 9.49

Cornilescu Quality factor: 0.666243

Alignment tensor information:

A'x= 2.307e-04

A'y= 1.405e-03

A'z=-1.636e-03

Saupe tensor

S'x= 3.461e-04

S'y= 2.108e-03

S'z=-2.454e-03

Alignment tensor eigenvectors

e[x]=( 0.578, 0.727, 0.371)

e[y]=( 0.338,-0.627, 0.701)

e[z]=( 0.743,-0.279,-0.608)

Alignment tensor in laboratory coordinates:

[-6.648e-04,1.380e-04,1.122e-03]

[ 1.380e-04,5.472e-04,-8.340e-04]

[ 1.122e-03,-8.340e-04,1.176e-04]

SVD condition number is 2.807e+01

Axial component Aa = -2.454e-03

Rhombic component Ar = -1.174e-03

Field=18.79 Teslas[ 2.27]

rhombicity R = 0.479

Asimmetry parameter etha =7.179e-01

GDO = 3.178e-03

ZY'Z'' Euler Angles (degrees)

Set 1

(-20.6,127.5,117.9)

Set 2

(159.4,-127.5,-62.1)

********Data set: #2

Computed data for frame #1

RDC Data:

I J Exp. [Hz] Comp. [Hz]

C5 H16 23.00 19.34

C12 H22 25.20 14.44

C14 H25 10.30 2.84

C14 H24 3.60 -12.76

C26 H37 -19.00 -18.00

C33 H43 -23.00 -17.10

C31 H41 -21.90 -16.08

C29 H39 -23.90 -21.28

C28 H38 -19.50 -19.74

C35 H45 21.30 5.36

C35 H46 -10.20 0.78

C47 H58 -25.00 2.22

C54 H64 -19.70 -2.80

C56 H66 0.00 -6.53

C56 H67 2.90 12.60

C68 H79 -4.20 -9.92

C75 H85 -1.20 -6.20

C73 H83 -5.00 -9.19

C71 H81 -5.30 -8.91

C70 H80 -4.50 -3.19

C77 H87 -1.30 -4.45

C77 H88 4.30 8.29

Cornilescu Quality factor: 0.641973

Alignment tensor information:

A'x= 3.871e-04

A'y= 1.140e-03

A'z=-1.527e-03

Saupe tensor

S'x= 5.806e-04

S'y= 1.710e-03

S'z=-2.291e-03

Alignment tensor eigenvectors

e[x]=( 0.569, 0.752, 0.332)

e[y]=( 0.354,-0.589, 0.726)

e[z]=( 0.742,-0.296,-0.602)

Alignment tensor in laboratory coordinates:

[-5.720e-04,2.632e-04,1.048e-03]

[ 2.632e-04,4.805e-04,-6.629e-04]

[ 1.048e-03,-6.629e-04,9.148e-05]

SVD condition number is 2.807e+01

Axial component Aa = -2.291e-03

Rhombic component Ar = -7.529e-04

Field=18.79 Teslas[ 2.27]

rhombicity R = 0.329

Asimmetry parameter etha =4.930e-01

GDO = 2.801e-03

ZY'Z'' Euler Angles (degrees)

Set 1

(-21.7,127.0,114.6)

Set 2

(158.3,-127.0,-65.4)

********Data set: #3

Computed data for frame #1

RDC Data:

I J Exp. [Hz] Comp. [Hz]

C5 H16 23.00 21.10

C12 H22 25.20 20.03

C14 H24 10.30 -16.76

C14 H25 3.60 8.14

C26 H37 -19.00 -16.78

C33 H43 -23.00 -15.09

C31 H41 -21.90 -15.97

C29 H39 -23.90 -16.42

C28 H38 -19.50 -13.97

C35 H46 21.30 18.73

C35 H45 -10.20 -8.56

C47 H58 -25.00 -6.74

C54 H64 -19.70 -7.75

C56 H66 0.00 -8.37

C56 H67 2.90 2.83

C68 H79 -4.20 -6.88

C75 H85 -1.20 -7.72

C73 H83 -5.00 -8.01

C71 H81 -5.30 -6.37

C70 H80 -4.50 -6.67

C77 H87 -1.30 -7.57

C77 H88 4.30 14.62

Cornilescu Quality factor: 0.572973

Alignment tensor information:

A'x=-1.089e-04

A'y=-5.980e-04

A'z= 7.069e-04

Saupe tensor

S'x=-1.633e-04

S'y=-8.971e-04

S'z= 1.060e-03

Alignment tensor eigenvectors

e[x]=(-0.318,-0.283, 0.905)

e[y]=( 0.769,-0.636, 0.071)

e[z]=( 0.555, 0.718, 0.420)

Alignment tensor in laboratory coordinates:

[-1.467e-04,5.642e-04,1.632e-04]

[ 5.642e-04,1.143e-04,2.681e-04]

[ 1.632e-04,2.681e-04,3.234e-05]

SVD condition number is 2.807e+01

Axial component Aa = 1.060e-03

Rhombic component Ar = 4.892e-04

Field=18.79 Teslas[ 2.27]

rhombicity R = 0.461

Asimmetry parameter etha =6.919e-01

GDO = 1.363e-03

ZY'Z'' Euler Angles (degrees)

Set 1

(52.3,65.2,175.5)

Set 2

(-127.7,-65.2,-4.5)

********Data set: #4

Computed data for frame #1

RDC Data:

I J Exp. [Hz] Comp. [Hz]

C5 H16 23.00 20.45

C12 H22 25.20 19.69

C14 H25 10.30 12.02

C14 H24 3.60 -18.98

C26 H37 -19.00 -18.45

C33 H43 -23.00 -16.28

C31 H41 -21.90 -17.56

C29 H39 -23.90 -17.61

C28 H38 -19.50 -14.51

C35 H46 21.30 19.86

C35 H45 -10.20 -10.70

C47 H58 -25.00 -7.70

C54 H64 -19.70 -8.23

C56 H66 0.00 -8.46

C56 H67 2.90 3.94

C68 H79 -4.20 -5.90

C75 H85 -1.20 -7.64

C73 H83 -5.00 -7.54

C71 H81 -5.30 -5.38

C70 H80 -4.50 -6.71

C77 H87 -1.30 -7.72

C77 H88 4.30 13.42

Cornilescu Quality factor: 0.507519

Alignment tensor information:

A'x=-9.083e-05

A'y=-7.746e-04

A'z= 8.655e-04

Saupe tensor

S'x=-1.362e-04

S'y=-1.162e-03

S'z= 1.298e-03

Alignment tensor eigenvectors

e[x]=(-0.524,-0.081, 0.848)

e[y]=( 0.633,-0.703, 0.323)

e[z]=( 0.570, 0.706, 0.420)

Alignment tensor in laboratory coordinates:

[-5.385e-05,6.894e-04,8.897e-05]

[ 6.894e-04,4.760e-05,4.391e-04]

[ 8.897e-05,4.391e-04,6.243e-06]

SVD condition number is 2.807e+01

Axial component Aa = 1.298e-03

Rhombic component Ar = 6.838e-04

Field=18.79 Teslas[ 2.27]

rhombicity R = 0.527

Asimmetry parameter etha =7.901e-01

GDO = 1.717e-03

ZY'Z'' Euler Angles (degrees)

Set 1

(51.1,65.2,159.1)

Set 2

(-128.9,-65.2,-20.9)

********Data set: #5

Computed data for frame #1

RDC Data:

I J Exp. [Hz] Comp. [Hz]

C5 H16 23.00 20.02

C12 H22 25.20 14.99

C14 H24 10.30 -10.59

C14 H25 3.60 -1.61

C26 H37 -19.00 -16.15

C33 H43 -23.00 -15.72

C31 H41 -21.90 -14.36

C29 H39 -23.90 -19.75

C28 H38 -19.50 -18.87

C35 H45 21.30 7.57

C35 H46 -10.20 0.34

C47 H58 -25.00 3.38

C54 H64 -19.70 -1.95

C56 H67 0.00 10.42

C56 H66 2.90 -5.95

C68 H79 -4.20 -10.27

C75 H85 -1.20 -5.80

C73 H83 -5.00 -9.08

C71 H81 -5.30 -9.30

C70 H80 -4.50 -2.76

C77 H87 -1.30 -3.87

C77 H88 4.30 9.88

Cornilescu Quality factor: 0.680194

Alignment tensor information:

A'x= 2.286e-04

A'y= 1.355e-03

A'z=-1.583e-03

Saupe tensor

S'x= 3.428e-04

S'y= 2.032e-03

S'z=-2.375e-03

Alignment tensor eigenvectors

e[x]=( 0.574, 0.727, 0.377)

e[y]=( 0.337,-0.629, 0.701)

e[z]=( 0.746,-0.275,-0.606)

Alignment tensor in laboratory coordinates:

[-6.530e-04,1.339e-04,1.085e-03]

[ 1.339e-04,5.367e-04,-7.986e-04]

[ 1.085e-03,-7.986e-04,1.163e-04]

SVD condition number is 2.807e+01

Axial component Aa = -2.375e-03

Rhombic component Ar = -1.126e-03

Field=18.79 Teslas[ 2.27]

rhombicity R = 0.474

Asimmetry parameter etha =7.113e-01

GDO = 3.070e-03

ZY'Z'' Euler Angles (degrees)

Set 1

(-20.3,127.3,118.3)

Set 2

(159.7,-127.3,-61.7)

********Data set: #6

Computed data for frame #1

RDC Data:

I J Exp. [Hz] Comp. [Hz]

C5 H16 23.00 19.37

C12 H22 25.20 14.65

C14 H25 10.30 2.28

C14 H24 3.60 -12.80

C26 H37 -19.00 -17.81

C33 H43 -23.00 -16.92

C31 H41 -21.90 -15.96

C29 H39 -23.90 -20.94

C28 H38 -19.50 -19.41

C35 H45 21.30 5.44

C35 H46 -10.20 1.48

C47 H58 -25.00 2.42

C54 H64 -19.70 -2.43

C56 H67 0.00 11.53

C56 H66 2.90 -6.04

C68 H79 -4.20 -9.29

C75 H85 -1.20 -5.71

C73 H83 -5.00 -8.60

C71 H81 -5.30 -8.31

C70 H80 -4.50 -2.80

C77 H87 -1.30 -4.02

C77 H88 4.30 8.67

Cornilescu Quality factor: 0.657434

Alignment tensor information:

A'x= 3.849e-04

A'y= 1.089e-03

A'z=-1.474e-03

Saupe tensor

S'x= 5.773e-04

S'y= 1.634e-03

S'z=-2.211e-03

Alignment tensor eigenvectors

e[x]=( 0.566, 0.753, 0.336)

e[y]=( 0.353,-0.589, 0.727)

e[z]=( 0.745,-0.293,-0.599)

Alignment tensor in laboratory coordinates:

[-5.602e-04,2.591e-04,1.011e-03]

[ 2.591e-04,4.700e-04,-6.276e-04]

[ 1.011e-03,-6.276e-04,9.019e-05]

SVD condition number is 2.807e+01

Axial component Aa = -2.211e-03

Rhombic component Ar = -7.045e-04

Field=18.79 Teslas[ 2.27]

rhombicity R = 0.319

Asimmetry parameter etha =4.779e-01

GDO = 2.695e-03

ZY'Z'' Euler Angles (degrees)

Set 1

(-21.4,126.8,114.8)

Set 2

(158.6,-126.8,-65.2)

********Data set: #7

Computed data for frame #1

RDC Data:

I J Exp. [Hz] Comp. [Hz]

C5 H16 23.00 21.13

C12 H22 25.20 20.23

C14 H24 10.30 -16.81

C14 H25 3.60 7.58

C26 H37 -19.00 -16.60

C33 H43 -23.00 -14.90

C31 H41 -21.90 -15.85

C29 H39 -23.90 -16.09

C28 H38 -19.50 -13.64

C35 H46 21.30 19.42

C35 H45 -10.20 -8.48

C47 H58 -25.00 -6.53

C54 H64 -19.70 -7.38

C56 H67 0.00 1.76

C56 H66 2.90 -7.88

C68 H79 -4.20 -6.25

C75 H85 -1.20 -7.23

C73 H83 -5.00 -7.42

C71 H81 -5.30 -5.78

C70 H80 -4.50 -6.27

C77 H87 -1.30 -7.14

C77 H88 4.30 15.00

Cornilescu Quality factor: 0.58285

Alignment tensor information:

A'x=-1.017e-04

A'y=-6.039e-04

A'z= 7.057e-04

Saupe tensor

S'x=-1.526e-04

S'y=-9.059e-04

S'z= 1.058e-03

Alignment tensor eigenvectors

e[x]=(-0.400,-0.223, 0.889)

e[y]=( 0.737,-0.655, 0.167)

e[z]=( 0.545, 0.722, 0.426)

Alignment tensor in laboratory coordinates:

[-1.349e-04,5.601e-04,1.258e-04]

[ 5.601e-04,1.038e-04,3.034e-04]

[ 1.258e-04,3.034e-04,3.106e-05]

SVD condition number is 2.807e+01

Axial component Aa = 1.058e-03

Rhombic component Ar = 5.022e-04

Field=18.79 Teslas[ 2.27]

rhombicity R = 0.474

Asimmetry parameter etha =7.117e-01

GDO = 1.368e-03

ZY'Z'' Euler Angles (degrees)

Set 1

(53.0,64.8,169.4)

Set 2

(-127.0,-64.8,-10.6)

********Data set: #8

Computed data for frame #1

RDC Data:

I J Exp. [Hz] Comp. [Hz]

C5 H16 23.00 20.48

C12 H22 25.20 19.89

C14 H25 10.30 11.46

C14 H24 3.60 -19.02

C26 H37 -19.00 -18.26

C33 H43 -23.00 -16.10

C31 H41 -21.90 -17.44

C29 H39 -23.90 -17.28

C28 H38 -19.50 -14.18

C35 H46 21.30 20.56

C35 H45 -10.20 -10.62

C47 H58 -25.00 -7.49

C54 H64 -19.70 -7.86

C56 H67 0.00 2.86

C56 H66 2.90 -7.97

C68 H79 -4.20 -5.28

C75 H85 -1.20 -7.15

C73 H83 -5.00 -6.95

C71 H81 -5.30 -4.78

C70 H80 -4.50 -6.31

C77 H87 -1.30 -7.29

C77 H88 4.30 13.80

Cornilescu Quality factor: 0.519903

Alignment tensor information:

A'x=-5.829e-05

A'y=-8.054e-04

A'z= 8.637e-04

Saupe tensor

S'x=-8.743e-05

S'y=-1.208e-03

S'z= 1.296e-03

Alignment tensor eigenvectors

e[x]=(-0.564,-0.049, 0.824)

e[y]=( 0.606,-0.703, 0.373)

e[z]=( 0.561, 0.710, 0.426)

Alignment tensor in laboratory coordinates:

[-4.206e-05,6.853e-04,5.153e-05]

[ 6.853e-04,3.711e-05,4.744e-04]

[ 5.153e-05,4.744e-04,4.955e-06]

SVD condition number is 2.807e+01

Axial component Aa = 1.296e-03

Rhombic component Ar = 7.471e-04

Field=18.79 Teslas[ 2.27]

rhombicity R = 0.577

Asimmetry parameter etha =8.650e-01

GDO = 1.754e-03

ZY'Z'' Euler Angles (degrees)

Set 1

(51.7,64.8,155.7)

Set 2

(-128.3,-64.8,-24.3)

********Data set: #9

Computed data for frame #1

RDC Data:

I J Exp. [Hz] Comp. [Hz]

C5 H16 23.00 20.04

C12 H22 25.20 14.21

C14 H24 10.30 -10.48

C14 H25 3.60 -0.10

C26 H37 -19.00 -16.79

C33 H43 -23.00 -15.33

C31 H41 -21.90 -14.29

C29 H39 -23.90 -20.64

C28 H38 -19.50 -18.26

C35 H45 21.30 7.52

C35 H46 -10.20 -1.83

C47 H58 -25.00 4.38

C54 H64 -19.70 -1.82

C56 H66 0.00 -6.41

C56 H67 2.90 11.44

C68 H79 -4.20 -10.02

C75 H85 -1.20 -5.84

C73 H83 -5.00 -9.47

C71 H81 -5.30 -8.66

C70 H80 -4.50 -1.97

C77 H88 -1.30 6.68

C77 H87 4.30 -3.71

Cornilescu Quality factor: 0.685162

Alignment tensor information:

A'x= 5.491e-04

A'y= 1.295e-03

A'z=-1.844e-03

Saupe tensor

S'x= 8.237e-04

S'y= 1.942e-03

S'z=-2.766e-03

Alignment tensor eigenvectors

e[x]=( 0.581, 0.738, 0.342)

e[y]=( 0.359,-0.610, 0.706)

e[z]=( 0.730,-0.288,-0.620)

Alignment tensor in laboratory coordinates:

[-6.303e-04,3.391e-04,1.272e-03]

[ 3.391e-04,6.287e-04,-7.480e-04]

[ 1.272e-03,-7.480e-04,1.601e-06]

SVD condition number is 2.807e+01

Axial component Aa = -2.766e-03

Rhombic component Ar = -7.458e-04

Field=18.79 Teslas[ 2.27]

rhombicity R = 0.270

Asimmetry parameter etha =4.044e-01

GDO = 3.322e-03

ZY'Z'' Euler Angles (degrees)

Set 1

(-21.5,128.3,115.8)

Set 2

(158.5,-128.3,-64.2)

********Data set: #10

Computed data for frame #1

RDC Data:

I J Exp. [Hz] Comp. [Hz]

C5 H16 23.00 19.39

C12 H22 25.20 13.87

C14 H25 10.30 3.79

C14 H24 3.60 -12.69

C26 H37 -19.00 -18.45

C33 H43 -23.00 -16.53

C31 H41 -21.90 -15.89

C29 H39 -23.90 -21.83

C28 H38 -19.50 -18.81

C35 H45 21.30 5.38

C35 H46 -10.20 -0.69

C47 H58 -25.00 3.42

C54 H64 -19.70 -2.30

C56 H66 0.00 -6.50

C56 H67 2.90 12.55

C68 H79 -4.20 -9.05

C75 H85 -1.20 -5.75

C73 H83 -5.00 -9.00

C71 H81 -5.30 -7.67

C70 H80 -4.50 -2.00

C77 H88 -1.30 5.47

C77 H87 4.30 -3.86

Cornilescu Quality factor: 0.659898

Alignment tensor information:

A'x= 6.993e-04

A'y= 1.036e-03

A'z=-1.735e-03

Saupe tensor

S'x= 1.049e-03

S'y= 1.554e-03

S'z=-2.603e-03

Alignment tensor eigenvectors

e[x]=( 0.534, 0.812, 0.236)

e[y]=( 0.426,-0.500, 0.754)

e[z]=( 0.730,-0.302,-0.613)

Alignment tensor in laboratory coordinates:

[-5.375e-04,4.644e-04,1.198e-03]

[ 4.644e-04,5.620e-04,-5.770e-04]

[ 1.198e-03,-5.770e-04,-2.450e-05]

SVD condition number is 2.807e+01

Axial component Aa = -2.603e-03

Rhombic component Ar = -3.365e-04

Field=18.79 Teslas[ 2.27]

rhombicity R = 0.129

Asimmetry parameter etha =1.939e-01

GDO = 3.033e-03

ZY'Z'' Euler Angles (degrees)

Set 1

(-22.4,127.8,107.4)

Set 2

(157.6,-127.8,-72.6)

********Data set: #11

Computed data for frame #1

RDC Data:

I J Exp. [Hz] Comp. [Hz]

C5 H16 23.00 21.15

C12 H22 25.20 19.45

C14 H24 10.30 -16.69

C14 H25 3.60 9.09

C26 H37 -19.00 -17.24

C33 H43 -23.00 -14.51

C31 H41 -21.90 -15.77

C29 H39 -23.90 -16.98

C28 H38 -19.50 -13.04

C35 H46 21.30 17.26

C35 H45 -10.20 -8.54

C47 H58 -25.00 -5.54

C54 H64 -19.70 -7.25

C56 H66 0.00 -8.34

C56 H67 2.90 2.78

C68 H79 -4.20 -6.01

C75 H85 -1.20 -7.27

C73 H83 -5.00 -7.82

C71 H81 -5.30 -5.14

C70 H80 -4.50 -5.48

C77 H88 -1.30 11.80

C77 H87 4.30 -6.99

Cornilescu Quality factor: 0.609617

Alignment tensor information:

A'x=-2.844e-04

A'y=-7.395e-04

A'z= 1.024e-03

Saupe tensor

S'x=-4.266e-04

S'y=-1.109e-03

S'z= 1.536e-03

Alignment tensor eigenvectors

e[x]=(-0.220,-0.326, 0.919)

e[y]=( 0.780,-0.625,-0.035)

e[z]=( 0.586, 0.709, 0.392)

Alignment tensor in laboratory coordinates:

[-1.122e-04,7.653e-04,3.130e-04]

[ 7.653e-04,1.958e-04,3.540e-04]

[ 3.130e-04,3.540e-04,-8.364e-05]

SVD condition number is 2.807e+01

Axial component Aa = 1.536e-03

Rhombic component Ar = 4.551e-04

Field=18.79 Teslas[ 2.27]

rhombicity R = 0.296

Asimmetry parameter etha =4.444e-01

GDO = 1.859e-03

ZY'Z'' Euler Angles (degrees)

Set 1

(50.4,66.9,-177.8)

Set 2

(-129.6,-66.9,2.2)

********Data set: #12

Computed data for frame #1

RDC Data:

I J Exp. [Hz] Comp. [Hz]

C5 H16 23.00 20.50

C12 H22 25.20 19.11

C14 H25 10.30 12.98

C14 H24 3.60 -18.91

C26 H37 -19.00 -18.90

C33 H43 -23.00 -15.71

C31 H41 -21.90 -17.37

C29 H39 -23.90 -18.17

C28 H38 -19.50 -13.58

C35 H46 21.30 18.39

C35 H45 -10.20 -10.68

C47 H58 -25.00 -6.49

C54 H64 -19.70 -7.74

C56 H66 0.00 -8.43

C56 H67 2.90 3.88

C68 H79 -4.20 -5.03

C75 H85 -1.20 -7.19

C73 H83 -5.00 -7.35

C71 H81 -5.30 -4.14

C70 H80 -4.50 -5.52

C77 H88 -1.30 10.60

C77 H87 4.30 -7.13

Cornilescu Quality factor: 0.546515

Alignment tensor information:

A'x=-2.926e-04

A'y=-8.911e-04

A'z= 1.184e-03

Saupe tensor

S'x=-4.388e-04

S'y=-1.337e-03

S'z= 1.776e-03

Alignment tensor eigenvectors

e[x]=(-0.474,-0.089, 0.876)

e[y]=( 0.648,-0.709, 0.278)

e[z]=( 0.596, 0.700, 0.394)

Alignment tensor in laboratory coordinates:

[-1.937e-05,8.906e-04,2.388e-04]

[ 8.906e-04,1.291e-04,5.251e-04]

[ 2.388e-04,5.251e-04,-1.097e-04]

SVD condition number is 2.807e+01

Axial component Aa = 1.776e-03

Rhombic component Ar = 5.986e-04

Field=18.79 Teslas[ 2.27]

rhombicity R = 0.337

Asimmetry parameter etha =5.057e-01

GDO = 2.177e-03

ZY'Z'' Euler Angles (degrees)

Set 1

(49.6,66.8,162.4)

Set 2

(-130.4,-66.8,-17.6)

********Data set: #13

Computed data for frame #1

RDC Data:

I J Exp. [Hz] Comp. [Hz]

C5 H16 23.00 20.07

C12 H22 25.20 14.42

C14 H24 10.30 -10.52

C14 H25 3.60 -0.66

C26 H37 -19.00 -16.61

C33 H43 -23.00 -15.15

C31 H41 -21.90 -14.17

C29 H39 -23.90 -20.31

C28 H38 -19.50 -17.94

C35 H45 21.30 7.60

C35 H46 -10.20 -1.13

C47 H58 -25.00 4.58

C54 H64 -19.70 -1.45

C56 H67 0.00 10.37

C56 H66 2.90 -5.92

C68 H79 -4.20 -9.40

C75 H85 -1.20 -5.35

C73 H83 -5.00 -8.89

C71 H81 -5.30 -8.06

C70 H80 -4.50 -1.57

C77 H88 -1.30 7.06

C77 H87 4.30 -3.28

Cornilescu Quality factor: 0.698669

Alignment tensor information:

A'x= 5.467e-04

A'y= 1.244e-03

A'z=-1.791e-03

Saupe tensor

S'x= 8.200e-04

S'y= 1.866e-03

S'z=-2.686e-03

Alignment tensor eigenvectors

e[x]=( 0.579, 0.738, 0.346)

e[y]=( 0.358,-0.612, 0.706)

e[z]=( 0.733,-0.285,-0.618)

Alignment tensor in laboratory coordinates:

[-6.185e-04,3.350e-04,1.235e-03]

[ 3.350e-04,6.182e-04,-7.127e-04]

[ 1.235e-03,-7.127e-04,3.136e-07]

SVD condition number is 2.807e+01

Axial component Aa = -2.686e-03

Rhombic component Ar = -6.976e-04

Field=18.79 Teslas[ 2.27]

rhombicity R = 0.260

Asimmetry parameter etha =3.895e-01

GDO = 3.218e-03

ZY'Z'' Euler Angles (degrees)

Set 1

(-21.2,128.2,116.1)

Set 2

(158.8,-128.2,-63.9)

********Data set: #14

Computed data for frame #1

RDC Data:

I J Exp. [Hz] Comp. [Hz]

C5 H16 23.00 19.43

C12 H22 25.20 14.08

C14 H25 10.30 3.23

C14 H24 3.60 -12.73

C26 H37 -19.00 -18.27

C33 H43 -23.00 -16.34

C31 H41 -21.90 -15.77

C29 H39 -23.90 -21.50

C28 H38 -19.50 -18.48

C35 H45 21.30 5.46

C35 H46 -10.20 0.01

C47 H58 -25.00 3.63

C54 H64 -19.70 -1.94

C56 H67 0.00 11.47

C56 H66 2.90 -6.01

C68 H79 -4.20 -8.42

C75 H85 -1.20 -5.26

C73 H83 -5.00 -8.42

C71 H81 -5.30 -7.07

C70 H80 -4.50 -1.61

C77 H88 -1.30 5.86

C77 H87 4.30 -3.43

Cornilescu Quality factor: 0.67488

Alignment tensor information:

A'x= 6.962e-04

A'y= 9.858e-04

A'z=-1.682e-03

Saupe tensor

S'x= 1.044e-03

S'y= 1.479e-03

S'z=-2.523e-03

Alignment tensor eigenvectors

e[x]=( 0.524, 0.821, 0.226)

e[y]=( 0.435,-0.486, 0.759)

e[z]=( 0.733,-0.299,-0.611)

Alignment tensor in laboratory coordinates:

[-5.257e-04,4.603e-04,1.161e-03]

[ 4.603e-04,5.515e-04,-5.416e-04]

[ 1.161e-03,-5.416e-04,-2.579e-05]

SVD condition number is 2.807e+01

Axial component Aa = -2.523e-03

Rhombic component Ar = -2.896e-04

Field=18.79 Teslas[ 2.27]

rhombicity R = 0.115

Asimmetry parameter etha =1.722e-01

GDO = 2.935e-03

ZY'Z'' Euler Angles (degrees)

Set 1

(-22.2,127.7,106.6)

Set 2

(157.8,-127.7,-73.4)

********Data set: #15

Computed data for frame #1

RDC Data:

I J Exp. [Hz] Comp. [Hz]

C5 H16 23.00 21.18

C12 H22 25.20 19.66

C14 H24 10.30 -16.74

C14 H25 3.60 8.53

C26 H37 -19.00 -17.06

C33 H43 -23.00 -14.33

C31 H41 -21.90 -15.66

C29 H39 -23.90 -16.65

C28 H38 -19.50 -12.71

C35 H46 21.30 17.95

C35 H45 -10.20 -8.46

C47 H58 -25.00 -5.33

C54 H64 -19.70 -6.89

C56 H67 0.00 1.70

C56 H66 2.90 -7.85

C68 H79 -4.20 -5.38

C75 H85 -1.20 -6.79

C73 H83 -5.00 -7.23

C71 H81 -5.30 -4.54

C70 H80 -4.50 -5.08

C77 H88 -1.30 12.19

C77 H87 4.30 -6.56

Cornilescu Quality factor: 0.618835

Alignment tensor information:

A'x=-2.869e-04

A'y=-7.347e-04

A'z= 1.022e-03

Saupe tensor

S'x=-4.303e-04

S'y=-1.102e-03

S'z= 1.532e-03

Alignment tensor eigenvectors

e[x]=(-0.310,-0.256, 0.916)

e[y]=( 0.753,-0.654, 0.072)

e[z]=( 0.580, 0.712, 0.395)

Alignment tensor in laboratory coordinates:

[-1.004e-04,7.612e-04,2.756e-04]

[ 7.612e-04,1.853e-04,3.894e-04]

[ 2.756e-04,3.894e-04,-8.492e-05]

SVD condition number is 2.807e+01

Axial component Aa = 1.532e-03

Rhombic component Ar = 4.478e-04

Field=18.79 Teslas[ 2.27]

rhombicity R = 0.292

Asimmetry parameter etha =4.384e-01

GDO = 1.852e-03

ZY'Z'' Euler Angles (degrees)

Set 1

(50.8,66.7,175.5)

Set 2

(-129.2,-66.7,-4.5)

********Data set: #16

Computed data for frame #1

RDC Data:

I J Exp. [Hz] Comp. [Hz]

C5 H16 23.00 20.54

C12 H22 25.20 19.32

C14 H25 10.30 12.42

C14 H24 3.60 -18.95

C26 H37 -19.00 -18.72

C33 H43 -23.00 -15.52

C31 H41 -21.90 -17.25

C29 H39 -23.90 -17.84

C28 H38 -19.50 -13.25

C35 H46 21.30 19.09

C35 H45 -10.20 -10.60

C47 H58 -25.00 -6.29

C54 H64 -19.70 -7.37

C56 H67 0.00 2.81

C56 H66 2.90 -7.94

C68 H79 -4.20 -4.40

C75 H85 -1.20 -6.70

C73 H83 -5.00 -6.76

C71 H81 -5.30 -3.55

C70 H80 -4.50 -5.12

C77 H88 -1.30 10.99

C77 H87 4.30 -6.70

Cornilescu Quality factor: 0.557951

Alignment tensor information:

A'x=-2.627e-04

A'y=-9.183e-04

A'z= 1.181e-03

Saupe tensor

S'x=-3.941e-04

S'y=-1.377e-03

S'z= 1.772e-03

Alignment tensor eigenvectors

e[x]=(-0.520,-0.044, 0.853)

e[y]=( 0.616,-0.710, 0.339)

e[z]=( 0.591, 0.702, 0.397)

Alignment tensor in laboratory coordinates:

[-7.586e-06,8.865e-04,2.013e-04]

[ 8.865e-04,1.186e-04,5.604e-04]

[ 2.013e-04,5.604e-04,-1.110e-04]

SVD condition number is 2.807e+01

Axial component Aa = 1.772e-03

Rhombic component Ar = 6.556e-04

Field=18.79 Teslas[ 2.27]

rhombicity R = 0.370

Asimmetry parameter etha =5.551e-01

GDO = 2.198e-03

ZY'Z'' Euler Angles (degrees)

Set 1

(49.9,66.6,158.3)

Set 2

(-130.1,-66.6,-21.7)

********

MSpin-RDC pluginmi. jul. 28 11:00:33 2021

gg2/gt2/gg2

CR_tr

!* MSpin-RDC Plugin *!

********

!* Computation flags *!

Method: SVD

Scaling mode: Hz

Field (T): 18.7923

1H Larmor Frequency: 800.13

Scale QCSA with axial component: False

Include CSA gel shift (isotropic) correction:False

Optimize CSA gel shift (isotropic) correction scale:False

Estimate CSA gel shift (isotropic) correction scale:False

Gel Shift Correction Scale: 0.15

Single Tensor: False

Superimpose: False

Average methyl groups: False

Average methylene groups: False

Average phenyl groups: False

Bootstrapping: False

RDC Std. Error [ppm]: 1

CSA Std. Error [ppm]: 0.01

PCS Std. Error [ppm]: 0.01

DQ Std. Error [Hz]: 1

********

!* Permutations *!

There are no permutations on the original data set

********

Data set: #1

Computed data for frame #1

RDC Data:

I J Exp. [Hz] Comp. [Hz]

C5 H16 23.00 21.42

C12 H22 25.20 26.20

C26 H37 -19.00 -23.45

C33 H43 -23.00 -22.81

C31 H41 -21.90 -25.30

C29 H39 -23.90 -18.40

C28 H38 -19.50 -17.69

C47 H58 -25.00 -19.45

C54 H64 -19.70 -13.50

C68 H79 -4.20 -2.80

C75 H85 -1.20 -8.78

C73 H83 -5.00 -4.85

C71 H81 -5.30 -3.81

C70 H80 -4.50 -12.02

Cornilescu Quality factor: 0.236102

Alignment tensor information:

A'x= 4.591e-04

A'y= 1.702e-03

A'z=-2.162e-03

Saupe tensor

S'x= 6.887e-04

S'y= 2.554e-03

S'z=-3.242e-03

Alignment tensor eigenvectors

e[x]=( 0.686, 0.675, 0.272)

e[y]=( 0.578,-0.278,-0.767)

e[z]=(-0.442, 0.684,-0.581)

Alignment tensor in laboratory coordinates:

[ 3.633e-04,5.911e-04,-1.224e-03]

[ 5.911e-04,-6.689e-04,1.306e-03]

[-1.224e-03,1.306e-03,3.056e-04]

SVD condition number is 4.035e+01

Axial component Aa = -3.242e-03

Rhombic component Ar = -1.243e-03

Field=18.79 Teslas[ 2.27]

rhombicity R = 0.383

Asimmetry parameter etha =5.752e-01

GDO = 4.042e-03

ZY'Z'' Euler Angles (degrees)

Set 1

(122.9,125.5,-109.5)

Set 2

(-57.1,-125.5,70.5)

********

MSpin-RDC pluginmi. jul. 28 11:05:47 2021

gg2/gt2/gg2

CR_E5C12

!* MSpin-RDC Plugin *!

********

!* Computation flags *!

Method: SVD

Scaling mode: Hz

Field (T): 18.7923

1H Larmor Frequency: 800.13

Scale QCSA with axial component: False

Include CSA gel shift (isotropic) correction:False

Optimize CSA gel shift (isotropic) correction scale:False

Estimate CSA gel shift (isotropic) correction scale:False

Gel Shift Correction Scale: 0.15

Single Tensor: False

Superimpose: False

Average methyl groups: False

Average methylene groups: False

Average phenyl groups: False

Bootstrapping: False

RDC Std. Error [ppm]: 1

CSA Std. Error [ppm]: 0.01

PCS Std. Error [ppm]: 0.01

DQ Std. Error [Hz]: 1

********

!* Permutations *!

There are no permutations on the original data set

********

Data set: #1

Computed data for frame #1

RDC Data:

I J Exp. [Hz] Comp. [Hz]

C5 H16 11.80 12.96

C12 H22 12.70 9.88

C26 H37 10.50 8.20

C33 H43 10.60 12.75

C47 H58 9.90 10.25

C54 H64 9.00 5.25

C68 H79 4.90 3.98

C75 H85 4.80 3.67

C71 H81 5.30 6.00

C70 H80 8.50 8.07

C77 H87 0.00 4.99

C77 H88 0.00 1.60

Cornilescu Quality factor: 0.273721

Alignment tensor information:

A'x=-1.237e-04

A'y=-1.257e-03

A'z= 1.380e-03

Saupe tensor

S'x=-1.855e-04

S'y=-1.885e-03

S'z= 2.070e-03

Alignment tensor eigenvectors

e[x]=( 0.569,-0.587, 0.575)

e[y]=( 0.632,-0.135,-0.763)

e[z]=( 0.526, 0.798, 0.294)

Alignment tensor in laboratory coordinates:

[-1.593e-04,7.284e-04,7.790e-04]

[ 7.284e-04,8.128e-04,2.357e-04]

[ 7.790e-04,2.357e-04,-6.535e-04]

SVD condition number is 3.076e+01

Axial component Aa = 2.070e-03

Rhombic component Ar = 1.133e-03

Field=18.79 Teslas[ 2.27]

rhombicity R = 0.547

Asimmetry parameter etha =8.208e-01

GDO = 2.764e-03

ZY'Z'' Euler Angles (degrees)

Set 1

(56.6,72.9,-127.0)

Set 2

(-123.4,-72.9,53.0)

********

MSpin-RDC pluginmi. jul. 28 12:27:35 2021

gg2/gt2/gg2F

CRF_all

!* MSpin-RDC Plugin *!

********

!* Computation flags *!

Method: SVD

Scaling mode: Hz

Field (T): 18.7923

1H Larmor Frequency: 800.13

Scale QCSA with axial component: False

Include CSA gel shift (isotropic) correction:False

Optimize CSA gel shift (isotropic) correction scale:False

Estimate CSA gel shift (isotropic) correction scale:False

Gel Shift Correction Scale: 0.15

Single Tensor: False

Superimpose: False

Average methyl groups: False

Average methylene groups: False

Average phenyl groups: False

Bootstrapping: False

RDC Std. Error [ppm]: 1

CSA Std. Error [ppm]: 0.01

PCS Std. Error [ppm]: 0.01

DQ Std. Error [Hz]: 1

********

!* Permutations *!

There are 4 possible permutations on the original data set

********

Data set: #1

Computed data for frame #1

RDC Data:

I J Exp. [Hz] Comp. [Hz]

C5 H16 18.00 19.58

C10 H20 23.00 18.32

C10 F11 -13.00 -1.37

C25 H36 -19.00 -16.64

C32 H42 -23.00 -16.52

C30 H40 -21.90 -14.32

C28 H38 -23.90 -21.82

C27 H37 -19.50 -21.21

C34 H44 21.30 -7.70

C34 H45 -10.20 0.20

C46 H57 -30.00 -8.17

C51 H61 -24.00 -8.60

C51 F52 13.00 -12.77

C66 H77 -4.20 -8.76

C73 H83 -1.20 -2.95

C71 H81 -5.00 -7.55

C69 H79 -5.30 -7.24

C68 H78 -4.50 2.31

C75 H85 -1.30 0.55

C75 H86 4.30 2.71

Cornilescu Quality factor: 0.688906

Alignment tensor information:

A'x= 3.138e-04

A'y= 1.885e-03

A'z=-2.198e-03

Saupe tensor

S'x= 4.707e-04

S'y= 2.827e-03

S'z=-3.298e-03

Alignment tensor eigenvectors

e[x]=( 0.615, 0.703, 0.358)

e[y]=( 0.345,-0.648, 0.679)

e[z]=( 0.709,-0.294,-0.641)

Alignment tensor in laboratory coordinates:

[-7.635e-04,1.723e-04,1.510e-03]

[ 1.723e-04,7.568e-04,-1.164e-03]

[ 1.510e-03,-1.164e-03,6.767e-06]

SVD condition number is 2.435e+01

Axial component Aa = -3.298e-03

Rhombic component Ar = -1.571e-03

Field=18.79 Teslas[ 2.27]

rhombicity R = 0.476

Asimmetry parameter etha =7.145e-01

GDO = 4.266e-03

ZY'Z'' Euler Angles (degrees)

Set 1

(-22.5,129.8,117.8)

Set 2

(157.5,-129.8,-62.2)

********Data set: #2

Computed data for frame #1

RDC Data:

I J Exp. [Hz] Comp. [Hz]

C5 H16 18.00 25.99

C10 H20 23.00 19.02

C10 F11 -13.00 1.10

C25 H36 -19.00 -20.85

C32 H42 -23.00 -15.52

C30 H40 -21.90 -17.06

C28 H38 -23.90 -22.77

C27 H37 -19.50 -14.85

C34 H45 21.30 14.21

C34 H44 -10.20 -17.73

C46 H57 -30.00 -12.12

C51 H61 -24.00 -19.08

C51 F52 13.00 -7.51

C66 H77 -4.20 -6.36

C73 H83 -1.20 -6.51

C71 H81 -5.00 -9.40

C69 H79 -5.30 -3.74

C68 H78 -4.50 -0.44

C75 H85 -1.30 -3.76

C75 H86 4.30 14.18

Cornilescu Quality factor: 0.502793

Alignment tensor information:

A'x=-1.237e-04

A'y=-1.899e-03

A'z= 2.022e-03

Saupe tensor

S'x=-1.855e-04

S'y=-2.848e-03

S'z= 3.034e-03

Alignment tensor eigenvectors

e[x]=( 0.277,-0.596, 0.754)

e[y]=( 0.752,-0.354,-0.556)

e[z]=( 0.598, 0.721, 0.351)

Alignment tensor in laboratory coordinates:

[-3.605e-04,1.397e-03,1.192e-03]

[ 1.397e-03,7.693e-04,1.932e-04]

[ 1.192e-03,1.932e-04,-4.088e-04]

SVD condition number is 2.435e+01

Axial component Aa = 3.034e-03

Rhombic component Ar = 1.775e-03

Field=18.79 Teslas[ 2.27]

rhombicity R = 0.585

Asimmetry parameter etha =8.777e-01

GDO = 4.123e-03

ZY'Z'' Euler Angles (degrees)

Set 1

(50.3,69.5,-143.6)

Set 2

(-129.7,-69.5,36.4)

********Data set: #3

Computed data for frame #1

RDC Data:

I J Exp. [Hz] Comp. [Hz]

C5 H16 18.00 19.37

C10 H20 23.00 17.93

C10 F11 -13.00 -2.00

C25 H36 -19.00 -16.53

C32 H42 -23.00 -16.25

C30 H40 -21.90 -13.80

C28 H38 -23.90 -22.42

C27 H37 -19.50 -21.53

C34 H44 21.30 -7.64

C34 H45 -10.20 -2.54

C46 H57 -30.00 -8.00

C51 H61 -24.00 -8.68

C51 F52 13.00 -13.35

C66 H77 -4.20 -8.48

C73 H83 -1.20 -2.01

C71 H81 -5.00 -7.22

C69 H79 -5.30 -6.70

C68 H78 -4.50 4.03

C75 H86 -1.30 0.28

C75 H85 4.30 1.97

Cornilescu Quality factor: 0.68924

Alignment tensor information:

A'x= 3.761e-04

A'y= 2.122e-03

A'z=-2.498e-03

Saupe tensor

S'x= 5.641e-04

S'y= 3.182e-03

S'z=-3.746e-03

Alignment tensor eigenvectors

e[x]=( 0.618, 0.702, 0.354)

e[y]=( 0.352,-0.650, 0.674)

e[z]=( 0.703,-0.292,-0.649)

Alignment tensor in laboratory coordinates:

[-8.268e-04,1.909e-04,1.724e-03]

[ 1.909e-04,8.672e-04,-1.309e-03]

[ 1.724e-03,-1.309e-03,-4.044e-05]

SVD condition number is 2.435e+01

Axial component Aa = -3.746e-03

Rhombic component Ar = -1.745e-03

Field=18.79 Teslas[ 2.27]

rhombicity R = 0.466

Asimmetry parameter etha =6.989e-01

GDO = 4.825e-03

ZY'Z'' Euler Angles (degrees)

Set 1

(-22.6,130.4,117.7)

Set 2

(157.4,-130.4,-62.3)

********Data set: #4

Computed data for frame #1

RDC Data:

I J Exp. [Hz] Comp. [Hz]

C5 H16 18.00 25.78

C10 H20 23.00 18.63

C10 F11 -13.00 0.47

C25 H36 -19.00 -20.73

C32 H42 -23.00 -15.25

C30 H40 -21.90 -16.54

C28 H38 -23.90 -23.37

C27 H37 -19.50 -15.17

C34 H45 21.30 11.47

C34 H44 -10.20 -17.67

C46 H57 -30.00 -11.95

C51 H61 -24.00 -19.16

C51 F52 13.00 -8.09

C66 H77 -4.20 -6.08

C73 H83 -1.20 -5.57

C71 H81 -5.00 -9.07

C69 H79 -5.30 -3.20

C68 H78 -4.50 1.28

C75 H86 -1.30 11.75

C75 H85 4.30 -2.34

Cornilescu Quality factor: 0.533381

Alignment tensor information:

A'x= 1.016e-04

A'y= 2.084e-03

A'z=-2.186e-03

Saupe tensor

S'x= 1.524e-04

S'y= 3.126e-03

S'z=-3.279e-03

Alignment tensor eigenvectors

e[x]=(-0.310, 0.610,-0.729)

e[y]=( 0.601, 0.720, 0.346)

e[z]=( 0.737,-0.331,-0.590)

Alignment tensor in laboratory coordinates:

[-4.238e-04,1.416e-03,1.407e-03]

[ 1.416e-03,8.798e-04,4.844e-05]

[ 1.407e-03,4.844e-05,-4.560e-04]

SVD condition number is 2.435e+01

Axial component Aa = -3.279e-03

Rhombic component Ar = -1.983e-03

Field=18.79 Teslas[ 2.27]

rhombicity R = 0.605

Asimmetry parameter etha =9.071e-01

GDO = 4.498e-03

ZY'Z'' Euler Angles (degrees)

Set 1

(-24.2,126.1,25.4)

Set 2

(155.8,-126.1,-154.6)

********

MSpin-RDC pluginmi. jul. 28 12:31:13 2021

gg2/gt2/gg2F

CRF_tr

!* MSpin-RDC Plugin *!

********

!* Computation flags *!

Method: SVD

Scaling mode: Hz

Field (T): 18.7923

1H Larmor Frequency: 800.13

Scale QCSA with axial component: False

Include CSA gel shift (isotropic) correction:False

Optimize CSA gel shift (isotropic) correction scale:False

Estimate CSA gel shift (isotropic) correction scale:False

Gel Shift Correction Scale: 0.15

Single Tensor: False

Superimpose: False

Average methyl groups: False

Average methylene groups: False

Average phenyl groups: False

Bootstrapping: False

RDC Std. Error [ppm]: 1

CSA Std. Error [ppm]: 0.01

PCS Std. Error [ppm]: 0.01

DQ Std. Error [Hz]: 1

********

!* Permutations *!

There are no permutations on the original data set

********

Data set: #1

Computed data for frame #1

RDC Data:

I J Exp. [Hz] Comp. [Hz]

C5 H16 18.00 28.29

C10 H20 23.00 19.85

C10 F11 -13.00 1.95

C25 H36 -19.00 -21.98

C32 H42 -23.00 -15.32

C30 H40 -21.90 -17.92

C28 H38 -23.90 -22.81

C27 H37 -19.50 -13.06

C46 H57 -30.00 -13.81

C51 H61 -24.00 -22.42

C51 F52 13.00 -5.94

C66 H77 -4.20 -6.41

C73 H83 -1.20 -8.32

C71 H81 -5.00 -10.59

C69 H79 -5.30 -3.57

C68 H78 -4.50 -2.25

Cornilescu Quality factor: 0.482641

Alignment tensor information:

A'x=-6.816e-04

A'y=-1.776e-03

A'z= 2.457e-03

Saupe tensor

S'x=-1.022e-03

S'y=-2.664e-03

S'z= 3.686e-03

Alignment tensor eigenvectors

e[x]=( 0.166,-0.542, 0.824)

e[y]=( 0.786,-0.433,-0.442)

e[z]=( 0.596, 0.721, 0.354)

Alignment tensor in laboratory coordinates:

[-2.413e-04,1.721e-03,1.042e-03]

[ 1.721e-03,7.439e-04,5.910e-04]

[ 1.042e-03,5.910e-04,-5.026e-04]

SVD condition number is 3.514e+01

Axial component Aa = 3.686e-03

Rhombic component Ar = 1.094e-03

Field=18.79 Teslas[ 2.27]

rhombicity R = 0.297

Asimmetry parameter etha =4.453e-01

GDO = 4.462e-03

ZY'Z'' Euler Angles (degrees)

Set 1

(50.4,69.3,-151.8)

Set 2

(-129.6,-69.3,28.2)

********

MSpin-RDC pluginmi. jul. 28 12:33:00 2021

gt2/gg2/gt2

CR_all

!* MSpin-RDC Plugin *!

********

!* Computation flags *!

Method: SVD

Scaling mode: Hz

Field (T): 18.7923

1H Larmor Frequency: 800.13

Scale QCSA with axial component: False

Include CSA gel shift (isotropic) correction:False

Optimize CSA gel shift (isotropic) correction scale:False

Estimate CSA gel shift (isotropic) correction scale:False

Gel Shift Correction Scale: 0.15

Single Tensor: False

Superimpose: False

Average methyl groups: False

Average methylene groups: False

Average phenyl groups: False

Bootstrapping: False

RDC Std. Error [ppm]: 1

CSA Std. Error [ppm]: 0.01

PCS Std. Error [ppm]: 0.01

DQ Std. Error [Hz]: 1

********

!* Permutations *!

There are 16 possible permutations on the original data set

********

Data set: #1

Computed data for frame #1

RDC Data:

I J Exp. [Hz] Comp. [Hz]

C5 H16 23.00 5.57

C12 H22 25.20 13.24

C14 H24 10.30 12.73

C14 H25 3.60 3.20

C26 H37 -19.00 -6.05

C33 H43 -23.00 -3.52

C31 H41 -21.90 -0.48

C29 H39 -23.90 -10.65

C28 H38 -19.50 -12.82

C35 H45 21.30 -7.13

C35 H46 -10.20 -5.33

C47 H58 -25.00 -21.06

C54 H64 -19.70 -14.15

C56 H66 0.00 2.25

C56 H67 2.90 11.33

C68 H79 -4.20 3.14

C75 H85 -1.20 -6.72

C73 H83 -5.00 -2.57

C71 H81 -5.30 4.70

C70 H80 -4.50 -10.88

C77 H87 -1.30 2.77

C77 H88 4.30 -15.65

Cornilescu Quality factor: 0.786894

Alignment tensor information:

A'x= 9.257e-04

A'y= 2.450e-03

A'z=-3.375e-03

Saupe tensor

S'x= 1.389e-03

S'y= 3.674e-03

S'z=-5.063e-03

Alignment tensor eigenvectors

e[x]=( 0.251,-0.617, 0.746)

e[y]=( 0.708,-0.409,-0.576)

e[z]=( 0.660, 0.672, 0.335)

Alignment tensor in laboratory coordinates:

[-1.866e-04,-2.350e-03,-1.572e-03]

[-2.350e-03,-7.636e-04,-6.086e-04]

[-1.572e-03,-6.086e-04,9.503e-04]

SVD condition number is 2.731e+01

Axial component Aa = -5.063e-03

Rhombic component Ar = -1.524e-03

Field=18.79 Teslas[ 2.27]

rhombicity R = 0.301

Asimmetry parameter etha =4.515e-01

GDO = 6.137e-03

ZY'Z'' Euler Angles (degrees)

Set 1

(45.5,70.5,-142.3)

Set 2

(-134.5,-70.5,37.7)

********Data set: #2

Computed data for frame #1

RDC Data:

I J Exp. [Hz] Comp. [Hz]

C5 H16 23.00 4.46

C12 H22 25.20 10.97

C14 H25 10.30 5.96

C14 H24 3.60 10.16

C26 H37 -19.00 -6.84

C33 H43 -23.00 -3.87

C31 H41 -21.90 -1.48

C29 H39 -23.90 -10.90

C28 H38 -19.50 -11.90

C35 H45 21.30 -6.72

C35 H46 -10.20 -7.00

C47 H58 -25.00 -20.26

C54 H64 -19.70 -14.52

C56 H66 0.00 4.06

C56 H67 2.90 10.26

C68 H79 -4.20 3.09

C75 H85 -1.20 -6.05

C73 H83 -5.00 -1.97

C71 H81 -5.30 3.88

C70 H80 -4.50 -10.57

C77 H87 -1.30 2.92

C77 H88 4.30 -17.28

Cornilescu Quality factor: 0.797856

Alignment tensor information:

A'x= 1.074e-03

A'y= 2.052e-03

A'z=-3.126e-03

Saupe tensor

S'x= 1.611e-03

S'y= 3.078e-03

S'z=-4.689e-03

Alignment tensor eigenvectors

e[x]=( 0.270,-0.629, 0.729)

e[y]=( 0.704,-0.387,-0.595)

e[z]=( 0.657, 0.674, 0.338)

Alignment tensor in laboratory coordinates:

[-2.526e-04,-2.126e-03,-1.342e-03]

[-2.126e-03,-6.880e-04,-7.316e-04]

[-1.342e-03,-7.316e-04,9.407e-04]

SVD condition number is 2.731e+01

Axial component Aa = -4.689e-03

Rhombic component Ar = -9.780e-04

Field=18.79 Teslas[ 2.27]

rhombicity R = 0.209

Asimmetry parameter etha =3.128e-01

GDO = 5.546e-03

ZY'Z'' Euler Angles (degrees)

Set 1

(45.8,70.2,-140.8)

Set 2

(-134.2,-70.2,39.2)

********Data set: #3

Computed data for frame #1

RDC Data:

I J Exp. [Hz] Comp. [Hz]

C5 H16 23.00 1.46

C12 H22 25.20 11.65

C14 H24 10.30 12.32

C14 H25 3.60 -6.98

C26 H37 -19.00 -8.57

C33 H43 -23.00 -8.33

C31 H41 -21.90 -3.55

C29 H39 -23.90 -14.13

C28 H38 -19.50 -20.13

C35 H46 21.30 12.71

C35 H45 -10.20 -13.90

C47 H58 -25.00 -25.94

C54 H64 -19.70 -16.32

C56 H66 0.00 -3.71

C56 H67 2.90 8.05

C68 H79 -4.20 4.42

C75 H85 -1.20 -5.20

C73 H83 -5.00 -2.10

C71 H81 -5.30 8.58

C70 H80 -4.50 -6.60

C77 H87 -1.30 10.60

C77 H88 4.30 -12.64

Cornilescu Quality factor: 0.671002

Alignment tensor information:

A'x= 6.302e-05

A'y= 3.364e-03

A'z=-3.427e-03

Saupe tensor

S'x= 9.453e-05

S'y= 5.046e-03

S'z=-5.141e-03

Alignment tensor eigenvectors

e[x]=( 0.213,-0.573, 0.791)

e[y]=( 0.710,-0.465,-0.528)

e[z]=( 0.671, 0.675, 0.308)

Alignment tensor in laboratory coordinates:

[ 1.584e-04,-2.671e-03,-1.959e-03]

[-2.671e-03,-8.101e-04,8.576e-05]

[-1.959e-03,8.576e-05,6.518e-04]

SVD condition number is 2.731e+01

Axial component Aa = -5.141e-03

Rhombic component Ar = -3.301e-03

Field=18.79 Teslas[ 2.27]

rhombicity R = 0.642

Asimmetry parameter etha =9.632e-01

GDO = 7.182e-03

ZY'Z'' Euler Angles (degrees)

Set 1

(45.2,72.1,-146.3)

Set 2

(-134.8,-72.1,33.7)

********Data set: #4

Computed data for frame #1

RDC Data:

I J Exp. [Hz] Comp. [Hz]

C5 H16 23.00 0.35

C12 H22 25.20 9.37

C14 H25 10.30 -4.21

C14 H24 3.60 9.76

C26 H37 -19.00 -9.36

C33 H43 -23.00 -8.67

C31 H41 -21.90 -4.56

C29 H39 -23.90 -14.38

C28 H38 -19.50 -19.21

C35 H46 21.30 11.04

C35 H45 -10.20 -13.49

C47 H58 -25.00 -25.15

C54 H64 -19.70 -16.70

C56 H66 0.00 -1.90

C56 H67 2.90 6.98

C68 H79 -4.20 4.37

C75 H85 -1.20 -4.53

C73 H83 -5.00 -1.50

C71 H81 -5.30 7.76

C70 H80 -4.50 -6.29

C77 H87 -1.30 10.75

C77 H88 4.30 -14.27

Cornilescu Quality factor: 0.701668

Alignment tensor information:

A'x= 2.131e-04

A'y= 2.964e-03

A'z=-3.177e-03

Saupe tensor

S'x= 3.197e-04

S'y= 4.446e-03

S'z=-4.766e-03

Alignment tensor eigenvectors

e[x]=( 0.209,-0.571, 0.794)

e[y]=( 0.713,-0.466,-0.524)

e[z]=( 0.669, 0.675, 0.310)

Alignment tensor in laboratory coordinates:

[ 9.241e-05,-2.447e-03,-1.730e-03]

[-2.447e-03,-7.346e-04,-3.726e-05]

[-1.730e-03,-3.726e-05,6.422e-04]

SVD condition number is 2.731e+01

Axial component Aa = -4.766e-03

Rhombic component Ar = -2.751e-03

Field=18.79 Teslas[ 2.27]

rhombicity R = 0.577

Asimmetry parameter etha =8.658e-01

GDO = 6.452e-03

ZY'Z'' Euler Angles (degrees)

Set 1

(45.3,72.0,-146.6)

Set 2

(-134.7,-72.0,33.4)

********Data set: #5

Computed data for frame #1

RDC Data:

I J Exp. [Hz] Comp. [Hz]

C5 H16 23.00 5.75

C12 H22 25.20 13.22

C14 H24 10.30 12.60

C14 H25 3.60 4.32

C26 H37 -19.00 -6.02

C33 H43 -23.00 -3.29

C31 H41 -21.90 -0.39

C29 H39 -23.90 -10.55

C28 H38 -19.50 -12.38

C35 H45 21.30 -6.72

C35 H46 -10.20 -5.17

C47 H58 -25.00 -20.86

C54 H64 -19.70 -14.17

C56 H67 0.00 9.67

C56 H66 2.90 3.14

C68 H79 -4.20 3.58

C75 H85 -1.20 -6.19

C73 H83 -5.00 -2.02

C71 H81 -5.30 4.96

C70 H80 -4.50 -10.49

C77 H87 -1.30 3.68

C77 H88 4.30 -16.11

Cornilescu Quality factor: 0.792306

Alignment tensor information:

A'x= 9.863e-04

A'y= 2.368e-03

A'z=-3.354e-03

Saupe tensor

S'x= 1.479e-03

S'y= 3.551e-03

S'z=-5.031e-03

Alignment tensor eigenvectors

e[x]=( 0.247,-0.618, 0.746)

e[y]=( 0.708,-0.411,-0.574)

e[z]=( 0.661, 0.671, 0.336)

Alignment tensor in laboratory coordinates:

[-2.200e-04,-2.327e-03,-1.526e-03]

[-2.327e-03,-7.324e-04,-6.516e-04]

[-1.526e-03,-6.516e-04,9.525e-04]

SVD condition number is 2.731e+01

Axial component Aa = -5.031e-03

Rhombic component Ar = -1.381e-03

Field=18.79 Teslas[ 2.27]

rhombicity R = 0.275

Asimmetry parameter etha =4.119e-01

GDO = 6.050e-03

ZY'Z'' Euler Angles (degrees)

Set 1

(45.4,70.4,-142.4)

Set 2

(-134.6,-70.4,37.6)

********Data set: #6

Computed data for frame #1

RDC Data:

I J Exp. [Hz] Comp. [Hz]

C5 H16 23.00 4.64

C12 H22 25.20 10.95

C14 H25 10.30 7.08

C14 H24 3.60 10.04

C26 H37 -19.00 -6.81

C33 H43 -23.00 -3.64

C31 H41 -21.90 -1.40

C29 H39 -23.90 -10.80

C28 H38 -19.50 -11.46

C35 H45 21.30 -6.31

C35 H46 -10.20 -6.84

C47 H58 -25.00 -20.06

C54 H64 -19.70 -14.54

C56 H67 0.00 8.60

C56 H66 2.90 4.95

C68 H79 -4.20 3.53

C75 H85 -1.20 -5.52

C73 H83 -5.00 -1.42

C71 H81 -5.30 4.15

C70 H80 -4.50 -10.18

C77 H87 -1.30 3.83

C77 H88 4.30 -17.74

Cornilescu Quality factor: 0.801225

Alignment tensor information:

A'x= 1.135e-03

A'y= 1.970e-03

A'z=-3.105e-03

Saupe tensor

S'x= 1.702e-03

S'y= 2.955e-03

S'z=-4.657e-03

Alignment tensor eigenvectors

e[x]=( 0.269,-0.631, 0.728)

e[y]=( 0.704,-0.388,-0.596)

e[z]=( 0.658, 0.672, 0.339)

Alignment tensor in laboratory coordinates:

[-2.860e-04,-2.103e-03,-1.296e-03]

[-2.103e-03,-6.569e-04,-7.746e-04]

[-1.296e-03,-7.746e-04,9.429e-04]

SVD condition number is 2.731e+01

Axial component Aa = -4.657e-03

Rhombic component Ar = -8.351e-04

Field=18.79 Teslas[ 2.27]

rhombicity R = 0.179

Asimmetry parameter etha =2.690e-01

GDO = 5.474e-03

ZY'Z'' Euler Angles (degrees)

Set 1

(45.6,70.2,-140.7)

Set 2

(-134.4,-70.2,39.3)

********Data set: #7

Computed data for frame #1

RDC Data:

I J Exp. [Hz] Comp. [Hz]

C5 H16 23.00 1.65

C12 H22 25.20 11.63

C14 H24 10.30 12.20

C14 H25 3.60 -5.85

C26 H37 -19.00 -8.54

C33 H43 -23.00 -8.09

C31 H41 -21.90 -3.47

C29 H39 -23.90 -14.02

C28 H38 -19.50 -19.69

C35 H46 21.30 12.86

C35 H45 -10.20 -13.50

C47 H58 -25.00 -25.74

C54 H64 -19.70 -16.34

C56 H67 0.00 6.39

C56 H66 2.90 -2.82

C68 H79 -4.20 4.86

C75 H85 -1.20 -4.68

C73 H83 -5.00 -1.55

C71 H81 -5.30 8.84

C70 H80 -4.50 -6.20

C77 H87 -1.30 11.51

C77 H88 4.30 -13.10

Cornilescu Quality factor: 0.679505

Alignment tensor information:

A'x= 1.219e-04

A'y= 3.284e-03

A'z=-3.406e-03

Saupe tensor

S'x= 1.828e-04

S'y= 4.926e-03

S'z=-5.109e-03

Alignment tensor eigenvectors

e[x]=( 0.208,-0.573, 0.793)

e[y]=( 0.711,-0.469,-0.525)

e[z]=( 0.672, 0.672, 0.310)

Alignment tensor in laboratory coordinates:

[ 1.250e-04,-2.648e-03,-1.914e-03]

[-2.648e-03,-7.790e-04,4.271e-05]

[-1.914e-03,4.271e-05,6.540e-04]

SVD condition number is 2.731e+01

Axial component Aa = -5.109e-03

Rhombic component Ar = -3.162e-03

Field=18.79 Teslas[ 2.27]

rhombicity R = 0.619

Asimmetry parameter etha =9.284e-01

GDO = 7.056e-03

ZY'Z'' Euler Angles (degrees)

Set 1

(45.0,72.0,-146.5)

Set 2

(-135.0,-72.0,33.5)

********Data set: #8

Computed data for frame #1

RDC Data:

I J Exp. [Hz] Comp. [Hz]

C5 H16 23.00 0.54

C12 H22 25.20 9.35

C14 H25 10.30 -3.09

C14 H24 3.60 9.63

C26 H37 -19.00 -9.33

C33 H43 -23.00 -8.44

C31 H41 -21.90 -4.47

C29 H39 -23.90 -14.28

C28 H38 -19.50 -18.78

C35 H46 21.30 11.19

C35 H45 -10.20 -13.09

C47 H58 -25.00 -24.95

C54 H64 -19.70 -16.71

C56 H67 0.00 5.31

C56 H66 2.90 -1.01

C68 H79 -4.20 4.81

C75 H85 -1.20 -4.01

C73 H83 -5.00 -0.95

C71 H81 -5.30 8.03

C70 H80 -4.50 -5.89

C77 H87 -1.30 11.66

C77 H88 4.30 -14.72

Cornilescu Quality factor: 0.707575

Alignment tensor information:

A'x= 2.719e-04

A'y= 2.884e-03

A'z=-3.155e-03

Saupe tensor

S'x= 4.078e-04

S'y= 4.325e-03

S'z=-4.733e-03

Alignment tensor eigenvectors

e[x]=( 0.203,-0.571, 0.796)

e[y]=( 0.713,-0.470,-0.520)

e[z]=( 0.671, 0.673, 0.311)

Alignment tensor in laboratory coordinates:

[ 5.902e-05,-2.424e-03,-1.684e-03]

[-2.424e-03,-7.034e-04,-8.031e-05]

[-1.684e-03,-8.031e-05,6.443e-04]

SVD condition number is 2.731e+01

Axial component Aa = -4.733e-03

Rhombic component Ar = -2.612e-03

Field=18.79 Teslas[ 2.27]

rhombicity R = 0.552

Asimmetry parameter etha =8.277e-01

GDO = 6.333e-03

ZY'Z'' Euler Angles (degrees)

Set 1

(45.1,71.9,-146.9)

Set 2

(-134.9,-71.9,33.1)

********Data set: #9

Computed data for frame #1

RDC Data:

I J Exp. [Hz] Comp. [Hz]

C5 H16 23.00 5.83

C12 H22 25.20 13.39

C14 H24 10.30 12.57

C14 H25 3.60 4.53

C26 H37 -19.00 -6.83

C33 H43 -23.00 -3.64

C31 H41 -21.90 -0.81

C29 H39 -23.90 -11.50

C28 H38 -19.50 -12.93

C35 H45 21.30 -7.02

C35 H46 -10.20 -4.36

C47 H58 -25.00 -22.22

C54 H64 -19.70 -15.50

C56 H66 0.00 2.91

C56 H67 2.90 9.34

C68 H79 -4.20 2.70

C75 H85 -1.20 -7.52

C73 H83 -5.00 -3.07

C71 H81 -5.30 3.90

C70 H80 -4.50 -12.26

C77 H88 -1.30 -18.20

C77 H87 4.30 5.98

Cornilescu Quality factor: 0.75773

Alignment tensor information:

A'x= 1.125e-03

A'y= 2.395e-03

A'z=-3.520e-03

Saupe tensor

S'x= 1.687e-03

S'y= 3.593e-03

S'z=-5.280e-03

Alignment tensor eigenvectors

e[x]=( 0.250,-0.617, 0.746)

e[y]=( 0.706,-0.411,-0.576)

e[z]=( 0.662, 0.671, 0.333)

Alignment tensor in laboratory coordinates:

[-2.778e-04,-2.433e-03,-1.542e-03]

[-2.433e-03,-7.534e-04,-7.382e-04]

[-1.542e-03,-7.382e-04,1.031e-03]

SVD condition number is 2.731e+01

Axial component Aa = -5.280e-03

Rhombic component Ar = -1.270e-03

Field=18.79 Teslas[ 2.27]

rhombicity R = 0.241

Asimmetry parameter etha =3.609e-01

GDO = 6.292e-03

ZY'Z'' Euler Angles (degrees)

Set 1

(45.4,70.5,-142.3)

Set 2

(-134.6,-70.5,37.7)

********Data set: #10

Computed data for frame #1

RDC Data:

I J Exp. [Hz] Comp. [Hz]

C5 H16 23.00 4.72

C12 H22 25.20 11.11

C14 H25 10.30 7.29

C14 H24 3.60 10.01

C26 H37 -19.00 -7.62

C33 H43 -23.00 -3.99

C31 H41 -21.90 -1.82

C29 H39 -23.90 -11.75

C28 H38 -19.50 -12.01

C35 H45 21.30 -6.61

C35 H46 -10.20 -6.03

C47 H58 -25.00 -21.43

C54 H64 -19.70 -15.87

C56 H66 0.00 4.72

C56 H67 2.90 8.27

C68 H79 -4.20 2.65

C75 H85 -1.20 -6.85

C73 H83 -5.00 -2.46

C71 H81 -5.30 3.08

C70 H80 -4.50 -11.94

C77 H88 -1.30 -19.82

C77 H87 4.30 6.13

Cornilescu Quality factor: 0.766665

Alignment tensor information:

A'x= 1.273e-03

A'y= 1.998e-03

A'z=-3.271e-03

Saupe tensor

S'x= 1.910e-03

S'y= 2.996e-03

S'z=-4.906e-03

Alignment tensor eigenvectors

e[x]=( 0.276,-0.632, 0.724)

e[y]=( 0.700,-0.384,-0.602)

e[z]=( 0.659, 0.673, 0.336)

Alignment tensor in laboratory coordinates:

[-3.437e-04,-2.209e-03,-1.312e-03]

[-2.209e-03,-6.778e-04,-8.612e-04]

[-1.312e-03,-8.612e-04,1.022e-03]

SVD condition number is 2.731e+01

Axial component Aa = -4.906e-03

Rhombic component Ar = -7.244e-04

Field=18.79 Teslas[ 2.27]

rhombicity R = 0.148

Asimmetry parameter etha =2.215e-01

GDO = 5.734e-03

ZY'Z'' Euler Angles (degrees)

Set 1

(45.6,70.3,-140.2)

Set 2

(-134.4,-70.3,39.8)

********Data set: #11

Computed data for frame #1

RDC Data:

I J Exp. [Hz] Comp. [Hz]

C5 H16 23.00 1.72

C12 H22 25.20 11.79

C14 H24 10.30 12.17

C14 H25 3.60 -5.65

C26 H37 -19.00 -9.35

C33 H43 -23.00 -8.45

C31 H41 -21.90 -3.89

C29 H39 -23.90 -14.98

C28 H38 -19.50 -20.25

C35 H46 21.30 13.67

C35 H45 -10.20 -13.79

C47 H58 -25.00 -27.10

C54 H64 -19.70 -17.67

C56 H66 0.00 -3.05

C56 H67 2.90 6.06

C68 H79 -4.20 3.98

C75 H85 -1.20 -6.00

C73 H83 -5.00 -2.60

C71 H81 -5.30 7.78

C70 H80 -4.50 -7.97

C77 H88 -1.30 -15.19

C77 H87 4.30 13.81

Cornilescu Quality factor: 0.628501

Alignment tensor information:

A'x= 2.605e-04

A'y= 3.312e-03

A'z=-3.572e-03

Saupe tensor

S'x= 3.908e-04

S'y= 4.968e-03

S'z=-5.358e-03

Alignment tensor eigenvectors

e[x]=( 0.207,-0.571, 0.794)

e[y]=( 0.710,-0.470,-0.524)

e[z]=( 0.673, 0.673, 0.308)

Alignment tensor in laboratory coordinates:

[ 6.726e-05,-2.754e-03,-1.930e-03]

[-2.754e-03,-7.999e-04,-4.386e-05]

[-1.930e-03,-4.386e-05,7.326e-04]

SVD condition number is 2.731e+01

Axial component Aa = -5.358e-03

Rhombic component Ar = -3.051e-03

Field=18.79 Teslas[ 2.27]

rhombicity R = 0.569

Asimmetry parameter etha =8.541e-01

GDO = 7.228e-03

ZY'Z'' Euler Angles (degrees)

Set 1

(45.0,72.0,-146.6)

Set 2

(-135.0,-72.0,33.4)

********Data set: #12

Computed data for frame #1

RDC Data:

I J Exp. [Hz] Comp. [Hz]

C5 H16 23.00 0.61

C12 H22 25.20 9.51

C14 H25 10.30 -2.89

C14 H24 3.60 9.60

C26 H37 -19.00 -10.15

C33 H43 -23.00 -8.80

C31 H41 -21.90 -4.89

C29 H39 -23.90 -15.23

C28 H38 -19.50 -19.33

C35 H46 21.30 12.00

C35 H45 -10.20 -13.38

C47 H58 -25.00 -26.31

C54 H64 -19.70 -18.04

C56 H66 0.00 -1.24

C56 H67 2.90 4.99

C68 H79 -4.20 3.93

C75 H85 -1.20 -5.33

C73 H83 -5.00 -1.99

C71 H81 -5.30 6.96

C70 H80 -4.50 -7.66

C77 H88 -1.30 -16.81

C77 H87 4.30 13.96

Cornilescu Quality factor: 0.658298

Alignment tensor information:

A'x= 4.104e-04

A'y= 2.912e-03

A'z=-3.322e-03

Saupe tensor

S'x= 6.157e-04

S'y= 4.367e-03

S'z=-4.983e-03

Alignment tensor eigenvectors

e[x]=( 0.202,-0.569, 0.797)

e[y]=( 0.713,-0.472,-0.518)

e[z]=( 0.671, 0.673, 0.310)

Alignment tensor in laboratory coordinates:

[ 1.292e-06,-2.530e-03,-1.700e-03]

[-2.530e-03,-7.243e-04,-1.669e-04]

[-1.700e-03,-1.669e-04,7.230e-04]

SVD condition number is 2.731e+01

Axial component Aa = -4.983e-03

Rhombic component Ar = -2.501e-03

Field=18.79 Teslas[ 2.27]

rhombicity R = 0.502

Asimmetry parameter etha =7.529e-01

GDO = 6.519e-03

ZY'Z'' Euler Angles (degrees)

Set 1

(45.1,72.0,-147.0)

Set 2

(-134.9,-72.0,33.0)

********Data set: #13

Computed data for frame #1

RDC Data:

I J Exp. [Hz] Comp. [Hz]

C5 H16 23.00 6.02

C12 H22 25.20 13.36

C14 H24 10.30 12.45

C14 H25 3.60 5.65

C26 H37 -19.00 -6.80

C33 H43 -23.00 -3.41

C31 H41 -21.90 -0.72

C29 H39 -23.90 -11.40

C28 H38 -19.50 -12.49

C35 H45 21.30 -6.62

C35 H46 -10.20 -4.21

C47 H58 -25.00 -22.02

C54 H64 -19.70 -15.52

C56 H67 0.00 7.68

C56 H66 2.90 3.80

C68 H79 -4.20 3.14

C75 H85 -1.20 -6.99

C73 H83 -5.00 -2.52

C71 H81 -5.30 4.16

C70 H80 -4.50 -11.86

C77 H88 -1.30 -18.66

C77 H87 4.30 6.89

Cornilescu Quality factor: 0.761447

Alignment tensor information:

A'x= 1.185e-03

A'y= 2.313e-03

A'z=-3.498e-03

Saupe tensor

S'x= 1.778e-03

S'y= 3.470e-03

S'z=-5.248e-03

Alignment tensor eigenvectors

e[x]=( 0.246,-0.617, 0.747)

e[y]=( 0.707,-0.413,-0.574)

e[z]=( 0.663, 0.670, 0.334)

Alignment tensor in laboratory coordinates:

[-3.111e-04,-2.409e-03,-1.496e-03]

[-2.409e-03,-7.222e-04,-7.812e-04]

[-1.496e-03,-7.812e-04,1.033e-03]

SVD condition number is 2.731e+01

Axial component Aa = -5.248e-03

Rhombic component Ar = -1.128e-03

Field=18.79 Teslas[ 2.27]

rhombicity R = 0.215

Asimmetry parameter etha =3.223e-01

GDO = 6.215e-03

ZY'Z'' Euler Angles (degrees)

Set 1

(45.3,70.5,-142.5)

Set 2

(-134.7,-70.5,37.5)

********Data set: #14

Computed data for frame #1

RDC Data:

I J Exp. [Hz] Comp. [Hz]

C5 H16 23.00 4.90

C12 H22 25.20 11.09

C14 H25 10.30 8.41

C14 H24 3.60 9.88

C26 H37 -19.00 -7.59

C33 H43 -23.00 -3.76

C31 H41 -21.90 -1.73

C29 H39 -23.90 -11.65

C28 H38 -19.50 -11.58

C35 H45 21.30 -6.21

C35 H46 -10.20 -5.88

C47 H58 -25.00 -21.23

C54 H64 -19.70 -15.89

C56 H67 0.00 6.61

C56 H66 2.90 5.61

C68 H79 -4.20 3.10

C75 H85 -1.20 -6.32

C73 H83 -5.00 -1.91

C71 H81 -5.30 3.35

C70 H80 -4.50 -11.55

C77 H88 -1.30 -20.28

C77 H87 4.30 7.04

Cornilescu Quality factor: 0.768286

Alignment tensor information:

A'x= 1.334e-03

A'y= 1.915e-03

A'z=-3.249e-03

Saupe tensor

S'x= 2.001e-03

S'y= 2.873e-03

S'z=-4.874e-03

Alignment tensor eigenvectors

e[x]=( 0.276,-0.635, 0.722)

e[y]=( 0.699,-0.383,-0.604)

e[z]=( 0.660, 0.671, 0.338)

Alignment tensor in laboratory coordinates:

[-3.771e-04,-2.186e-03,-1.266e-03]

[-2.186e-03,-6.466e-04,-9.043e-04]

[-1.266e-03,-9.043e-04,1.024e-03]

SVD condition number is 2.731e+01

Axial component Aa = -4.874e-03

Rhombic component Ar = -5.816e-04

Field=18.79 Teslas[ 2.27]

rhombicity R = 0.119

Asimmetry parameter etha =1.790e-01

GDO = 5.673e-03

ZY'Z'' Euler Angles (degrees)

Set 1

(45.5,70.3,-140.1)

Set 2

(-134.5,-70.3,39.9)

********Data set: #15

Computed data for frame #1

RDC Data:

I J Exp. [Hz] Comp. [Hz]

C5 H16 23.00 1.91

C12 H22 25.20 11.77

C14 H24 10.30 12.04

C14 H25 3.60 -4.53

C26 H37 -19.00 -9.33

C33 H43 -23.00 -8.22

C31 H41 -21.90 -3.80

C29 H39 -23.90 -14.87

C28 H38 -19.50 -19.81

C35 H46 21.30 13.83

C35 H45 -10.20 -13.39

C47 H58 -25.00 -26.90

C54 H64 -19.70 -17.69

C56 H67 0.00 4.40

C56 H66 2.90 -2.16

C68 H79 -4.20 4.42

C75 H85 -1.20 -5.48

C73 H83 -5.00 -2.05

C71 H81 -5.30 8.04

C70 H80 -4.50 -7.58

C77 H88 -1.30 -15.65

C77 H87 4.30 14.72

Cornilescu Quality factor: 0.635293

Alignment tensor information:

A'x= 3.192e-04

A'y= 3.232e-03

A'z=-3.551e-03

Saupe tensor

S'x= 4.788e-04

S'y= 4.847e-03

S'z=-5.326e-03

Alignment tensor eigenvectors

e[x]=(-0.202, 0.570,-0.796)

e[y]=(-0.711, 0.474, 0.520)

e[z]=( 0.674, 0.671, 0.310)

Alignment tensor in laboratory coordinates:

[ 3.388e-05,-2.730e-03,-1.884e-03]

[-2.730e-03,-7.687e-04,-8.691e-05]

[-1.884e-03,-8.691e-05,7.348e-04]

SVD condition number is 2.731e+01

Axial component Aa = -5.326e-03

Rhombic component Ar = -2.912e-03

Field=18.79 Teslas[ 2.27]

rhombicity R = 0.547

Asimmetry parameter etha =8.202e-01

GDO = 7.110e-03

ZY'Z'' Euler Angles (degrees)

Set 1

(44.9,72.0,33.1)

Set 2

(-135.1,-72.0,-146.9)

********Data set: #16

Computed data for frame #1

RDC Data:

I J Exp. [Hz] Comp. [Hz]

C5 H16 23.00 0.80

C12 H22 25.20 9.49

C14 H25 10.30 -1.76

C14 H24 3.60 9.48

C26 H37 -19.00 -10.12

C33 H43 -23.00 -8.56

C31 H41 -21.90 -4.81

C29 H39 -23.90 -15.13

C28 H38 -19.50 -18.89

C35 H46 21.30 12.16

C35 H45 -10.20 -12.98

C47 H58 -25.00 -26.11

C54 H64 -19.70 -18.06

C56 H67 0.00 3.32

C56 H66 2.90 -0.35

C68 H79 -4.20 4.38

C75 H85 -1.20 -4.81

C73 H83 -5.00 -1.44

C71 H81 -5.30 7.22

C70 H80 -4.50 -7.27

C77 H88 -1.30 -17.27

C77 H87 4.30 14.87

Cornilescu Quality factor: 0.662406

Alignment tensor information:

A'x= 4.689e-04

A'y= 2.832e-03

A'z=-3.300e-03

Saupe tensor

S'x= 7.034e-04

S'y= 4.247e-03

S'z=-4.951e-03

Alignment tensor eigenvectors

e[x]=(-0.196, 0.567,-0.800)

e[y]=(-0.714, 0.477, 0.513)

e[z]=( 0.672, 0.671, 0.311)

Alignment tensor in laboratory coordinates:

[-3.209e-05,-2.506e-03,-1.654e-03]

[-2.506e-03,-6.931e-04,-2.099e-04]

[-1.654e-03,-2.099e-04,7.252e-04]

SVD condition number is 2.731e+01

Axial component Aa = -4.951e-03

Rhombic component Ar = -2.363e-03

Field=18.79 Teslas[ 2.27]

rhombicity R = 0.477

Asimmetry parameter etha =7.158e-01

GDO = 6.407e-03

ZY'Z'' Euler Angles (degrees)

Set 1

(45.0,71.9,32.7)

Set 2

(-135.0,-71.9,-147.3)

********

MSpin-RDC pluginmi. jul. 28 12:42:02 2021

gt2/gg2/gt2

CR_tr

!* MSpin-RDC Plugin *!

********

!* Computation flags *!

Method: SVD

Scaling mode: Hz

Field (T): 18.7923

1H Larmor Frequency: 800.13

Scale QCSA with axial component: False

Include CSA gel shift (isotropic) correction:False

Optimize CSA gel shift (isotropic) correction scale:False

Estimate CSA gel shift (isotropic) correction scale:False

Gel Shift Correction Scale: 0.15

Single Tensor: False

Superimpose: False

Average methyl groups: False

Average methylene groups: False

Average phenyl groups: False

Bootstrapping: False

RDC Std. Error [ppm]: 1

CSA Std. Error [ppm]: 0.01

PCS Std. Error [ppm]: 0.01

DQ Std. Error [Hz]: 1

********

!* Permutations *!

There are no permutations on the original data set

********

Data set: #1

Computed data for frame #1

RDC Data:

I J Exp. [Hz] Comp. [Hz]

C5 H16 23.00 6.62

C12 H22 25.20 14.55

C26 H37 -19.00 -13.97

C33 H43 -23.00 -6.66

C31 H41 -21.90 -4.43

C29 H39 -23.90 -20.04

C28 H38 -19.50 -17.76

C47 H58 -25.00 -35.12

C54 H64 -19.70 -27.39

C68 H79 -4.20 -3.61

C75 H85 -1.20 -6.96

C73 H83 -5.00 -7.29

C71 H81 -5.30 1.53

C70 H80 -4.50 -3.71

Cornilescu Quality factor: 0.520714

Alignment tensor information:

A'x=-2.942e-04

A'y=-3.830e-03

A'z= 4.124e-03

Saupe tensor

S'x=-4.413e-04

S'y=-5.745e-03

S'z= 6.186e-03

Alignment tensor eigenvectors

e[x]=( 0.136,-0.160, 0.978)

e[y]=( 0.820, 0.572,-0.021)

e[z]=(-0.556, 0.805, 0.209)

Alignment tensor in laboratory coordinates:

[-1.306e-03,-3.634e-03,-4.533e-04]

[-3.634e-03,1.409e-03,7.850e-04]

[-4.533e-04,7.850e-04,-1.026e-04]

SVD condition number is 6.492e+01

Axial component Aa = 6.186e-03

Rhombic component Ar = 3.536e-03

Field=18.79 Teslas[ 2.27]

rhombicity R = 0.572

Asimmetry parameter etha =8.573e-01

GDO = 8.353e-03

ZY'Z'' Euler Angles (degrees)

Set 1

(124.6,77.9,-178.8)

Set 2

(-55.4,-77.9,1.2)

********

MSpin-RDC pluginmi. jul. 28 12:44:30 2021

gt2/gg2/gt2

E5C12

!* MSpin-RDC Plugin *!

********

!* Computation flags *!

Method: SVD

Scaling mode: Hz

Field (T): 18.7923

1H Larmor Frequency: 800.13

Scale QCSA with axial component: False

Include CSA gel shift (isotropic) correction:False

Optimize CSA gel shift (isotropic) correction scale:False

Estimate CSA gel shift (isotropic) correction scale:False

Gel Shift Correction Scale: 0.15

Single Tensor: False

Superimpose: False

Average methyl groups: False

Average methylene groups: False

Average phenyl groups: False

Bootstrapping: False

RDC Std. Error [ppm]: 1

CSA Std. Error [ppm]: 0.01

PCS Std. Error [ppm]: 0.01

DQ Std. Error [Hz]: 1

********

!* Permutations *!

There are no permutations on the original data set

********

Data set: #1

Computed data for frame #1

RDC Data:

I J Exp. [Hz] Comp. [Hz]

C5 H16 11.80 12.35

C12 H22 12.70 10.81

C26 H37 10.50 9.34

C33 H43 10.60 11.95

C47 H58 9.90 9.68

C54 H64 9.00 7.93

C68 H79 4.90 7.24

C75 H85 4.80 6.70

C71 H81 5.30 5.35

C70 H80 8.50 4.39

C77 H87 0.00 0.40

C77 H88 0.00 2.61

Cornilescu Quality factor: 0.220156

Alignment tensor information:

A'x=-1.955e-04

A'y=-7.174e-04

A'z= 9.129e-04

Saupe tensor

S'x=-2.933e-04

S'y=-1.076e-03

S'z= 1.369e-03

Alignment tensor eigenvectors

e[x]=( 0.659, 0.547, 0.516)

e[y]=( 0.748,-0.410,-0.521)

e[z]=(-0.074, 0.730,-0.680)

Alignment tensor in laboratory coordinates:

[-4.817e-04,1.005e-04,2.592e-04]

[ 1.005e-04,3.066e-04,-6.614e-04]

[ 2.592e-04,-6.614e-04,1.751e-04]

SVD condition number is 3.474e+01

Axial component Aa = 1.369e-03

Rhombic component Ar = 5.218e-04

Field=18.79 Teslas[ 2.27]

rhombicity R = 0.381

Asimmetry parameter etha =5.716e-01

GDO = 1.705e-03

ZY'Z'' Euler Angles (degrees)

Set 1

(95.8,132.8,-134.7)

Set 2

(-84.2,-132.8,45.3)

********

MSpin-RDC pluginmi. jul. 28 13:33:43 2021

gt2/gg2/gt2F

CRF_all

!* MSpin-RDC Plugin *!

********

!* Computation flags *!

Method: SVD

Scaling mode: Hz

Field (T): 18.7923

1H Larmor Frequency: 800.13

Scale QCSA with axial component: False

Include CSA gel shift (isotropic) correction:False

Optimize CSA gel shift (isotropic) correction scale:False

Estimate CSA gel shift (isotropic) correction scale:False

Gel Shift Correction Scale: 0.15

Single Tensor: False

Superimpose: False

Average methyl groups: False

Average methylene groups: False

Average phenyl groups: False

Bootstrapping: False

RDC Std. Error [ppm]: 1

CSA Std. Error [ppm]: 0.01

PCS Std. Error [ppm]: 0.01

DQ Std. Error [Hz]: 1

********

!* Permutations *!

There are 4 possible permutations on the original data set

********

Data set: #1

Computed data for frame #1

RDC Data:

I J Exp. [Hz] Comp. [Hz]

C5 H16 18.00 6.45

C10 H20 23.00 18.11

C10 F11 -13.00 0.80

C25 H36 -19.00 -9.57

C32 H42 -23.00 -6.50

C30 H40 -21.90 -4.13

C28 H38 -23.90 -13.60

C27 H37 -19.50 -14.43

C34 H44 21.30 -9.02

C34 H45 -10.20 -7.39

C46 H57 -30.00 -22.65

C51 H61 -24.00 -13.04

C51 F52 13.00 14.49

C66 H77 -4.20 2.96

C73 H83 -1.20 -6.20

C71 H81 -5.00 -1.95

C69 H79 -5.30 4.03

C68 H78 -4.50 -10.84

C75 H85 -1.30 2.35

C75 H86 4.30 -20.54

Cornilescu Quality factor: 0.740538

Alignment tensor information:

A'x= 1.126e-03

A'y= 2.091e-03

A'z=-3.217e-03

Saupe tensor

S'x= 1.689e-03

S'y= 3.137e-03

S'z=-4.826e-03

Alignment tensor eigenvectors

e[x]=( 0.286,-0.628, 0.724)

e[y]=( 0.703,-0.376,-0.604)

e[z]=( 0.652, 0.681, 0.334)

Alignment tensor in laboratory coordinates:

[-2.426e-04,-2.183e-03,-1.354e-03]

[-2.183e-03,-7.515e-04,-7.682e-04]

[-1.354e-03,-7.682e-04,9.941e-04]

SVD condition number is 2.432e+01

Axial component Aa = -4.826e-03

Rhombic component Ar = -9.648e-04

Field=18.79 Teslas[ 2.27]

rhombicity R = 0.200

Asimmetry parameter etha =2.999e-01

GDO = 5.697e-03

ZY'Z'' Euler Angles (degrees)

Set 1

(46.3,70.5,-140.2)

Set 2

(-133.7,-70.5,39.8)

********Data set: #2

Computed data for frame #1

RDC Data:

I J Exp. [Hz] Comp. [Hz]

C5 H16 18.00 5.37

C10 H20 23.00 16.48

C10 F11 -13.00 6.54

C25 H36 -19.00 -10.75

C32 H42 -23.00 -11.63

C30 H40 -21.90 -6.62

C28 H38 -23.90 -16.14

C27 H37 -19.50 -23.08

C34 H45 21.30 17.72

C34 H44 -10.20 -17.06

C46 H57 -30.00 -25.46

C51 H61 -24.00 -15.98

C51 F52 13.00 16.98

C66 H77 -4.20 2.03

C73 H83 -1.20 -6.57

C71 H81 -5.00 -4.09

C69 H79 -5.30 7.17

C68 H78 -4.50 -6.66

C75 H85 -1.30 11.75

C75 H86 4.30 -13.28

Cornilescu Quality factor: 0.587966

Alignment tensor information:

A'x=-3.020e-04

A'y=-3.176e-03

A'z= 3.478e-03

Saupe tensor

S'x=-4.531e-04

S'y=-4.765e-03

S'z= 5.218e-03

Alignment tensor eigenvectors

e[x]=(-0.240, 0.550,-0.800)

e[y]=( 0.665, 0.694, 0.278)

e[z]=( 0.708,-0.465,-0.532)

Alignment tensor in laboratory coordinates:

[ 3.211e-04,-2.569e-03,-1.954e-03]

[-2.569e-03,-8.671e-04,3.813e-04]

[-1.954e-03,3.813e-04,5.460e-04]

SVD condition number is 2.432e+01

Axial component Aa = 5.218e-03

Rhombic component Ar = 2.874e-03

Field=18.79 Teslas[ 2.27]

rhombicity R = 0.551

Asimmetry parameter etha =8.263e-01

GDO = 6.978e-03

ZY'Z'' Euler Angles (degrees)

Set 1

(-33.3,122.1,19.2)

Set 2

(146.7,-122.1,-160.8)

********Data set: #3

Computed data for frame #1

RDC Data:

I J Exp. [Hz] Comp. [Hz]

C5 H16 18.00 6.31

C10 H20 23.00 18.69

C10 F11 -13.00 -1.65

C25 H36 -19.00 -10.54

C32 H42 -23.00 -6.60

C30 H40 -21.90 -4.58

C28 H38 -23.90 -14.56

C27 H37 -19.50 -14.32

C34 H44 21.30 -8.73

C34 H45 -10.20 -6.72

C46 H57 -30.00 -24.05

C51 H61 -24.00 -13.89

C51 F52 13.00 13.11

C66 H77 -4.20 2.96

C73 H83 -1.20 -6.51

C71 H81 -5.00 -1.91

C69 H79 -5.30 3.49

C68 H78 -4.50 -11.90

C75 H86 -1.30 -23.58

C75 H85 4.30 5.88

Cornilescu Quality factor: 0.704692

Alignment tensor information:

A'x= 1.392e-03

A'y= 1.958e-03

A'z=-3.350e-03

Saupe tensor

S'x= 2.087e-03

S'y= 2.938e-03

S'z=-5.025e-03

Alignment tensor eigenvectors

e[x]=( 0.303,-0.640, 0.706)

e[y]=( 0.693,-0.360,-0.625)

e[z]=( 0.654, 0.679, 0.335)

Alignment tensor in laboratory coordinates:

[-3.640e-04,-2.245e-03,-1.283e-03]

[-2.245e-03,-7.179e-04,-9.490e-04]

[-1.283e-03,-9.490e-04,1.082e-03]

SVD condition number is 2.432e+01

Axial component Aa = -5.025e-03

Rhombic component Ar = -5.668e-04

Field=18.79 Teslas[ 2.27]

rhombicity R = 0.113

Asimmetry parameter etha =1.692e-01

GDO = 5.844e-03

ZY'Z'' Euler Angles (degrees)

Set 1

(46.1,70.5,-138.5)

Set 2

(-133.9,-70.5,41.5)

********Data set: #4

Computed data for frame #1

RDC Data:

I J Exp. [Hz] Comp. [Hz]

C5 H16 18.00 5.24

C10 H20 23.00 17.06

C10 F11 -13.00 4.09

C25 H36 -19.00 -11.72

C32 H42 -23.00 -11.73

C30 H40 -21.90 -7.07

C28 H38 -23.90 -17.10

C27 H37 -19.50 -22.98

C34 H45 21.30 18.39

C34 H44 -10.20 -16.77

C46 H57 -30.00 -26.87

C51 H61 -24.00 -16.83

C51 F52 13.00 15.60

C66 H77 -4.20 2.03

C73 H83 -1.20 -6.87

C71 H81 -5.00 -4.04

C69 H79 -5.30 6.63

C68 H78 -4.50 -7.71

C75 H86 -1.30 -16.32

C75 H85 4.30 15.28

Cornilescu Quality factor: 0.538197

Alignment tensor information:

A'x=-3.954e-05

A'y=-3.308e-03

A'z= 3.348e-03

Saupe tensor

S'x=-5.931e-05

S'y=-4.962e-03

S'z= 5.021e-03

Alignment tensor eigenvectors

e[x]=(-0.228, 0.553,-0.802)

e[y]=( 0.668, 0.688, 0.284)

e[z]=( 0.708,-0.471,-0.526)

Alignment tensor in laboratory coordinates:

[ 1.996e-04,-2.631e-03,-1.883e-03]

[-2.631e-03,-8.335e-04,2.005e-04]

[-1.883e-03,2.005e-04,6.339e-04]

SVD condition number is 2.432e+01

Axial component Aa = 5.021e-03

Rhombic component Ar = 3.269e-03

Field=18.79 Teslas[ 2.27]

rhombicity R = 0.651

Asimmetry parameter etha =9.764e-01

GDO = 7.046e-03

ZY'Z'' Euler Angles (degrees)

Set 1

(-33.6,121.7,19.5)

Set 2

(146.4,-121.7,-160.5)

********

MSpin-RDC pluginmi. jul. 28 13:38:01 2021

gt2/gg2/gt2F

CRF_tr

!* MSpin-RDC Plugin *!

********

!* Computation flags *!

Method: SVD

Scaling mode: Hz

Field (T): 18.7923

1H Larmor Frequency: 800.13

Scale QCSA with axial component: False

Include CSA gel shift (isotropic) correction:False

Optimize CSA gel shift (isotropic) correction scale:False

Estimate CSA gel shift (isotropic) correction scale:False

Gel Shift Correction Scale: 0.15

Single Tensor: False

Superimpose: False

Average methyl groups: False

Average methylene groups: False

Average phenyl groups: False

Bootstrapping: False

RDC Std. Error [ppm]: 1

CSA Std. Error [ppm]: 0.01

PCS Std. Error [ppm]: 0.01

DQ Std. Error [Hz]: 1

********

!* Permutations *!

There are no permutations on the original data set

********

Data set: #1

Computed data for frame #1

RDC Data:

I J Exp. [Hz] Comp. [Hz]

C5 H16 18.00 4.61

C10 H20 23.00 20.20

C10 F11 -13.00 -9.01

C25 H36 -19.00 -17.09

C32 H42 -23.00 -13.69

C30 H40 -21.90 -9.98

C28 H38 -23.90 -22.87

C27 H37 -19.50 -25.14

C46 H57 -30.00 -35.29

C51 H61 -24.00 -22.18

C51 F52 13.00 7.00

C66 H77 -4.20 1.15

C73 H83 -1.20 -9.02

C71 H81 -5.00 -5.07

C69 H79 -5.30 4.40

C68 H78 -4.50 -12.05

Cornilescu Quality factor: 0.390955

Alignment tensor information:

A'x= 8.133e-04

A'y= 3.188e-03

A'z=-4.001e-03

Saupe tensor

S'x= 1.220e-03

S'y= 4.782e-03

S'z=-6.002e-03

Alignment tensor eigenvectors

e[x]=(-0.149, 0.508,-0.848)

e[y]=(-0.708, 0.544, 0.450)

e[z]=( 0.690, 0.668, 0.279)

Alignment tensor in laboratory coordinates:

[-2.873e-04,-3.134e-03,-1.683e-03]

[-3.134e-03,-6.320e-04,-3.159e-04]

[-1.683e-03,-3.159e-04,9.193e-04]

SVD condition number is 4.675e+01

Axial component Aa = -6.002e-03

Rhombic component Ar = -2.375e-03

Field=18.79 Teslas[ 2.27]

rhombicity R = 0.396

Asimmetry parameter etha =5.935e-01

GDO = 7.516e-03

ZY'Z'' Euler Angles (degrees)

Set 1

(44.1,73.8,27.9)

Set 2

(-135.9,-73.8,-152.1)

********

MSpin-RDC pluginmi. jul. 28 13:40:36 2021
